# Photochemical Insights on Acyl Azolium Salts Enable the Design of a Tandem Hydrogen Atom Transfer/Halogen Atom Transfer Acylation of Alkyl Bromides and Chlorides

**DOI:** 10.1021/jacs.5c10923

**Published:** 2025-08-14

**Authors:** Ian MacLean, Daniel Jan Grenda, Elena Echávarri, Stephan Muth, Patrick Nuernberger, Leyre Marzo

**Affiliations:** † Organic Chemistry Department (Módulo 1), 16722 Universidad Autónoma de Madrid, C/Francisco Tomas y Valiente 7, 28049 Madrid, Spain; ‡ Institut für Physikalische und Theoretische Chemie, Fakultät für Chemie und Pharmazie, 9147 Universität Regensburg, 93040 Regensburg, Germany; § Institute for Advanced Research in Chemical Sciences (IAdChem) Universidad Autónoma de Madrid, 28049 Madrid, Spain

## Abstract

Acyl azolium salts have been recently described as good HAT mediators in their excited state-similarly to diaryl ketones-and good acylating reagents, with the beneficial property of enabling incorporation of the acyl residue into the final molecule. Nevertheless, despite significant synthetic efforts in the field, knowledge and understanding of the key reactive intermediates—especially through spectroscopic investigations—remain elusive. Herein, we present a photochemical study of the reactivity of acyl azolium salts that comprises the detection and characterization of the triplet excited state and the decisive ketyl radical intermediate. Moreover, this mechanistic insight allowed us the development of an alternative method based on a silane-mediated tandem HAT/XAT activation strategy that enables not only the acylation of alkyl bromides but also the acylation of the more challenging alkyl chlorides. The method has proven to be efficient, regardless of the electronic properties of the acyl azolium or the substituents present in the alkyl halide. Furthermore, its robustness has been proven through the functionalization of natural product derivatives either as acyl azolium or alkyl bromide derivatives.

## Graphical abstract

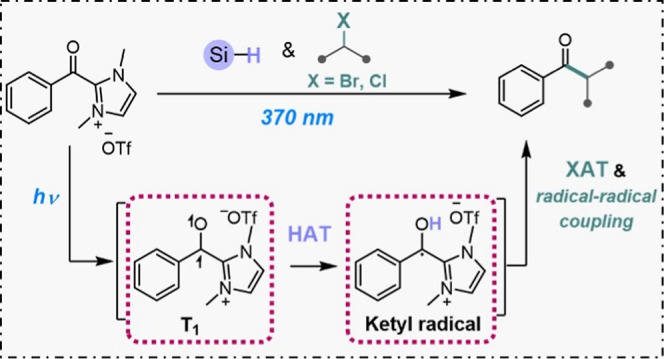

## Introduction

The photochemistry of ketones has fascinated chemists for more than a century. Indeed, one of the earliest discoveries was made by the pioneering chemists Ciamician and Silber that observed the photoreduction of benzoquinone to hydroquinone when dissolved in ethanol and exposed to light.^1^ Since then, many efforts have been devoted to the development of new photochemical transformations involving ketones and to advancing the understanding of the underlying mechanisms.^2^ In particular, diaryl ketones have attracted significant attention from chemists due to their rich excited-state behavior and applications in synthetic photochemistry, materials science, and photobiology.^2^ Upon UV-light irradiation, diaryl ketones typically undergo efficient intersystem crossing from the excited singlet state to a long-lived triplet state (Scheme 1A).^3^ From the triplet state, these compounds can participate in a variety of Norrish-type photochemical reactions, such as hydrogen atom abstraction, fragmentation, or radical coupling reactions.^4^ Benzophenone is probably one of the most deeply studied diaryl ketones, and many works have been devoted to the study of its photochemistry,^5^ either under laser flash photolysis,^6^ ultrafast spectroscopy,^7^ or using calculations,^8^ including the characterization of the triplet excited state or the intermediate ketyl radical^9^ formed after the HAT event.^10^ Moreover, in the field of photocatalysis, many diaryl ketone derivatives are being employed as (co)-catalytic HAT abstractors for the C–H activation of nonacidic positions under photocatalytic conditions for the generation of C-centered radicals.^11^

**Scheme 1:**
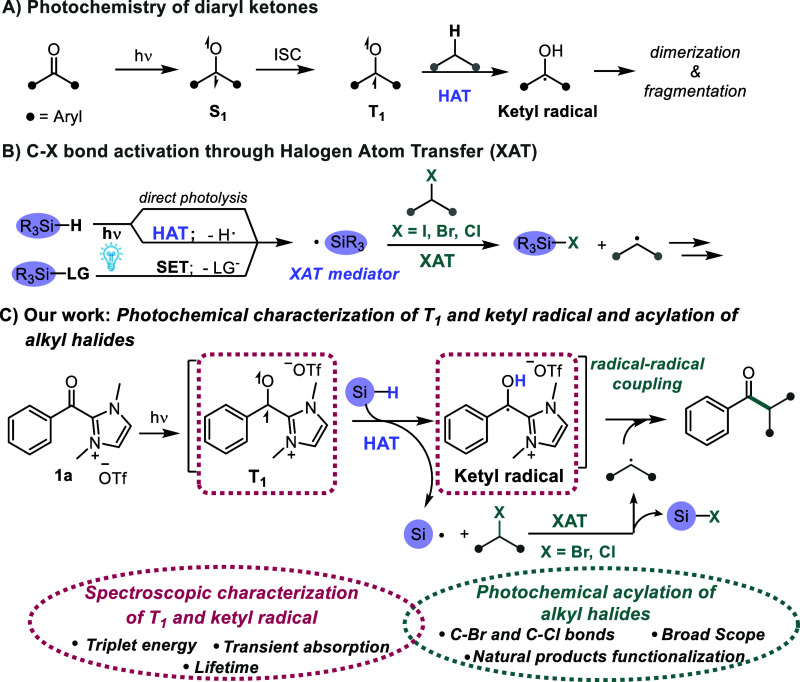
(A) Photochemistry of Diaryl Ketones; (B) C-Centered Radicals from C–X Bonds through XAT; (C) Photochemical Acylation of Alkyl Halides and Characterization of Intermediates

Recently, acyl azolium salts (**1a** in Scheme 1C), another class of diaryl ketones able to act as an HAT abstractor and, at the same time, incorporate the acyl residue in the final molecule, have been described. Acyl azolium salts are key intermediates in the NHC organocatalytic activation of carboxylic acid derivatives,^12^ and their structural similarity to diaryl ketones has made them excellent targets to perform photochemical transformations. Indeed, in 2020, pioneering work by Hopkinson showed the photochemical potential of these acyl azolium salt intermediates in organic synthesis.^13^ Later, Scheidt described their HAT abstractor ability in the acylation of highly stabilized alkyl radicals^14^ that has been also applied to the synthesis of α,β-unsaturated ketones^15^ and the acylation of C-centered radicals derived from the radical addition to styrene derivatives.^16^ Nevertheless, despite the efforts devoted to expand their synthetic utility,^17^ the photochemical investigation of their reactivity and the spectroscopic detection and characterization of reactive intermediates is still very limited.

Another interesting mode to generate alkyl radicals in a very efficient manner is the halogen atom transfer (XAT) strategy.^18^ This activation mode is not dependent on the reduction potential of the starting materials or the electronic environment of the C–X bond (as in the HAT process), opening the door to activation of any position within a single molecule. Silicon radicals have become a very popular alternative because of their high XAT rates and the large variety of methods available to generate the key Si-centered radical (Scheme 1B).^19^ Of the three principal approaches for their preparation—the direct photolysis under UV-light irradiation,^20^ the HAT activation,^21^ or the single electron transfer (SET) strategy (reduction step) to the corresponding silane bearing an appropriate leaving group (LG)^22^—the most popular is the activation through a HAT event. This Si-centered radical acts as a XAT mediator in the activation of alkyl iodides, bromides, or chlorides, to form new carbon-centered radicals that then engage in different radical addition processes.

Our interest in the combination of NHC catalysis with light^17c^ prompted us to study the photochemistry of acyl azolium salts, including the characterization of the key reactive intermediates, affording essential insights for the development of a tandem HAT/XAT acylation of alkyl halides (Scheme 1C). Thus, herein we present a spectroscopic identification of the triplet state of acyl azolium salt **1a** and the reaction paths followed from there. This study includes transient absorption spectroscopy experiments, allowing the detection of the key ketyl radical formed upon HAT. The mechanistic knowledge derived from these spectroscopic experiments has enabled the development of an innovative tandem HAT/XAT acylation method to directly acylate alkyl bromides and the more challenging alkyl chlorides, that are not accessible through other NHC-mediated approaches.^23^ We hypothesized that the excited acyl azolium salt will undergo HAT with a silane, generating the corresponding Si-centered radical and the ketyl radical. Additional support is given by the molecular orbital model of n → π ketone photochemistry described by Zimmerman,^24^ according to which the addition of the positively charged imidazolium ring to one side of the ketone would increase the electrophilicity of the carbonyl oxygen, enhancing the reactivity toward electron-rich Si–H. Next, the Si-centered radical will activate the alkyl halide through a XAT event creating a new C-centered radical, followed by a radical–radical recombination step with the persistent ketyl radical, ultimately affording the desired acylated products.

## Photochemical Characterization

With this concept in mind, we initially investigated the photochemistry of acyl azolium salt **1a**. Upon photoexcitation, diaryl ketones reach the singlet excited state S_1_, which evolves through ISC to the triplet state T_1_.^14^ Measuring the emission spectrum of **1a**, no strong emission is observed at room temperature, whereas a pronounced phosphorescence is observable when cooling the solution to 77 K, with the emission bands lying within the 400–600 nm spectral range. In addition, the excitation spectrum matches the absorption spectrum (Figure 1a). Moreover, the triplet energy of the acyl azolium could be determined to be 68.1 kcal/mol (2.96 eV), which is in good agreement with the theoretical value described earlier (66.12 kcal/mol, 2.87 eV)^15^ and correlates well with the triplet energy of benzophenone (68.3 kcal/mol, 2.96 eV).^25^ Time-correlated single photon counting (TCSPC) experiments disclosed a 6 ms lifetime of the triplet state at 77 K (Figure 1b) that decreased when the temperature is increased (vide infra). This value is comparable to the triplet lifetime reported for benzophenone.^26^

**Figure 1:**
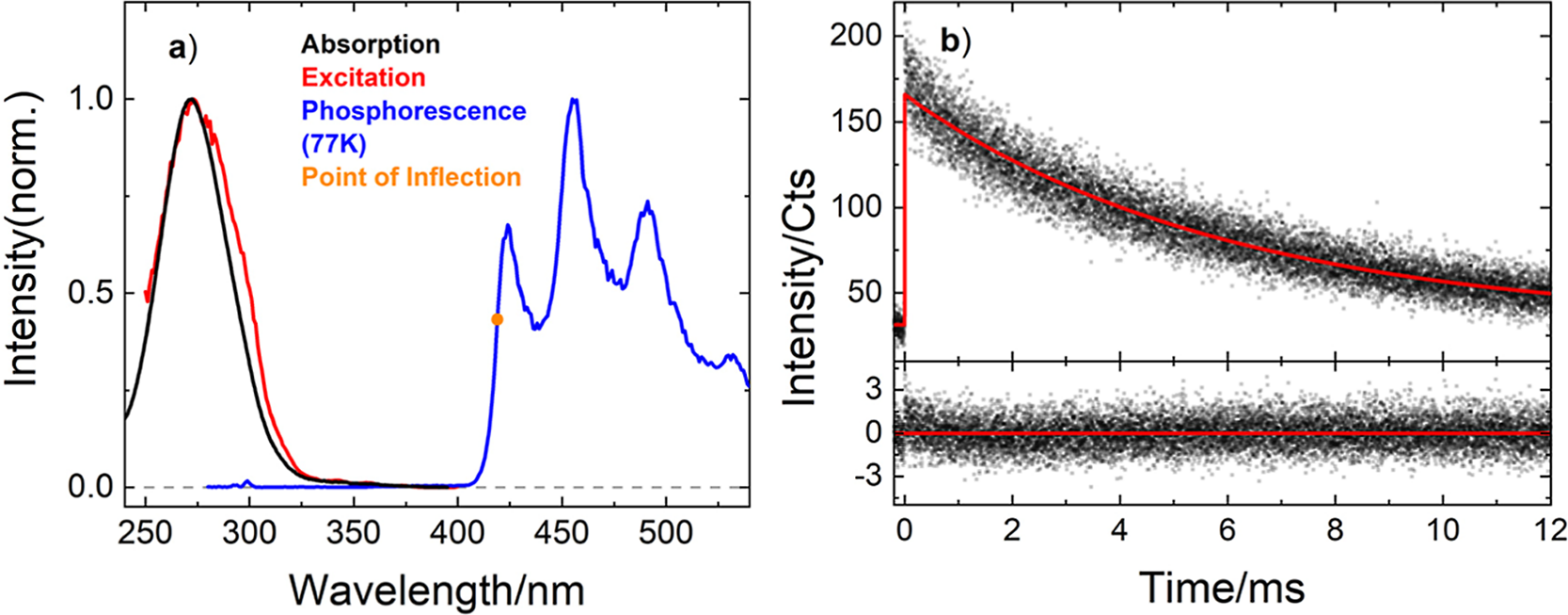
(a) Absorption, excitation, and phosphorescence spectra of **1a** in ACN. The triplet energy is determined from the point of inflection on the short wavelength edge of the emission spectrum. (b) TCSPC data (top, black) of a sample of **1a** in ACN at 77 K. The fit (top, red) corresponds to a phosphorescence lifetime of 6 ms. The Poisson-weighted residues are shown in the bottom.

### Transient Absorption Spectroscopy

In addition to the triplet state, we were interested in the subsequent photochemistry originating from excited **1a**, and in particular the detection of the ketyl radical intermediate generated upon HAT. Thus, we conducted transient absorption experiments on a ns–ms time scale (Figure 2, see Supporting Information for details). Excitation at 266 nm in ACN and DCM, respectively, yielded a short-lived component attributed to the triplet state **1a­(T**_**1**_ **)** as well as longer-lived components which are ascribed to the ketyl radical and possible photoproducts (Scheme 2a).

**Figure 2:**
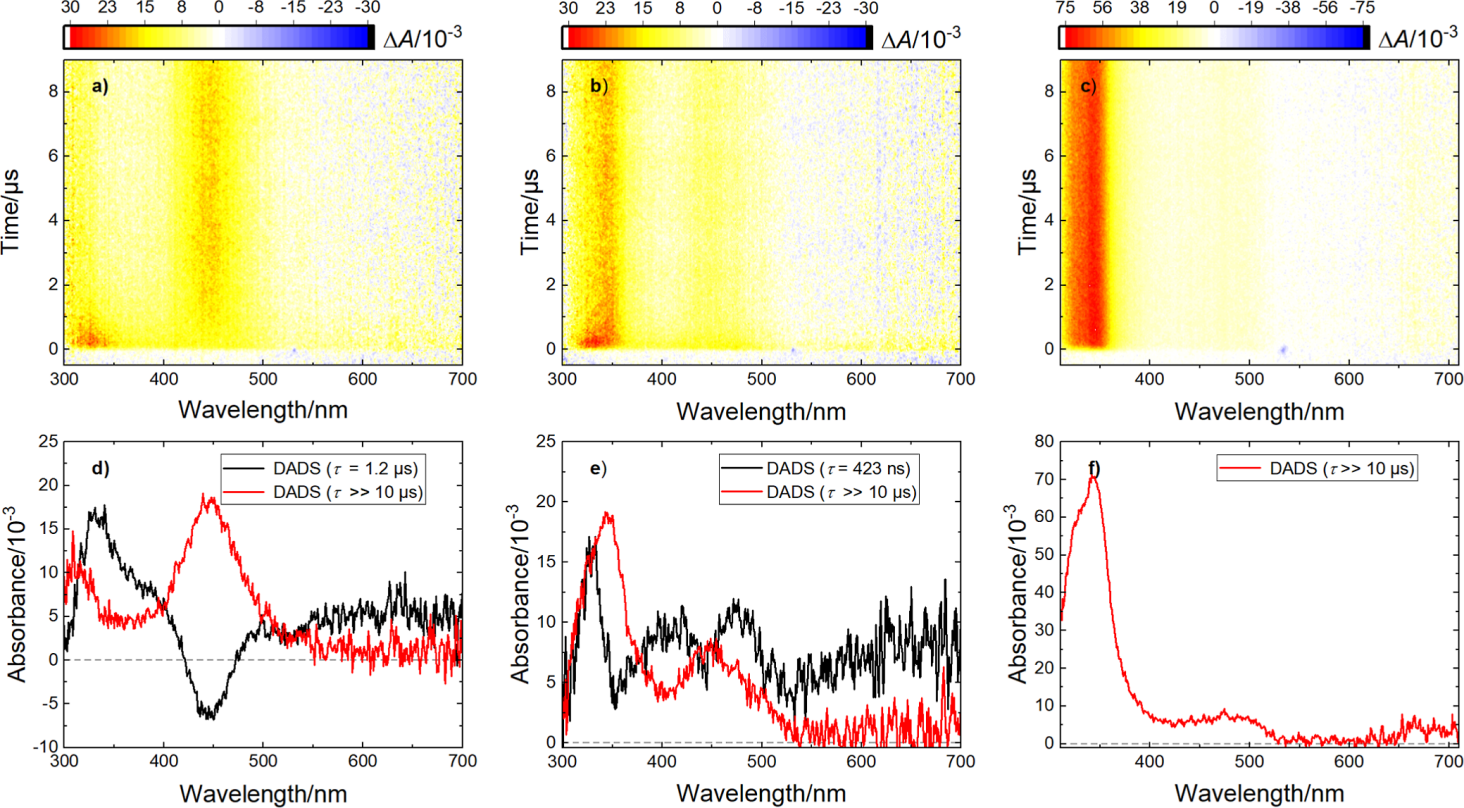
(a) Transient absorption data at room temperature of **1a** in ACN shows a shorter-lived and a longer-lived component, which may be attributed to **1a­(T**_**1**_ **)** and the ketyl radical, respectively. (b) The same experiment in DCM shows similar signals, though with a reduced triplet lifetime. (c) In THF, only a long-lived species is observable. (d) Global lifetime analysis (with decay-associated difference spectra, DADS, displayed here) reveals a triplet lifetime of 1.2 μs in ACN, while a new long-lived signal, attributed to the ketyl radical, emerges from this state. Note that the negative black signal and the positive red signal around 450 nm are characteristic signatures of one species growing in with a rise time corresponding to the decay time of another species. (e) A similar behavior is observed in DCM, though with a shorter triplet lifetime of only 423 ns. (f) Only a long-lived signal, matching the ketyl radical absorption, is observable in THF, indicating quenching of the triplet via HAT from the solvent below the experiment’s temporal resolution.

**Scheme 2:**
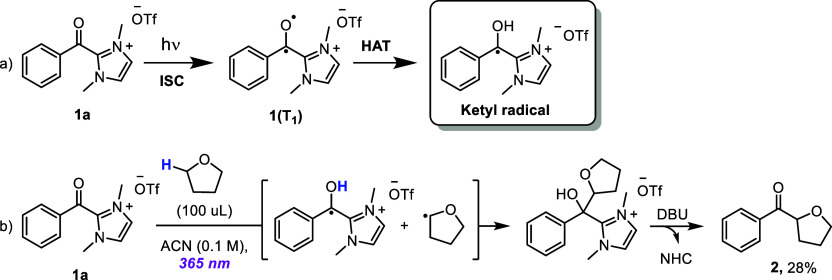
(a) Formation of **1a­(T**_**1**_ **)** and the Ketyl Radical after Photoexcitation of **1a** and HAT Event. (b) Photochemical Synthesis of Phenyl­(tetrahydrofuran-2-yl)­methanone **2**

In THF, on the other hand, **1a­(T**_**1**_ **)** is no longer observable, most likely due to fast HAT being possible from the solvent itself. Hence, only the ketyl radical is present within the available timeframes. This is corroborated by the fact that when irradiating **1a** in the presence of 100 μL of THF, **2** is isolated in 28% yield (Scheme 2b). Both results corroborate a fast HAT between **1a­(T1)** and THF, forming the ketyl radical and the THF radical, that recombine to finally afford **2** (Scheme 2b).

## Results and Discussion

With these insights in hand, we proceeded to study the reaction between **1a** and benzyl bromide **3a** in the presence of TTMSS (tris­(trimethylsilyl)­silane) (Table 1). Ketone **4a** could be isolated in 94% yield using 2 equiv of **1a**, 1 equiv of **3a** and TTMSS, and acetonitrile as a solvent in a 0.01 M concentration, under 370 nm Kessil lamp irradiation for 24 h at room temperature. The use of other irradiation sources such as 365 nm, 385 nm, or 410 nm LED afforded lower yields due to the lower power of the light source in comparison to the Kessil lamp (see Supporting Information). Other solvents such as acetone, dichloromethane, or THF afforded lower yields, while the use of DMSO, DMF, toluene, hexafluorobenzene, or the trifluoromethyl toluene afforded no conversion at all. Changes in the concentration or the stoichiometry rates of the reagents also provided lower yields.

**Table 1:**
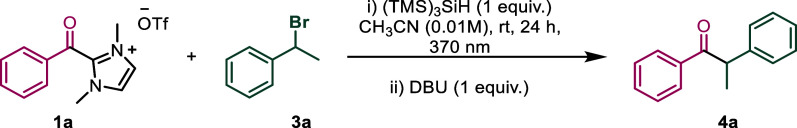
Screening of the Reaction Conditions^a^

entry	deviation from standard conditions^a^	yield (%)^b^
1	none	100 (94)^c^
2	365, 385, or 410 nm LED, instead of 370 nm Kessil lamp	30–53
3	acetone, DCM, DCE, or THF instead of CH_3_CN	15–36
4	toluene, DMSO, DMF, or C_6_F_6_, PhCF_3_ instead of CH_3_CN	0
5	0.1 or 0.2 M instead of 0.01 M	45–54
6	3 equiv of **1a**	64
7	1/2/2 stoichiometry of **1a**/**2a**/TTMSS	48

a Standard conditions: the reaction is carried out with **1a** (0.1 mmol, 2 equiv), **3a** (0.05 mmol, 1 equiv), TTMSS (0.05 mmol, 1 equiv) in 5 mL of CH_3_CN, r.t., 370 nm Kessil lamp, 24 h.

b NMR yield determined with an internal standard.

c Isolated yield.

Next, the scope of the reaction between acyl azolium salts **1** and alkyl halides **3** was investigated (Scheme 3). Initially, a variety of acyl azolium salts **1** were subjected to their reaction with benzyl bromide **3a**. The reaction was compatible with strong electron-withdrawing groups in the *meta* or *para* position, including a nitrile group, affording the final products in good to very good yields (**4b**–**4d**). It is worth noting that the reaction was compatible with halogen atoms in the *ortho* or *para* position of the aromatic ring (**4b**–**4d**), opening the door to further functionalization of the molecules. Unfortunately, with strong electron-donating groups such as the methoxy group (**4h**), the reaction did not work,^27^ although better results were obtained with soft electron-donating substituents (**4i**) or electron-rich heteroarenes such as a furane (**4j**).^28^

**Scheme 3:**
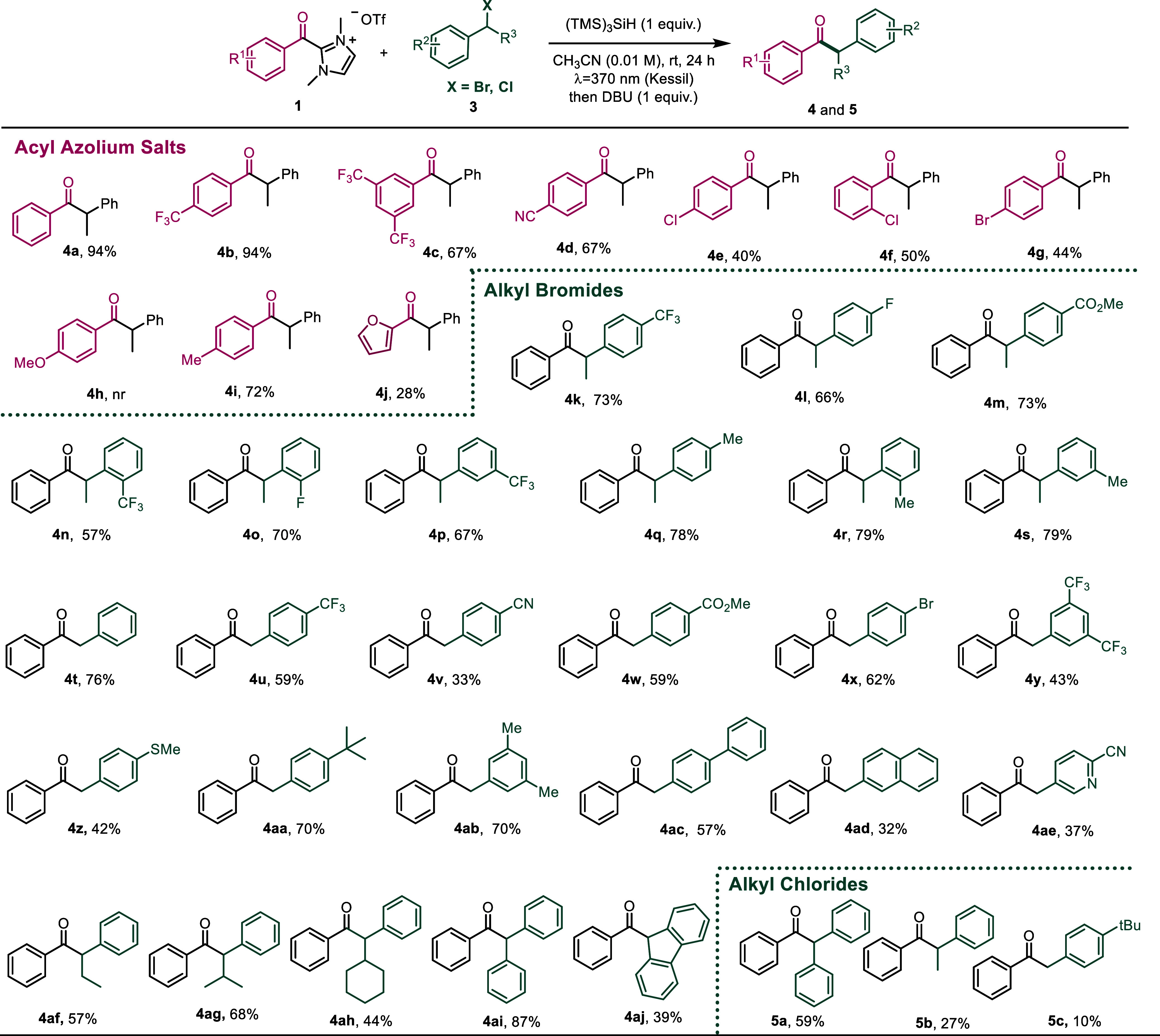
Substrate Scope^a^

a Standard reaction conditions: **1** (0.1 mmol, 2 equiv), **3** (0.05 mmol, 1 equiv), TTMSS (0.05 mmol, 1 equiv) in 5 mL of CH_3_CN, r.t., 370 nm Kessil lamp, 24 h.

Then, a variety of alkyl bromides **3** were subjected to the reaction conditions. The reaction afforded good to very good yields with 1-bromoethylbenzene derivatives bearing either electron-withdrawing or soft electron-donating substituents in the *ortho*, *meta*, or *para* position of the aromatic ring (**4k**–**4s**). Fluorinated substituents are well tolerated, as well as an ester group, susceptible to reaction with the NHC released upon treatment with the base or alkyl ones, obtaining the final products in high yields in all the cases. Next, the primary benzyl bromide was successfully employed, affording **4t** in 76% yield. With primary benzyl bromide derivatives, a similar behavior was observed as with the secondary ones. Thus, the electronic nature of the substituent and its position in the aromatic ring does not have a significant impact in the reactivity (**4u**–**4ae**). It is worth highlighting that the reaction is compatible with bromo substituents in the aromatic ring (**4x**) or with the pyridine moiety (**4ae**), commonly present in the structure of bioactive compounds.^29^ Moreover, **4z** bearing a strong electron-donating thioether substituent was afforded in a good yield, and the presence of more conjugated systems such as a biphenyl or a naphthyl derivative is also tolerated (**4ad** was obtained in a lower yield due to the lower solubility of the starting material under the reaction conditions). Next, other secondary alkyl bromides bearing different alkyl substituents were studied in the reaction. The reaction tolerated an ethyl, an isopropyl, or a cyclohexyl substituent affording ketones **4af**, **4ag**, and **4ah** in good yields. In addition, alkyl bromides bearing a second aryl substituent also underwent the reaction smoothly providing **4ai** and **4aj** in good yields.^27^ A common limitation of NHC-catalyzed acylation methods for alkyl halides is the activation of alkyl chloride derivatives,^24^ which exhibit higher reduction potential or are less reactive in nucleophilic substitution reactions. Given that our reaction is independent of the redox potentials, we overviewed that it would allow to overcome this synthetic limitation. Therefore, different alkyl chlorides were submitted to the reaction conditions, being possible to successfully obtain compound products **5a**–**5c** bearing both alkyl or aryl substituents, proving the potential of this methodology.

Additionally, to prove the robustness and applicability of the method, it was applied to the functionalization of three natural product derivatives. A derivative of the systemic herbicide Dicamba, with hormonal action,^30^ and a derivative of Bexarotene, an antineoplastic agent used to treat certain types of cancer,^31^ both functionalized with the NHC moiety, underwent the reaction affording the products **6** and **7** in 21% and 55% yields, respectively. Additionally, a brominated derivative of the hormone estrone could also be acylated in a good yield (**8**, Scheme 4).

**Scheme 4:**
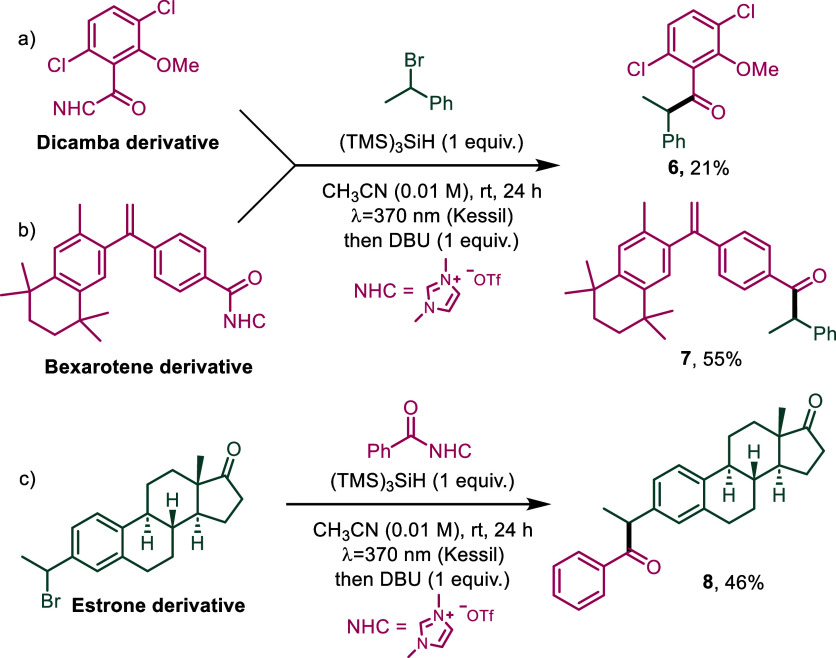
Late-Stage Functionalization of Several Natural Product Derivatives

## Conclusions

In summary, a joint spectroscopic and synthetic study allowed the identification of the photochemical reaction pathway and the associated intermediates that occur after the photoexcitation of acyl azolium salts. We were able to identify the triplet state through its phosphorescence spectrum and determine the triplet energy. Transient absorption spectroscopy allowed the visualization of the subsequent HAT process by unveiling the absorption spectrum of the ketyl radical intermediate, corroborating that the underlying mechanism corresponds to a HAT activation process. With these valuable inferences, we could develop a direct acylation method for alkyl bromides and the more challenging alkyl chlorides through a photochemical HAT/XAT activation process. We have demonstrated the broad scope of the reaction and further substantiated its robustness and versatile applicability through the functionalization of three natural product derivatives.

## Supplementary Material



## Data Availability

The data that supports the findings of this study are available in the Supporting Information of this article. Primary spectroscopic data are openly available in the repository RADAR4Chem at DOI: 10.22000/c8xzzxuu35trrdqj.

## Notes

Additional experimental details, materials, and methods, including experimental procedures and characterization of the products (PDF)
