# Supplementary material for: Photochemical Insights on Acyl Azolium Salts Enable the Design of a Tandem Hydrogen Atom Transfer/Halogen Atom Transfer Acylation of Alkyl Bromides and Chlorides
Source: J Am Chem Soc. 2025 Aug 14;147(34):31324–31. doi: 10.1021/jacs.5c10923 (PMC12395483; doi:10.1021/jacs.5c10923)
Supplement: Supplementary file 1 [file ja5c10923_si_001.pdf]

SUPPORTING INFORMATION

Photochemical insights on acyl azolium salts enable the design of a tandem hydrogen atom transfer/halogen atom transfer acylation of alkyl bromides and chlorides.

Ian MacLean,[a] Daniel Jan Grenda,[b] Elena Echávarri,[a] Stephan Muth,[b] Patrick Nuernberger,[b] Leyre Marzo,*[a,c]

[a] Organic Chemistry Department (Módulo 1) Universidad Autónoma de Madrid, 28049 Madrid (Spain)

[b] Institut für Physikalische und Theoretische Chemie Fakultät für Chemie und Pharmazie Universität Regensburg, 93040 Regensburg (Germany).

[c] Institute for Advanced Research in Chemical Sciences (IAdChem) Universidad Autónoma de Madrid, 28049 Madrid (Spain)

*E-mail:leyre.marzo@uam.es

Index

1. General methods and materials S3

2. Photochemical setup S5

3. Synthesis and characterization of reactants and products S7

3.1. General procedure A: Synthesis of *N*-methyl-C2-acyl imidazoles (S1a-S1l) S7

3.2. General procedure B: Synthesis of dimethylacylimidazolium salts (1a – 1l) S11

3.3. General procedure C: Synthesis of alkyl bromides (3b, 3i, 3v, 3w and 3x) S15

3.4. Synthesis of (8*R*,9*S*,13*S*,14*S*)-3-(1-bromoethyl)-13-methyl-6,7,8,9,11,12,13,14,15,16-decahydro-17*H*-cyclopenta[*a*]phenanthren-17-one (3ab) S17

3.5. Photochemical synthesis of phenyl(tetrahydrofuran-2-yl)methanone (2) S18

3.6. General procedure D: Synthesis of acylated products S19

5. Unsuccessful examples S31

5.1. Alkyl halides S31

5.2. Acyl azolium salts S31

6. Spectroscopic studies S32

6.1. Determination of the triplet energy S32

6.2. Determination of the phosphorescence rate constant S32

6.3. Transient absorption and observation of the hydrogen atom transfer S33

7. NMR spectroscopic data S42

8. References S88

1. General methods and materials

Commercial-grade reagents and solvents were purchased from Acros Organics, Alfa Aesar, Fluorochem, Sigma-Aldrich, BLD Pharm, and TCI Chemicals. All synthesis photochemical were carried out using argon atmosphere and using dry solvents with analytical standard grades.

Analytical TLC was performed using pre-coated aluminum-backed plates (Merck TLC Silicagel 60 F254) and visualized by ultraviolet irradiation. Chromatographic purification of products was accomplished by flash chromatography using silica gel (Merck Geduran® Si 60) unless another stationary phase is specified.

NMR spectra were acquired on a BRUKER AVANCE 300, BRUKER AVANCE-II 300 or BRUKER AVANCE NEO 500 spectrometer operating at 300 or 500 MHz for ^{1}H, 75 or 125 MHz for ^{13}C and 282 or 470 MHz for ^{19}F. Chemical shifts were internally referenced to residual solvent signals ($CDCl_3$: δ 7.26 ppm for ^{1}H NMR and δ 77.0 ppm for ^{13}C-NMR; DMSO-d6: δ 2.50 ppm for ^{1}H NMR and δ 39.5 ppm for ^{13}C-NMR). Data for ^{1}H NMR are reported as follows: chemical shift (δ ppm), multiplicity (*s* = singlet, *d* = doublet, *t* = triplet, *q* = quartet, *m* = multiplet, *br* = broad), coupling constant (Hz) and integration.

High-Resolution Mass Spectra (HRMS) were obtained on an Agilent Technologies 6120 Quadrupole LC/MS coupled with an SFC Agilent Technologies 1260 Infinity Series instrument for the MS (ESI) (Electrospray Ionization). MassWorks software version 4.0.0.0 (Cerno Bioscience) was used for formula identification. MassWorks is an MS calibration software which calibrates isotope profiles to achieve high mass accuracy and enables elemental composition determination on conventional mass spectrometers of unit mass resolution allowing highly accurate comparisons between calibrated and theoretical spectra.

The solvents used for the spectroscopic studies, namely dichloromethane (Fisher Scientific, ≥ 99.8 %), acetonitrile (Roth, UV/IR-Grade, ≥99.9 %) and tetrahydrofuran (Aldrich, anhydrous, 99.9 %, inhibitor-free), were of spectroscopic grade.

Stationary UV/Vis absorption spectra were recorded using either a Shimadzu UV-1800 or an Agilent Cary60 spectrometer. Quartz cuvettes (Starna or Hellma) allowed for a sample thickness of 10 mm. Where degassing was necessary, an in-house modified version of these cuvettes with an attached round flask was utilized. Oxygen exclusion was achieved by subjecting the sample in these modified cuvettes to three cycles of freeze-pump-thaw using a vacuum of the order of magnitude 10^{-3} mbar with subsequent argon flushing (*ca.* 1.2 bar).

For stationary emission and excitation spectra, a Fluorolog 3 from Horiba was utilized. The same spectrometer was employed for time-correlated single photon counting (TCSPC) experiments, by setting the monochromator of the exit slit to the emission maximum and triggering a laser diode (260 nm, PicoQuant, PLS-8-2-989) *via* a digital delay generator (Stanford Research Systems, DG645) for excitation.

Transient absorption measurements were performed with a previously described[1], in-house built setup, which will only be discussed briefly at this point. A stirred sample is excited using the fourth harmonic (266 nm) of a Nd:YAG laser (Surelite II, Continuum, *ca.* 10 mJ, *ca.* 10 ns) and probed with an orthogonal 150 W Xe-flash lamp pulse (Applied Photophysics). The detector setup consists of a spectrograph (200is, Bruker) and a streak camera (C7700, Hamamatsu Photonics), which allows simultaneous spectral and temporal resolution within a single measurement cycle. Using mechanical shutters pump and probe pulse can be blocked separately or simultaneously, allowing for measurements of light intensities with both beam paths open I_{Data}, blocked pump I_{Ref} and blocked pump and probe paths I_{Dark} (and I'_{Dark}). These values are recorded for N measurement cycles and the transient absorption changes ΔA at each wavelength λ and time t are calculated as shown in equation S1.

$$\Delta A(\lambda,t) = -\log\left(\frac{\left(I_{\mathrm{Data}}(\lambda,t) - I_{\mathrm{Dark}}(\lambda,t)\right)}{\left(I_{\mathrm{Ref}}(\lambda,t) - I'_{\mathrm{Dark}}(\lambda,t)\right)}\right) \qquad (S1)$$

Results are matrices of 512x512 elements with each element being the difference absorption at a certain wavelength and time, which can then be modelled using global fit methods (*vide infra*).

Global lifetime analysis

Assuming the sum of the concentrations of all species to be constant and equal to the concentration of molecules initially excited by the pump pulse, the data matrix $\Delta A(\lambda, t)$ can be modeled as the sum of each species associated difference spectrum (SADS) multiplied with the concentration of said species. Hence, fitting the data matrix $\boldsymbol{\Delta A}$ means solving the minimization problem shown in equation S2, with $\mathbf{F}$ being a matrix containing the time-dependent concentration profiles and $\mathbf{S}$ containing the SADS of each species.

$$||\boldsymbol{\Delta A} - \mathbf{FS}||^2 = \mathrm{Min} \qquad (S2)$$

Assuming only first-order reactions, the entries of $\mathbf{F}$ are exclusively sums of exponential functions, convoluted with the instrument response function, which is assumed to be a gaussian. Then, the results of the fit are so-called decay-associated difference spectra (DADS), as well as the rate constants associated with them.

The details of the fitting routine were described previously.[1]

For measurements on a 500 ns timescale, the width of the IRF is known[2] to be about 15 ns and was fixed to this value.

2. Photochemical setup

A custom-made photoreactor setup was used for the optimization of the photocatalytic reactions (Figure S1). The vial is placed inside the fitted well in which irradiation takes place at the desired wavelengths (365, 385 or 420) using 380 mW single LEDs (**Figure S1**). Reaction temperature is kept at 20-25 ºC using a recirculating chiller. Kessil® LED PhotoReaction Lightning PR160L 370 nm and cooling fans (**Figure S3**) were also used for photochemical reactions. **Figures S4** to **S6** illustrate relevant photophysical properties of the lamps.

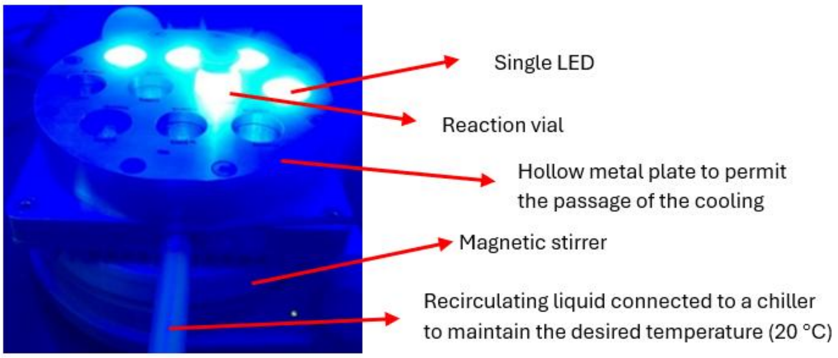

Figure S1: Custom-made photoreactor setup employed during photocatalytic reactions.

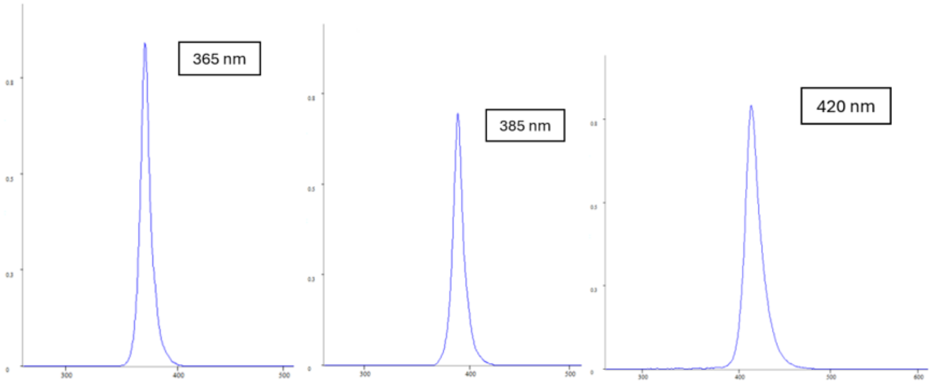

Figure S2: Emission spectrums of the LEDs used in custom-made photoreactor.

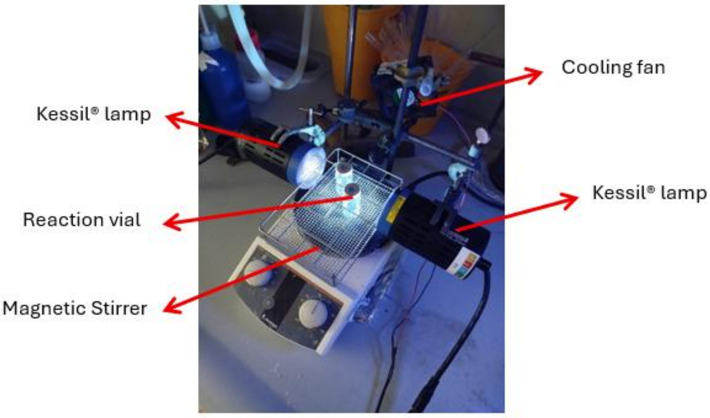

Figure S3: Photochemical setup with Kessil® lamps.

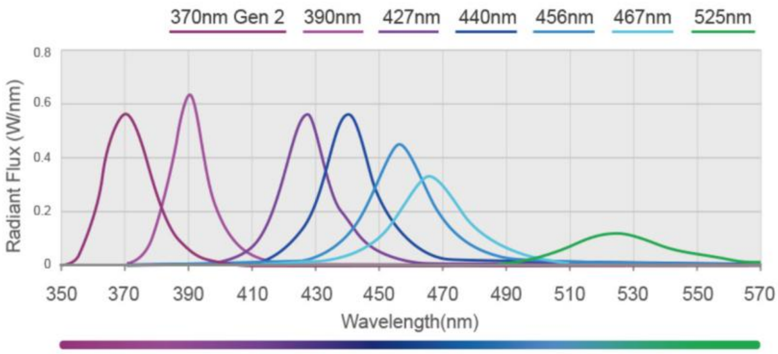

Figure S4: Emission spectrums of Kessil® Lamps

Power Consumption (AC)	370nm Gen 2 (max 44W), 390nm (max 52W), 427nm & 440nm (max 45W), 456nm (max 50W), 467nm (max 44W), 525nm (max 44W)
Input Voltage	100-240 VAC
Operating Temperature	0 - 40°C / 32 - 104°F
Beam Angle	56°
Wavelength Options	370nm, 390nm, 427nm, 440nm, 456nm, 467nm, 525nm
Average Intensity of PR160 series	399mW/cm2 (measured from 1 cm distance)
Dimensions (H x D)	4.49" x 2.48" / 11.4cm x 6.3cm

Figure S5: Technical specifications of the Kessil® Lamps.

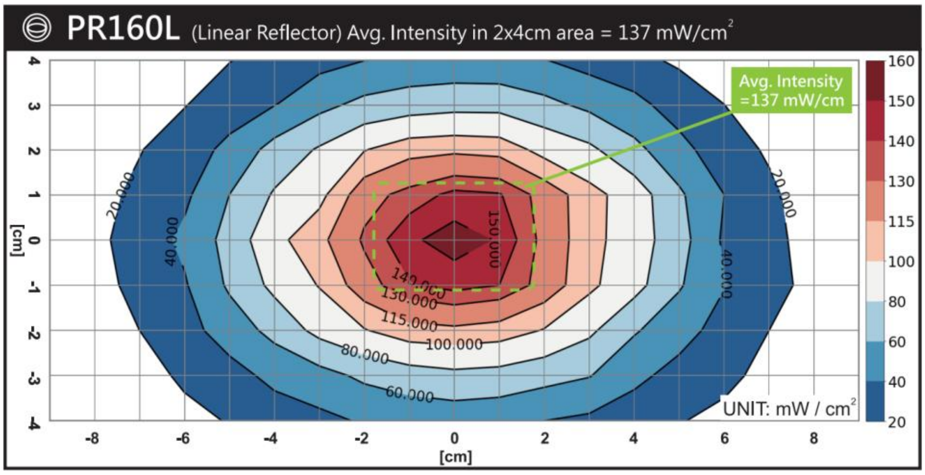

Figure S6: Cross section of illumination area of Kessil® lamp at 6cm distance.

3. Synthesis and characterization of reactants and products

3.1. General procedure A: Synthesis of *N*-methyl-C2-acyl imidazoles (S1a-S1l)

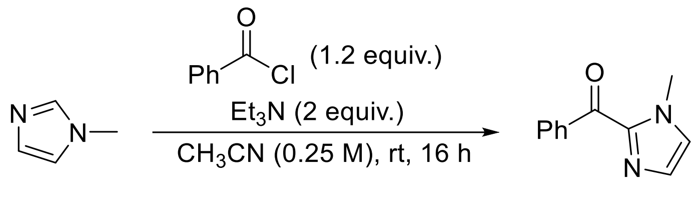

The corresponding acyl chloride (19.2 mmol) was dissolved in 64.0 mL of dry CH_3CN (0.25 M). 2-Methyl imidazole (1.27 mL, 16 mmol) and triethylamine (4.46 mL, 32 mmol) were added dropwise, at that time a precipitate began to form. The mixture was stirred for 16 hours, concentrated and partitioned between water and ethyl acetate. The layers were separated, and the aqueous layer was extracted with ethyl acetate (3 x 20 mL). The organic layers were washed with $NaHCO_{3\,(sat.)}$ and brine, then dried with $MgSO_4$, filtered and concentrated. The residue was purified by silica gel flash chromatography to afford the desired *N*-methyl-C2-acyl imidazoles.

(1-methyl-1H-imidazol-2-yl)(phenyl)methanone (S1a)

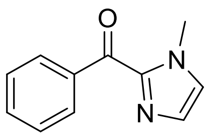

S1a

Following the **general procedure A**, benzoyl chloride (2.23 mL, 19.2 mmol), 2-methylimidazole (1.27 mL, 16.0 mmol) and triethylamine (4.36 mL, 32 mmol) gave product **S1a** (2.91 g, 97%) as a colorless oil after purification (Cyclohexane:EtOAc = 80:20).

^{1}H NMR (300 MHz, $CDCl_3$) δ 8.30 – 8.23 (m, 2H), 7.61 – 7.53 (m, 1H), 7.51 – 7.42 (m, 2H), 7.23 (d, *J* = 1.0 Hz, 1H), 7.10 (d, *J* = 1.0 Hz, 1H), 4.08 (s, 3H).

Spectroscopic data are consistent with those reported in the literature.[3]

(1-methyl-1H-imidazol-2-yl)(4-(trifluoromethyl)phenyl)methanone (S1b)

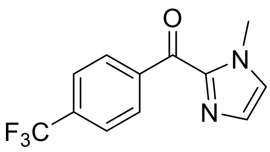

S1b

Following the **general procedure A**, 4-(trifluoromethyl)benzoyl chloride (530 µL, 3.6 mmol), 2-methylimidazole (240 µL, 3.0 mmol) and triethylamine (840 µL, 6.0 mmol) gave product **S1b** (521.3 mg, 68%) as a yellow solid after purification (Cyclohexane:EtOAc = 80:20).

^{1}H NMR (300 MHz, $CDCl_3$) δ 8.40 – 8.34 (m, 2H), 7.79 – 7.69 (m, 2H), 7.25 (d, *J* = 1.0 Hz, 1H), 7.15 (d, *J* = 1.0 Hz, 1H), 4.11 (s, 3H).

Spectroscopic data are consistent with those reported in the literature.[3]

(3,5-bis(trifluoromethyl)phenyl)(1-methyl-1H-imidazol-2-yl)methanone (S1c)

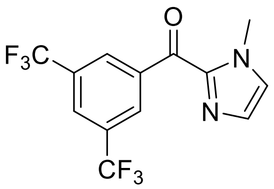
S1c

Following the **general procedure A**, 3,5-bis(trifluoromethyl)benzoyl chloride (650.0 µL, 3.6 mmol), 2-methylimidazole (240 µL, 3.0 mmol) and triethylamine (840 µL, 6.0 mmol) gave product **S1c** (704.8 mg, 73%) as a white solid after purification (Cyclohexane:EtOAc = 80:20).

^{1}H NMR (300 MHz, $CDCl_3$) δ 8.87 (s, 2H), 8.06 (s, 1H), 7.29 (s, 1H), 7.18 (s, 1H), 4.12 (s, 3H).

^{13}C{^{1}H} NMR (125 MHz, $CDCl_3$) δ 180.3, 142.4, 139.0, 131.6 (q, *J* = 33.9 Hz), 131.3 – 131.2 (m), 130.3, 128.0, 125.8 – 125.6 (m), 123.3 (q, *J* = 272.8 Hz), 36.7.

^{19}F{^{1}H} NMR (471 MHz, $CDCl_3$) δ -63.0.

HRMS (ESI$^+$) Calculated for $C_{13}H_9F_6N_2O$ $[M + H]^+$: 323.0541, found: 323.0536.

(4-isocyanophenyl)(1-methyl-1H-imidazol-2-yl)methanone (S1d)

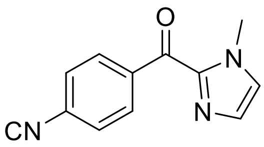
S1d

Following the **general procedure A**, 4-cyanobenzoyl chloride (298.0 mg, 1.8 mmol), 2-methylimidazole (120 µL, 1.5 mmol) and triethylamine (420 µL, 3.0 mmol) gave product **S1d** (165.0 mg, 52%) as a white solid after purification (Cyclohexane:EtOAc = 75:25).

^{1}H NMR (300 MHz, $CDCl_3$) δ 8.39 – 8.33 (m, 2H), 7.77 – 7.72 (m, 2H), 7.24 (s, 1H), 7.15 (s, 1H), 4.08 (s, 3H).

Spectroscopic data are consistent with those reported in the literature.[3]

(4-chlorophenyl)(1-methyl-1H-imidazol-2-yl)methanone (S1e)

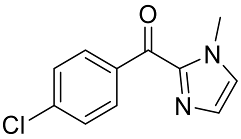
S1e

Following the **general procedure A**, 4-chlorobenzoyl chloride (230 µL, 1.8 mmol), 2-methylimidazole (120 µL, 1.5 mmol) and triethylamine (420 µL, 3.0 mmol) gave product **S1e** (259.2 mg, 78%) as a pale-yellow solid after purification (Cyclohexane:EtOAc = 80:20).

^{1}H NMR (300 MHz, $CDCl_3$) δ 8.32 – 8.23 (m, 2H), 7.48 – 7.41 (m, 2H), 7.22 (d, *J* = 1.0 Hz, 1H), 7.11 (d, *J* = 1.0 Hz, 1H), 4.07 (s, 3H).

Spectroscopic data are consistent with those reported in the literature.[3]

(2-chlorophenyl)(1-methyl-1H-imidazol-2-yl)methanone (S1f)

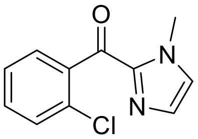
S1f

Following the **general procedure A**, 2-chlorobenzoyl chloride (460.0 µL, 3.6 mmol), 2-methylimidazole (240 µL, 3.0 mmol) and triethylamine (840 µL, 6.0 mmol) gave product **S1f** (432.4 mg, 61%) as a white solid after purification (Cyclohexane:EtOAc = 80:20).

^{1}**H NMR** (300 MHz, $CDCl_3$) δ 7.57 – 7.52 (m, 1H), 7.46 – 7.31 (m, 3H), 7.19 (d, *J* = 0.9 Hz, 1H), 7.12 (d, *J* = 0.9 Hz, 1H), 4.11 (s, 3H).

Spectroscopic data are consistent with those reported in the literature.[3]

(4-bromophenyl)(1-methyl-1H-imidazol-2-yl)methanone (S1g)

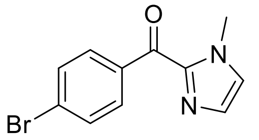

S1g

Following the **general procedure A**, 4-bromobenzoyl chloride (790.1 mg, 3.6 mmol), 2-methylimidazole (240 µL, 3.0 mmol) and triethylamine (840 µL, 6.0 mmol) gave product **S1g** (521.9 mg, 66%) as a white solid after purification (Cyclohexane:EtOAc = 80:20).

^{1}**H NMR** (300 MHz, $CDCl_3$) δ 8.23 – 8.17 (m, 2H), 7.65 – 7.60 (m, 2H), 7.23 (d, *J* = 1.0 Hz, 1H), 7.12 (d, *J* = 1.0 Hz, 1H), 4.08 (s, 3H).

Spectroscopic data are consistent with those reported in the literature.[4]

(4-methoxyphenyl)(1-methyl-1H-imidazol-2-yl)methanone (S1h)

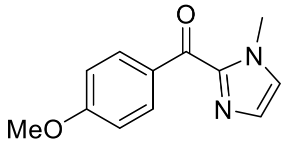

S1h

Following the **general procedure 1**, 4-methoxybenzoyl chloride (614.0 mg, 3.6 mmol), 2-methylimidazole (240 µL, 3.0 mmol) and triethylamine (840 µL, 6.0 mmol) gave product **S1h** (398.6 mg, 61%) as a white solid after purification (Cyclohexane:EtOAc = 80:20).

^{1}**H NMR** (300 MHz, $CDCl_3$) δ 8.38 – 8.31 (m, 2H), 7.18 (d, *J* = 1.0 Hz, 1H), 7.05 (d, *J* = 1.0 Hz, 1H), 6.98 – 6.91 (m, 2H), 4.03 (s, 3H), 3.85 (s, 3H).

Spectroscopic data are consistent with those reported in the literature.[3]

(1-methyl-1H-imidazol-2-yl)(p-tolyl)methanone (S1i)

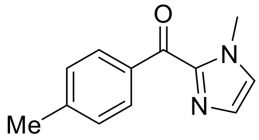

S1i

Following the **general procedure A**, 4-methylbenzoyl chloride (480.0 µL, 3.6 mmol), 2-methylimidazole (240 µL, 3.0 mmol) and triethylamine (840 µL, 6.0 mmol) gave product **S1i** (462.8 mg, 70%) as a yellow oil after purification (Cyclohexane:EtOAc = 80:20).

^{1}**H NMR** (300 MHz, $CDCl_3$) δ 8.22 – 8.17 (m, 2H), 7.31 – 7.26 (m, 2H), 7.22 (s, 1H), 7.09 (s, 1H), 4.07 (s, 3H), 2.42 (s, 3H).

Spectroscopic data are consistent with those reported in the literature.[3]

Furan-2-yl(1-methyl-1H-imidazol-2-yl)methanone (S1j)

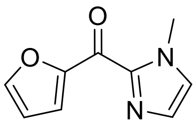

S1j

Following the **general procedure A**, 2-furoyl chloride (355.0 µL, 3.6 mmol), 2-methylimidazole (240 µL, 3.0 mmol) and triethylamine (840 µL, 6.0 mmol) gave product **S1j** (504.9 mg, 80%) as a white solid after purification (Cyclohexane:EtOAc = 75:25).

^{1}H NMR (300 MHz, $CDCl_3$) δ 7.79 (dd, *J* = 3.6, 0.8 Hz, 1H), 7.46 (dd, *J* = 1.8, 0.8 Hz, 1H), 6.88 (d, *J* = 1.0 Hz, 1H), 6.85 (s, 1H), 6.31 (dd, *J* = 3.6, 1.8 Hz, 1H), 3.76 (s, 3H).

Spectroscopic data are consistent with those reported in the literature.[5]

(3,6-dichloro-2-methoxyphenyl)(1-methyl-1H-imidazol-2-yl)methanone (S1k)

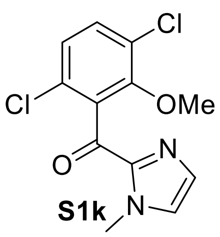

S1k

Following the **general procedure A**, 3,6-dichloro-2-methoxybenzoyl chloride (718.5 mg, 3.0 mmol), 2-methylimidazole (200 µL, 2.5 mmol) and triethylamine (697 µL, 5.0 mmol) gave product **S1k** (560.4 mg, 66%) as a white solid after purification (Cyclohexane:EtOAc = 80:20).

^{1}H NMR (300 MHz, $CDCl_3$) δ 7.32 (d, *J* = 8.6 Hz, 1H), 7.13 (s, 1H), 7.09 (s, 1H), 7.08 (d, *J* = 8.6 Hz, 1H), 4.07 (s, 3H), 3.75 (s, 3H).

^{13}C{^{1}H} NMR (75 MHz, $CDCl_3$) δ 182.7, 153.7, 142.8, 135.1, 131.5, 130.7, 129.3, 127.8, 126.4, 125.8, 62.0, 35.9.

HRMS (ESI$^+$) Calculated for $C_{12}H_{11}Cl_2N_2O_2$ $[M + H]^+$: 285.0119, found: 285.0116

(1-methyl-1H-imidazol-2-yl)(4-(1-(3,5,5,8,8-pentamethyl-5,6,7,8-tetrahydronaphthalen-2-yl)vinyl)phenyl)methanone (S1l)

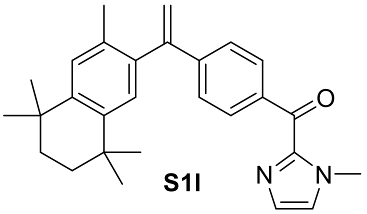

S1l

Following the general procedure 1, 4-(1-(3,5,5,8,8-pentamethyl-5,6,7,8-tetrahydronaphthalen-2-yl)vinyl)benzoyl chloride (121.1 mg, 0.33 mmol), 2-methylimidazole (26.3 µL, 0.33 mmol) and triethylamine (92.9 µL, 0.66 mmol) gave product **S1l** (108.3 mg, 80%) as a white solid after purification (Cyclohexane:EtOAc = 80:20).

^{1}H NMR (300 MHz, $CDCl_3$) δ 8.26 – 8.20 (m, 2H), 7.43 – 7.36 (m, 2H), 7.22 (s, 1H), 7.14 (s, 1H), 7.09 (s, 1H), 7.08 (s, 1H), 5.83 (d, *J* = 1.4 Hz, 1H), 5.33 (d, *J* = 1.4 Hz, 1H), 4.07 (s, 3H), 1.98 (s, 3H), 1.71 (s, 4H), 1.31 (s, 6H), 1.28 (s, 6H).

^{13}C{^{1}H} NMR (75 MHz, $CDCl_3$) δ 183.8, 149.4, 145.4, 144.4, 143.4, 142.4, 138.3, 136.3, 132.9, 131.1, 129.4, 128.1, 128.1, 126.8, 126.4, 116.8, 36.5, 35.4, 34.1, 34.0, 32.1, 32.0, 20.1.

HRMS (ESI$^+$) Calculated for $C_{28}H_{33}N_2O$ $[M + H]^+$: 413.2515, found: 413.2514.

3.2. General procedure B: Synthesis of dimethylacylimidazolium salts (1a – 1l)

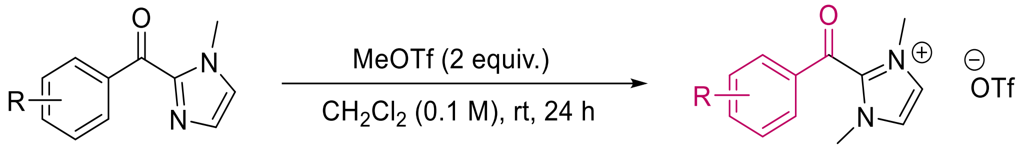

S1 1

To an oven dried round bottom flask equipped with a magnetic stir bar was added the corresponding acyl imidazole (16 mmol), followed by CH_2Cl_2 dry (160 mL, 0.1 M). Methyl trifluoromethanesulfonate (3.62 mL, 32 mmol) was added dropwise and the mixture is stirred 24 h. Then, Et_2O is added dropwise until the solid stopped precipitating and the suspension was filtered and washed with additional Et_2O to provide the final dimethylacylimidazolium salt.

2-benzoyl-1,3-dimethyl-1H-imidazol-3-ium trifluoromethanesulfonate (1a)

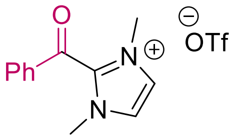

1a

Following the **general procedure B**, (1-methyl-1H-imidazol-2-yl)(phenyl)methanone **S1a** (2.979 g, 16 mmol) gave product **1a** (1.772 g, 59%) as a fluffy white solid.

^{1}H NMR (300 MHz, $CDCl_3$) δ 8.04 – 7.98 (m, 2H), 7.85 – 7.79 (m, 1H), 7.79 (s, 2H), 7.69 – 7.62 (m, 2H), 3.88 (s, 6H).

Spectroscopic data are consistent with those reported in the literature.[3]

1,3-dimethyl-2-(4-(trifluoromethyl)benzoyl)-1H-imidazol-3-ium trifluoromethanesulfonate (1b)

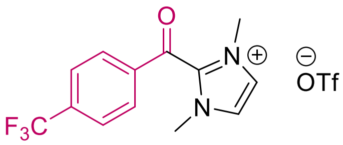

1b

Following the **general procedure B**, (1-methyl-1H-imidazol-2-yl)(4-(trifluoromethyl)phenyl)methanone **S1b** (521.3 mg, 2.05 mmol) gave product **1b** (592.2 mg, 69%) as a fluffy white solid.

^{1}H NMR (300 MHz, DMSO-d6) δ 8.16 – 8.11 (m, 2H), 8.06 – 8.01 (m, 4H), 3.81 (s, 6H).

Spectroscopic data are consistent with those reported in the literature.[3]

2-(3,5-bis(trifluoromethyl)benzoyl)-1,3-dimethyl-1H-imidazol-3-ium trifluoromethanesulfonate (1c)

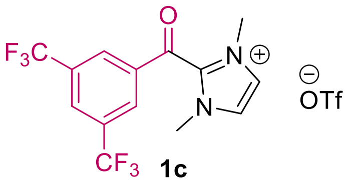

1c

Following the **general procedure B**, (3,5-bis(trifluoromethyl)phenyl)(1-methyl-1H-imidazol-2-yl)methanone **S1c** (704.8 mg, 2.19 mmol) gave product **1c** (647.6 mg, 61%) as a white solid.

^{1}H NMR (500 MHz, DMSO-d6) δ 8.59 (s, 2H), 8.54 (s, 1H), 8.06 (s, 2H), 3.84 (s, 6H).

^{13}C{^{1}H} NMR (125 MHz, DMSO-d6) δ 178.3, 137.5, 137.2, 131.3 (q, *J* = 33.9 Hz), 130.8 – 130.7 (m), 128.1 – 127.9 (m), 126.5, 122.8 (q, *J* = 273.2 Hz), 120.7 (q, *J* = 322.1 Hz), 38.0.

^{19}F{^{1}H} NMR (471 MHz, DMSO) δ -61.6, -78.0.

HRMS (ESI[+]) Calculated for $C_{13}H_8F_6N_2O$ $[M - C_2H_3F_3O_3S + H]^+$: 323.0614, found: 323.0608.

2-(4-cyanobenzoyl)-1,3-dimethyl-1H-imidazol-3-ium trifluoromethanesulfonate (1d)

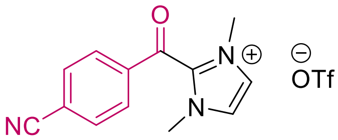

1d

Following the **general procedure B**, (4-isocyanophenyl)(1-methyl-1H-imidazol-2-yl)methanone **S1d** (157.0 mg, 0.74 mmol) gave product **1d** (173.2 mg, 62%) as a white solid.

^{1}H NMR (300 MHz, DMSO-d6) δ 8.17 – 8.13 (m, 2H), 8.11 – 8.06 (m, 2H), 8.02 (s, 2H), 3.79 (s, 6H).

Spectroscopic data are consistent with those reported in the literature.[3]

2-(4-chlorobenzoyl)-1,3-dimethyl-1H-imidazol-3-ium trifluoromethanesulfonate (1e)

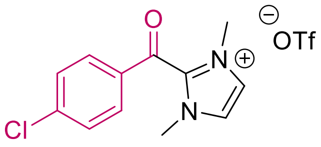

1e

Following the **general procedure B**, (4-chlorophenyl)(1-methyl-1H-imidazol-2-yl)methanone **S1e** (249.2 mg, 1.17 mmol) gave product **1e** (315.4 mg, 70%) as a white solid.

^{1}H NMR (300 MHz, DMSO-d6) δ 7.99 (s, 2H), 7.98 – 7.93 (m, 2H), 7.79 – 7.73 (m, 2H), 3.80 (s, 6H).

Spectroscopic data are consistent with those reported in the literature.[3]

2-(2-chlorobenzoyl)-1,3-dimethyl-1H-imidazol-3-ium trifluoromethanesulfonate (1f)

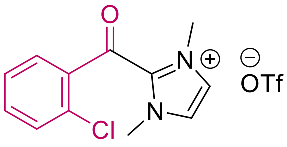

1f

Following the **general procedure B**, (2-chlorophenyl)(1-methyl-1H-imidazol-2-yl)methanone **S1f** (406.3 mg, 1.84 mmol) gave product **1f** (655.4 mg, 93%) as a white solid.

^{1}H NMR (500 MHz, $CDCl_3$) δ 7.90 (dd, *J* = 7.7, 1.7 Hz, 1H), 7.81 (s, 2H), 7.68 (ddd, *J* = 8.0, 7.5, 1.7 Hz, 1H), 7.59 (td, *J* = 7.5, 1.1 Hz, 1H), 7.55 (dd, *J* = 8.0, 1.1 Hz, 1H), 3.88 (s, 6H).

$^{13}C\{^1H\}$ NMR (125 MHz, $CDCl_3$) δ 179.1, 138.3, 135.9, 134.7, 132.5, 132.0, 131.2, 129.1, 126.5, 120.8 (q, *J* = 319.9 Hz), 37.9.

$^{19}F\{^1H\}$ NMR (282 MHz, $CDCl_3$) δ -78.4.

HRMS (ESI$^+$) Calculated for $C_{11}H_{10}ClN_2O$ [M - $C_2H_3F_3O_3S$ + H]+ : 221.0476, found: 221.0477

2-(4-bromobenzoyl)-1,3-dimethyl-1H-imidazol-3-ium trifluoromethanesulfonate (1g)

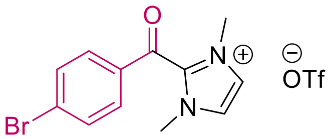

1g

Following the **general procedure B**, (4-bromophenyl)(1-methyl-1H-imidazol-2-yl)methanone **S1g** (521.9 mg, 1.97 mmol) gave product **1g** (644.3 mg, 76%) as a white solid.

^1H NMR (300 MHz, DMSO) δ 7.96 (s, 2H), 7.90 – 7.83 (m, 4H), 3.79 (s, 6H).

Spectroscopic data are consistent with those reported in the literature.[4]

2-(4-methoxybenzoyl)-1,3-dimethyl-1H-imidazol-3-ium trifluoromethanesulfonate (1h)

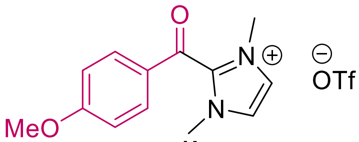

1h

Following the **general procedure B**, (4-methoxyphenyl)(1-methyl-1H-imidazol-2-yl)methanone **S1h** (398.6 mg, 1.84 mmol) gave product **1h** (486.1 mg, 69%) as a fluffy white solid.

^1H NMR (300 MHz, $CDCl_3$) δ 8.02 – 7.96 (m, 2H), 7.71 (s, 2H), 7.14 – 7.07 (m, 2H), 3.94 (s, 3H), 3.87 (s, 6H).

Spectroscopic data are consistent with those reported in the literature.[3]

1,3-dimethyl-2-(4-methylbenzoyl)-1H-imidazol-3-ium trifluoromethanesulfonate (1i)

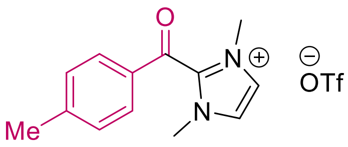

1i

Following the **general procedure B**, (1-methyl-1H-imidazol-2-yl)(p-tolyl)methanone **S1i** (420.6 mg, 2.10 mmol) gave product **1i** (406.9 mg, 53%) as a white solid.

^1H NMR (300 MHz, $CDCl_3$) δ 7.96 – 7.87 (m, 2H), 7.73 (s, 2H), 7.48 – 7.44 (m, 2H), 3.88 (s, 6H), 2.50 (s, 3H).

Spectroscopic data are consistent with those reported in the literature.[3]

2-(furan-2-carbonyl)-1,3-dimethyl-1H-imidazol-3-ium trifluoromethanesulfonate (1j)

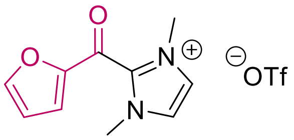

1j

Following the **general procedure B**, Furan-2-yl(1-methyl-1H-imidazol-2-yl)methanone **S1j** (504.9 mg, 2.86 mmol) gave product **1j** (548.8 mg, 68%) as a pale yellow solid

^{1}H NMR (300 MHz, $CDCl_3$) δ 7.95 (dd, *J* = 1.7, 0.7 Hz, 1H), 7.89 (d, *J* = 3.9 Hz, 1H), 7.76 (s, 2H), 6.83 (dd, *J* = 3.9, 1.7 Hz, 1H), 3.96 (s, 6H).

Spectroscopic data are consistent with those reported in the literature.[6]

2-(3,6-dichloro-2-methoxybenzoyl)-1,3-dimethyl-1H-imidazol-3-ium trifluoromethanesulfonate (1k)

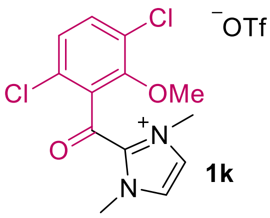

1k

Following the **general procedure B**, (3,6-dichloro-2-methoxyphenyl)(1-methyl-1H-imidazol-2-yl)methanone **S1k** (560.4 mg, 1.96 mmol) gave product **1k** (532.2 mg, 61%) as a white solid.

^{1}H NMR (300 MHz, $CDCl_3$) δ 8.03 (s, 2H), 7.61 (d, *J* = 8.7 Hz, 1H), 7.28 (d, *J* = 8.7 Hz, 1H), 3.95 (s, 6H), 3.91 (s, 3H).

^{13}C{^{1}H} NMR (75 MHz, $CDCl_3$) δ 176.9, 155.0, 137.0, 135.7, 131.3, 129.8, 128.0, 127.4, 127.1, 120.7 (q, *J* = 320.2 Hz), 63.3, 38.1.

^{19}F{^{1}H} NMR (282 MHz, $CDCl_3$) δ -78.5.

HRMS (ESI$^+$) Calculated for $C_{13}H_{13}Cl_2N_2O_2$ $[M - CF_3O_3S]^+$: 299,0342, found: 299.0342

1,3-dimethyl-2-(4-(1-(3,5,5,8,8-pentamethyl-5,6,7,8-tetrahydronaphthalen-2-yl)vinyl)benzoyl)-1H-imidazol-3-ium trifluoromethanesulfonate (1l)

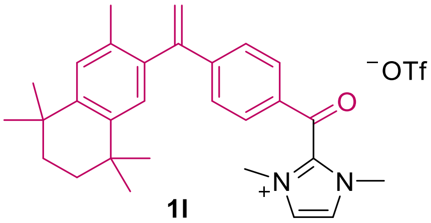

1l

Following the **general procedure B**, (1-methyl-1H-imidazol-2-yl)(4-(1-(3,5,5,8,8-pentamethyl-5,6,7,8-tetrahydronaphthalen-2-yl)vinyl)phenyl)methanone **S1l** (156.0 mg, 0.38 mmol) gave product **1l** (102.2 mg, 47%) as a white solid.

^{1}H NMR (300 MHz, $CDCl_3$) δ 7.98 (d, *J* = 8.4 Hz, 2H), 7.65 (s, 2H), 7.55 (d, *J* = 8.4 Hz, 2H), 7.10 (s, 2H), 5.97 (s, 1H), 5.49 (s, 1H), 3.89 (s, 6H), 1.95 (s, 3H), 1.70 (s, 4H), 1.31 (s, 6H), 1.27 (s, 6H).

^{13}C{^{1}H} NMR (75 MHz, $CDCl_3$) δ 179.8, 149.8, 148.5, 145.0, 142.8, 139.6, 137.1, 133.1, 132.6, 130.8, 128.4, 128.2, 128.1, 125.7, 120.8 (q, *J* = 320.3 Hz), 119.6, 37.3, 35.3, 35.2, 34.1, 34.0, 32.0, 32.0, 20.0.

^{19}F{^{1}H} NMR (282 MHz, $CDCl_3$) δ -78.4.

HRMS (ESI$^+$) Calculated for $C_{29}H_{35}N_2O$ $[M - CF_3O_3S]^+$: 427.2744, found: 427.2732

3.3. General procedure C: Synthesis of alkyl bromides (3b, 3i, 3v, 3w and 3x)

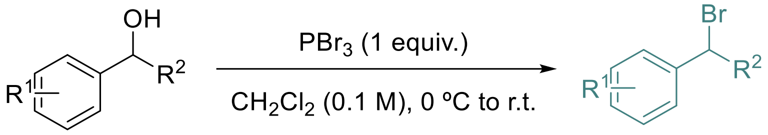

3

To a solution of the corresponding alcohol (1.5 mmol) in CH_2Cl_2 (15.0 mL, 0.1 M) at 0 ºC, PBr_3 (142 µL, 1 equiv.) was slowly added. The mixture was then allowed to warm to room temperature and stirred for 0.5 hours. After confirming the reaction's completion by TLC, the mixture was quenched with ice water, and the CH_2Cl_2 was removed under vacuum. The residue was extracted with AcOEt (3 x 30 mL), and the organic layer was dried over $MgSO_4$ and concentrated. The resulting alkyl bromides were used in the subsequent reaction step without further purification, unless otherwise specified.

1-(1-bromoethyl)-4-(trifluoromethyl)benzene (3b)

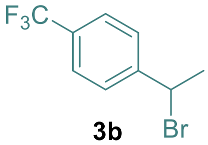

3b

Following the **general procedure C**, 1-(4-(trifluoromethyl)phenyl)ethan-1-ol (231 µL, 1.5 mmol) gave product **3b** (151.4 mg, 40%) as a colorless oil after purification on silica gel (98:2 CyHex/EtOAc).

^{1}H NMR (300 MHz, $CDCl_3$) δ 7.63 – 7.59 (m, 2H), 7.57 – 7.51 (m, 2H), 5.20 (q, *J* = 7.0 Hz, 1H), 2.05 (d, *J* = 7.0 Hz, 3H).

Spectroscopic data are consistent with those reported in the literature.[7]

1-(1-bromoethyl)-2-methylbenzene (3i)

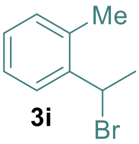

3i

Following the **general procedure C**, 1-(*o*-tolyl)ethan-1-ol (205 µL, 1.5 mmol) gave product **3i** (225.0 mg, 75%) as a colorless oil.

^{1}H NMR (300 MHz, $CDCl_3$) δ 7.72 (dd, *J* = 7.3, 1.8 Hz, 1H), 7.44 – 7.28 (m, 3H), 5.59 (q, *J* = 6.9 Hz, 1H), 2.57 (s, 3H), 2.23 (d, *J* = 6.9 Hz, 3H).

Spectroscopic data are consistent with those reported in the literature.[8]

1-(1-bromoethyl)-3-methylbenzene (3j)

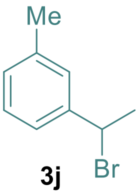

3j

Following the **general procedure C**, 1-(*m*-tolyl)ethan-1-ol (205 µL, 1.5 mmol) gave product **3j** (290.7 mg, 97%) as a colorless oil.

^{1}H NMR (300 MHz, $CDCl_3$) δ 7.37 – 7.29 (m, 3H), 7.22 – 7.14 (m, 1H), 5.27 (q, *J* = 6.9 Hz, 1H), 2.45 (s, 3H), 2.13 (d, *J* = 6.9 Hz, 3H).

Spectroscopic data are consistent with those reported in the literature.[8]

(1-bromopropyl)benzene (3v)

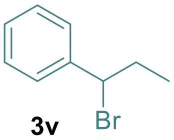

3v

Following the **general procedure C,** 1-phenylpropan-1-ol (206 µL, 1.5 mmol) gave product **3v** (145.2 mg, 49%) as a colorless oil after purification on silica gel (98:2 CyHex/EtOAc).

^{1}H NMR (300 MHz, $CDCl_3$) δ 7.44 – 7.28 (m, 5H), 4.90 (dd, *J* = 8.1, 6.8 Hz, 1H), 2.40 – 2.11 (m, 2H), 1.02 (t, *J* = 7.3 Hz, 3H).

Spectroscopic data are consistent with those reported in the literature.[9]

(1-bromo-2-methylpropyl)benzene (3w)

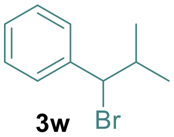

3w

Following the **general procedure C**, 2-methyl-1-phenylpropan-1-ol (234 µL, 1.5 mmol) gave product **3w** (319.0 mg, Cuant.) as a colorless oil.

^{1}H NMR (300 MHz, $CDCl_3$) δ 7.49 – 7.30 (m, 5H), 4.82 (d, *J* = 8.4 Hz, 1H), 2.42 (dhept, *J* = 8.4, 6.6 Hz, 1H), 1.29 (d, *J* = 6.6 Hz, 3H), 0.96 (d, *J* = 6.6 Hz, 3H).

Spectroscopic data are consistent with those reported in the literature.[9]

(Bromo(cyclohexyl)methyl)benzene (3x)

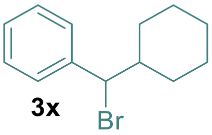

3x

Following the **general procedure C**, cyclohexyl(phenyl)methanol (285.42 mg, 1.5 mmol) gave product **3x** (349.2 mg, 92%) as a colorless oil.

^{1}H NMR (300 MHz, $CDCl_3$) δ 7.26 – 7.08 (m, 5H), 4.60 (d, *J* = 9.1 Hz, 1H), 2.21 (dt, *J* = 13.9, 2.6 Hz, 1H), 1.94 – 1.79 (m, 1H), 1.74 – 1.63 (m, 1H), 1.57 – 1.44 (m, 2H), 1.42 – 1.32 (m, 1H), 1.26 – 1.13 (m, 1H), 1.08 – 0.87 (m, 3H), 0.81 – 0.66 (m, 1H).

Spectroscopic data are consistent with those reported in the literature.[9]

3.4. Synthesis of (8*R*,9*S*,13*S*,14*S*)-3-(1-bromoethyl)-13-methyl-6,7,8,9,11,12,13,14,15,16-decahydro-17*H*-cyclopenta[*a*]phenanthren-17-one (3ab)

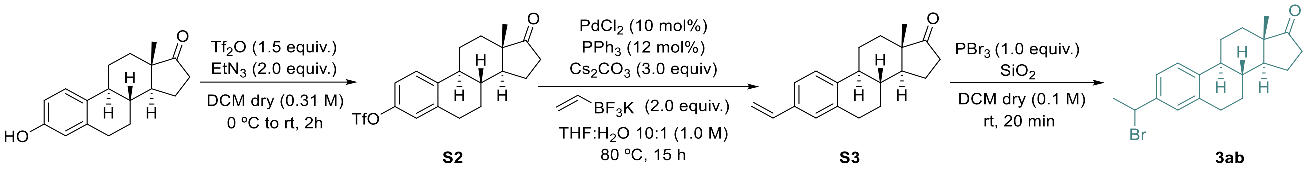

To a solution of the estrone (675.9 mg, 2.5 mmol, 1.0 equiv.) in dry DCM (0.31 M), triethylamine (5.0 mmol, 2.0 equiv.) was added. Then, the reaction mixture was cooled to 0 ºC and trifluoromethanesulfonic anhydride (3.7 mmol, 1.5 equiv.) was added dropwise. After it was warmed to room temperature and stirred for 2 hours, the reaction was quenched with water (20 mL) and extracted with DCM (3 x 40 mL). The combined organic layers were dried over $MgSO_4$, filtered and concentrated under reduced pressure. The crude was purified through flash column chromatography (Cyclohexane:EtOAC, 80:20) to afford the product **S2** as a colorless oil in 76% yield.

^{1}H NMR (300 MHz, $CDCl_3$**)** δ 7.33 (d, *J* = 8.7 Hz, 1H), 7.07 – 6.95 (m, 2H), 2.93 (dd, *J* = 8.7, 4.5 Hz, 2H), 2.62 – 1.81 (m, 7H), 1.77 – 1.36 (m, 6H), 0.91 (s, 3H).

Spectroscopic data are consistent with those reported in the literature.[10]

A sealed vial was charged with the (8*R*,9*S*,13*S*,14*S*)-13-methyl-17-oxo-7,8,9,11,12,13,14,15,16,17-decahydro-6*H*-cyclopenta[*a*]phenanthren-3-yl trifluoromethanesulfonate (**S2,** 603.6 mg, 1.5 mmol, 1.0 equiv.), potassium vinylfluoroborate (3 mmol, 2.0 equiv.), palladium (**II**) chloride (0.15 mmol, 0.1 equiv.), triphenyl phosphine (0.18 mmol, 0.12 equiv.) and cesium carbonate (4.5 mmol, 3 equiv.). Then, dry THF and distilled water (10:1, 1 M) were added, and the reaction was stirred at 80 ºC for 15 h. The mixture was filtered, extracted with water (15 mL) and DCM (3 x 30 mL). The combined layers were dried over $MgSO_4$, filtered and concentrated under reduced pressure. The crude was purified through flash column chromatography (Cyclohexane:EtOAC, 90:10) to afford the product **S3** as a white solid in 27% yield.

^{1}H NMR (300 MHz, $CDCl_3$) δ 7.31 – 7.17 (m, 2H), 7.15 (s, 1H), 6.67 (dd, *J* = 17.6, 10.9 Hz, 1H), 5.71 (dd, *J* = 17.6, 1.1 Hz, 1H), 5.20 (dd, *J* = 10.9, 1.1 Hz, 1H), 2.92 (dd, *J* = 8.9, 4.3 Hz, 2H), 2.59 – 2.39 (m, 2H), 2.39 – 2.24 (m, 1H), 2.24 – 1.91 (m, 4H), 1.75 – 1.35 (m, 6H), 0.92 (s, 3H).

Spectroscopic data are consistent with those reported in the literature.[10]

To a solution of the (8*R*,9*S*,13*S*,14*S*)-13-methyl-3-vinyl-6,7,8,9,11,12,13,14,15,16-decahydro-17*H*-cyclopenta[*a*]phenanthren-17-one (**S3,** 38.0 mg, 0.14 mmol, 1.0 equiv.) and silica gel (76.0 mg) in DCM (0.1 M), phosphorus tribromide (12.7μL, 0.14 mmol, 1.0 equiv.) was added dropwise. The reaction mixture was stirred for 20 minutes. Afterward, it was filtered and rinsed with DCM, and the filtrate was washed with saturated $NaHCO_3$ solution and brine. The combined layers were dried over $MgSO_4$, filtered and concentrated under reduced pressure to afford the product **3ab** as a viscous solid in 79% yield.

^{1}H NMR (300 MHz, $CDCl_3$) δ 7.31 – 7.20 (m, 2H), 7.17 (s, 1H), 5.19 (q, *J* = 6.9 Hz, 1H), 2.92 (dd, *J* = 8.9, 4.3 Hz, 2H), 2.59 – 2.37 (m, 2H), 2.37 – 2.09 (m, 3H), 2.04 (d, *J* = 6.9 Hz, 3H), 2.01 – 1.90 (m, 1H), 1.78 – 1.35 (m, 6H), 1.26 (s, 1H), 0.91 (s, 3H).

Spectroscopic data are consistent with those reported in the literature.[11]

3.5. Photochemical synthesis of phenyl(tetrahydrofuran-2-yl)methanone (2)

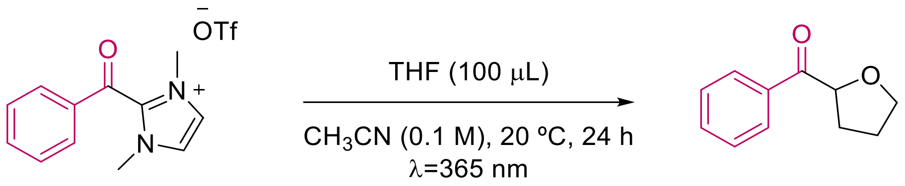

1a **2**, 28%

In a vial equipped with a magnetic stir bar, 2-benzoyl-1,3-dimethyl-1H-imidazol-3-ium trifluoromethanesulfonate **1a** (35.0 mg, 0.1 mmol) was dissolved in dry acetonitrile (5 mL, 0.01 M). Then, THF (100 µL, 1.23 mmol) was added. The reaction mixture was degassed using freeze-pump-thaw cycling (3 cycles, 10 min each under vacuum). Afterward, the mixture was stirred and irradiated at 365 nm for 24 h. Upon completion, the vial was removed from the light source, and DBU (15 µL, 0.1 mmol) was added. The reaction was stirred for an additional 10 minutes, then concentrated under reduced pressure and purified by flash chromatography on silica gel to afford phenyl(tetrahydrofuran-2-yl)methanone **2** (4.9 mg, 28%) as a colorless oil.

^{1}H NMR (300 MHz, $CDCl_3$) δ 8.02 – 7.97 (m, 2H), 7.64 – 7.59 (m, 1H), 7.47 (t, *J* = 7.5 Hz, 2H), 5.26 (dd, *J* = 8.4, 5.9 Hz, 1H), 4.09 – 3.92 (m, 2H), 2.34 – 2.25 (m, 1H), 2.20 – 2.11 (m, 1H), 2.03 – 1.92 (m, 2H).

Spectroscopic data are consistent with those reported in the literature.

3.6. General procedure D: Synthesis of acylated products

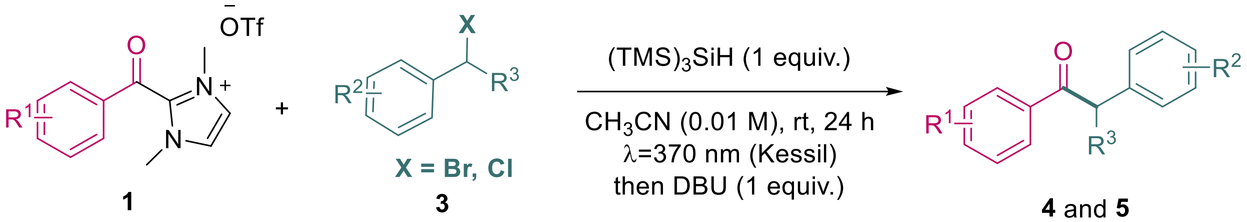

In a vial equipped with a magnetic stir bar, the corresponding dimethylacylimidazolium salt (0.1 mmol, 2 equiv.) was dissolved in dry acetonitrile (5 mL, 0.01 M). The corresponding alkyl halide (0.05 mmol) and $(TMS)_3SiH$ (0.05 mmol, 1 equiv.) were then added. The reaction mixture was degassed using freeze-pump-thaw cycling (3 cycles, 10 min each under vacuum). Afterward, the mixture was stirred and irradiated with two Kessil PhotoReaction PR160l LEDs (370 nm, 100% intensity, 3.0 cm distance) for 24 h. Upon completion, the vial was removed from the light source, and DBU (8 µL, 1 equiv.) was added. The reaction was stirred for an additional 10 minutes, then concentrated under reduced pressure and purified by flash chromatography on silica gel to afford the desired product.

1,2-diphenylpropan-1-one (4a)

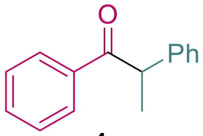

4a

Following the **general procedure D**, 2-benzoyl-1,3-dimethyl-1H-imidazol-3-ium trifluoromethanesulfonate **1a** (35.0 mg, 0.1 mmol) and (1-bromoethyl)benzene (6.8 µL, 0.05 mmol) gave product **3a** (10.2 mg, 97%) as a colorless oil after purification (Cyclohexane:EtOAc = 98:2). Alternatively, using (1-chloroethyl)benzene (8.7 µL, 0.05 mmol) instead of (1-bromoethyl)benzene gave product **4a** in a 27% NMR yield.

^{1}H NMR (300 MHz, $CDCl_3$) δ 7.98 – 7.92 (m, 2H), 7.52 – 7.44 (m, 1H), 7.42 – 7.34 (m, 2H), 7.32 – 7.27 (m, 4H), 7.25 – 7.15 (m, 1H), 4.69 (q, J = 6.9 Hz, 1H), 1.54 (d, J = 6.9 Hz, 3H).

Spectroscopic data are consistent with those reported in the literature.[12]

2-phenyl-1-(4-(trifluoromethyl)phenyl)propan-1-one (4b)

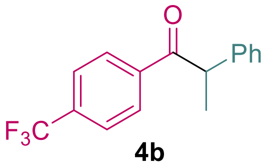

4b

Following the **general procedure D**, 1,3-dimethyl-2-(4-(trifluoromethyl)benzoyl)-1H-imidazol-3-ium trifluoromethanesulfonate **1b** (41.8 mg, 0.1 mmol) and (1-bromoethyl)benzene **3a** (6.8 µL, 0.05 mmol) gave product **4b** (13.1 mg, 94%) as a colorless oil after purification (Cyclohexane:EtOAc = 98:2).

^{1}H NMR (300 MHz, $CDCl_3$) δ 8.02 (d, J = 8.2 Hz, 2H), 7.63 (d, J = 8.2 Hz, 2H), 7.35 – 7.28 (m, 3H), 7.25 – 7.18 (m, 2H), 4.65 (q, J = 6.8 Hz, 1H), 1.55 (d, J = 6.8 Hz, 3H).

Spectroscopic data are consistent with those reported in the literature.[13]

1-(3,5-bis(trifluoromethyl)phenyl)-2-phenylpropan-1-one (4c)

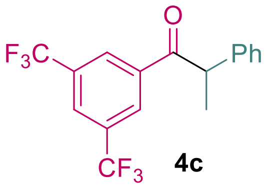
4c

Following the **general procedure D**, 2-(3,5-bis(trifluoromethyl)benzoyl)-1,3-dimethyl-1H-imidazol-3-ium trifluoromethanesulfonate **1c** (48.6 mg, 0.1 mmol) and (1-bromoethyl)benzene **3a** (6.8 μL, 0.05 mmol) gave product **4c** (11.6 mg, 67%) as a white solid after purification (Cyclohexane:EtOAc = 98:2).

^{1}H NMR (300 MHz, $CDCl_3$) δ 8.35 (s, 2H), 7.95 (s, 1H), 7.36 – 7.29 (m, 2H), 7.27 – 7.20 (m, 3H), 4.64 (q, *J* = 6.8 Hz, 1H), 1.58 (d, *J* = 6.8 Hz, 3H).

^{13}C{^{1}H} NMR (125 MHz, $CDCl_3$) δ 197.4, 140.2, 137.9, 132.3 (q, *J* = 33.9 Hz), 129.6, 128.9, 127.8, 127.7, 126.1 – 126.0 (m), 123.0 (q, *J* = 272.8 Hz), 48.8, 19.3.

^{19}F{^{1}H} NMR (282 MHz, $CDCl_3$) δ -63.1.

HRMS (ESI$^+$) Calculated for $C_{17}H_{13}F_6O$ $[M + H]^+$: 347.0792, found: 347.0798

4-(2-phenylpropanoyl)benzonitrile (4d)

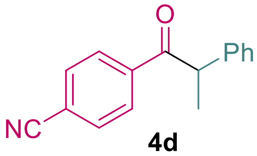
4d

Following the **general procedure D**, 2-(4-cyanobenzoyl)-1,3-dimethyl-1H-imidazol-3-ium trifluoromethanesulfonate **1d** (37.5 mg, 0.1 mmol) and (1-bromoethyl)benzene **3a** (6.8 μL, 0.05 mmol) gave product **4d** (7.9 mg, 67%) as a colorless oil after purification (Cyclohexane:EtOAc = 95:5).

^{1}H NMR (300 MHz, $CDCl_3$) δ 8.01 – 7.96 (m, 2H), 7.69 – 7.63 (m, 2H), 7.35 – 7.27 (m, 2H), 7.25 – 7.19 (m, 3H), 4.61 (q, *J* = 6.8 Hz, 1H), 1.54 (d, *J* = 6.8 Hz, 3H).

Spectroscopic data are consistent with those reported in the literature.[14]

1-(4-chlorophenyl)-2-phenylpropan-1-one (4e)

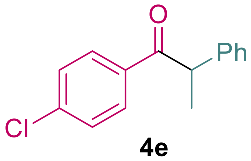
4e

Following the **general procedure D**, 2-(4-chlorobenzoyl)-1,3-dimethyl-1H-imidazol-3-ium trifluoromethanesulfonate **1e** (38.5 mg, 0.1 mmol) and (1-bromoethyl)benzene **3a** (6.8 μL, 0.05 mmol) gave product **4e** (4.9 mg, 40%) as a yellow oil after purification (Cyclohexane:EtOAc = 95:5).

^{1}H NMR (300 MHz, $CDCl_3$) δ 7.87 (m, 2H), 7.37 – 7.27 (m, 4H), 7.24 – 7.17 (m, 3H), 4.61 (q, *J* = 6.9 Hz, 1H), 1.52 (d, *J* = 6.9 Hz, 3H).

Spectroscopic data are consistent with those reported in the literature.[15]

1-(2-chlorophenyl)-2-phenylpropan-1-one (4f)

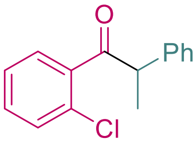
4f

Following the **general procedure D**, 2-(2-chlorobenzoyl)-1,3-dimethyl-1H-imidazol-3-ium trifluoromethanesulfonate **1f** (38.5 mg, 0.1 mmol) and (1-bromoethyl)benzene **3a** (6.8 μL, 0.05 mmol) gave product **4f** (6.1 mg, 50%) as a colorless oil after purification (Cyclohexane:EtOAc = 95:5).

^{1}**H NMR** (300 MHz, $CDCl_3$) δ 7.37 – 7.27 (m, 3H), 7.25 – 7.19 (m, 4H), 7.21 – 7.10 (m, 1H), 7.07 (m, 1H), 4.52 (q, *J* = 6.9 Hz, 1H), 1.58 (d, *J* = 6.9 Hz, 3H).

Spectroscopic data are consistent with those reported in the literature.[16]

1-(4-bromophenyl)-2-phenylpropan-1-one (4g)

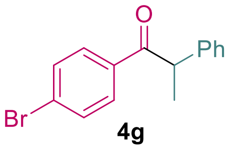

4g

Following the **general procedure D**, 2-(4-bromobenzoyl)-1,3-dimethyl-1H-imidazol-3-ium trifluoromethanesulfonate **1g** (42.9 mg, 0.1 mmol) and (1-bromoethyl)benzene **3a** (6.8 µL, 0.05 mmol) gave product **4g** (6.3 mg, 44%) as a white solid after purification (Cyclohexane:EtOAc = 95:5).

^{1}**H NMR** (300 MHz, $CDCl_3$) δ 7.80 (d, *J* = 8.6 Hz, 2H), 7.51 (d, *J* = 8.6 Hz, 2H), 7.33 – 7.27 (m, 2H), 7.25 – 7.19 (m, 3H), 4.60 (q, *J* = 6.8 Hz, 1H), 1.52 (d, *J* = 6.8 Hz, 3H).

Spectroscopic data are consistent with those reported in the literature.[15]

2-phenyl-1-(*p*-tolyl)propan-1-one (4i)

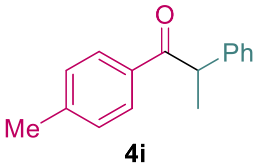

4i

Following the **general procedure D**, 1,3-dimethyl-2-(4-methylbenzoyl)-1H-imidazol-3-ium trifluoromethanesulfonate **1i** (36.4 mg, 0.1 mmol) and (1-bromoethyl)benzene **3a** (6.8 µL, 0.05 mmol) gave product **4i** (8.1 mg, 72%) as a yellow oil after purification (Cyclohexane:EtOAc = 95:5).

^{1}**H NMR** (300 MHz, $CDCl_3$) δ 7.89 – 7.82 (m, 2H), 7.30 – 7.27 (m, 4H), 7.23 – 7.14 (m, 3H), 4.66 (q, *J* = 6.9 Hz, 1H), 2.35 (s, 3H), 1.52 (d, *J* = 6.9 Hz, 3H).

Spectroscopic data are consistent with those reported in the literature.[15]

1-(furan-2-yl)-2-phenylpropan-1-one (4j)

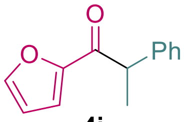

4j

Following the **general procedure D**, 2-(4-bromobenzoyl)-1,3-dimethyl-1H-imidazol-3-ium trifluoromethanesulfonate **1j** (42.9 mg, 0.1 mmol) and (1-bromoethyl)benzene **3a** (6.8 µL, 0.05 mmol) gave product **4j** (2.8 mg, 28%) as a white solid after purification (Cyclohexane:EtOAc = 95:5).

^{1}**H NMR** (300 MHz, $CDCl_3$) δ 7.52 (dd, *J* = 1.7, 0.8 Hz, 1H), 7.35 – 7.28 (m, 4H), 7.24 – 7.19 (m, 1H), 7.13 (dd, *J* = 3.6, 0.8 Hz, 1H), 6.46 (dd, *J* = 3.6, 1.7 Hz, 1H), 4.49 (q, *J* = 7.0 Hz, 1H), 1.53 (d, *J* = 7.0 Hz, 3H).

Spectroscopic data are consistent with those reported in the literature.[17]

1-phenyl-2-(4-(trifluoromethyl)phenyl)propan-1-one (4k)

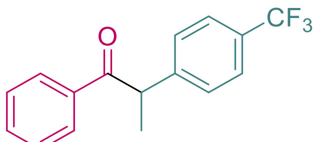

4k

Following the **general procedure D**, 2-benzoyl-1,3-dimethyl-1H-imidazol-3-ium trifluoromethanesulfonate **1a** (35.0 mg, 0.1 mmol) and 1-(1-bromoethyl)-4-(trifluoromethyl)benzene **3b** (13.3 mg, 0.05 mmol) gave product **4k** (10.1 mg, 73%) as a colorless oil after purification (Cyclohexane:EtOAc = 95:5).

^{1}H NMR (300 MHz, $CDCl_3$) δ 7.98 – 7.89 (m, 2H), 7.59 – 7.47 (m, 3H), 7.45 – 7.36 (m, 4H), 4.77 (q, *J* = 6.9 Hz, 1H), 1.56 (d, *J* = 6.9 Hz, 3H).

Spectroscopic data are consistent with those reported in the literature.[16]

2-(4-fluorophenyl)-1-phenylpropan-1-one (4l)

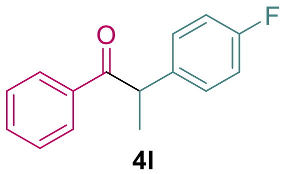

4l

Following the **general procedure D**, 2-benzoyl-1,3-dimethyl-1H-imidazol-3-ium trifluoromethanesulfonate **1a** (35.0 mg, 0.1 mmol) and 1-(1-bromoethyl)-4-fluorobenzene **3c** (7.0 μL, 0.05 mmol) gave product **4l** (7.5 mg, 66%) as a colorless oil after purification (Cyclohexane:EtOAc = 95:5).

^{1}H NMR (300 MHz, $CDCl_3$) δ 7.97 – 7.90 (m, 2H), 7.53 – 7.46 (m, 1H), 7.43 – 7.36 (m, 2H), 7.28 – 7.22 (m, 2H), 7.02 – 6.94 (m, 2H), 4.68 (q, *J* = 6.8 Hz, 1H), 1.52 (d, *J* = 6.9 Hz, 3H).

Spectroscopic data are consistent with those reported in the literature.[18]

Methyl 4-(1-oxo-1-phenylpropan-2-yl)benzoate (4m)

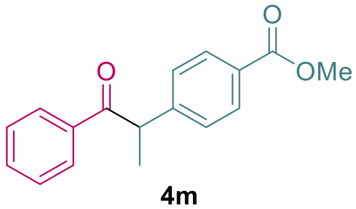

4m

Following the **general procedure D**, 2-benzoyl-1,3-dimethyl-1H-imidazol-3-ium trifluoromethanesulfonate **1a** (35.0 mg, 0.1 mmol) and methyl 4-(1-bromoethyl)benzoate **3d** (12.1 mg, 0.05 mmol) gave product **4m** (10.2 mg, 76%) as a white solid after purification (Cyclohexane:EtOAc = 95:5).

^{1}H NMR (300 MHz, $CDCl_3$) δ 8.01 – 7.89 (m, 4H), 7.53 – 7.45 (m, 1H), 7.42 – 7.32 (m, 4H), 4.74 (q, *J* = 6.9 Hz, 1H), 3.88 (s, 3H), 1.55 (d, *J* = 6.9 Hz, 3H).

Spectroscopic data are consistent with those reported in the literature.[19]

1-Phenyl-2-(2-(trifluoromethyl)phenyl)propan-1-one (4n)

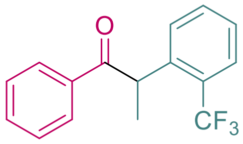

4n

Following the **general procedure D**, 2-benzoyl-1,3-dimethyl-1H-imidazol-3-ium trifluoromethanesulfonate **1a** (35.0 mg, 0.1 mmol) and 1-(1-bromoethyl)-2-(trifluoromethyl)benzene **3e** (8.9 μL, 0.05 mmol) gave product **4n** (8.0 mg, 57%) as a colorless oil after purification (Cyclohexane:EtOAc = 95:5).

^{1}H NMR (300 MHz, $CDCl_3$) δ 7.95 – 7.81 (m, 2H), 7.69 (d, *J* = 7.5 Hz, 1H), 7.52 – 7.28 (m, 6H), 5.04 (q, *J* = 6.7 Hz, 1H), 1.55 (d, *J* = 6.9 Hz, 3H).

Spectroscopic data are consistent with those reported in the literature.[20]

2-(2-fluorophenyl)-1-phenylpropan-1-one (4o)

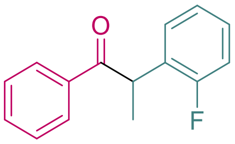

4o

Following the **general procedure D**, 2-benzoyl-1,3-dimethyl-1H-imidazol-3-ium trifluoromethanesulfonate **1a** (35.0 mg, 0.1 mmol) and 1-(1-bromoethyl)-2-fluorobenzene **3f** (7.2 µL, 0.05 mmol) gave product **4o** (8.0 mg, 70%) as a colorless oil after purification (Cyclohexane:EtOAc = 95:5).

^{1}H NMR (300 MHz, $CDCl_3$) δ 7.99 – 7.93 (m, 2H), 7.53 – 7.45 (m, 1H), 7.44 – 7.35 (m, 2H), 7.23 – 7.14 (m, 2H), 7.10 – 7.01 (m, 2H), 5.03 (q, *J* = 6.9 Hz, 1H), 1.52 (d, *J* = 6.9 Hz, 3H).

Spectroscopic data are consistent with those reported in the literature.[21]

1-phenyl-2-(3-(trifluoromethyl)phenyl)propan-1-one (4p)

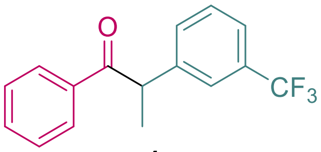

4p

Following the **general procedure D**, 2-benzoyl-1,3-dimethyl-1H-imidazol-3-ium trifluoromethanesulfonate **1a** (35.0 mg, 0.1 mmol) and 1-(1-bromoethyl)-3-(trifluoromethyl)benzene **3g** (9.1 µL, 0.05 mmol) gave product **4p** (9.3 mg, 67%) as a colorless oil after purification (Cyclohexane:EtOAc = 95:5).

^{1}H NMR (300 MHz, $CDCl_3$) δ 7.98 – 7.90 (m, 2H), 7.57 (s, 1H), 7.55 – 7.38 (m, 6H), 4.78 (q, *J* = 6.9 Hz, 1H), 1.57 (d, *J* = 6.9 Hz, 3H).

^{13}C{^{1}H} NMR (75 MHz, $CDCl_3$) δ 199.9, 142.4, 136.3, 133.3, 131.6, 131.3, 131.2, 129.5, 128.8, 124.8 (q, *J* = 3.8 Hz), 124.1 (q, *J* = 272.5 Hz), 124.0 (q, *J* = 3.8 Hz), 47.5, 19.7.

^{19}F{^{1}H} NMR (282 MHz, $CDCl_3$) δ -62.6.

HRMS (ESI$^+$) Calculated for $C_{16}H_{14}F_3O$ $[M + H]^+$: 279.0991, found: 279.0956

1-phenyl-2-(p-tolyl)propan-1-one (4q)

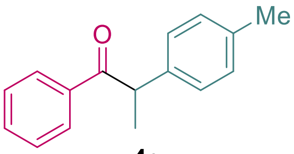

4q

Following the **general procedure D**, 2-benzoyl-1,3-dimethyl-1H-imidazol-3-ium trifluoromethanesulfonate **1a** (35.0 mg, 0.1 mmol) and 1-(1-bromoethyl)-4-methylbenzene **3h** (7.6 µL, 0.05 mmol) gave product **4q** (8.7 mg, 78%) as a colorless oil after purification (Cyclohexane:EtOAc = 98:2).

^{1}H NMR (300 MHz, $CDCl_3$) δ 7.98 – 7.91 (m, 2H), 7.51 – 7.42 (m, 1H), 7.41 – 7.33 (m, 2H), 7.20 – 7.06 (m, 4H), 4.65 (q, *J* = 6.9 Hz, 1H), 2.28 (s, 3H), 1.51 (d, *J* = 6.9 Hz, 3H).

Spectroscopic data are consistent with those reported in the literature.[12]

1-phenyl-2-(o-tolyl)propan-1-one (4r)

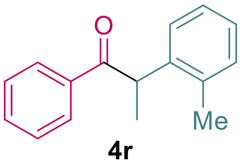

4r

Following the **general procedure D**, 2-benzoyl-1,3-dimethyl-1H-imidazol-3-ium trifluoromethanesulfonate **1a** (35.0 mg, 0.1 mmol) and 1-(1-bromoethyl)-2-methylbenzene **3i** (9.9 mg, 0.05 mmol) gave product **4r** (8.9 mg, 79%) as a colorless oil after purification (Cyclohexane:EtOAc = 98:2).

^{1}H NMR (300 MHz, $CDCl_3$) δ 7.84 – 7.80 (m, 2H), 7.49 – 7.42 (m, 1H), 7.39 – 7.31 (m, 2H), 7.22 – 7.18 (m, 1H), 7.13 – 7.00 (m, 3H), 4.76 (q, *J* = 6.8 Hz, 1H), 2.50 (s, 3H), 1.47 (d, *J* = 6.8 Hz, 3H).

Spectroscopic data are consistent with those reported in the literature.[12]

1-phenyl-2-(m-tolyl)propan-1-one (4s)

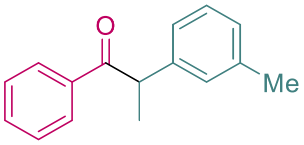

4s

Following the **general procedure D**, 2-benzoyl-1,3-dimethyl-1H-imidazol-3-ium trifluoromethanesulfonate **1a** (35.0 mg, 0.1 mmol) and 1-(1-bromoethyl)-3-methylbenzene **3j** (9.9 mg, 0.05 mmol) gave product **4s** (8.9 mg, 79%) as a colorless oil after purification (Cyclohexane:EtOAc = 98:2).

^{1}H NMR (300 MHz, $CDCl_3$) δ 8.00 – 7.91 (m, 2H), 7.51 – 7.44 (m, 1H), 7.41 – 7.34 (m, 2H), 7.21 – 7.15 (m, 1H), 7.11 – 7.08 (m, 2H), 7.03 – 6.99 (m, 1H), 4.65 (q, *J* = 6.9 Hz, 1H), 2.30 (s, 3H), 1.52 (d, *J* = 6.9 Hz, 3H).

Spectroscopic data are consistent with those reported in the literature.[12]

1,2-diphenylethan-1-one (4t)

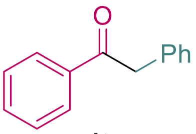

4t

Following the **general procedure D**, 2-benzoyl-1,3-dimethyl-1H-imidazol-3-ium trifluoromethanesulfonate **1a** (35.0 mg, 0.1 mmol) and benzyl bromide **3k** (6.0 µL, 0.05 mmol) gave product **4t** (7.5 mg, 76%) as a white solid after purification (Cyclohexane:EtOAc = 98:2).

^{1}H NMR (500 MHz, $CDCl_3$) δ 8.03 – 8.00 (m, 2H), 7.58 – 7.54 (m, 1H), 7.48 – 7.44 (m, 2H), 7.35 – 7.31 (m, 2H), 7.29 – 7.25 (m, 3H), 4.29 (s, 2H).

Spectroscopic data are consistent with those reported in the literature.[12]

1-Phenyl-2-(4-(trifluoromethyl)phenyl)ethan-1-one (4u)

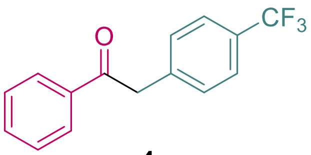

4u

Following the **general procedure D**, 2-benzoyl-1,3-dimethyl-1H-imidazol-3-ium trifluoromethanesulfonate **1a** (35.0 mg, 0.1 mmol) and 1-(bromomethyl)-4-(trifluoromethyl)benzene **3l** (11.9 mg, 0.05 mmol) gave product **4u** (7.8 mg, 59%) as a white solid after purification (Cyclohexane:EtOAc = 95:5).

^{1}H NMR (300 MHz, $CDCl_3$) δ 8.04 – 7.99 (m, 2H), 7.63 – 7.56 (m, 3H), 7.53 – 7.45 (m, 2H), 7.42 – 7.35 (m, 2H), 4.36 (s, 2H).

Spectroscopic data are consistent with those reported in the literature.[22]

4-(2-oxo-2-phenylethyl)benzonitrile (4v)

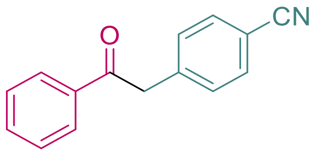

4v

Following the **general procedure D**, 2-benzoyl-1,3-dimethyl-1H-imidazol-3-ium trifluoromethanesulfonate **1a** (35.0 mg, 0.1 mmol) and 4-(bromomethyl)benzonitrile **3m** (9.8 mg, 0.05 mmol) gave product **4v** (3.6 mg, 33%) as a white solid after purification (Cyclohexane:EtOAc = 92:8).

^{1}H NMR (300 MHz, $CDCl_3$) δ 8.04 – 7.97 (m, 2H), 7.66 – 7.57 (m, 3H), 7.53 – 7.45 (m, 2H), 7.41 – 7.34 (m, 2H), 4.36 (s, 2H).

Spectroscopic data are consistent with those reported in the literature.[23]

Methyl 4-(2-oxo-2-phenylethyl)benzoate (4w)

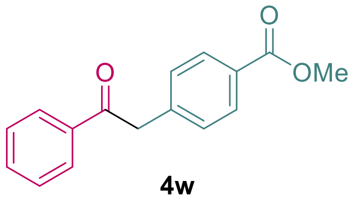

4w

Following the **general procedure D**, 2-benzoyl-1,3-dimethyl-1H-imidazol-3-ium trifluoromethanesulfonate **1a** (35.0 mg, 0.1 mmol) and methyl 4-(bromomethyl)benzoate **3n** (11.4 mg, 0.05 mmol) gave product **4w** (7.5 mg, 59%) as a white solid after purification (Cyclohexane:EtOAc = 92:8).

^{1}H NMR (300 MHz, $CDCl_3$) δ 8.04 – 7.97 (m, 4H), 7.58 (m, 1H), 7.51 – 7.43 (m, 2H), 7.38 – 7.31 (m, 2H), 4.35 (s, 2H), 3.90 (s, 3H).

Spectroscopic data are consistent with those reported in the literature.[18]

2-(4-bromophenyl)-1-phenylethan-1-one (4x)

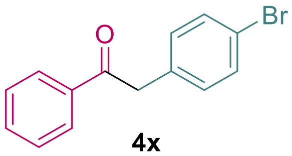

4x

Following the **general procedure D**, 2-benzoyl-1,3-dimethyl-1H-imidazol-3-ium trifluoromethanesulfonate **1a** (35.0 mg, 0.1 mmol) and 4-bromobenzyl bromide **3o** (12.5 mg, 0.05 mmol) gave product **4x** (17.0 mg, 62%) as a white solid after purification (Cyclohexane:EtOAc = 95:5).

^{1}H NMR (300 MHz, $CDCl_3$) δ 8.02 – 7.97 (m, 2H), 7.61 – 7.55 (m, 1H), 7.51 – 7.43 (m, 4H), 7.14 (d, J = 8.3 Hz, 2H), 4.24 (s, 2H).

Spectroscopic data are consistent with those reported in the literature.[24]

2-(3,5-bis(trifluoromethyl)phenyl)-1-phenylethan-1-one (4y)

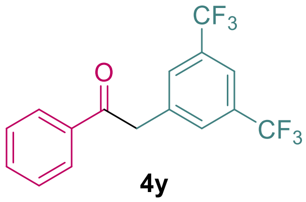

4y

Following the **general procedure D**, 2-benzoyl-1,3-dimethyl-1H-imidazol-3-ium trifluoromethanesulfonate **1a** (35.0 mg, 0.1 mmol) and 1-(bromomethyl)-3,5-bis(trifluoromethyl)benzene **3p** (9.2 μL, 0.05 mmol) gave product **4y** (7.1 mg, 43%) as a white solid after purification (Cyclohexane:EtOAc = 95:5).

^{1}**H NMR** (300 MHz, $CDCl_3$) δ 8.06 – 8.00 (m, 2H), 7.81 (s, 1H), 7.72 (s, 2H), 7.67 – 7.60 (m, 1H), 7.57 – 7.49 (m, 2H), 4.45 (s, 2H).

Spectroscopic data are consistent with those reported in the literature.[25]

2-(4-(methylthio)phenyl)-1-phenylethan-1-one (4z)

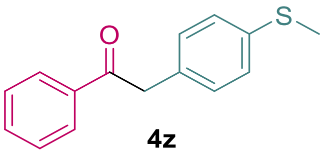

4z

Following the **general procedure D**, 2-benzoyl-1,3-dimethyl-1H-imidazol-3-ium trifluoromethanesulfonate **1a** (35.0 mg, 0.1 mmol) and (4-(bromomethyl)phenyl)(methyl)sulfane **3q** (10.9 mg, 0.05 mmol) gave product **4z** (5.1 mg, 42%) as a white solid after purification (Cyclohexane:EtOAc = 95:5).

^{1}**H NMR** (300 MHz, $CDCl_3$) δ 8.04 – 7.96 (m, 2H), 7.60 – 7.52 (m, 1H), 7.50 – 7.42 (m, 2H), 7.25 – 7.16 (m, 4H), 4.24 (s, 2H), 2.46 (s, 3H).

Spectroscopic data are consistent with those reported in the literature.[23]

2-(4-(*tert*-butyl)phenyl)-1-phenylethan-1-one (4aa)

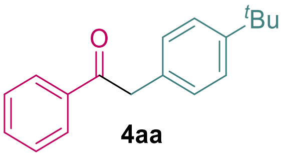

4aa

Following the **general procedure D**, 2-benzoyl-1,3-dimethyl-1H-imidazol-3-ium trifluoromethanesulfonate **1a** (35.0 mg, 0.1 mmol) and 1-(bromomethyl)-4-(*tert*-butyl)benzene **3r** (9.2 μL, 0.05 mmol) gave product **4aa** (8.8 mg, 70%) as a white solid after purification (Cyclohexane:EtOAc = 98:2). Alternatively, using 1-(tert-butyl)-4-(chloromethyl)benzene (9.7 μL, 0.05 mmol) instead of 1-(bromomethyl)-4-(*tert*-butyl)benzene gave product **4aa** in a 10% NMR yield.

^{1}**H NMR** (300 MHz, $CDCl_3$) δ 8.06 – 8.00 (m, 2H), 7.60 – 7.52 (m, 1H), 7.51 – 7.42 (m, 2H), 7.38 – 7.32 (m, 2H), 7.23 – 7.18 (m, 2H), 4.26 (s, 2H), 1.30 (s, 9H).

Spectroscopic data are consistent with those reported in the literature.[22]

2-(3,5-dimethylphenyl)-1-phenylethan-1-one (4ab)

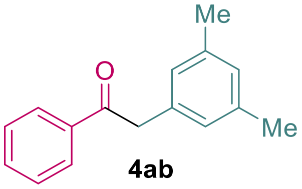

4ab

Following the **general procedure D**, 2-benzoyl-1,3-dimethyl-1H-imidazol-3-ium trifluoromethanesulfonate **1a** (35.0 mg, 0.1 mmol) and 1-(bromomethyl)-3,5-dimethylbenzene **3s** (10.0 mg, 0.05 mmol) gave product **4ab** (7.9 mg, 70%) as a colorless oil after purification (Cyclohexane:EtOAc = 95:5).

^{1}**H NMR** (300 MHz, $CDCl_3$) δ 8.05 – 7.99 (m, 2H), 7.59 – 7.52 (m, 1H), 7.50 – 7.42 (m, 2H), 6.91 – 6.86 (m, 3H), 4.21 (s, 2H), 2.29 (s, 6H).

Spectroscopic data are consistent with those reported in the literature.[26]

2-([1,1'-biphenyl]-4-yl)-1-phenylethan-1-one (4ac)

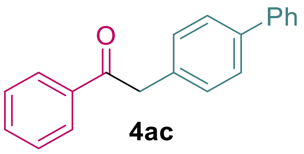

4ac

Following the **general procedure D**, 2-benzoyl-1,3-dimethyl-1H-imidazol-3-ium trifluoromethanesulfonate **1a** (35.0 mg, 0.1 mmol) and 4-(bromomethyl)-1,1'-biphenyl **3t** (12.4 mg, 0.05 mmol) gave product **4ac** (7.8 mg, 57%) as a colorless oil after purification (Cyclohexane:EtOAc = 95:5).

^{1}H NMR (300 MHz, $CDCl_3$) δ 8.09 – 8.01 (m, 2H), 7.61 – 7.53 (m, 5H), 7.52 – 7.39 (m, 4H), 7.35 m, 3H), 4.34 (s, 2H).

Spectroscopic data are consistent with those reported in the literature.[22]

2-(naphthalen-2-yl)-1-phenylethan-1-one (4ad)

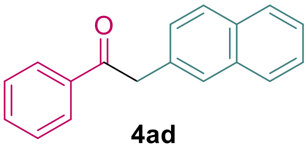

4ad

Following the **general procedure D**, 2-benzoyl-1,3-dimethyl-1H-imidazol-3-ium trifluoromethanesulfonate **1a** (35.0 mg, 0.1 mmol) and 2-(bromomethyl)naphthalene **3u** (11.0 mg, 0.05 mmol) gave product **4ad** (4.0 mg, 32%) as a colorless oil after purification (Cyclohexane:EtOAc = 95:5).

^{1}H NMR (300 MHz, $CDCl_3$) δ 8.08 – 8.03 (m, 2H), 7.84 – 7.77 (m, 3H), 7.73 (s, 1H), 7.59 – 7.53 (m, 1H), 7.49 – 7.43 (m, 4H), 7.40 (m, 1H), 4.45 (s, 2H).

Spectroscopic data are consistent with those reported in the literature.[27]

5-(2-oxo-2-phenylethyl)picolinonitrile (4ae)

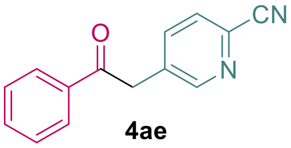

4ae

Following the **general procedure D**, 2-(4-bromobenzoyl)-1,3-dimethyl-1H-imidazol-3-ium trifluoromethanesulfonate **1a** (42.9 mg, 0.1 mmol) and 5-(bromomethyl)picolinonitrile **3v** (9.9 mg, 0.05 mmol) gave product **3ae** (4.1 mg, 37%) as a white solid after purification (Cyclohexane:EtOAc = 80:20).

^{1}H NMR (300 MHz, $CDCl_3$) δ 8.61 (d, *J* = 2.2 Hz, 1H), 8.04 – 8.00 (m, 2H), 7.76 (dd, *J* = 8.0, 2.2 Hz, 1H), 7.69 (dd, *J* = 8.0, 0.9 Hz, 1H), 7.67 – 7.61 (m, 1H), 7.56 – 7.49 (m, 2H), 4.40 (s, 2H).

^{13}C{H} NMR (75 MHz, $CDCl_3$) δ 195.1, 152.3, 138.5, 136.1, 134.5, 134.2, 132.6, 129.1, 128.5, 128.2, 117.3, 42.3.

HRMS (ESI$^+$) Calculated for $C_{14}H_{11}N_2O$ [M + H]+ : 223 .0866, found: 223.0887

1,2-diphenylbutan-1-one (4af)

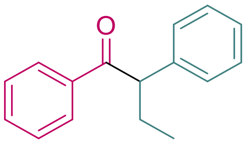

4af

Following the **general procedure D**, 2-benzoyl-1,3-dimethyl-1H-imidazol-3-ium trifluoromethanesulfonate **1a** (35.0 mg, 0.1 mmol) and (1-bromopropyl)benzene **3w** (9.9 mg, 0.05 mmol) gave product **4af** (6.4 mg, 57%) as a white solid after purification (Cyclohexane:EtOAc = 98:2).

^{1}H NMR (300 MHz, $CDCl_3$) δ 7.98 – 7.94 (m, 2H), 7.51 – 7.45 (m, 1H), 7.42 – 7.34 (m, 2H), 7.32 – 7.28 (m, 4H), 7.23 – 7.19 (m, 1H), 4.44 (t, *J* = 7.4 Hz, 1H), 2.31 – 2.11 (m, 1H), 1.94 – 1.82 (m, 1H), 0.91 (t, *J* = 7.4 Hz, 3H).

Spectroscopic data are consistent with those reported in the literature.[19]

3-methyl-1,2-diphenylbutan-1-one (4ag)

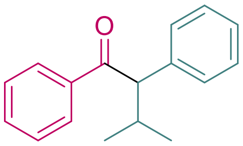

4ag

Following the **general procedure D**, 2-benzoyl-1,3-dimethyl-1H-imidazol-3-ium trifluoromethanesulfonate **1a** (35.0 mg, 0.1 mmol) and (1-bromo-2-methylpropyl)benzene **3x** (10.7 mg, 0.05 mmol) gave product **4ag** (8.1 mg, 68%) as a white solid after purification (Cyclohexane:EtOAc = 98:2).

^{1}H NMR (300 MHz, $CDCl_3$) δ 8.02 – 7.93 (m, 2H), 7.55 – 7.43 (m, 1H), 7.45 – 7.34 (m, 2H), 7.38 – 7.25 (m, 3H), 7.26 – 7.13 (m, 2H), 4.21 (d, *J* = 10.1 Hz, 1H), 2.59 (dhept, *J* = 10.q, 6.7 Hz, 1H), 1.01 (d, *J* = 6.7 Hz, 3H), 0.76 (d, *J* = 6.7 Hz, 3H).

Spectroscopic data are consistent with those reported in the literature.[28]

2-cyclohexyl-1,2-diphenylethan-1-one (4ah)

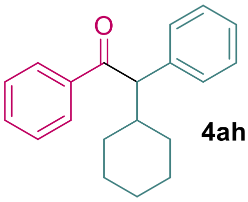

4ah

Following the **general procedure D**, 2-benzoyl-1,3-dimethyl-1H-imidazol-3-ium trifluoromethanesulfonate **1a** (35.0 mg, 0.1 mmol) and (bromo(cyclohexyl)methyl)benzene **3y** (12.7 mg, 0.05 mmol) gave product **4ah** (6.1 mg, 44%) as a colorless oil after purification (Cyclohexane:EtOAc = 98:2).

^{1}H NMR (300 MHz, $CDCl_3$) δ 7.99 – 7.95 (m, 2H), 7.52 – 7.46 (m, 1H), 7.43 – 7.27 (m, 5H), 7.23 – 7.15 (m, 2H), 4.31 (d, *J* = 10.2 Hz, 1H), 2.38 – 2.22 (m, 1H), 1.88 – 1.77 (m, 1H), 1.71 – 1.60 (m, 3H), 1.36 – 1.25 (m, 2H), 1.20 – 1.12 (m, 2H), 1.05 – 0.93 (m, 1H), 0.92 – 0.83 (m, 1H).

Spectroscopic data are consistent with those reported in the literature.[29]

1,2,2-triphenylethan-1-one (4ai)

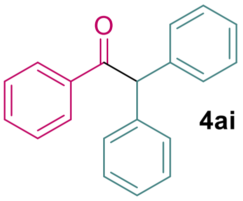

4ai

Following the **general procedure D**, 2-benzoyl-1,3-dimethyl-1H-imidazol-3-ium trifluoromethanesulfonate **1a** (35.0 mg, 0.1 mmol) and bromodiphenylmethane **3z** (12.4 mg, 0.05 mmol) gave product **4ai** (11.8 mg, 87%) as a yellow solid after purification (Cyclohexane:EtOAc = 95:5). Alternatively, using (chloromethylene)dibenzene (8.9 µL, 0.05 mmol) instead of bromodiphenylmethane gave product **4ai** in a 59% NMR yield.

^{1}H NMR (300 MHz, $CDCl_3$) δ 8.04 – 7.97 (m, 2H), 7.55 – 7.48 (m, 1H), 7.45 – 7.38 (m, 2H), 7.36 – 7.27 (m, 10H), 6.04 (s, 1H).

Spectroscopic data are consistent with those reported in the literature.[30]

(9*H*-fluoren-9-yl)(phenyl)methanone (4aj)

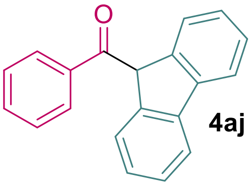

4aj

Following the **general procedure D**, 2-benzoyl-1,3-dimethyl-1H-imidazol-3-ium trifluoromethanesulfonate **1a** (35.0 mg, 0.1 mmol) and 9-Bromofluorene **3aa** (12.3 mg, 0.05 mmol) gave product **4aj** (5.2 mg, 39%) as a colorless oil after purification (Cyclohexane:EtOAc = 99:1).

^{1}H NMR (300 MHz, $CDCl_3$) δ 7.85 (d, *J* = 7.6 Hz, 2H), 7.75 – 7.71 (m, 2H), 7.53 – 7.50 (m, 1H), 7.47 – 7.35 (m, 6H), 7.30 – 7.27 (m, 2H), 5.61 (s, 1H).

Spectroscopic data are consistent with those reported in the literature.[31]

1-(3,6-dichloro-2-methoxyphenyl)-2-phenylpropan-1-one (6)

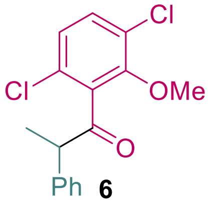

6

Following the **general procedure D**, 2-(3,6-dichloro-2-methoxybenzoyl)-1,3-dimethyl-1H-imidazol-3-ium trifluoromethanesulfonate **1k** (44.9 mg, 0.1 mmol) and (1-bromoethyl)benzene **3a** (6.8 µL, 0.05 mmol) gave product **6** (3.3 mg, 21%) as a colorless oil after purification (Cyclohexane:EtOAc = 95:5).

^{1}H NMR (300 MHz, $CDCl_3$) δ 7.28 – 7.18 (m, 6H), 6.99 (d, *J* = 8.6 Hz, 1H), 4.27 (q, *J* = 7.1 Hz, 1H), 3.67 (s, 3H), 1.57 (d, *J* = 7.1 Hz, 3H).

^{13}C NMR (125 MHz, $CDCl_3$) δ 202.7, 153.4, 138.6, 136.7, 131.6, 128.9, 128.8, 128.6, 128.5, 127.5, 126.2, 62.5, 53.8, 17.3.

HRMS (ESI$^+$) Calculated for $C_{16}H_{15}Cl_2O_2$ $[M + Na]^+$: 332.0263, found: 332.0259

2-phenyl-1-(4-(1-(5,5,8,8-tetramethyl-5,6,7,8-tetrahydronaphthalen-2-yl)vinyl)phenyl)propan-1-one (7)

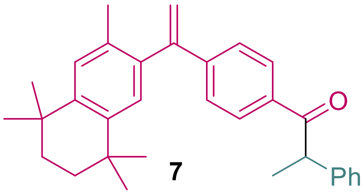

7

Following the **general procedure D**, 1,3-dimethyl-2-(4-(1-(3,5,5,8,8-pentamethyl 5,6,7,8-tetrahydronaphthalen-2-yl)vinyl)benzoyl)-1H-imidazol-3-ium trifluoromethanesulfonate **1l** (57.7 mg, 0.1 mmol) and (1-bromoethyl)benzene **3a** (6.8 µL, 0.05 mmol) gave product **7** (12.0 mg, 55%) as a white solid after purification (Cyclohexane:EtOAc = 98:2).

^{1}H NMR (300 MHz, $CDCl_3$) δ 7.87 (d, *J* = 8.5 Hz, 2H), 7.31 – 7.27 (m, 5H), 7.23 – 7.15 (m, 2H), 7.06 (m, 2H), 5.77 (d, *J* = 1.4 Hz, 1H), 5.28 (d, *J* = 1.4 Hz, 1H), 4.65 (q, *J* = 6.9 Hz, 1H), 1.91 (s, 3H), 1.58 – 1.49 (m, 7H), 1.29 (s, 6H), 1.25 (s, 6H).

^{13}C{^{1}H} NMR (125 MHz, $CDCl_3$) δ 200.0, 149.1, 145.4, 144.5, 142.4, 141.7, 138.1, 135.5, 132.8, 129.1, 129.1, 128.2, 128.1, 127.9, 127.0, 126.7, 117.0, 48.1, 35.3, 35.3, 34.1, 34.0, 32.1, 32.0, 32.0, 20.0, 19.7.

HRMS (ESI$^+$) Calculated for $C_{32}H_{37}O$ $[M + H]^+$: 437.2766, found: 437.2729

(8*R*,9*S*,13*S*,14*S*)-13-methyl-3-(1-oxo-1-phenylpropan-2-yl)-6,7,8,9,11,12,13,14,15,16-decahydro-17H-cyclopenta[*a*]phenanthren-17-one (8)

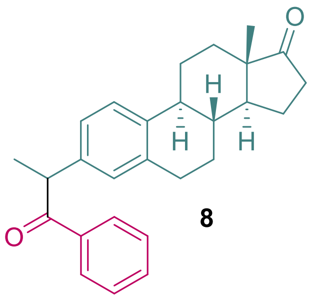

8

Following the **general procedure D**, 2-(4-bromobenzoyl)-1,3-dimethyl-1H-imidazol-3-ium trifluoromethanesulfonate **1a** (42.9 mg, 0.1 mmol) and (8*R*,9*S*,13*S*,14*S*)-3 (bromoethyl)-13-methyl-6,7,8,9,11,12,13,14,15,16-decahydro-17*H*-cyclopenta[*a*]phenanthren-17-one **3ab** (18.1 mg, 0.05 mmol) gave product **8** (8.5 mg, 46%) as a colorless oil after purification (Cyclohexane:EtOAc = 90:10).

^{1}H NMR (300 MHz, $CDCl_3$) δ 8.00 – 7.93 (m, 2H), 7.52 – 7.45 (m, 1H), 7.43 – 7.36 (m, 2H), 7.21 (dd, *J* = 8.2, 3.4 Hz, 1H), 7.11 – 7.05 (m, 1H), 7.03 – 6.98 (m, 1H), 4.64 (q, *J* = 6.8 Hz, 1H), 2.90 – 2.82 (m, 2H), 2.57 – 2.42 (m, 1H), 2.41 – 2.34 (m, 1H), 2.30 – 1.90 (m, 5H), 1.63 – 1.44 (m, 9H), 0.88 (s, 3H).

Spectroscopic data are consistent with those reported in the literature.[32]

5. Unsuccessful examples

5.1. Alkyl halides

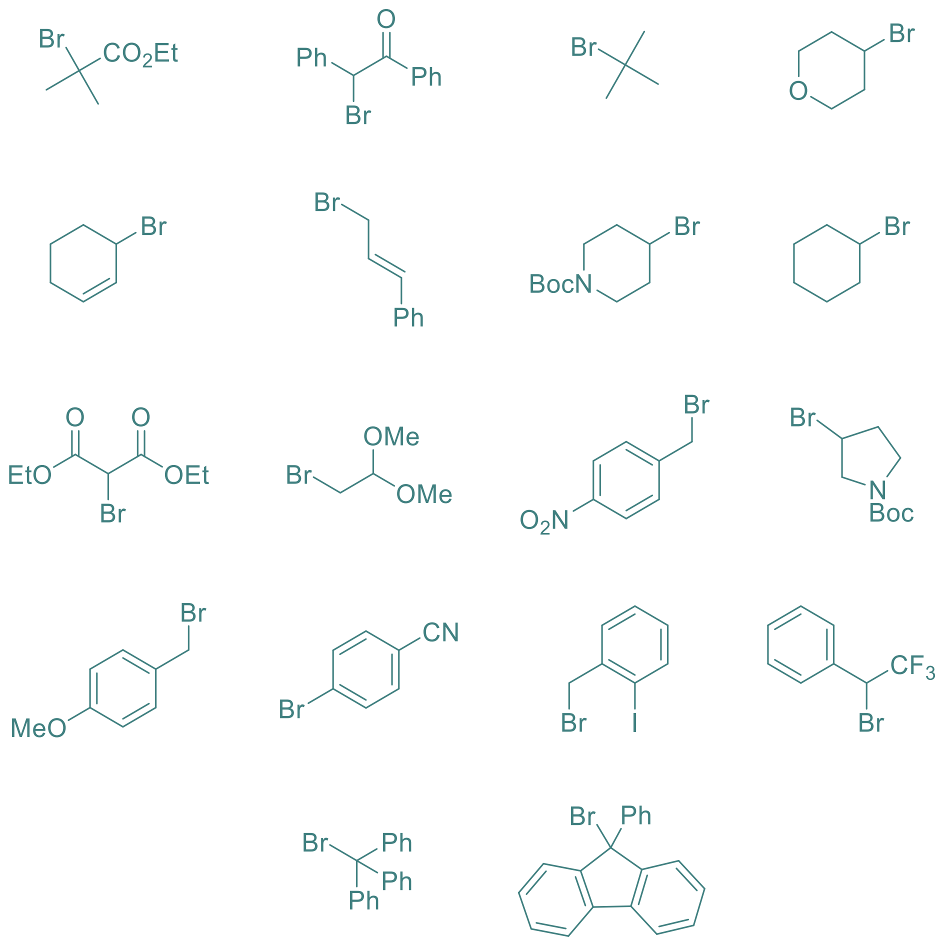

5.2. Acyl azolium salts

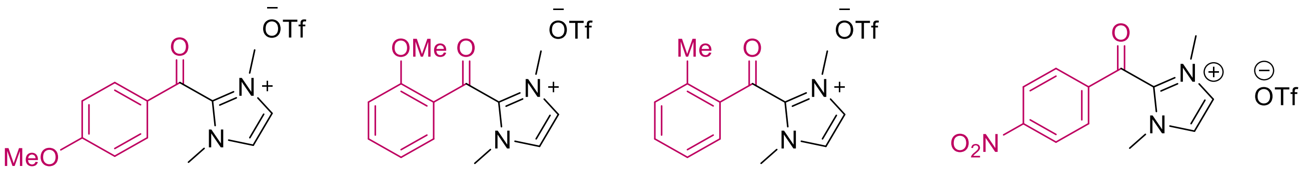

6. Spectroscopic studies

6.1. Determination of the triplet energy

Photoexcitation of **1a** in ACN is possible with wavelengths below 325 nm for which the molecule exhibits a significant absorbance. If cooled to 77 K, pronounced phosphorescence is observable (**Figure S7**, excitation wavelength: 275 nm, entrance slit: 1.75 nm, exit slit: 1.5 nm, integration time: 0.1s). The emission band lies in the spectral regime above 400 nm and shows a vibrational fine structure, which matches the C=O stretching vibration with a wavenumber of about 1600 cm^{-1}. The excitation spectrum (emission wavelength: 455 nm, entrance slit: 1.5 nm, exit slit: 1.5 nm, integration time: 0.1 s) is in good agreement with the absorption spectrum.

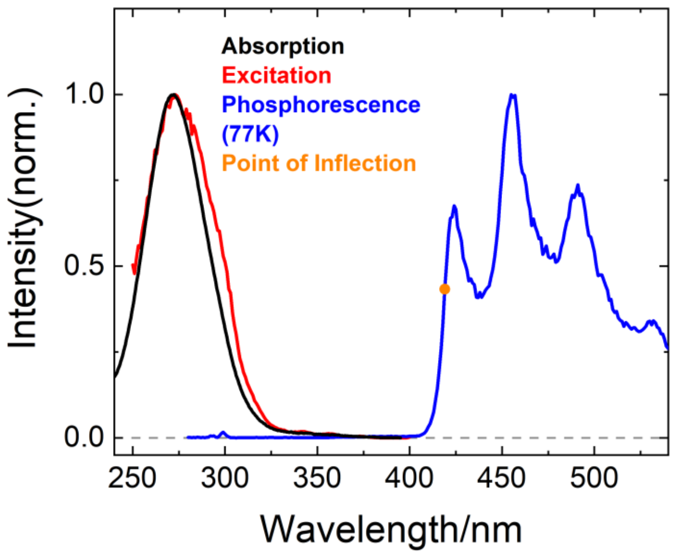

Figure S7*:* After photoexcitation, **1a** exhibits low-temperature phosphorescence (blue). The Phosphorescence spectrum shows a vibrational structure with a peak-distance around 1600 cm^{-1}, matching a C=O vibration. By determining the wavelength of the point of inflection on its short edge (orange), the triplet energy can be calculated. Absorption (black) and excitation (red) spectra are in good agreement.

By determining the point of inflection on the short wavelength edge of the phosphorescence[33] (419 nm), E_T can be determined to be 68.1 kcal/mol, which compares well with the value obtained from theory (66.12 kcal/mol)[34] and the triplet energy of benzophenone (68.3 kcal/mol)[35].

6.2. Determination of the phosphorescence rate constant

The phosphorescence lifetime can be obtained from a sample of **1a** in ACN at 77 K using time-correlated single photon counting (TCSPC)[36]. The resulting data is fitted using a single exponential convolved with a gaussian as the IRF[2] (**Figure S8**), yielding a lifetime of 6 ms, which compares well to benzophenone in a mixture of organic solvents (diethyl ether, isopentane and ethanol in ratio 5:5:2) at 77 K (6 ms)[37]. Since nonradiative rates are suppressed at low temperatures, the lifetime of **1a(T_1)** is shorter at room temperature.

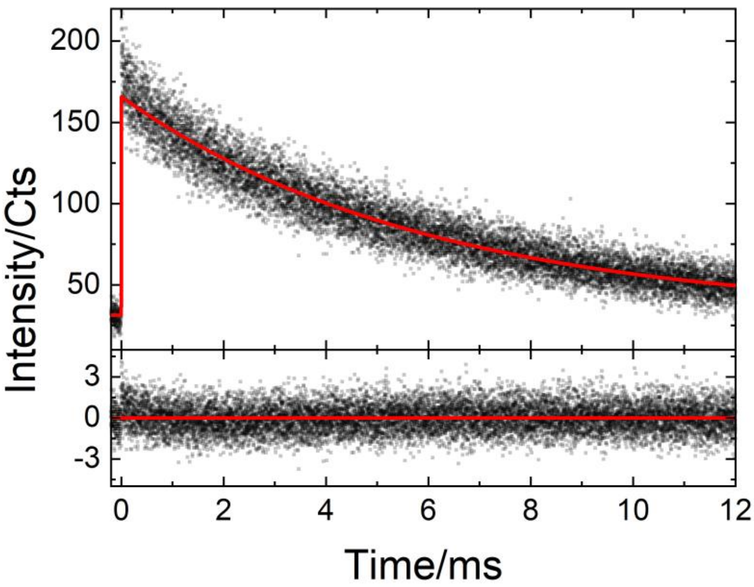

Figure S8*:* TCSPC data (top, black) of a sample of **1a** in ACN at 77 K. The fit (top, red) determines the phosphorescence lifetime to be 6 ms. The poisson-weighted residues are shown in the bottom. The datapoint at 0 contained scattering photons from the excitation source and was hence patched.[2]

6.3. Transient absorption and observation of the hydrogen atom transfer

Time-resolved absorption measurements of **1a** after photoexcitation reveal a broad absorption band, the formation of which coincides with the pump pulse (**Figure S9**).

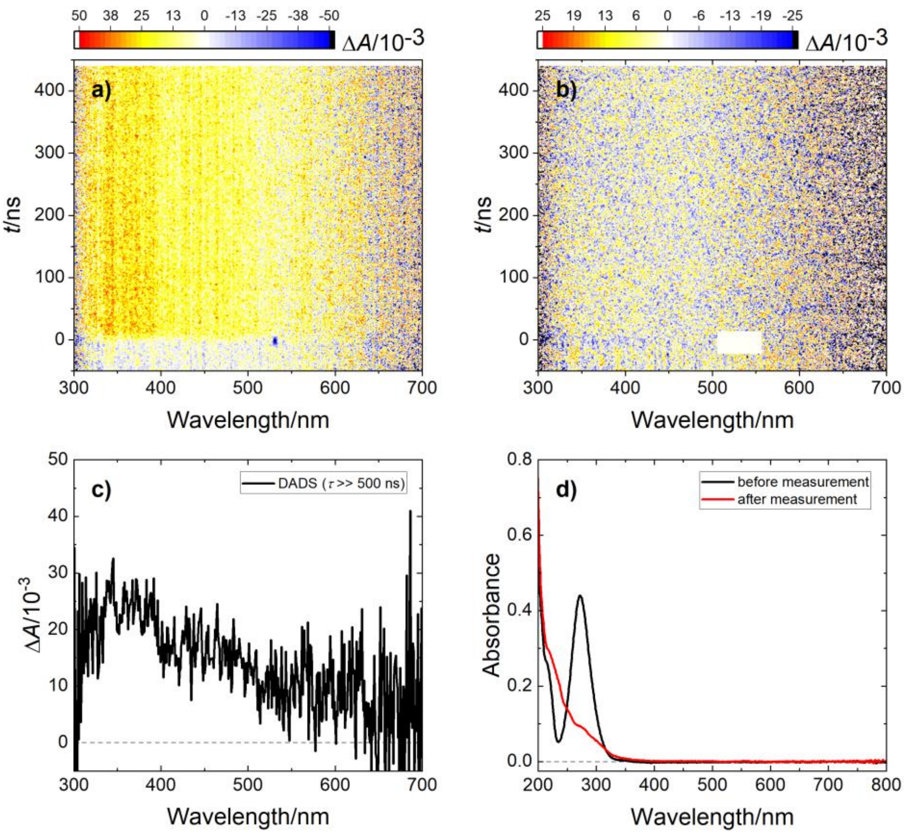

Figure S9: **a)** Transient Absorption data of **1a** in ACN on a 500 ns timescale reveals a broad absorption forming immediately after photoexcitation. **b)** The data can be globally fitted using a single exponential, leading to clean residues. Scattering light of the pump beam at 532 nm is patched from the data (white rectangle). The width of the IRF was fixed to 15 ns. **c)** The DADS obtained from the fit, shows a broad absorption lasting much longer than 500 ns. **d)** During the measurement strong photodegradation of the sample occurs.

Similar experiments on a 10 µs timescale revealed that this signal decays with a lifetime of 1.2 µs, forming new species with absorption maxima at 315 nm and 450 nm respectively (**Figure S10**). These species outlast the 10 µs timeframe.

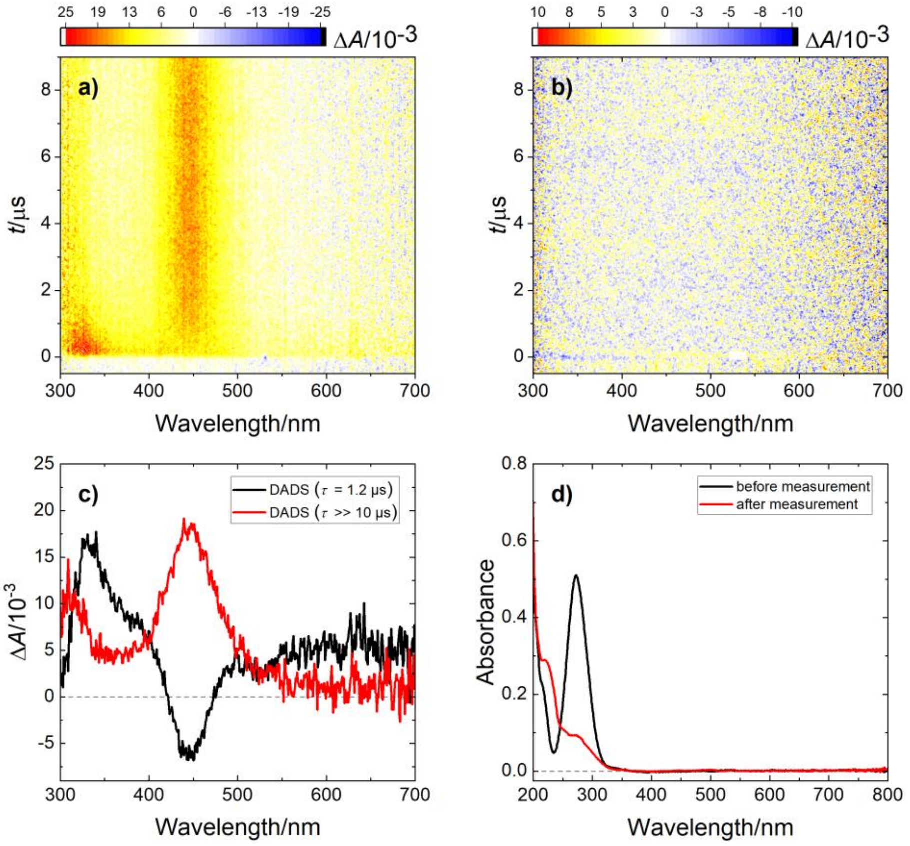

Figure S10: **a)** Transient Absorption data of **1a** in ACN on a 10 µs timescale shows how the species observed on shorter timeframes decays while forming two new absorption bands. **b)** Two exponentials are needed to describe the data with clean residues. Scattering light of the pump beam at 532 nm is patched from the data (white rectangle). **c)** DADS from the fit nicely show how new species are formed at 315 and 450 nm, as described by the DADS with 1.2 µs lifetime. A second DADS with a lifetime far exceeding the timeframe describes these new signals persisting far over 10 µs. **d)** During the measurement strong photodegradation of the sample occurs.

On even longer timescales, one can observe how the signal at 450 nm decays with a lifetime of 166 µs whereas an absorption remains at the short wavelength edge of the measurement window, with a decay constant of 1.3 ms (**Figure S11**).

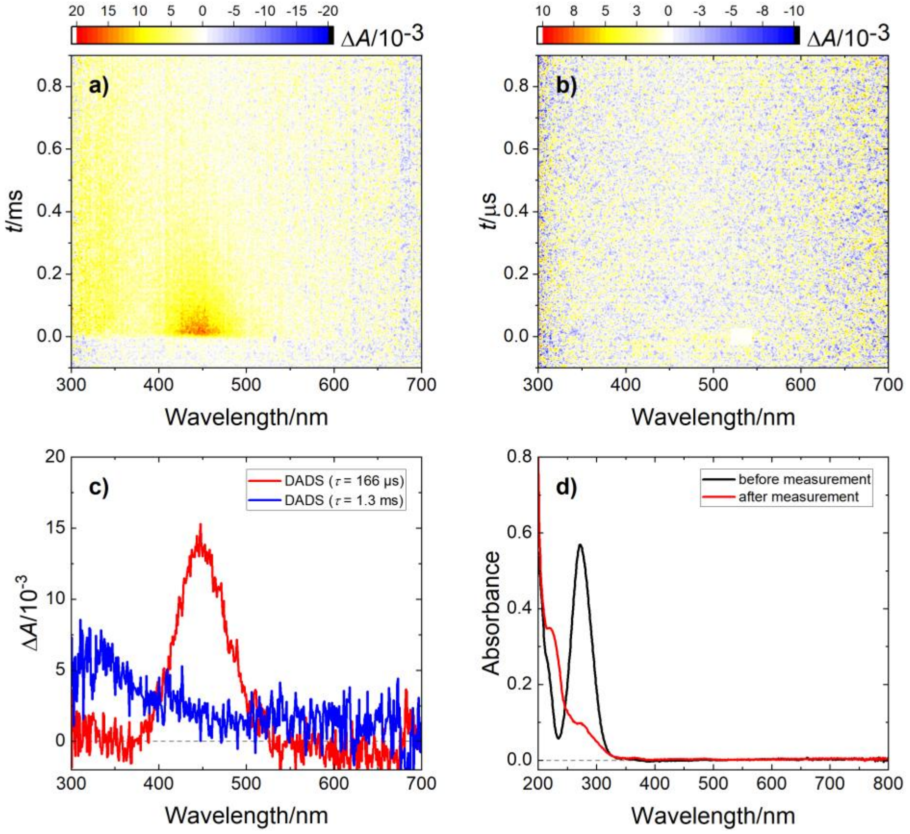

Figure S11*:* **a)** Transient Absorption data of **1a** in ACN on a 1 ms timescale reveals decay of the species absorbing at 450 nm, while the signal at 315 nm remains longer. **b)** Two exponentials are needed to describe the data with clean residues. Scattering light of the pump beam at 532 nm is patched from the data (white rectangle). **c)** DADS show the decay of the two respective species with lifetimes of 166 µs and 1.3 ms respectively. **d)** During the measurement strong photodegradation of the sample occurs.

The spectral features obtained from these TA datasets are reminiscent of species related to the structurally similar **BP**. The broad, short-lived absorption band retains similar spectral features to **BP**'s triplet-triplet absorption, whereas the species with a ms-lifetime resembles a blue-shifted version of the **BP** ketyl radical (**Figure S12**).[38]

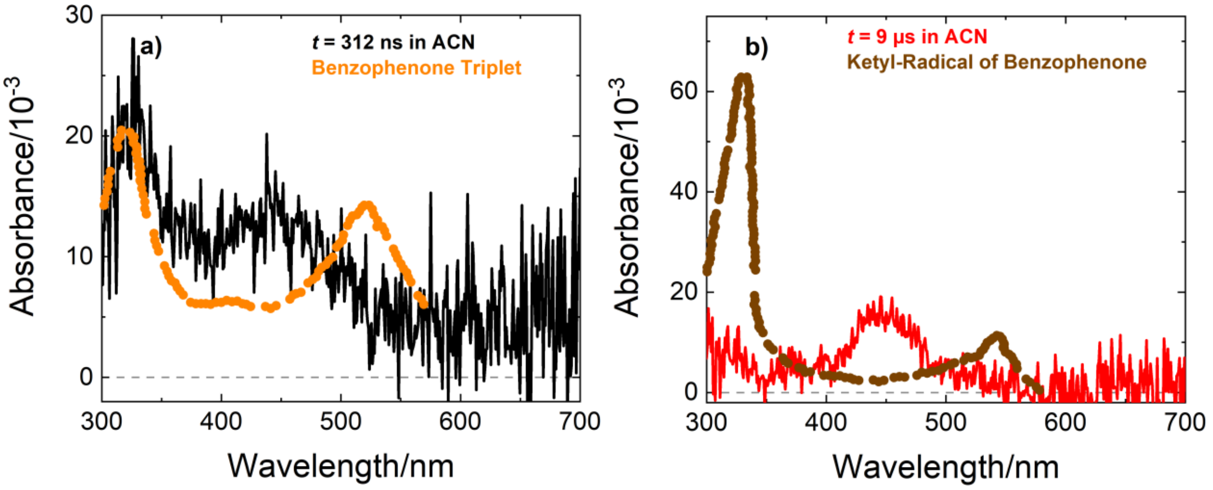

Figure S12: **a)** The signal obtained shortly after photoexcitation of **1a** looks similar to a blue-shifted version of the triplet state of **BP. b)** On a µs-timescale another signal is formed, appearing like a blue-shifted spectrum of the ketyl-radical of **BP**. The two reference spectra are adapted from Reference 38**.**

Hence, the processes depicted in **Scheme S1** appear most plausible for an interpretation of these signals. Photoexcitation of **1a** leads to formation of the triplet state, which is then able to perform HAT, forming the ketyl-radical.

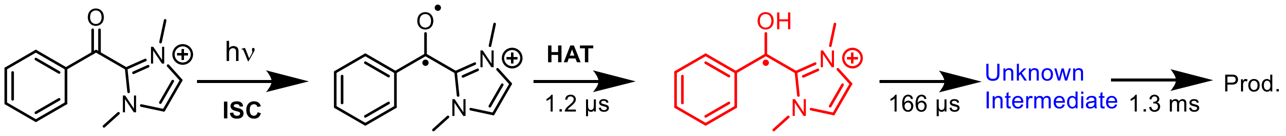

Scheme S1: a) After photoexcitation of **1a** the triplet state **1a(T_1)** is formed and has a lifetime of 1.2 µs, partially decaying *via* HAT and thus forming the ketyl radical absorbing at 450 nm. This radical then decays with a lifetime of 166 µs towards an unknown intermediate absorbing around 300 nm, which itself decays with a lifetime of 1.3 ms.

As for possible HAT-donors, the most likely candidate appears to be residual water in the solution, since the barrier for HAT from ACN is rather high (11.6 kcal/mol).[3] Possible pathways for the degradation of the ketyl radical are addition of the geminate hydroxyl radical, reduction towards the alcohol, or dimerization, analogous to **BP**[39], although this appears less likely due to Coulombic repulsion which would have to be overcome.

The hypsochromy may be explained by the positive charge of **1a** and its intermediates, since it is known that reduction of the ketyl radical of **BP** to the radical anion results in the opposite effect, namely a bathochromic shift.[40] While these deliberations are an interesting subject for future studies, they are beyond the scope of this work. Instead, we aimed at investigating these species in different solvents, namely DCM and THF.

Transient absorption of **1a** in DCM shows a very similar behavior as in ACN, although lifetimes differ significantly. On short timescales, one can once again observe the absorption of **1a(T_1)** (**Figure S13**).

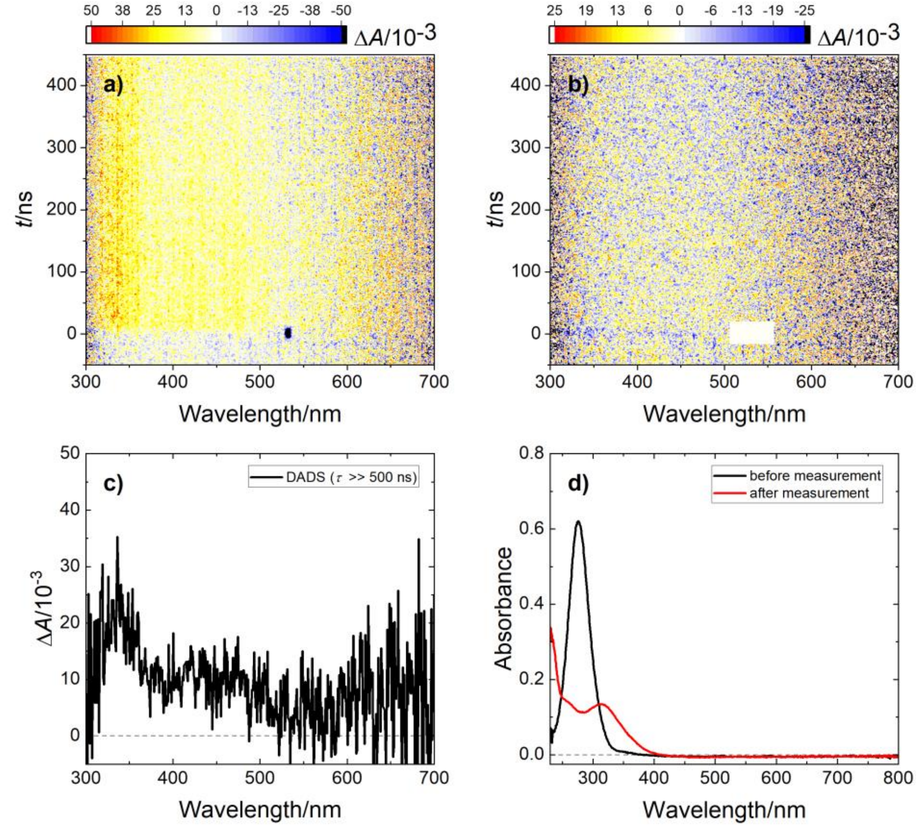

Figure S13*:* **a)** Transient Absorption data of **1a** in DCM on a 500 ns timescale shows very similar signals to the data in ACN (**Figure S9**). **b)** One exponential is needed to describe the data with clean residues. Scattering light of the pump beam at 532 nm is patched from the data (white rectangle). The width of the IRF was fixed to 15 ns. **c)** The DADS shows the spectrum of **1a(T$_1$)** outlasting the covered time window. **d)** During the measurement, strong photodegradation of the sample occurs, however in contrast to the data in ACN a pronounced absorption band arises around 350 nm.

While the behavior is very similar to ACN, for DCM there are differences in the spectrum after the measurement. A pronounced absorption band arises around 350 nm, which may belong to the unknown intermediate already observed with a 1.3 ms lifetime in ACN.

On the 10 µs timescale, **1a(T$_1$)** again converts to the ketyl radical, however the lifetime is reduced to 423 ns, and the process is less pronounced, indicating a more efficient alternative relaxation pathway (**Figure S14**).

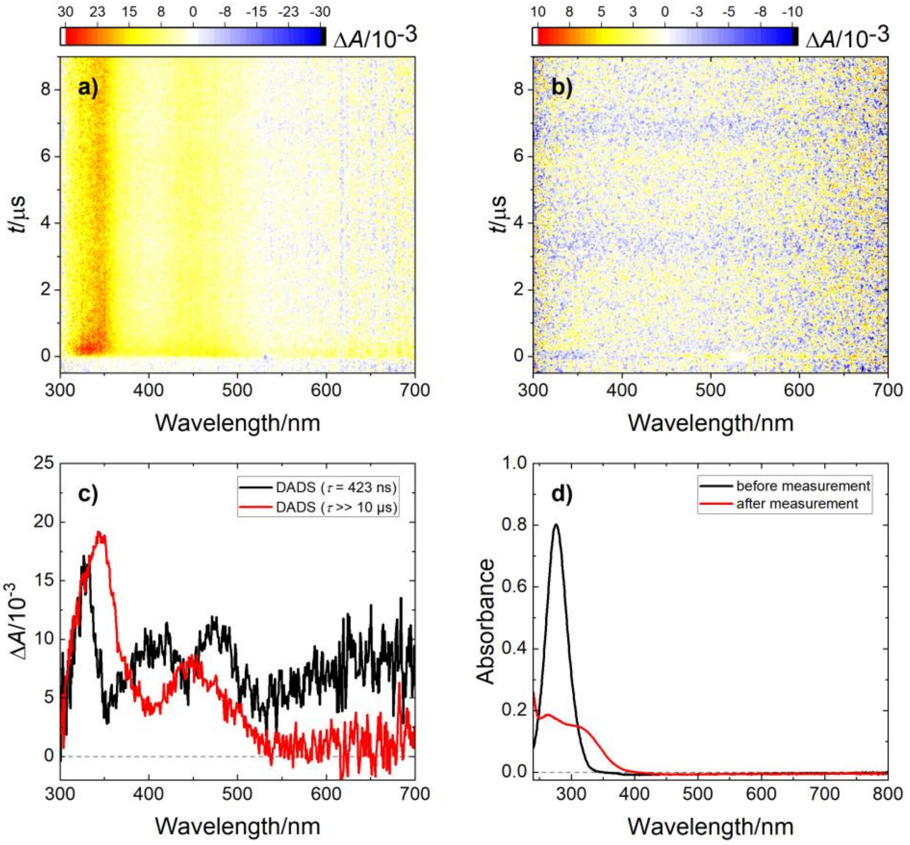

Figure S14*:* **a)** Transient Absorption of **1a** in DCM on a 10 µs timescale again reveals the HAT forming the ketyl radical from **1a(T$_1$)**, although with a shorter lifetime and less pronounced. **b)** Two exponentials are needed to describe the data with clean residues. Scattering light of the pump beam at 532 nm is patched from the data (white rectangle). **c)** The DADS with a 423 ns lifetime once again shows a dip at 450 nm, where the ketyl radical emerges. The second DADS describes the decay of the ketyl radical with a lifetime far exceeding 10 µs. **d)** During the measurement, strong photodegradation of the sample occurs and a new absorption band arises around 350 nm.

On even longer timescales, one can once again observe the decay of the ketyl radical with a lifetime of 210 µs. Other spectral components remain for more than 1 ms, the component at 350 nm can even be observed minutes after excitation. The exact nature of these products remains to be elucidated but is not part of this study.

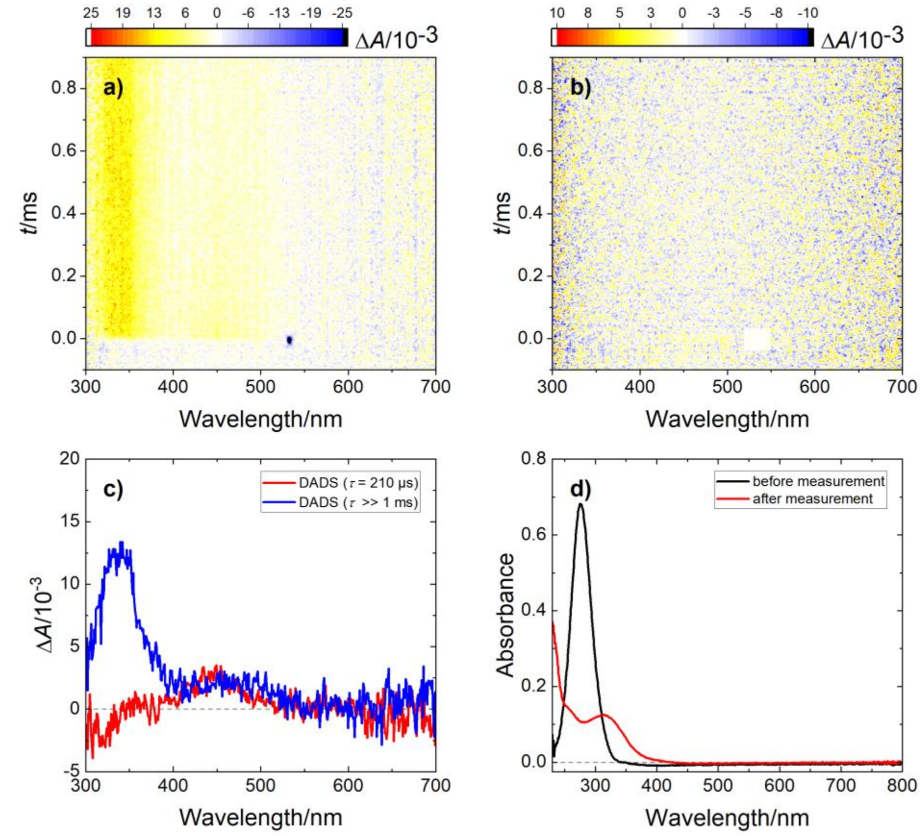

Figure S15*:* a) Transient Absorption of **1a** in DCM on a 1 ms timescale reveals a shorter-lived component as well as several long-lived signals. **b)** Two exponentials are needed to describe the data with clean residues. Scattering light of the pump beam at 532 nm is patched from the data (white rectangle). **c)** A DADS with a 210 µs lifetime possibly describes the decay of the ketyl radical, while a long-lived DADS consists of the spectral components of possible products. **d)** During the measurement, strong photodegradation of the sample occurs and a new absorption band arises around 350 nm, which matches the long-lived component at this wavelength.

Since the broad absorption band with a lifetime in the ns-µs regime is assigned to the triplet state **1a(T$_1$)** and transforms *via* HAT towards the ketyl radical, lasting for hundreds of µs, addition of a good hydrogen-atom donor like THF should lead to a drastic reduction in the triplet lifetime and very fast formation of the ketyl radical. Therefore, the TA measurements were repeated using THF as the solvent. Indeed, on a 500 ns timescale, no signal of **1a(T$_1$)** is observable, and the spectral features of the ketyl radical are immediately present upon excitation of **1a** (**Figure S16**). One can also observe very strong photodegradation and the formation of a new signal around 350 nm.

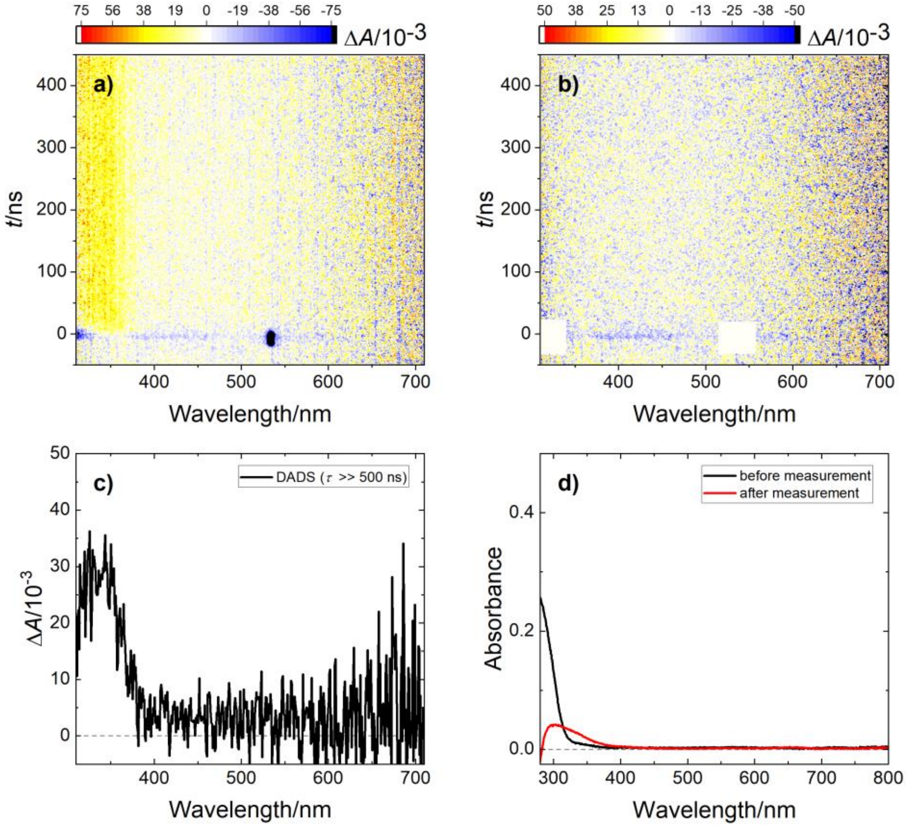

Figure S16*:* **a)** Transient Absorption of **1a** in THF shows no signals associated with **1a(T_1)**. Instead, the spectral features of the ketyl radical are present immediately. **b)** A single exponential is needed to describe the data. The negative signals in the residues stem from emission of THF, which weakly absorbs at 266 nm. Scattering light of the pump beam is patched from the data (white rectangles). The width of the IRF was fixed to 15 ns. **c)** A DADS with lifetime exceeding 500 ns describes the decay of the ketyl radical. **d)** During the measurement, strong photodegradation of the sample occurs and a new absorption band arises around 350 nm.

Due to a better signal-to-noise ratio, the ketyl signal is more pronounced on a 10 µs timescale (**Figure S17**).

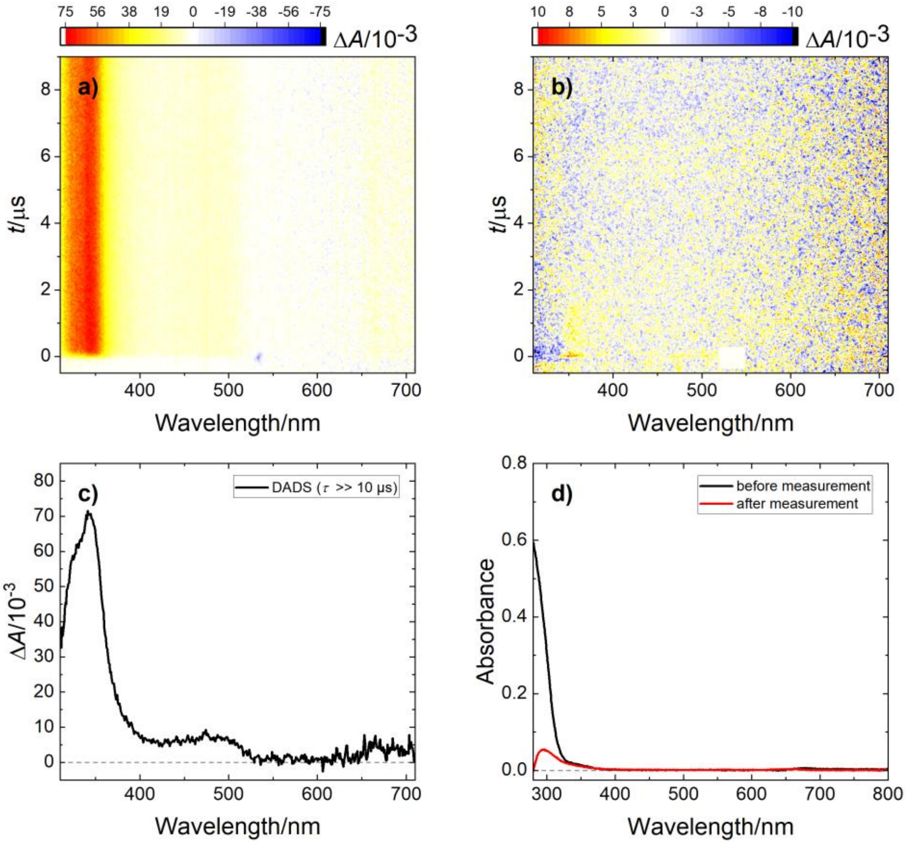

Figure S17*:* **a)** Transient Absorption of **1a** in THF shows the signal of the ketyl radical outlasting the measurement window. **b)** A single exponential is needed to describe the data. Scattering light of the pump beam is patched from the data (white rectangle). **c)** A DADS with lifetime exceeding 10 µs describes the decay of the ketyl radical. **d)** During the measurement, strong photodegradation of the sample occurs.

These findings corroborate the assignment of the short-lived signal from the measurements in DCM and ACN as the triplet state **1a(T$_1$)** as well as the signal emerging from it as the ketyl radical. Since in THF the solvent itself acts as the hydrogen-atom donor, one would expect to observe coupling between **1a** and THF (**Scheme S2**). Indeed, this is the case, since after deprotonation the NHC group eliminates and the expected ketone can be isolated in 28 % yield.

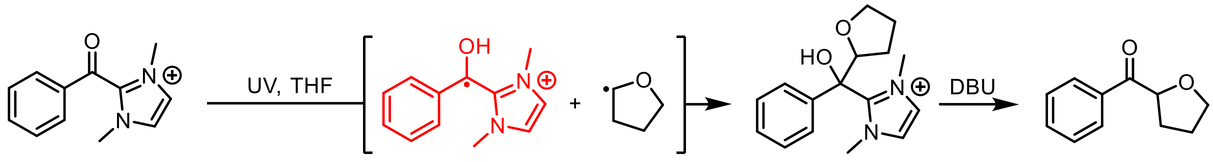

1a **Ketyl radical** **2**, 28%

Scheme S2: Formation of the protonated ketyl radical after photoexcitation of **1a** and HAT event, followed by the photochemical synthesis of phenyl(tetrahydrofuran-2-yl)methanone **2**.

Hence, we can confirm that **1a** is able to perform HAT from its triplet state in a similar manner to **BP**, with the lifetime of **1a(T$_1$)** lying in the ns-µs regime depending on the solvent, whereas the emerging ketyl radical persists into the 100 µs regime.

7. NMR spectroscopic data

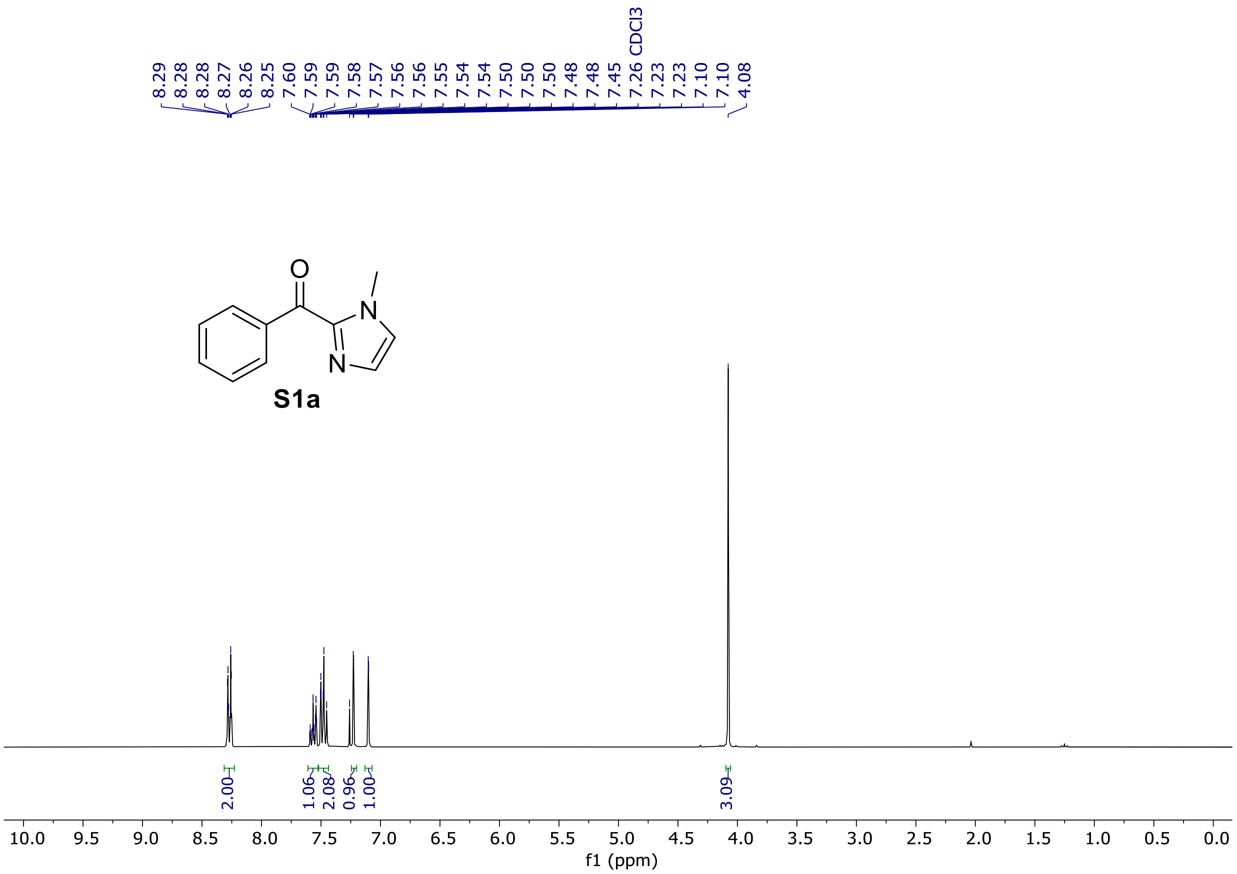

Figure S18: ^{1}H NMR spectrum of **S1a** in $CDCl_3$ (300 MHz)

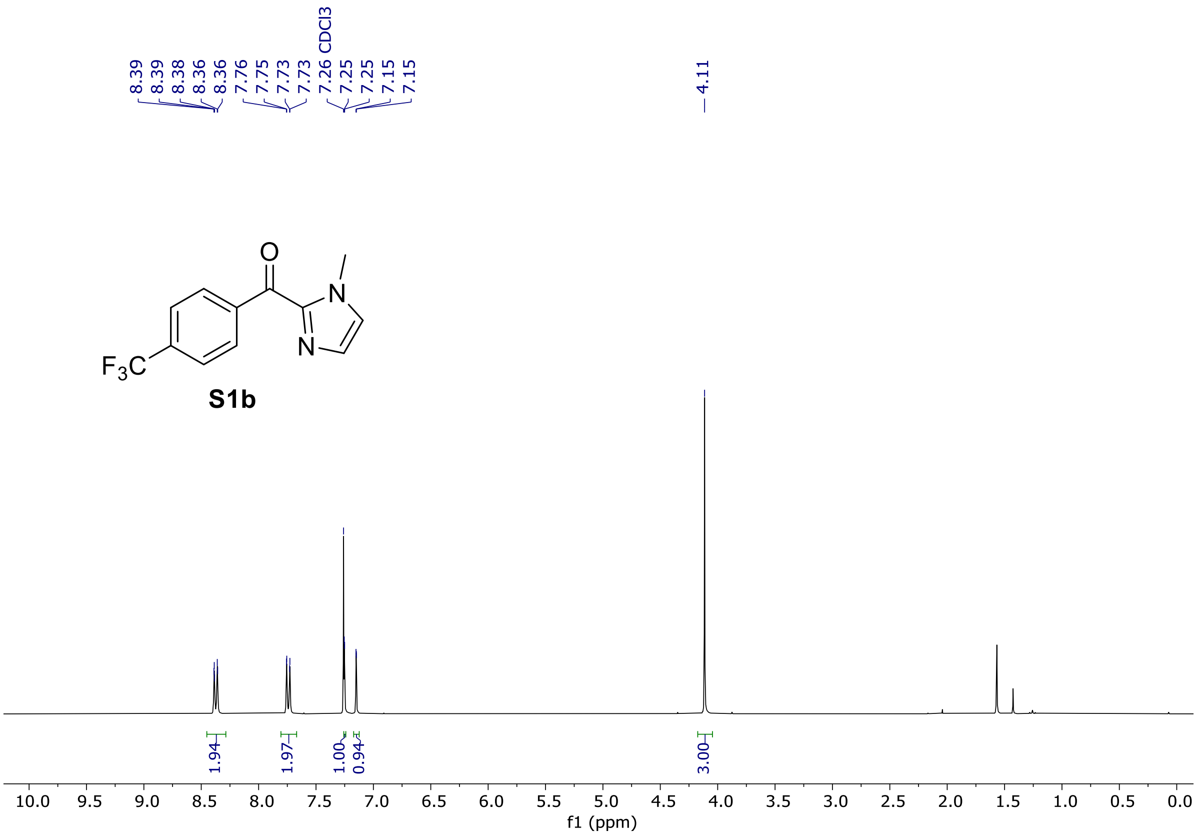

Figure S19: ^{1}H NMR spectrum of **S1b** in $CDCl_3$ (300 MHz)

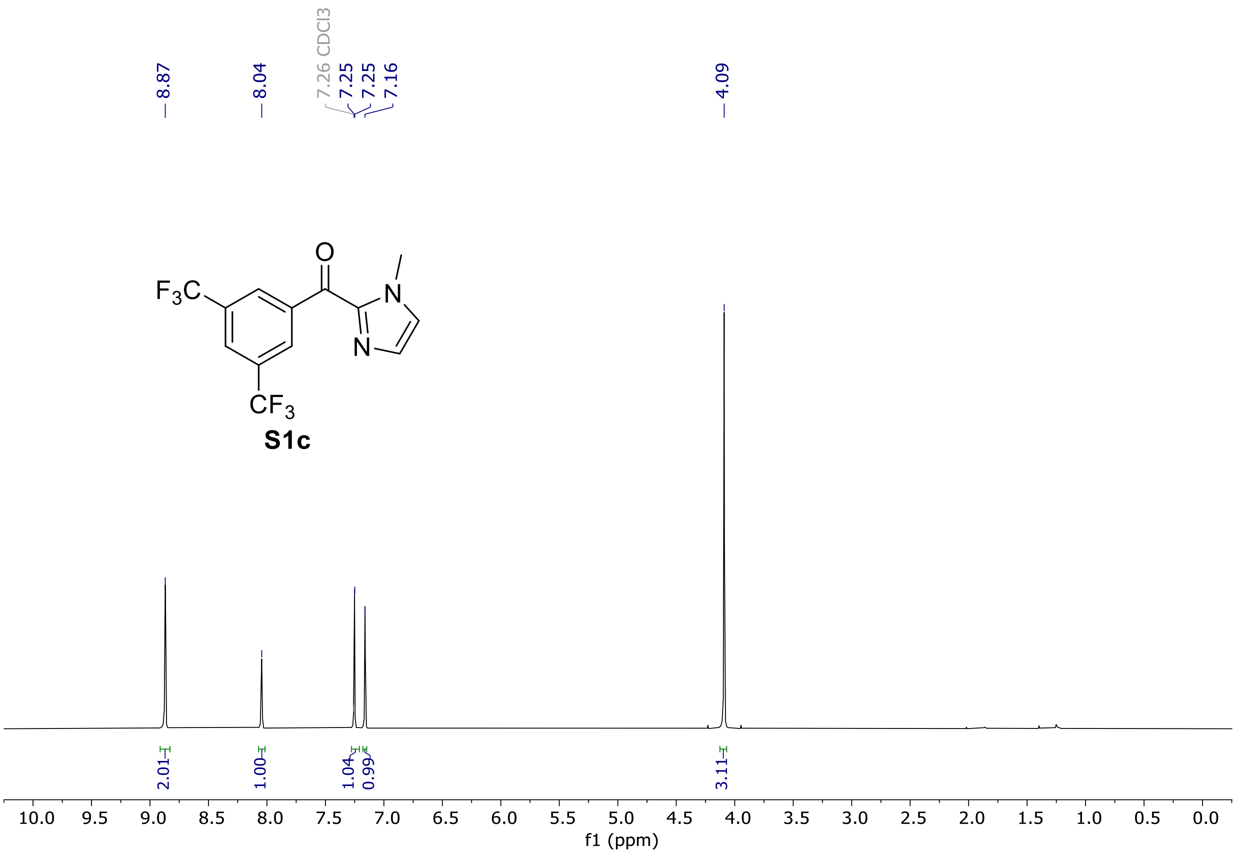

Figure S20: ^{1}H NMR spectrum of **S1c** in $CDCl_3$ (500 MHz)

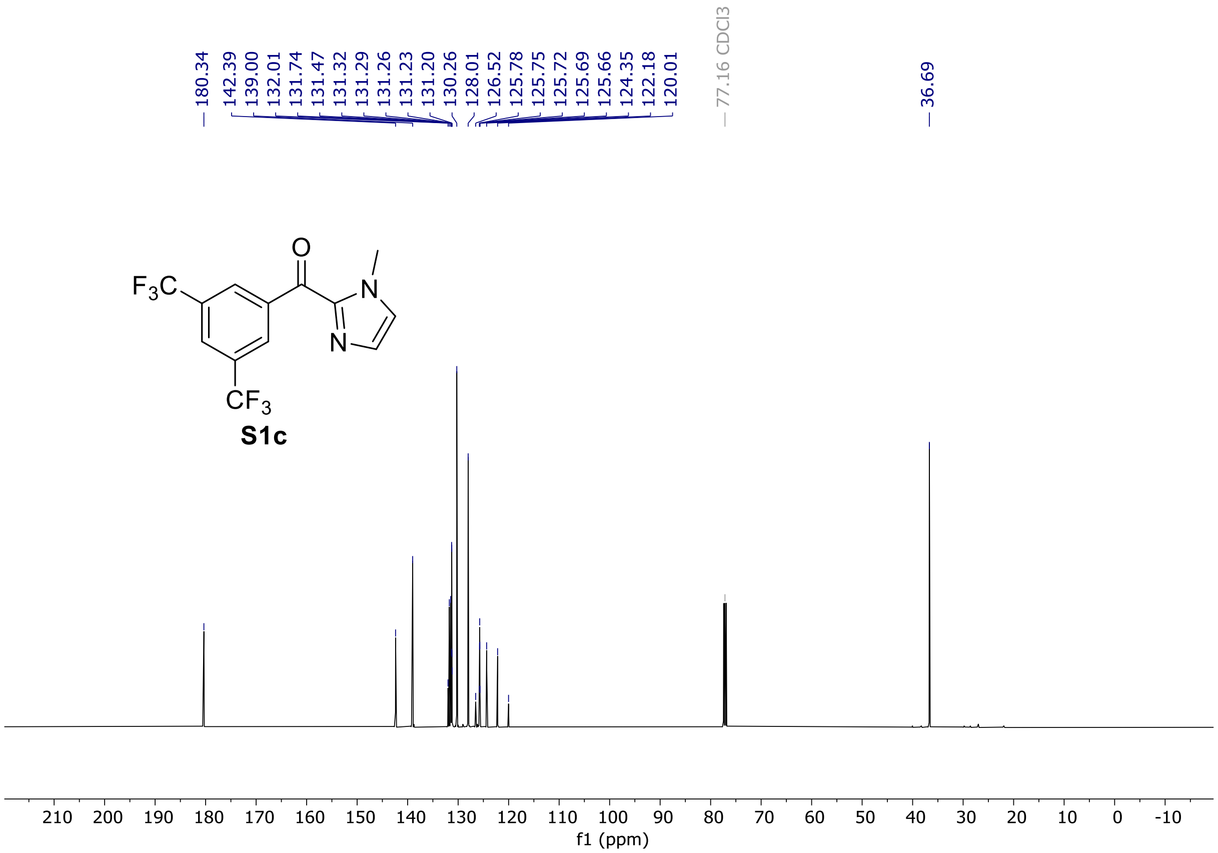

Figure S21: $^{13}C\{^{1}H\}$ NMR spectrum of **S1c** in $CDCl_3$ (125 MHz)

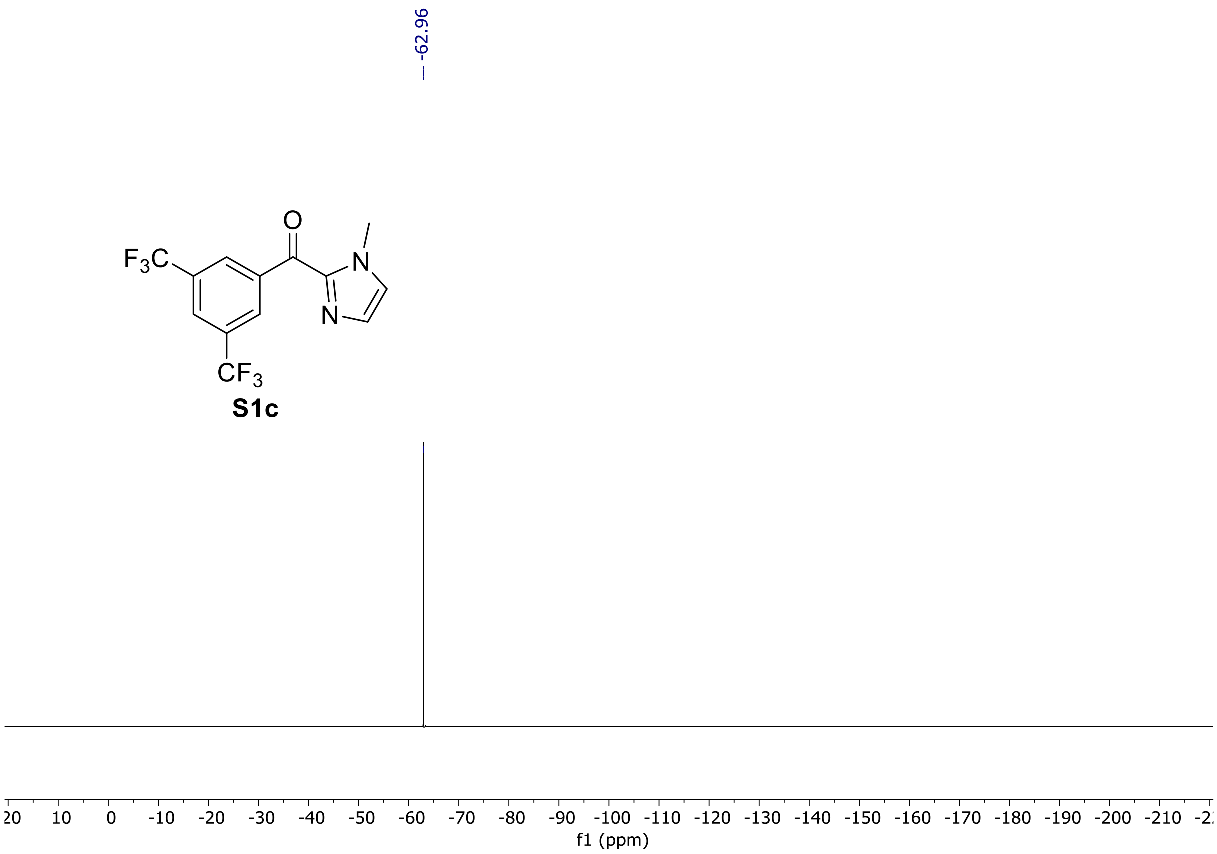

Figure S22: $^{19}F\{^1H\}$ NMR spectrum of **S1c** in $CDCl_3$ (282 MHz)

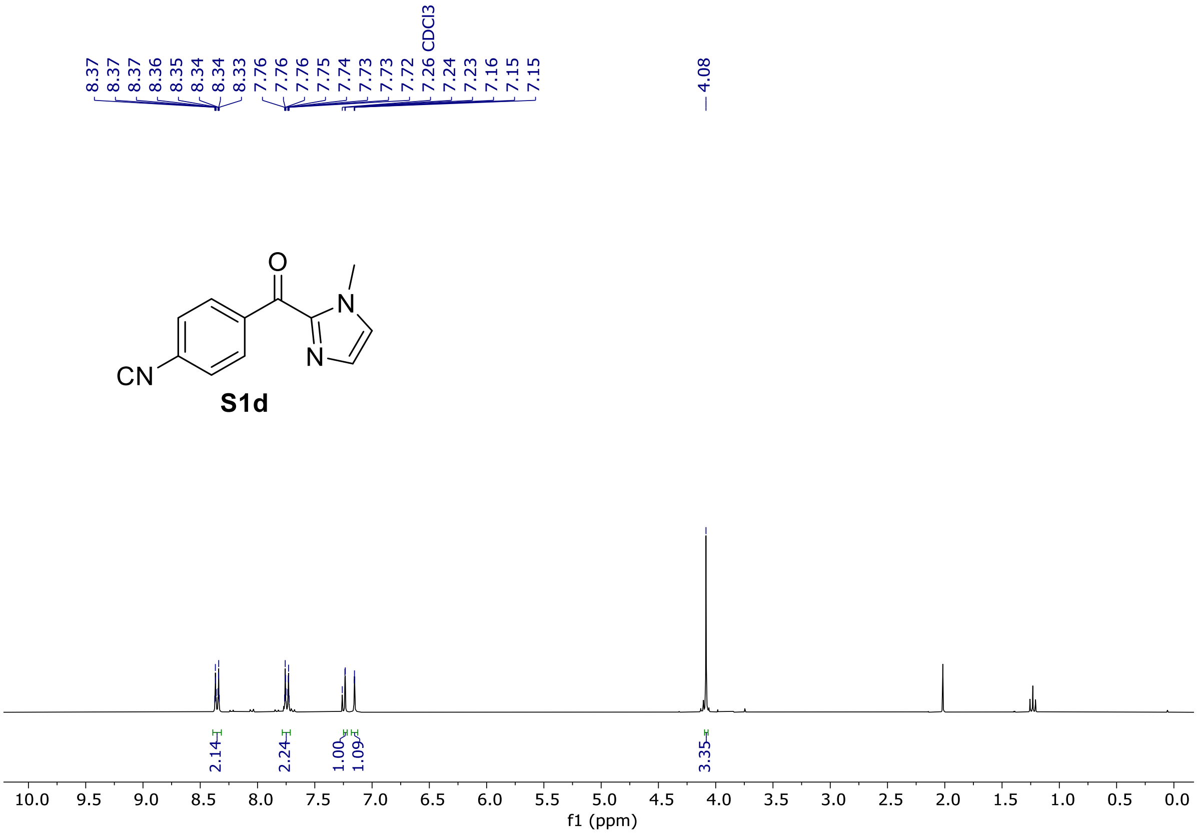

Figure S23: ^1H NMR spectrum of **S1d** in $CDCl_3$ (300 MHz)

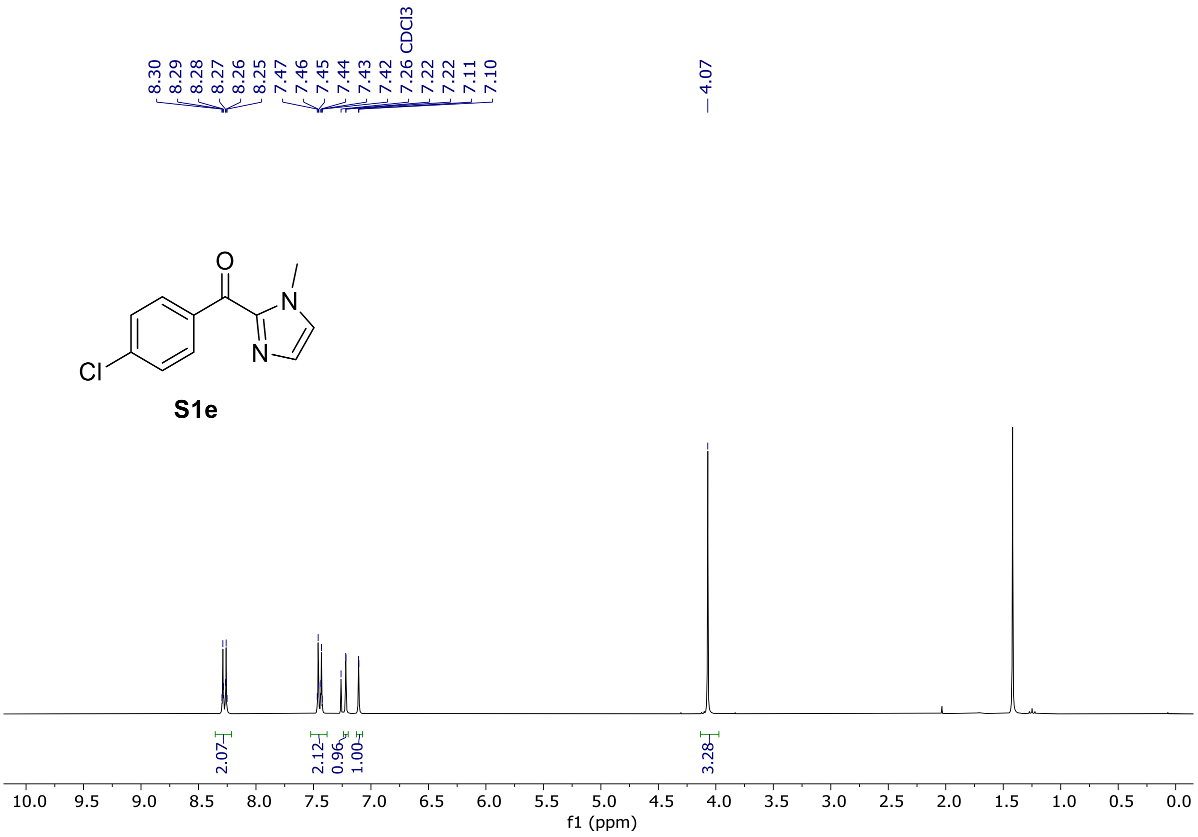

Figure S24: ^{1}H NMR spectrum of **S1e** in $CDCl_3$ (300 MHz)

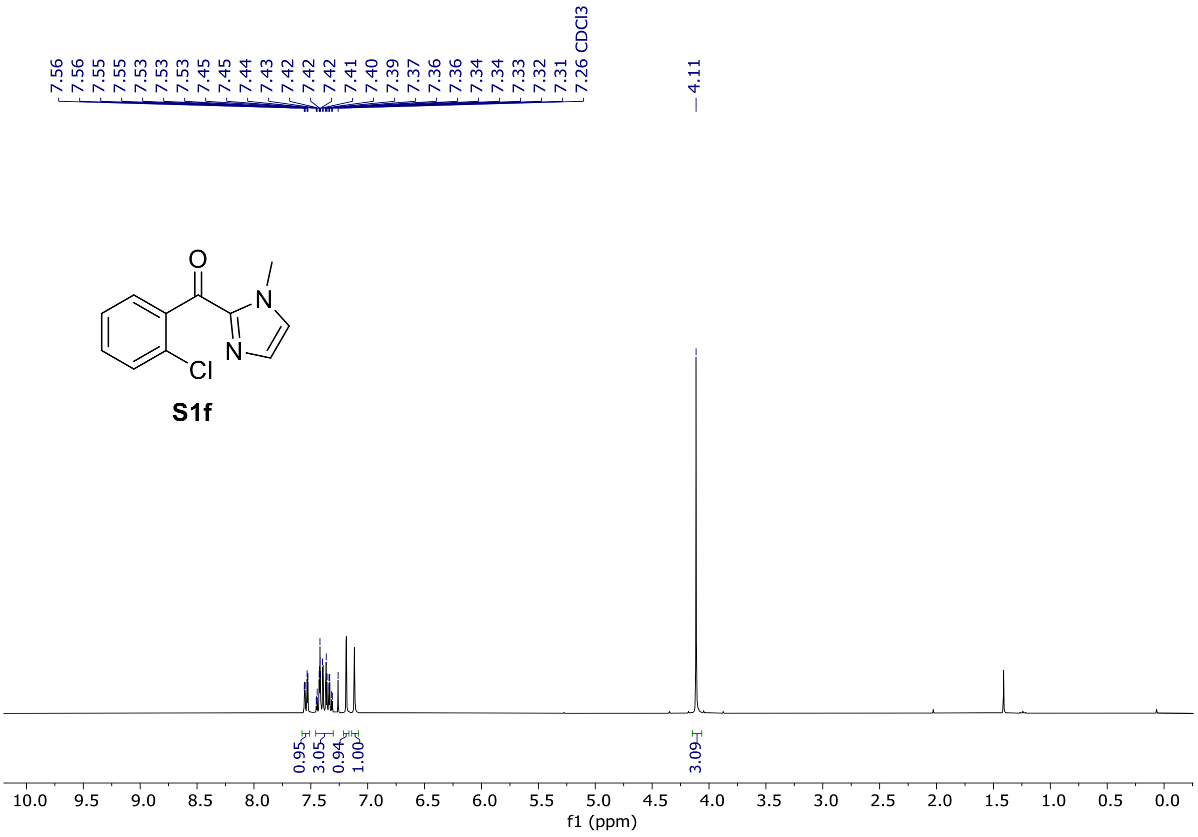

Figure S25: ^{1}H NMR spectrum of **S1f** in $CDCl_3$ (300 MHz)

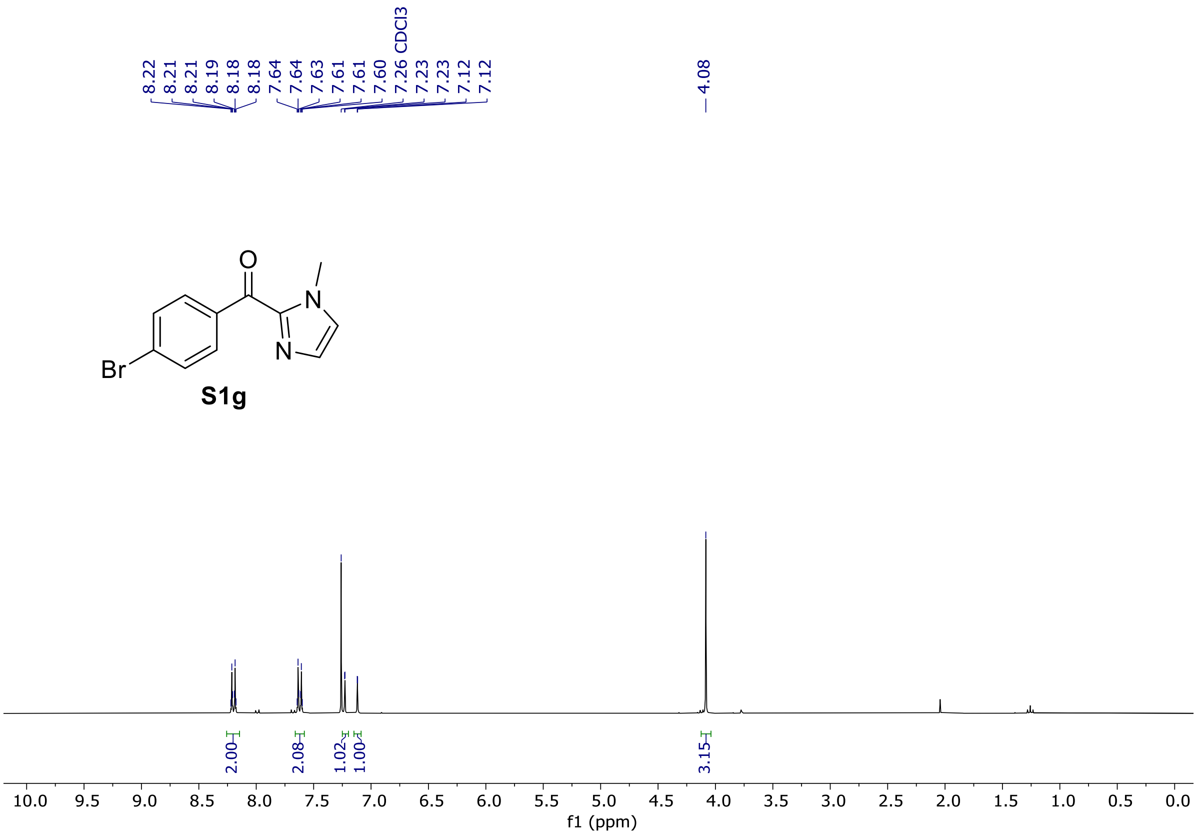

Figure S26: ^{1}H NMR spectrum of **S1g** in $CDCl_3$ (300 MHz)

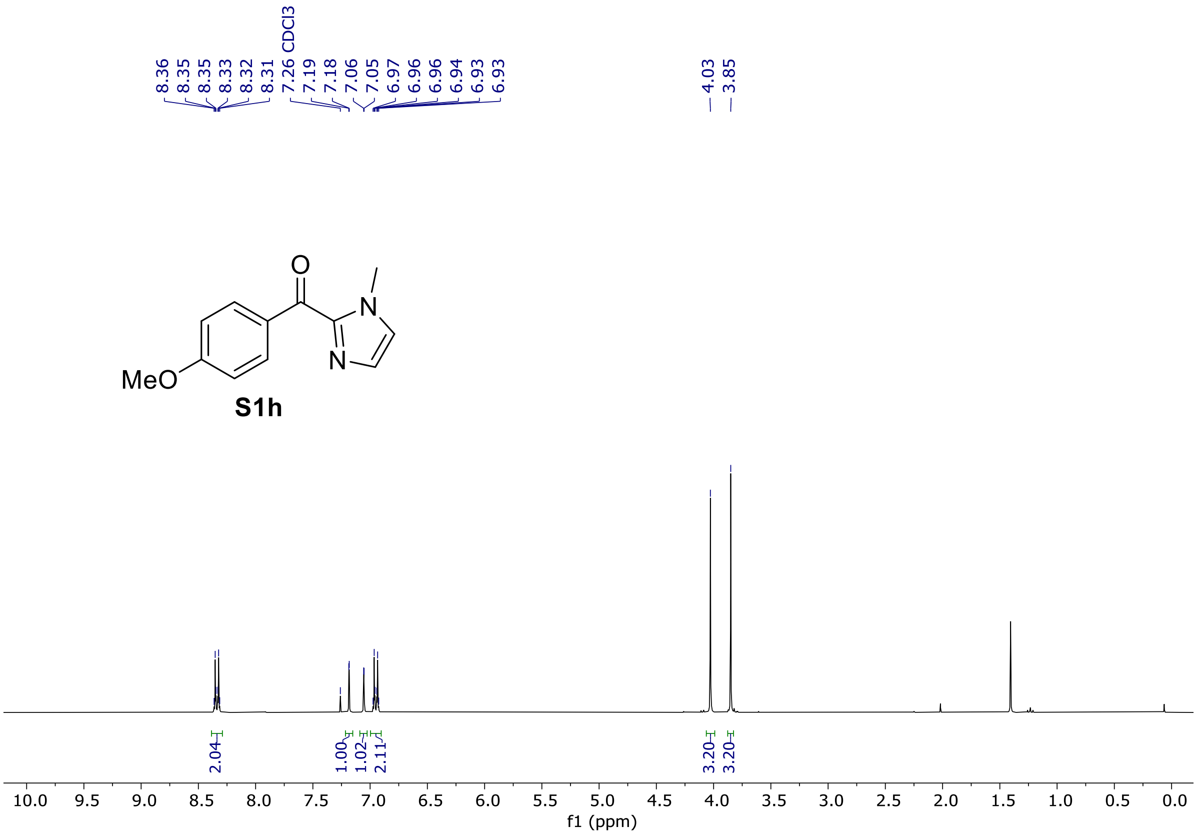

Figure S27: ^{1}H NMR spectrum of **S1h** in $CDCl_3$ (300 MHz)

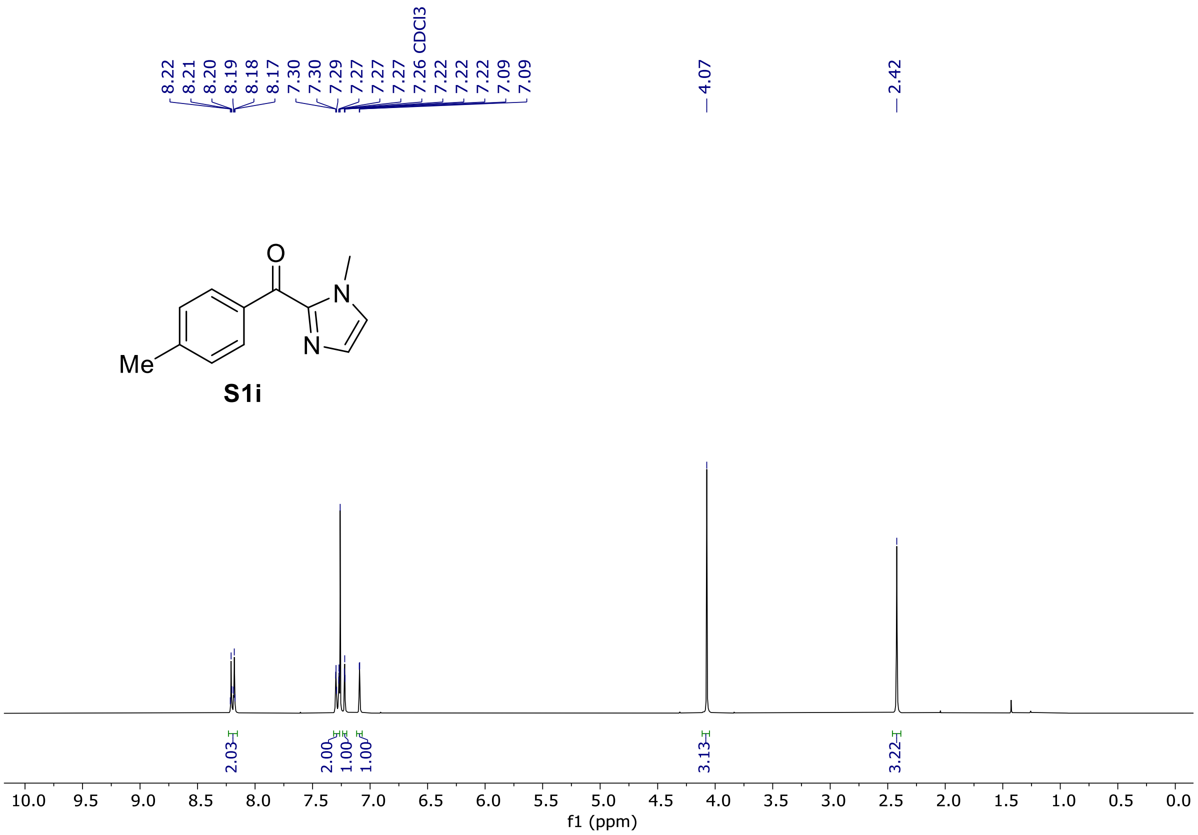

Figure S28: ^{1}H NMR spectrum of **S1i** in $CDCl_3$ (300 MHz)

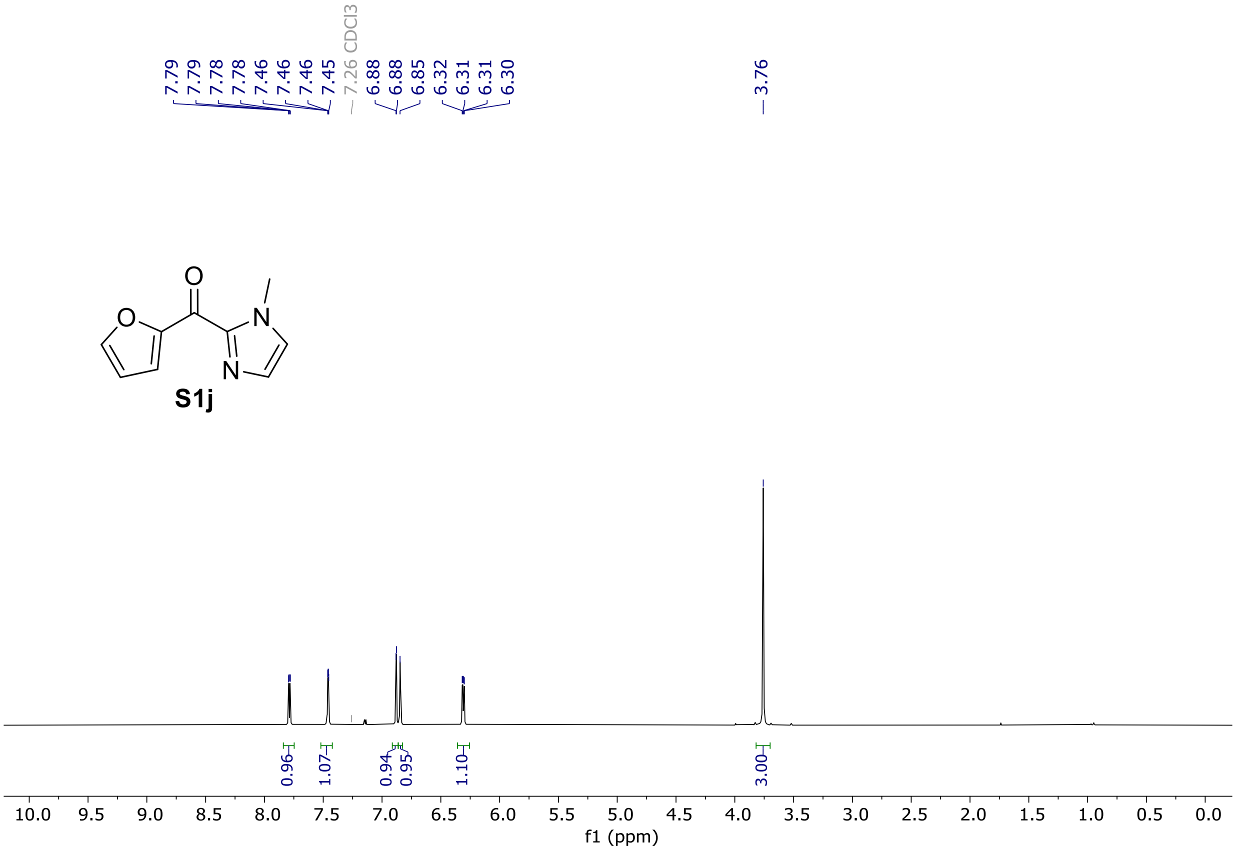

Figure S29: ^{1}H NMR spectrum of **S1j** in $CDCl_3$ (300 MHz)

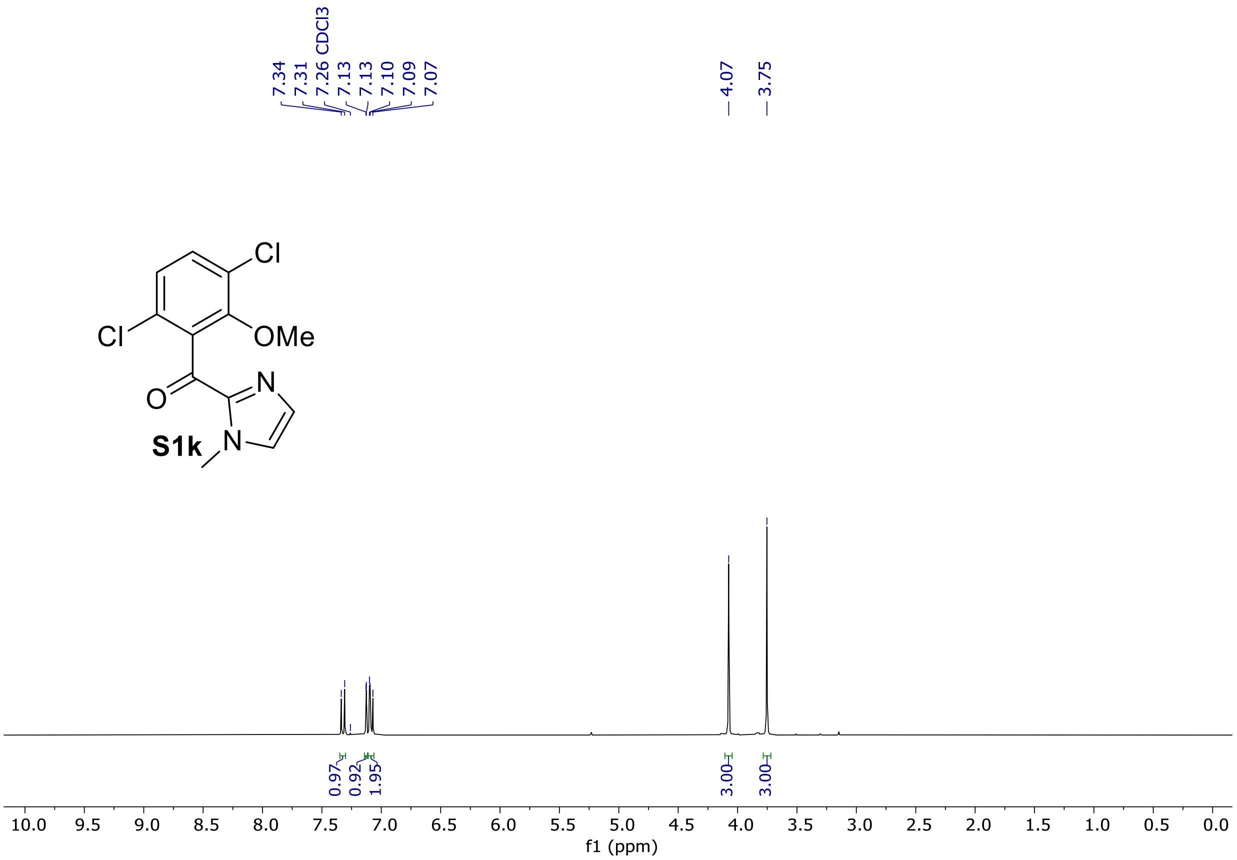

Figure S30: ^{1}H NMR spectrum of **S1k** in $CDCl_3$ (300 MHz)

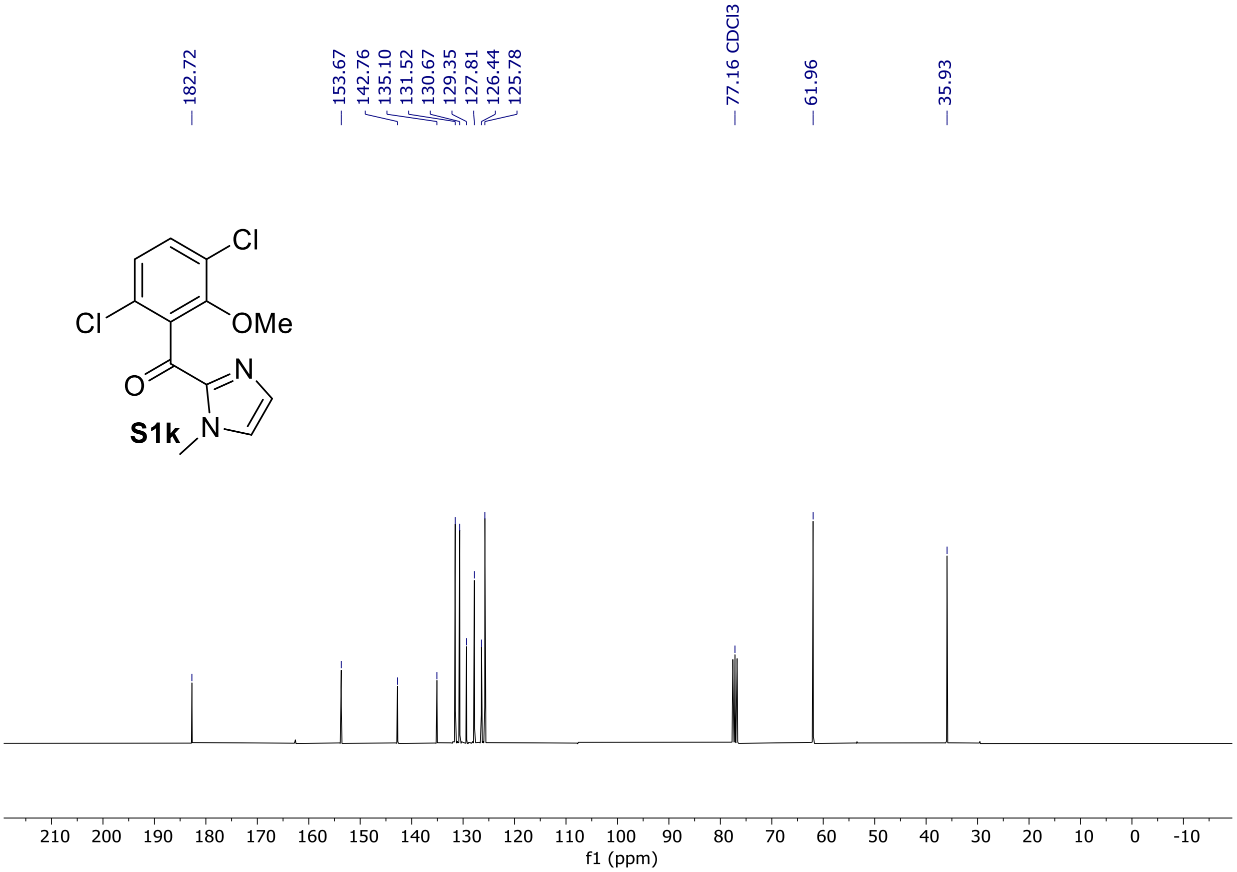

Figure S31: $^{13}C\{^{1}H\}$ NMR spectrum of **S1k** in $CDCl_3$ (75 MHz)

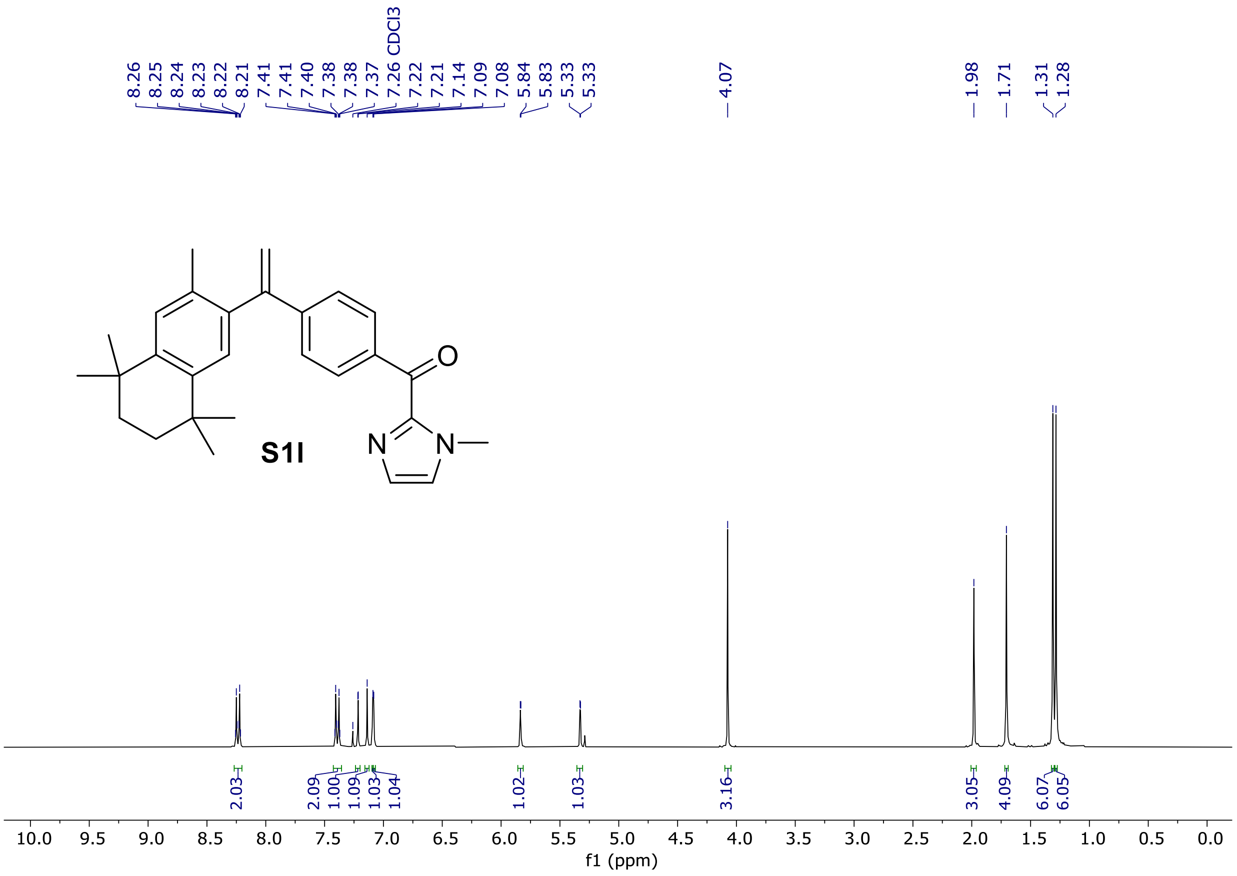

Figure S32: ^{1}H NMR spectrum of **S1l** in $CDCl_3$ (300 MHz)

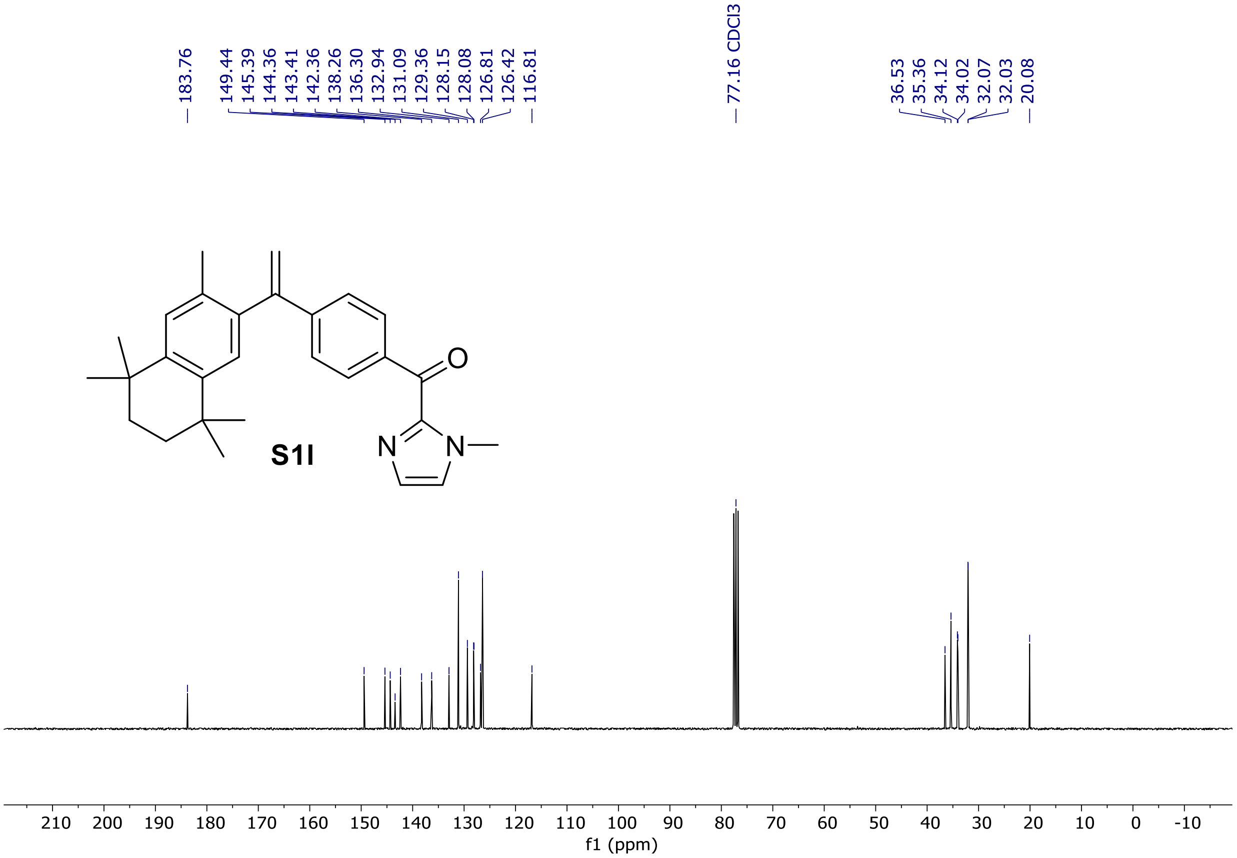

Figure S33: $^{13}C\{^{1}H\}$ NMR spectrum of **S1l** in $CDCl_3$ (75 MHz)

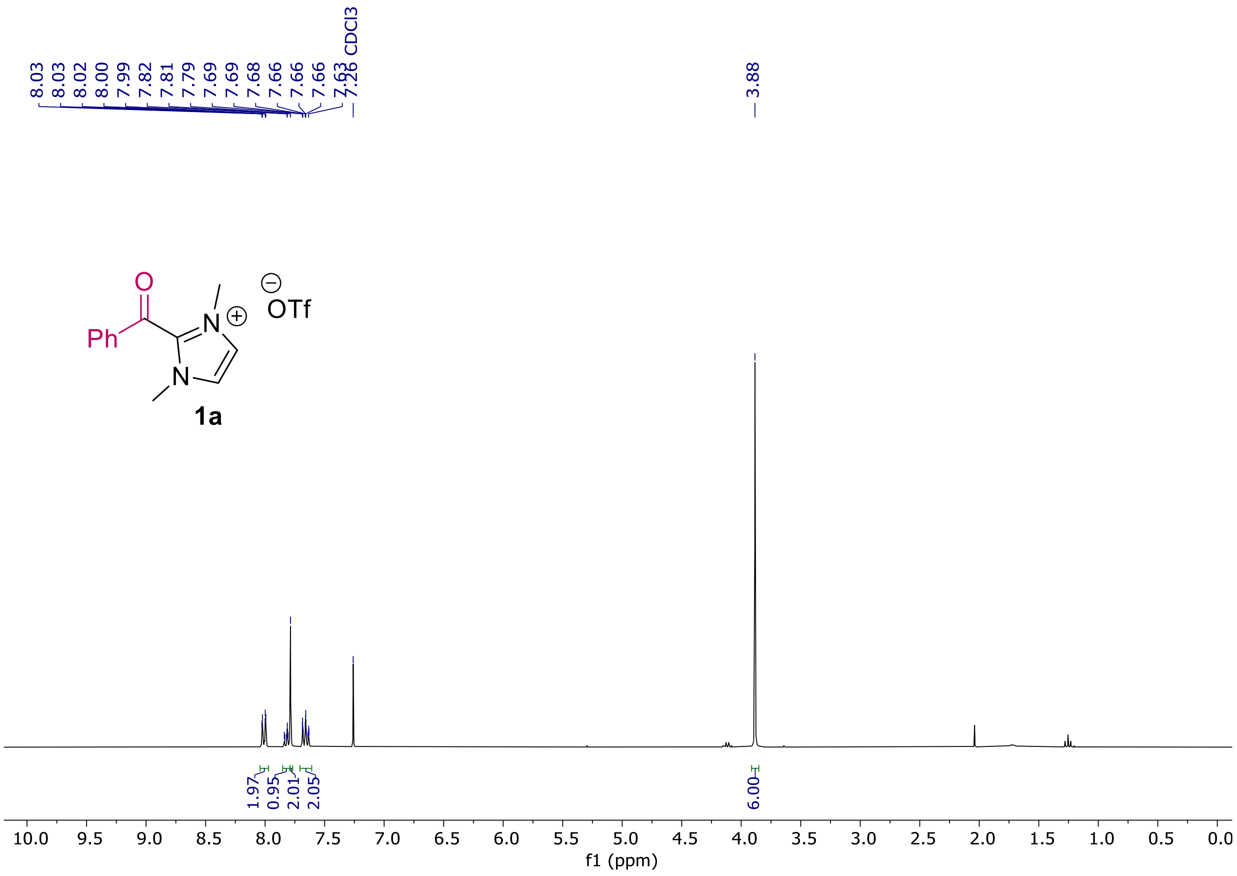

Figure S34: ^{1}H NMR spectrum of **1a** in $CDCl_3$ (300 MHz)

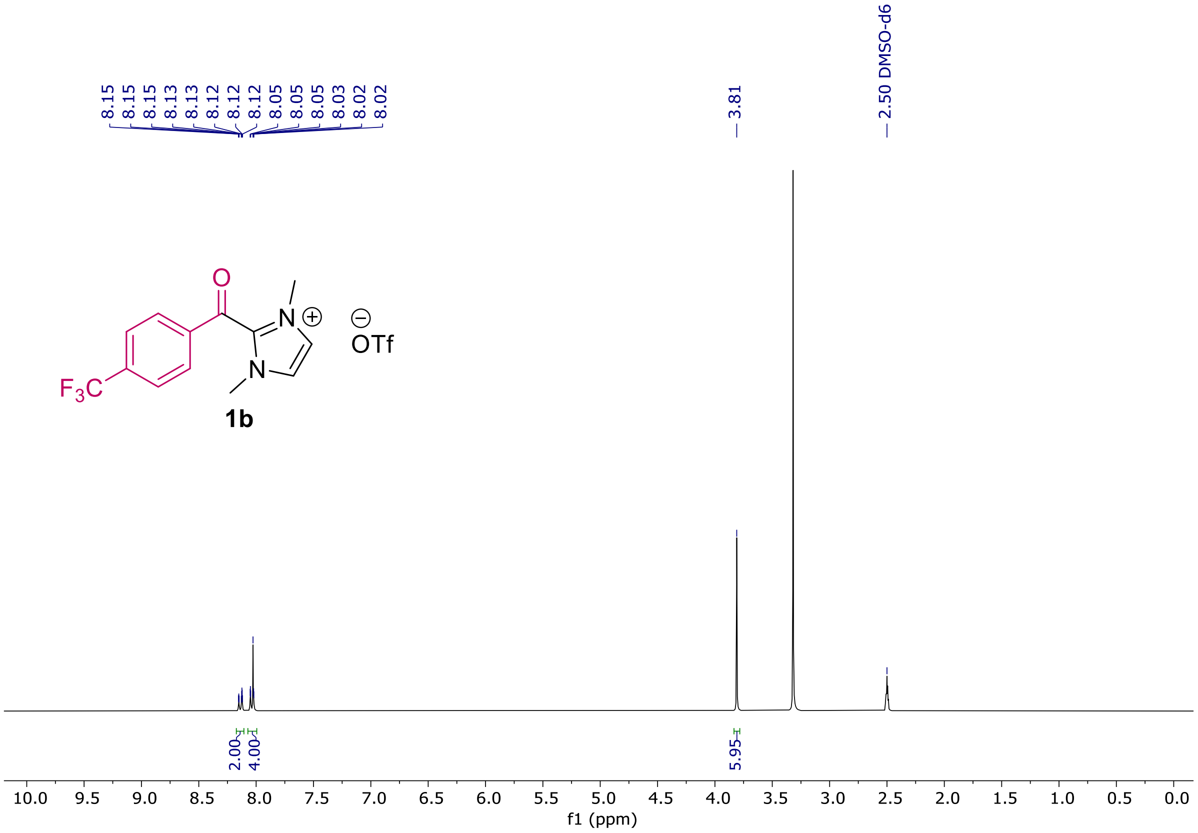

Figure S35: ^{1}H NMR spectrum of **1b** in DMSO-d6 (300 MHz)

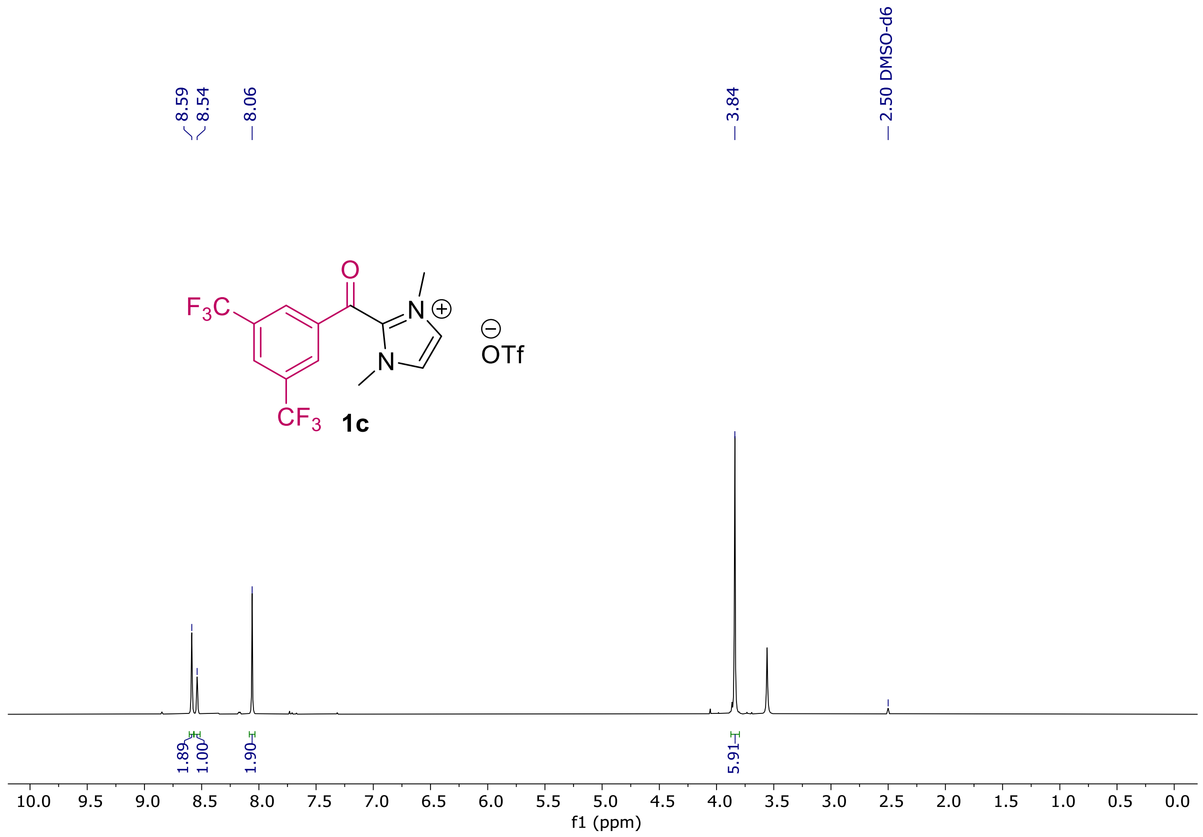

Figure S36: ^{1}H NMR spectrum of **1c** in DMSO-d6 (500 MHz)

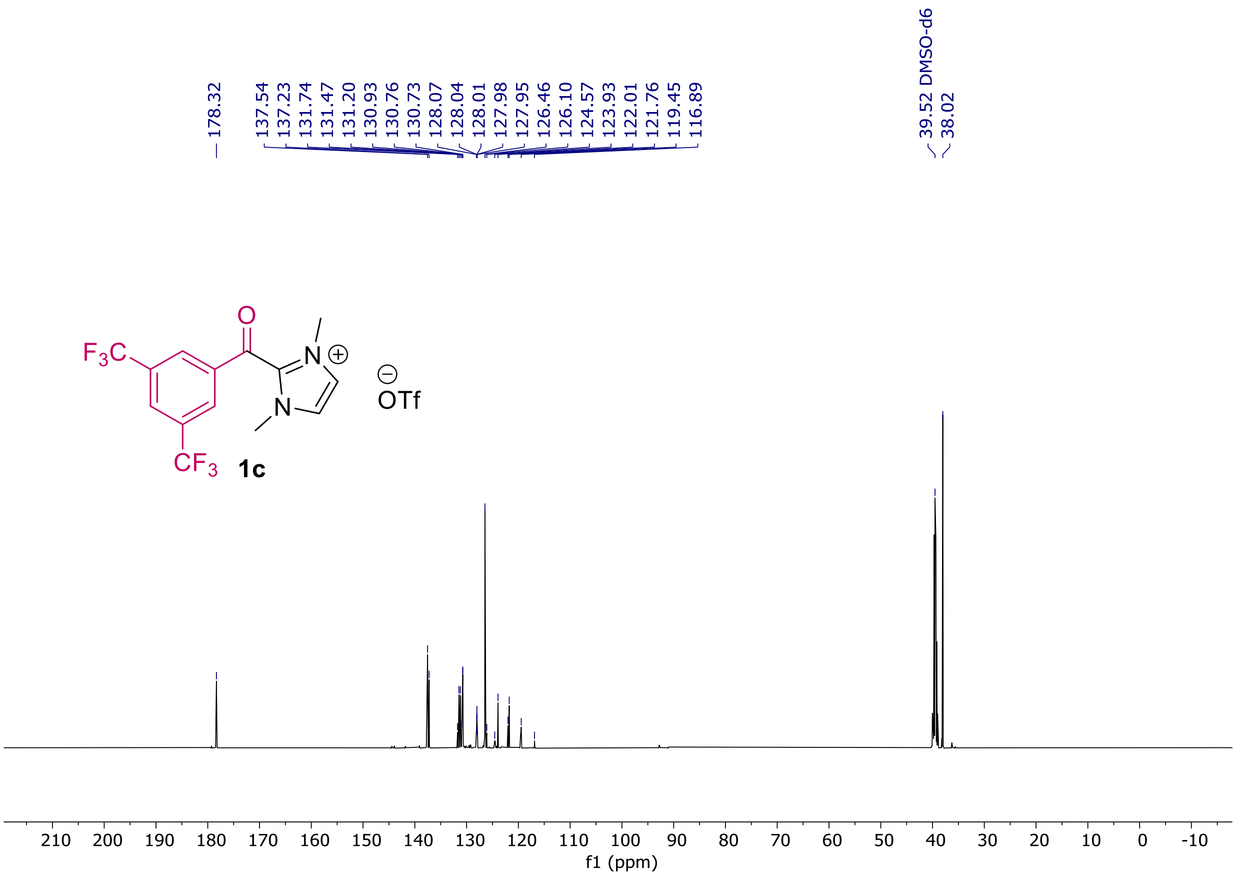

Figure S37: $^{13}C\{^{1}H\}$ NMR spectrum of **1c** in DMSO-d6 (125 MHz)

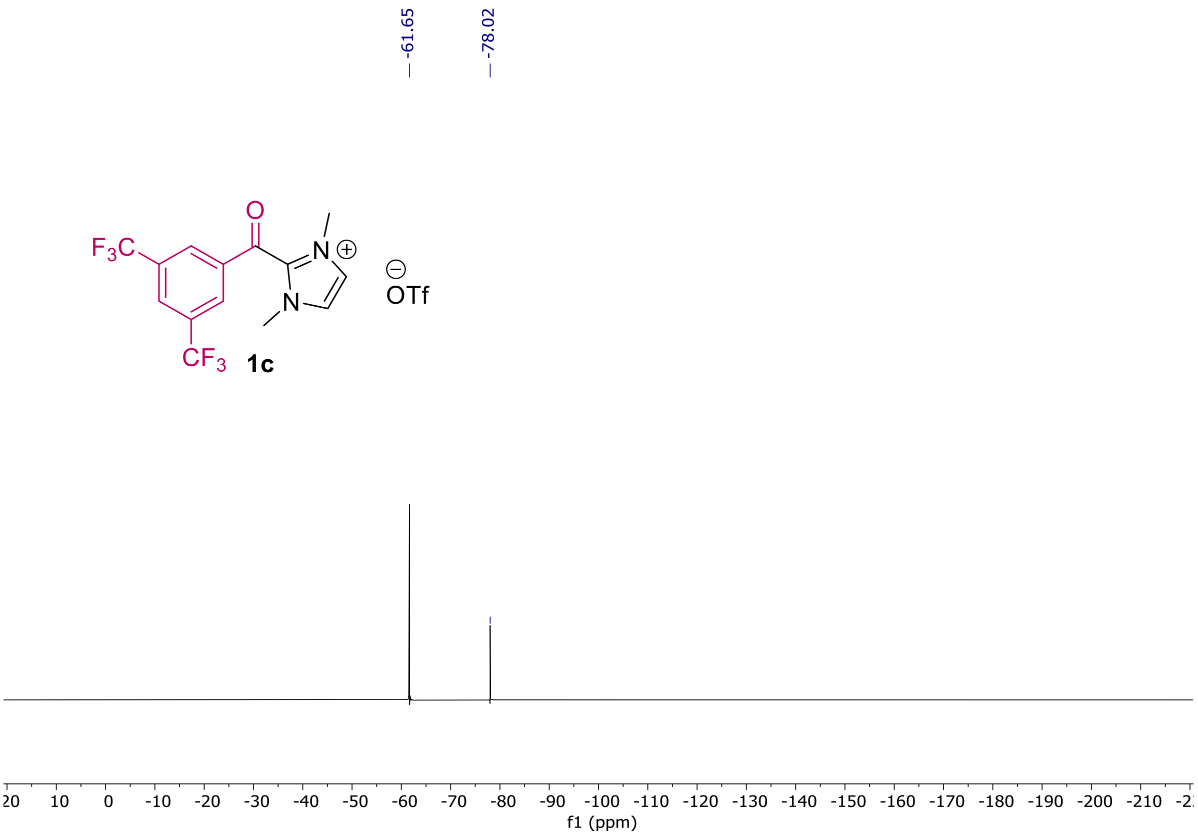

Figure S38: $^{19}F\{^{1}H\}$ NMR spectrum of **1c** in DMSO-d6 (282 MHz)

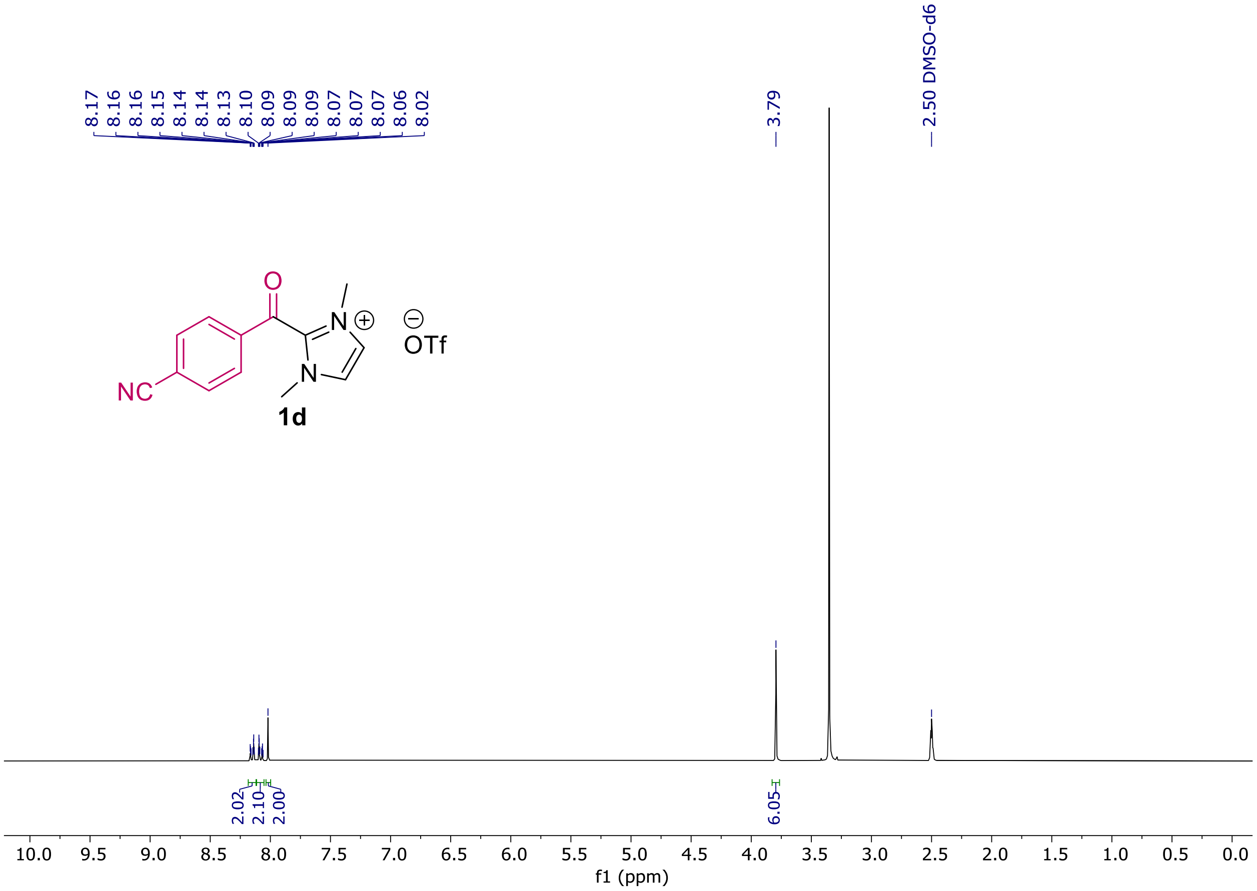

Figure S39: ^{1}H NMR spectrum of **1d** in DMSO-d6 (300 MHz)

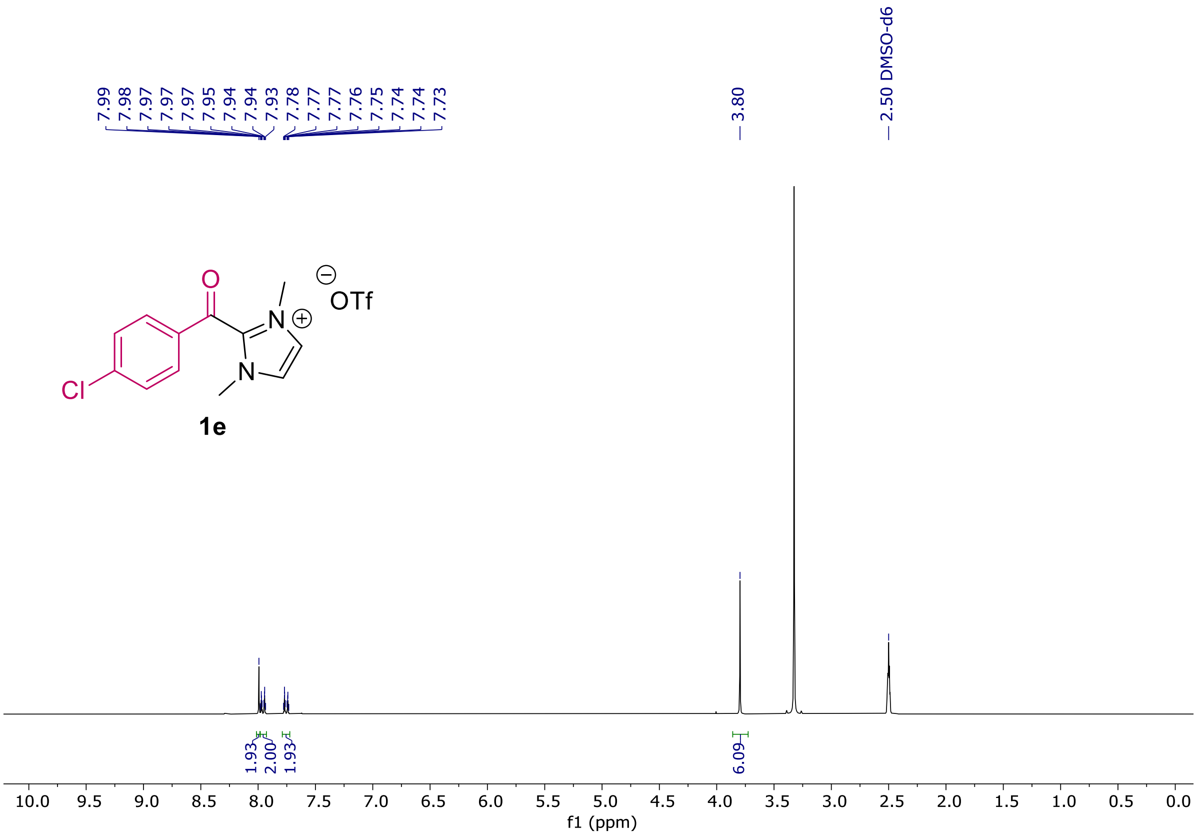

Figure S40: ^{1}H NMR spectrum of **1e** in DMSO-d6 (300 MHz)

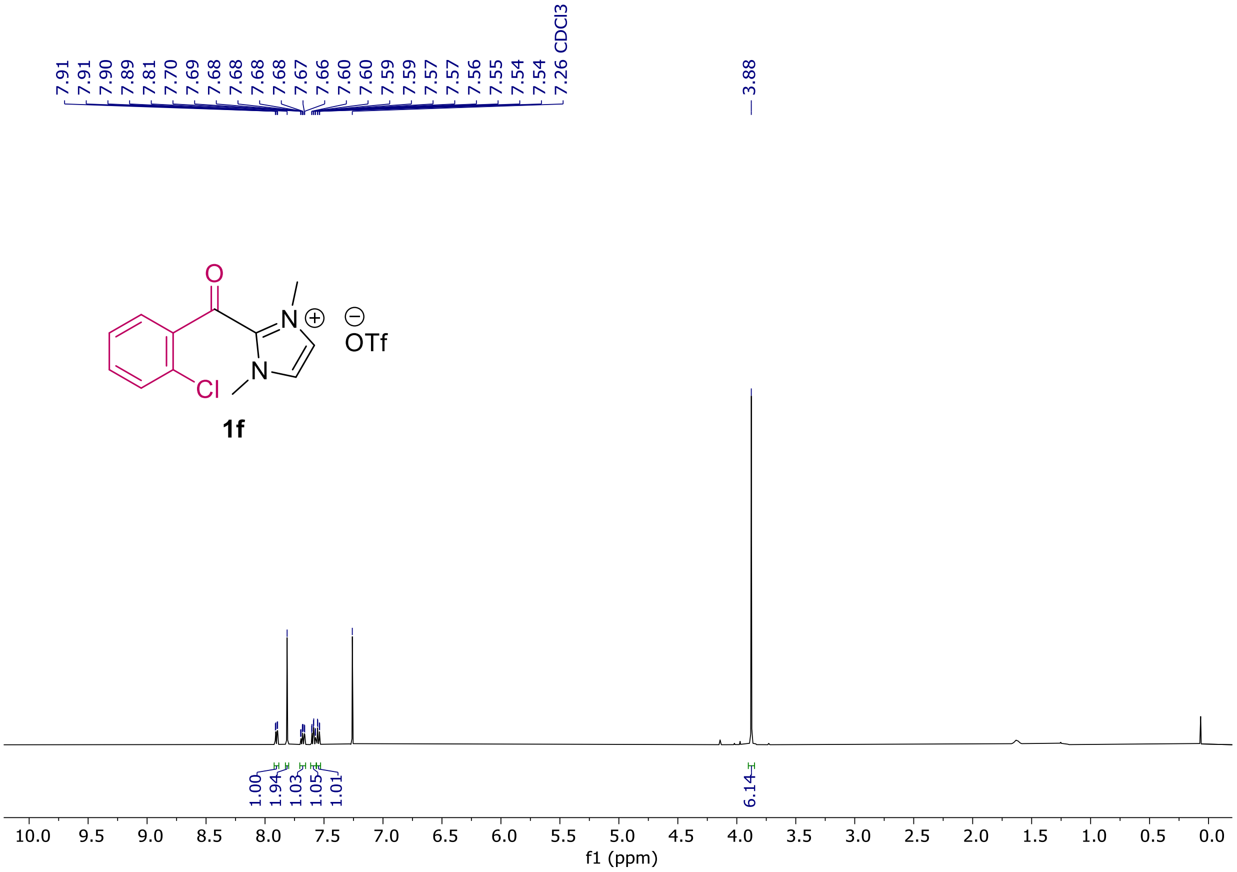

Figure S41: ^{1}H NMR spectrum of **1f** in $CDCl_3$ (500 MHz)

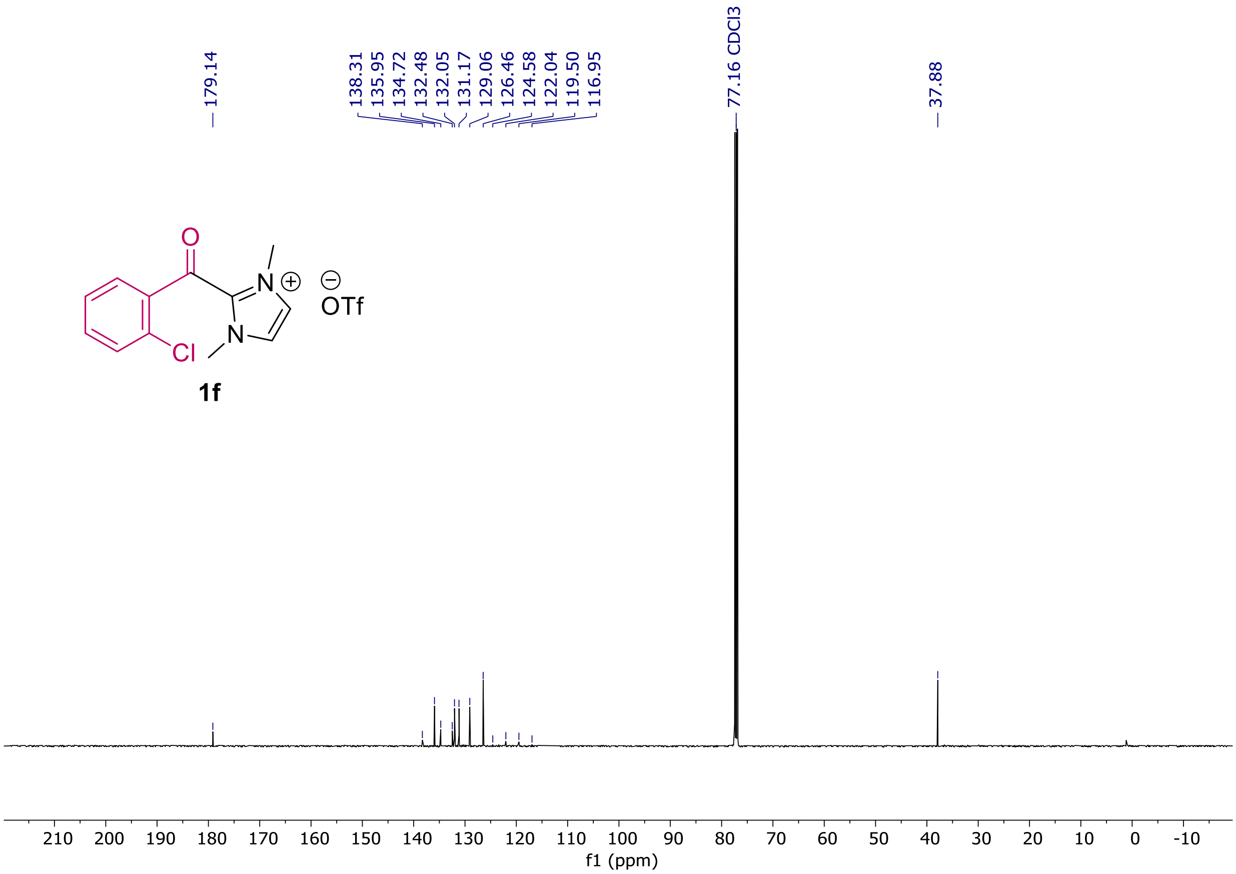

Figure S42: $^{13}C\{^1H\}$ NMR spectrum of **1f** in $CDCl_3$ (125 MHz)

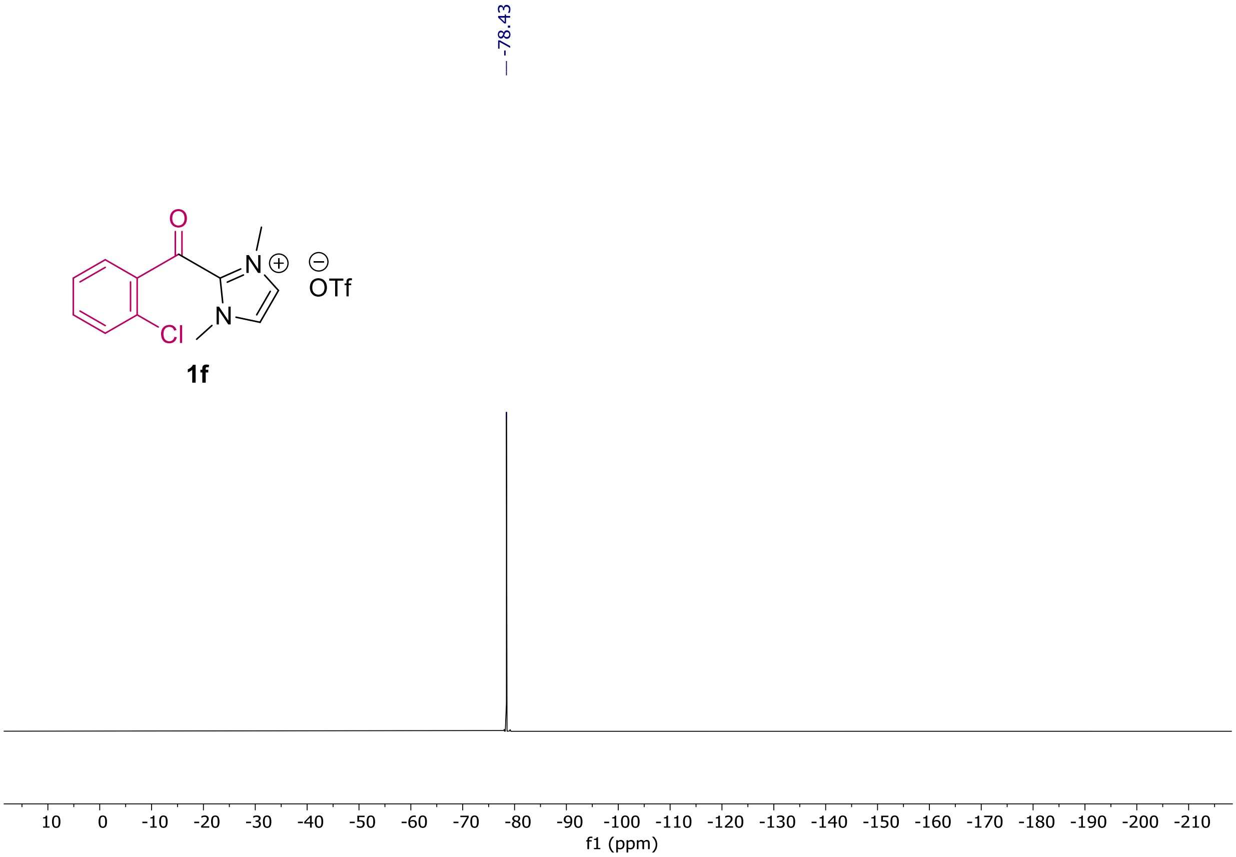

Figure S43: $^{19}F\{^1H\}$ NMR spectrum of **1f** in $CDCl_3$ (282 MHz)

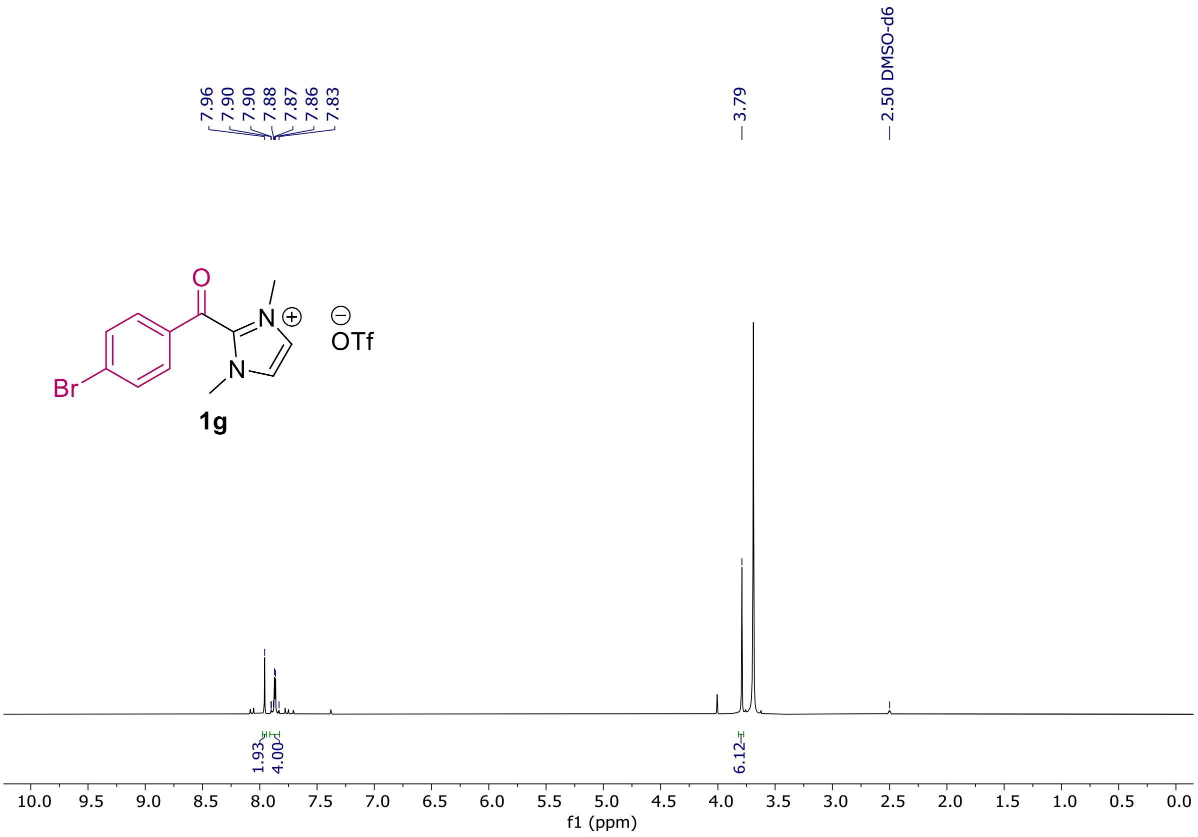

Figure S44: ^{1}H NMR spectrum of **1g** in DMSO-d6 (500 MHz)

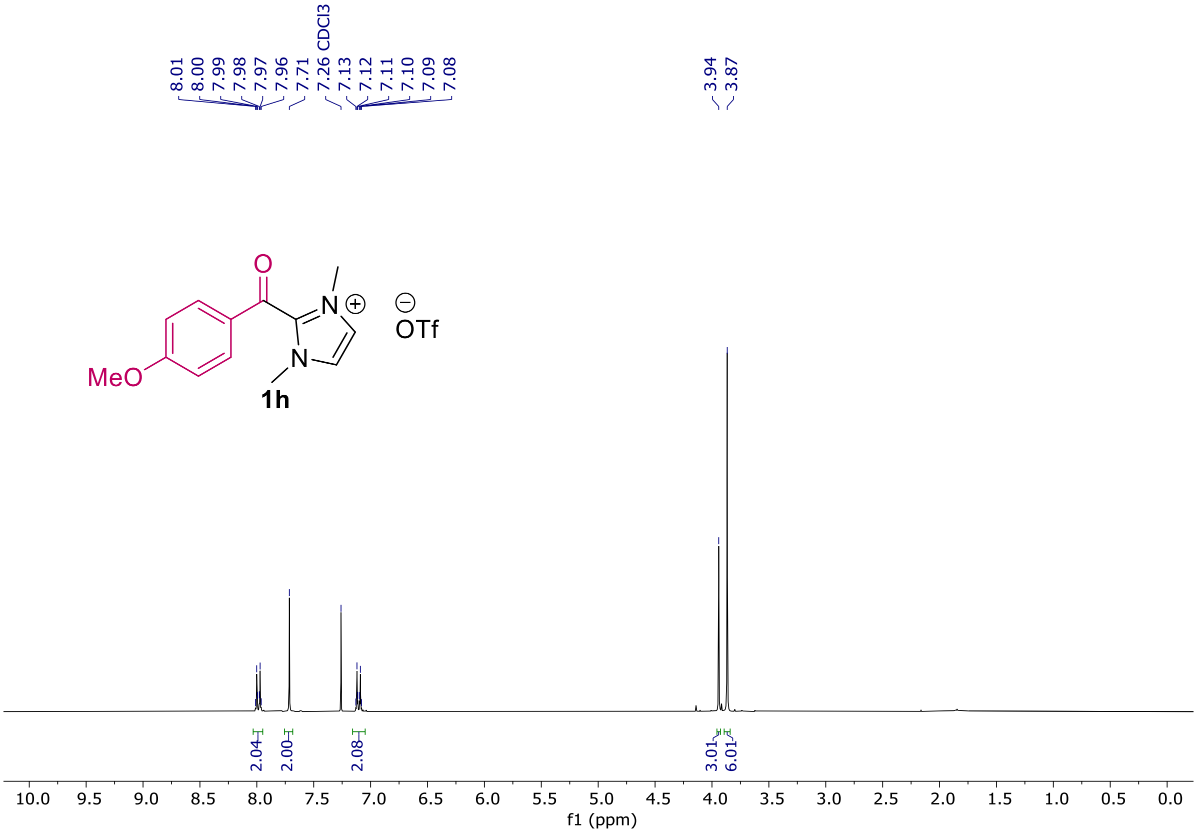

Figure S45: ^{1}H NMR spectrum of **1h** in $CDCl_3$ (300 MHz)

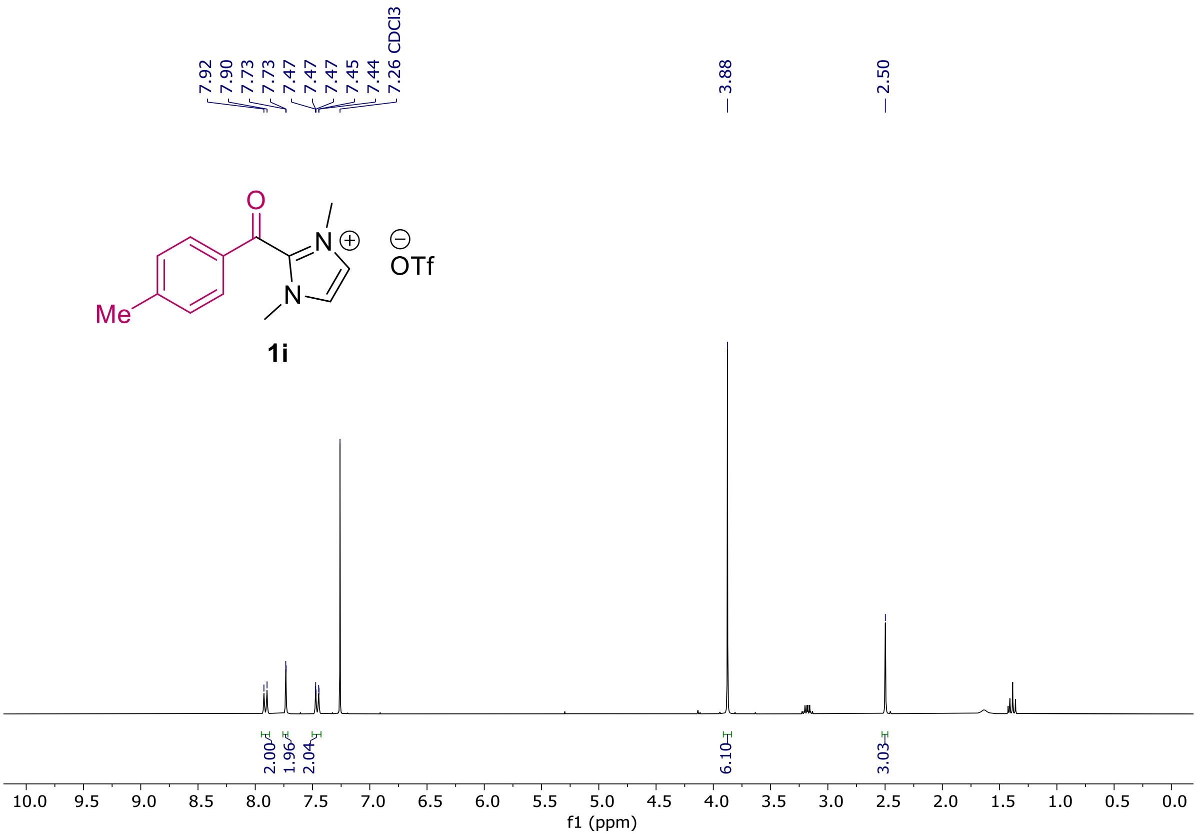

Figure S46: ^{1}H NMR spectrum of **1i** in $CDCl_3$ (300 MHz)

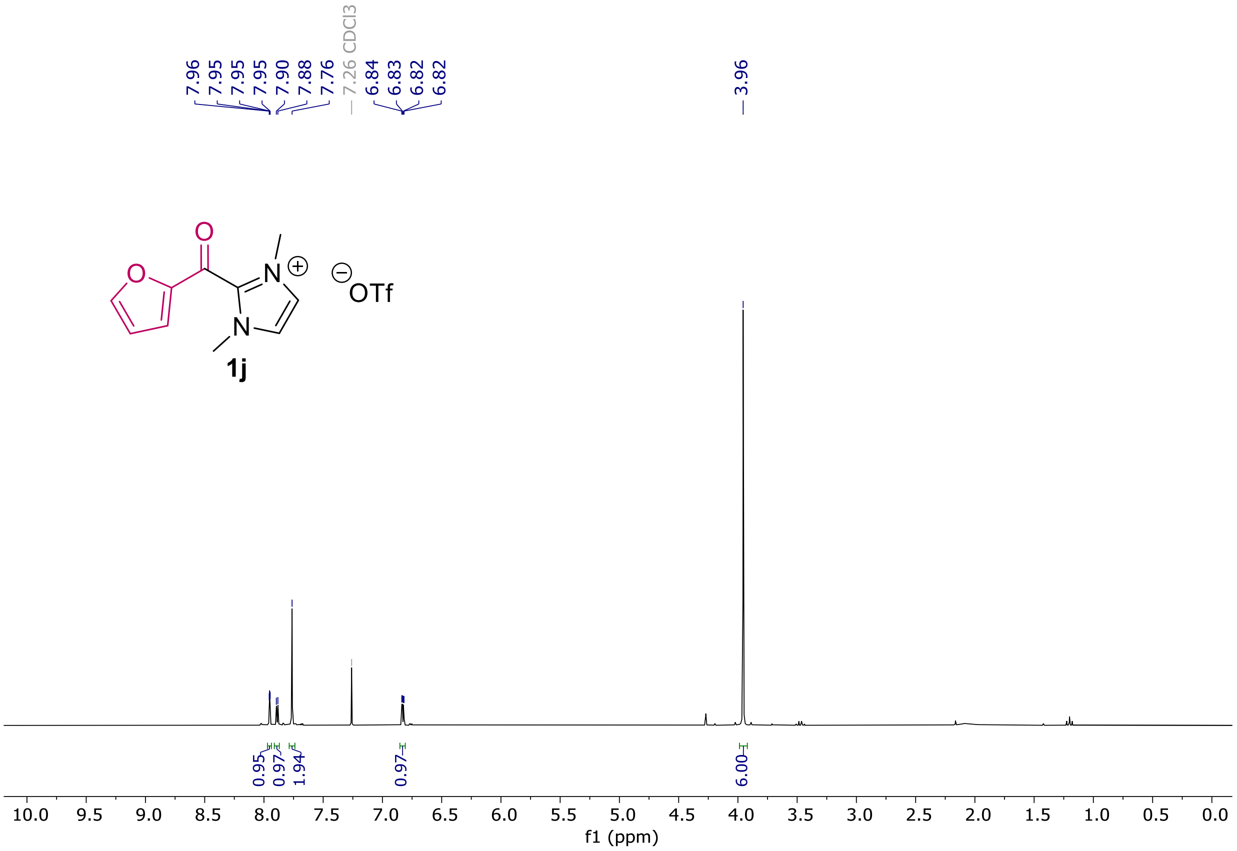

Figure S47: ^{1}H NMR spectrum of **1j** in $CDCl_3$ (300 MHz)

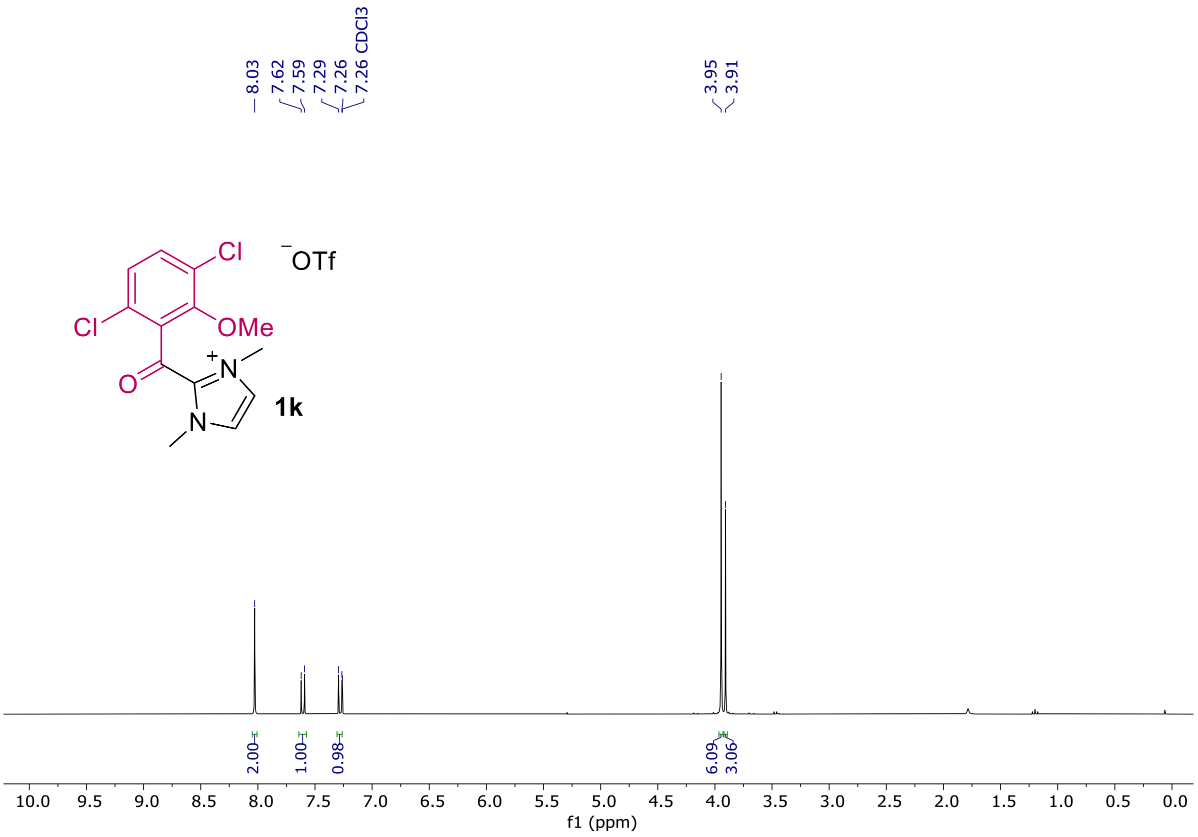

Figure S48: ^{1}H NMR spectrum of **1k** in $CDCl_3$ (300 MHz)

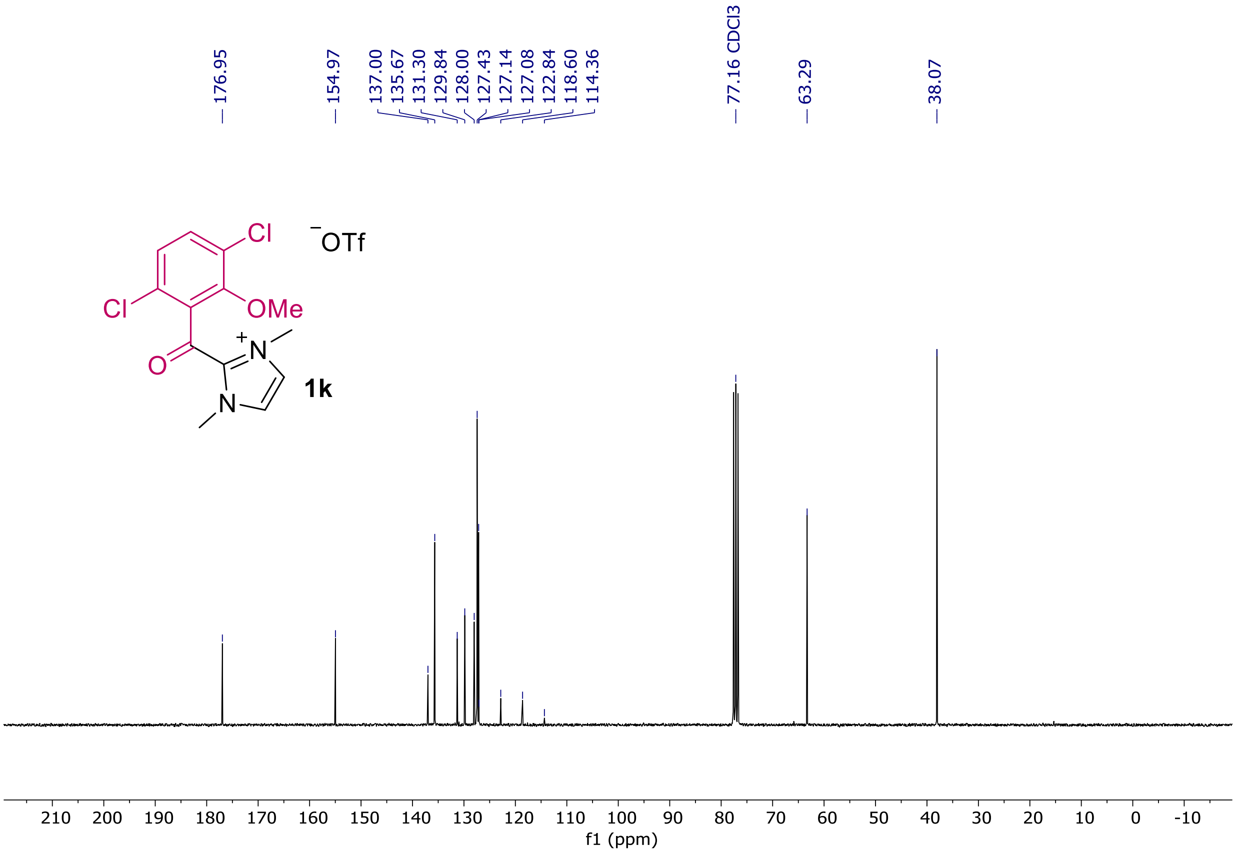

Figure S49: $^{13}C\{^{1}H\}$ NMR spectrum of **1k** in $CDCl_3$ (75 MHz)

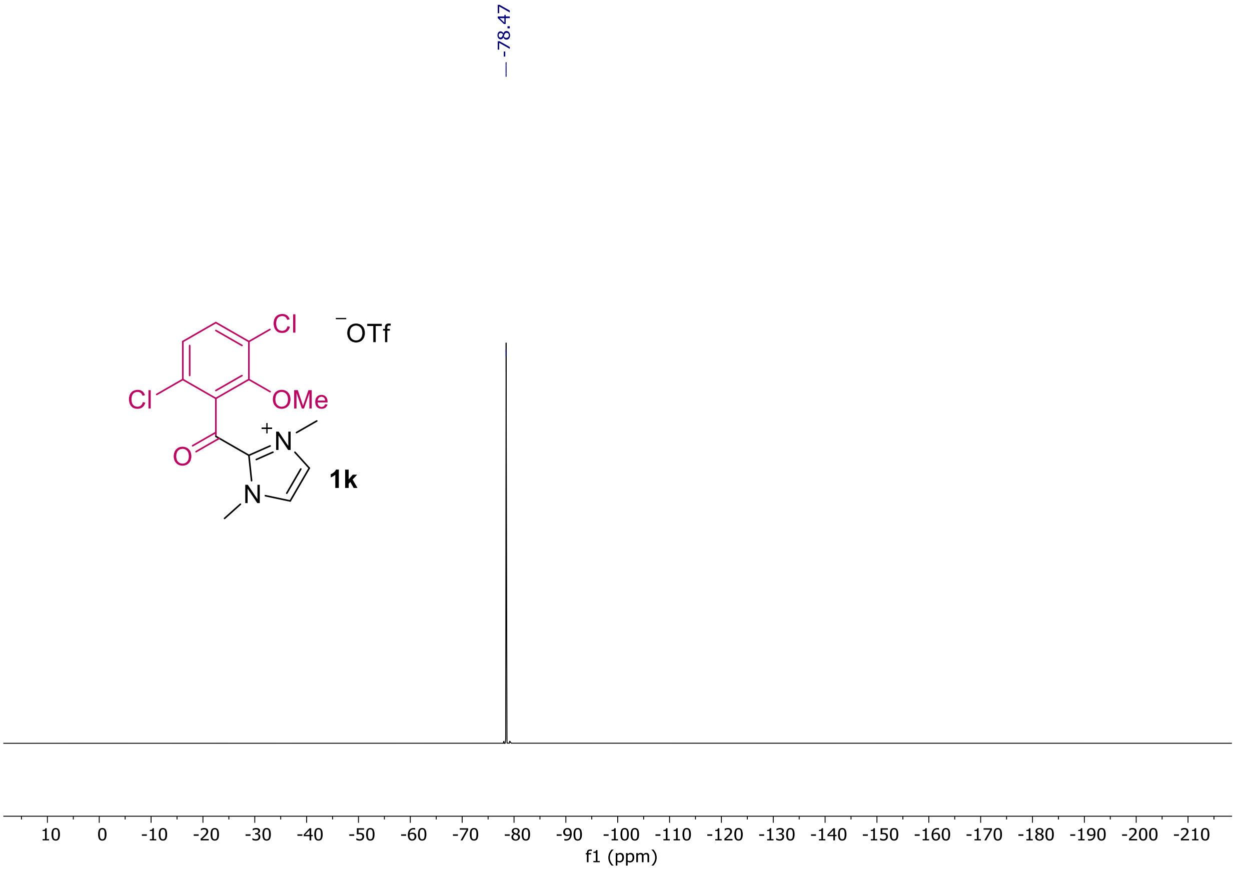

Figure S50: $^{19}F\{^1H\}$ NMR spectrum of **1k** in $CDCl_3$ (282 MHz)

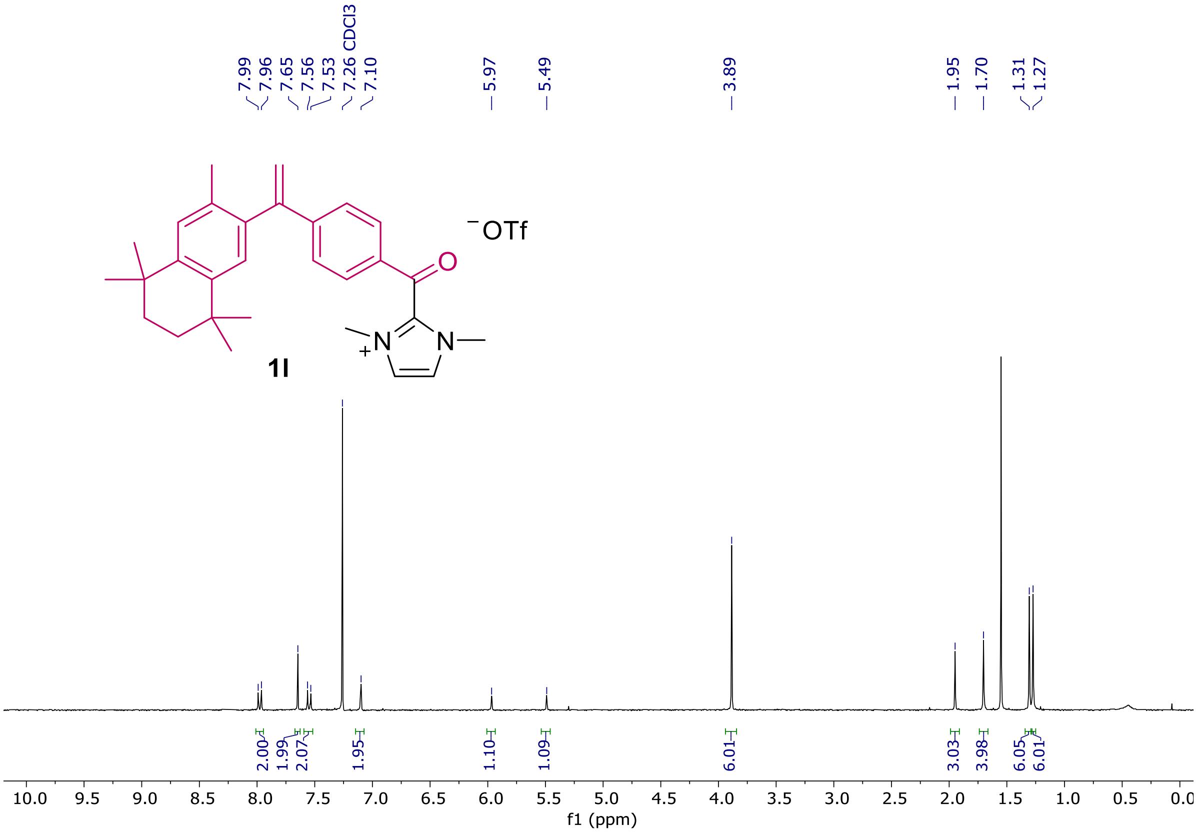

Figure S51: ^1H NMR spectrum of **1l** in $CDCl_3$ (300 MHz)

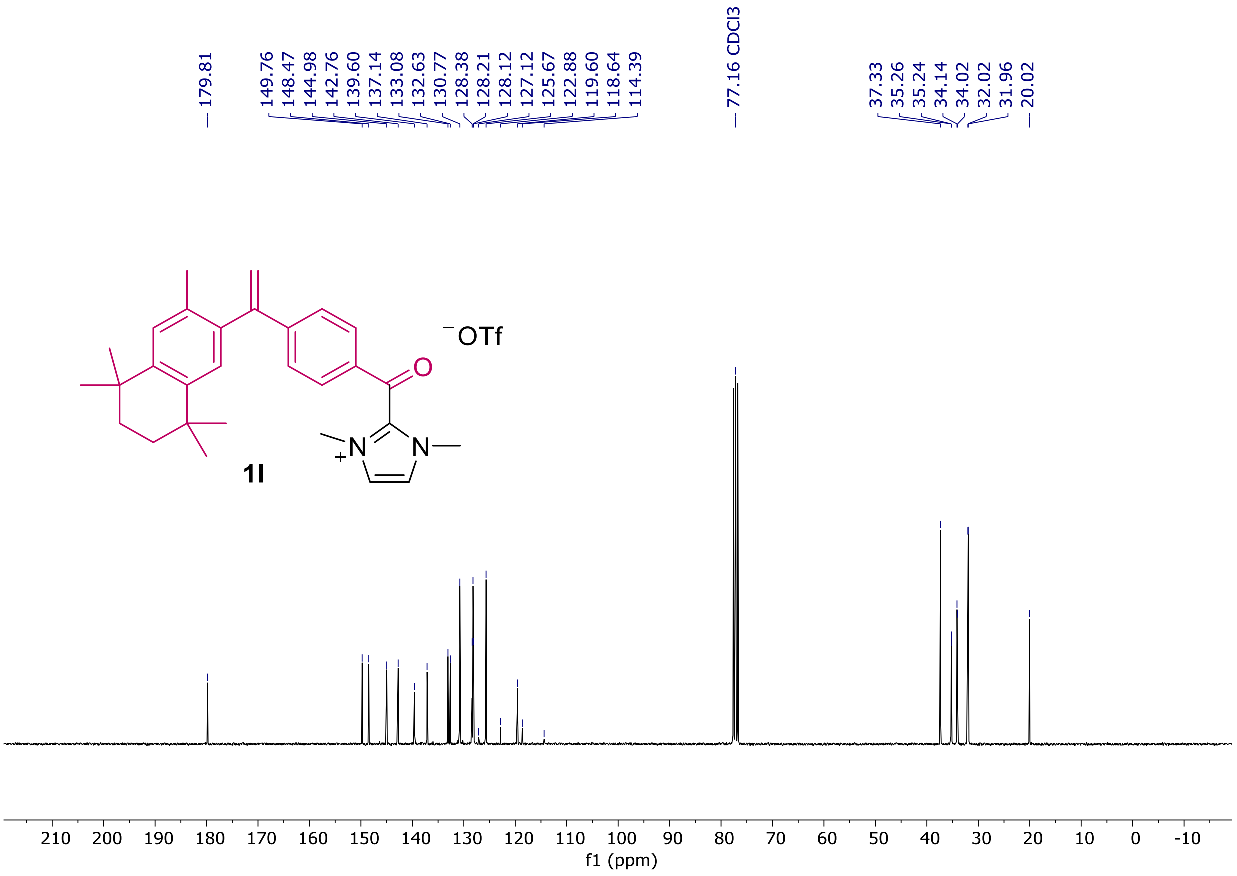

Figure S52: $^{13}C\{^{1}H\}$ NMR spectrum of **1l** in $CDCl_3$ (75 MHz)

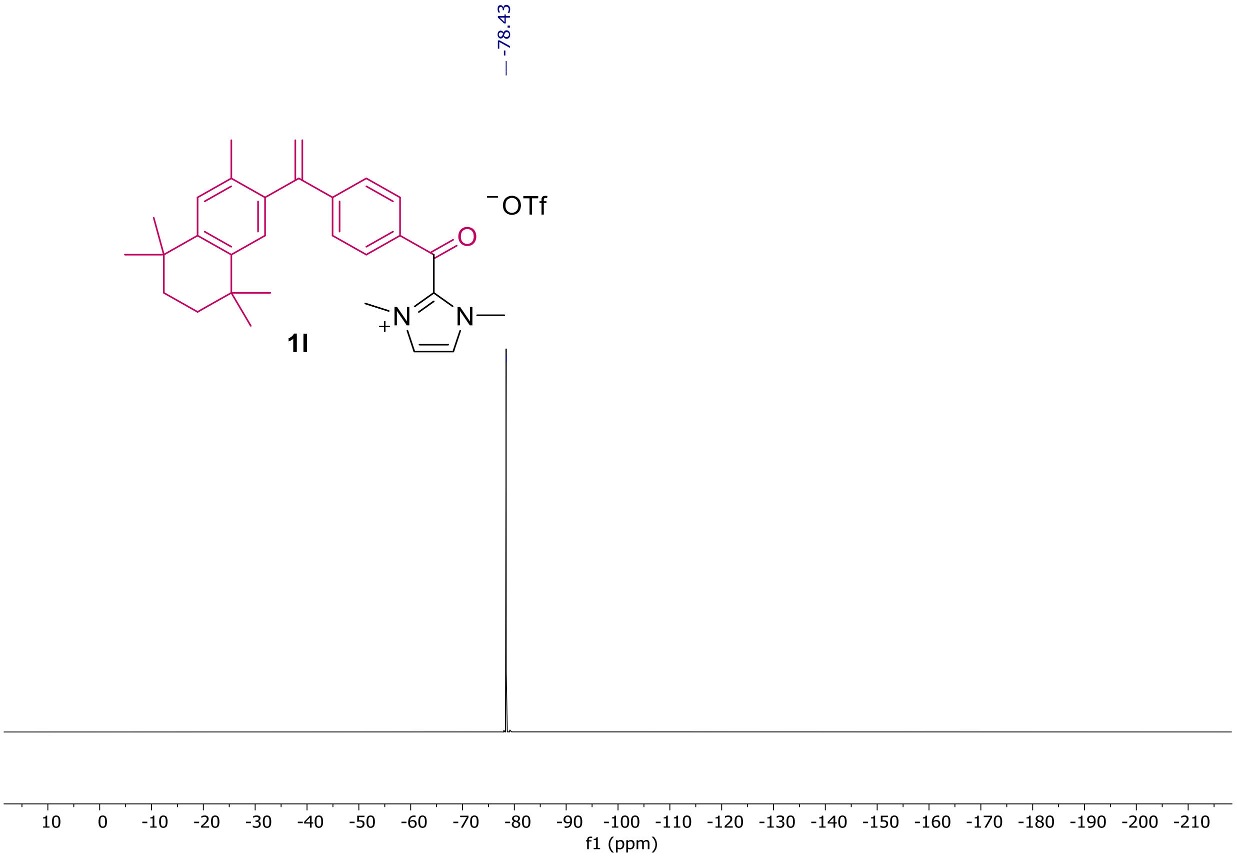

Figure S53: $^{19}F\{^{1}H\}$ NMR spectrum of **1l** in $CDCl_3$ (282 MHz)

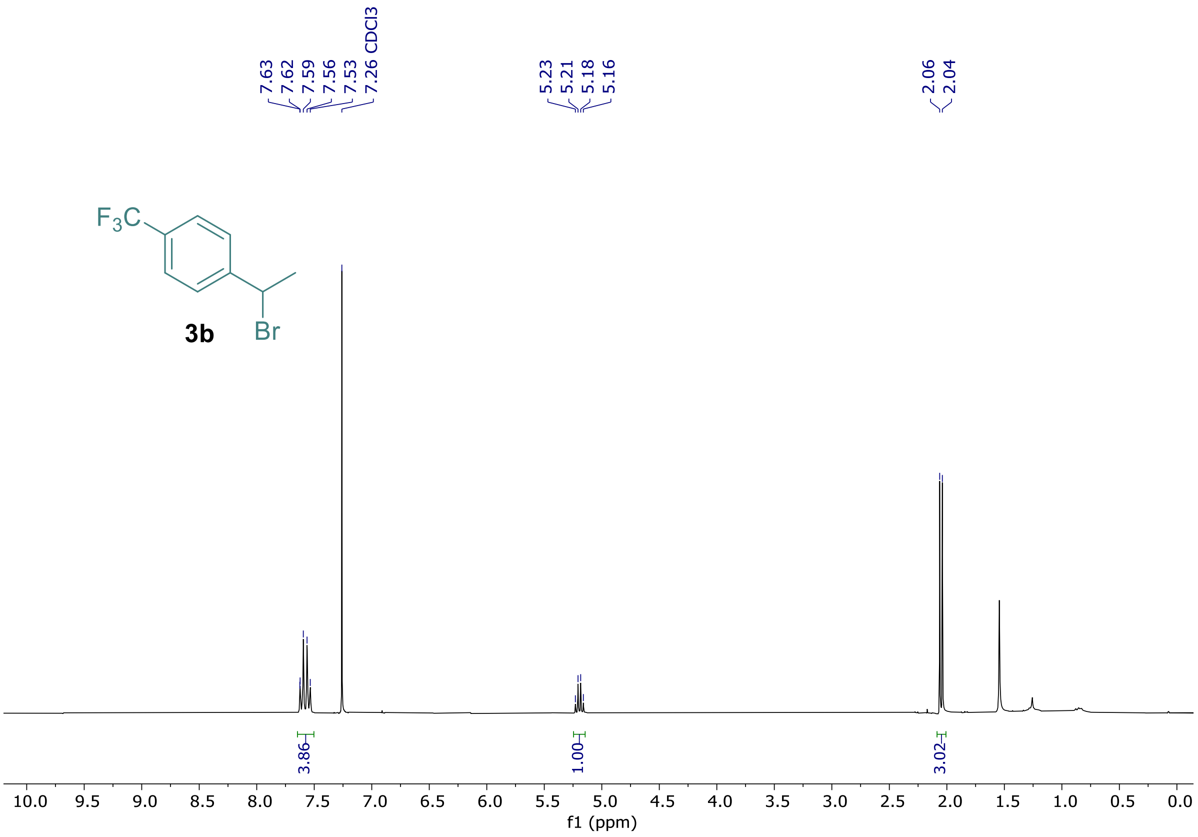

Figure S54: ^{1}H NMR spectrum of **3b** in $CDCl_3$ (300 MHz)

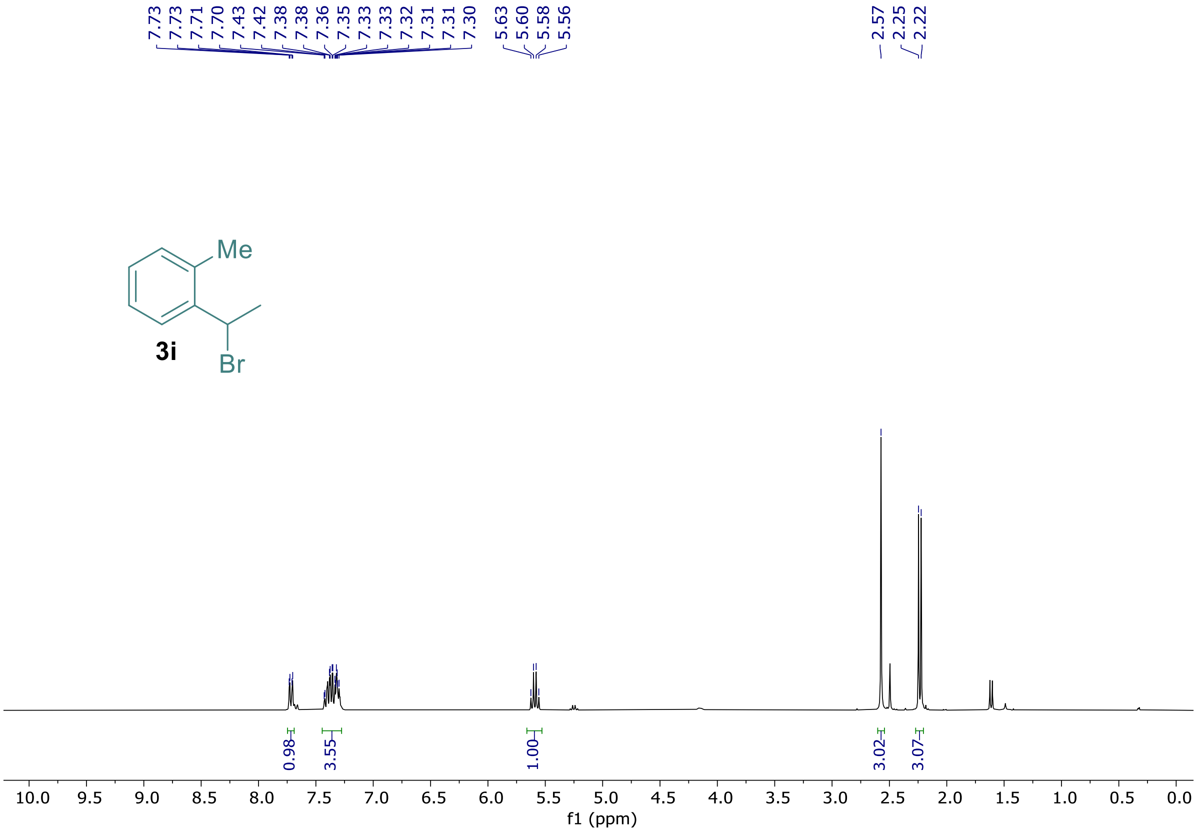

Figure S55: ^{1}H NMR spectrum of **3i** in $CDCl_3$ (300 MHz)

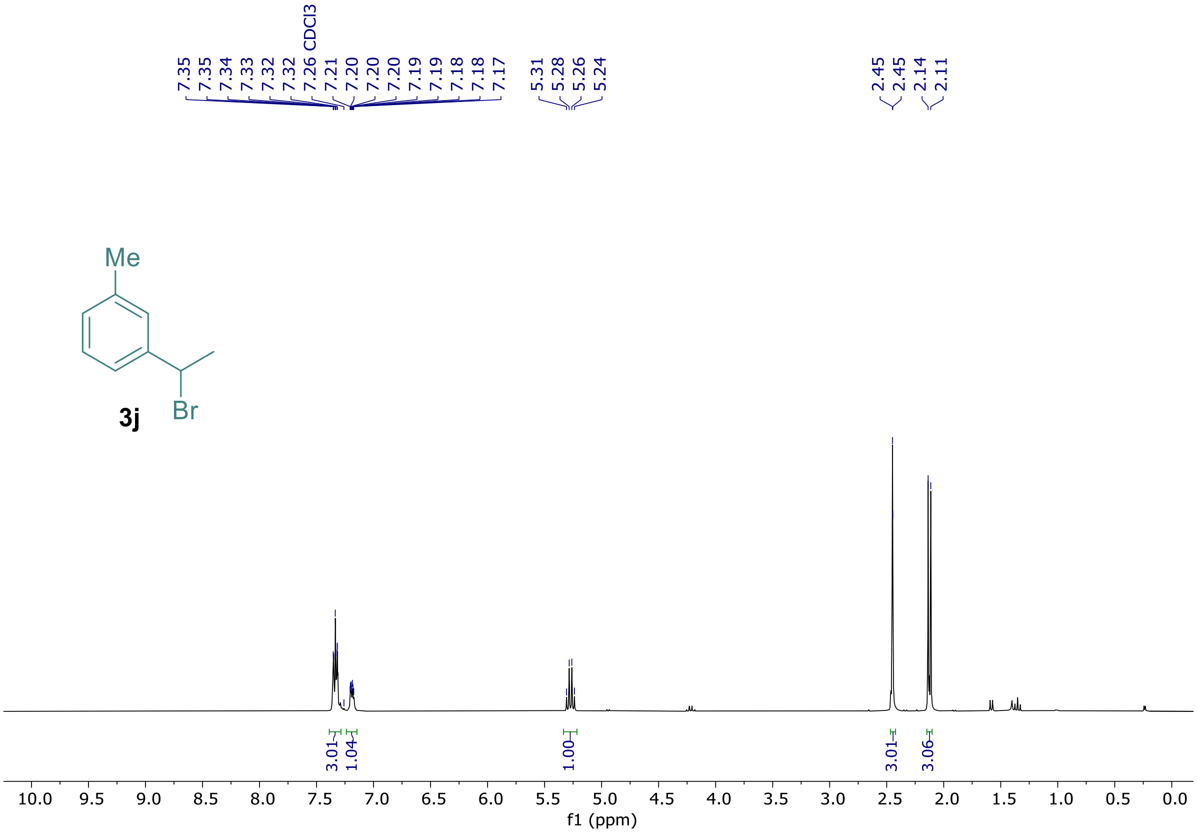

Figure S56: ^{1}H NMR spectrum of **3j** in $CDCl_3$ (300 MHz)

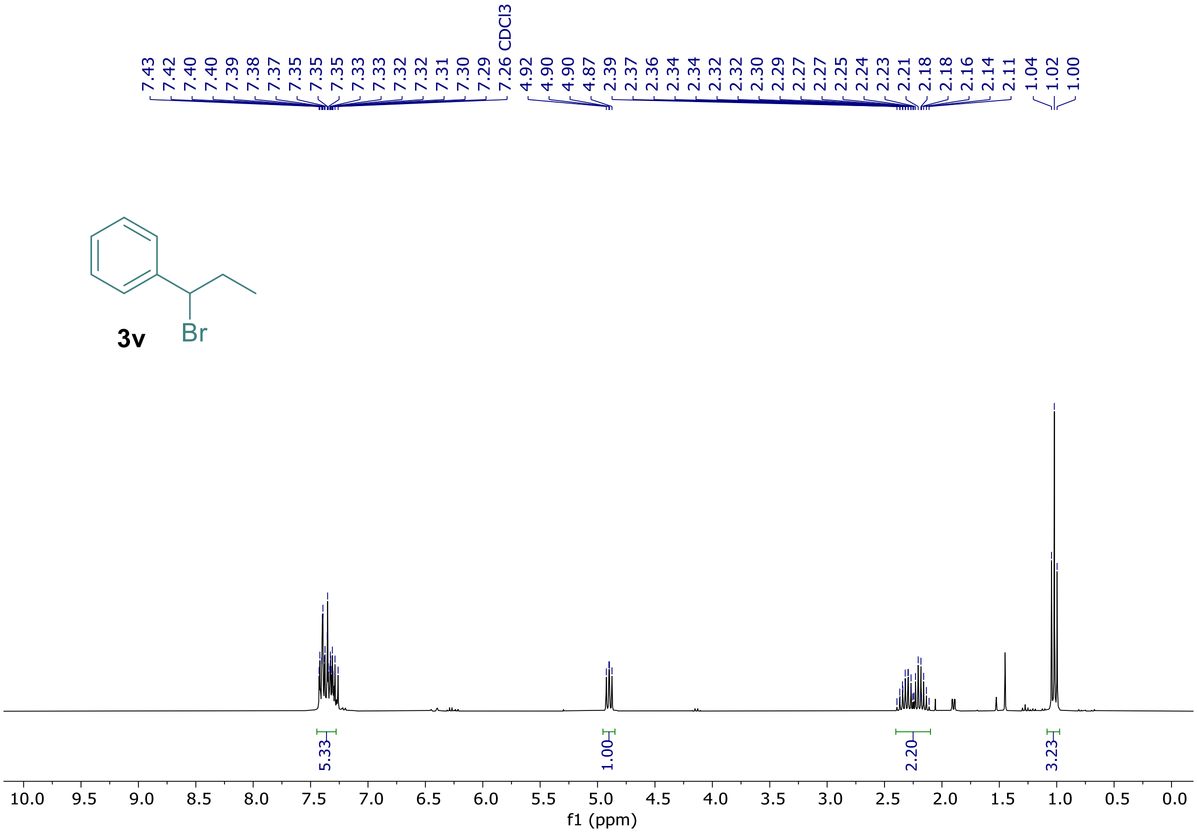

Figure S57: ^{1}H NMR spectrum of **3v** in $CDCl_3$ (300 MHz)

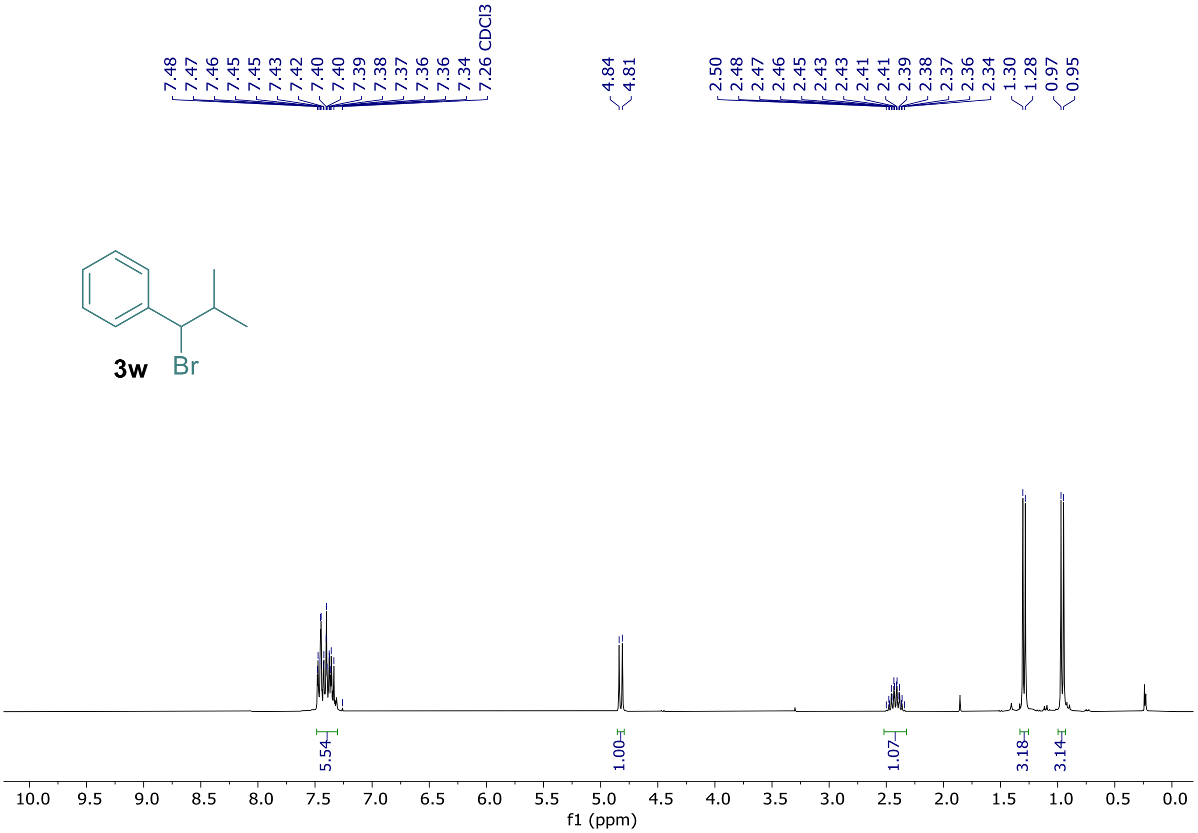

Figure S58: ^{1}H NMR spectrum of **3w** in $CDCl_3$ (300 MHz)

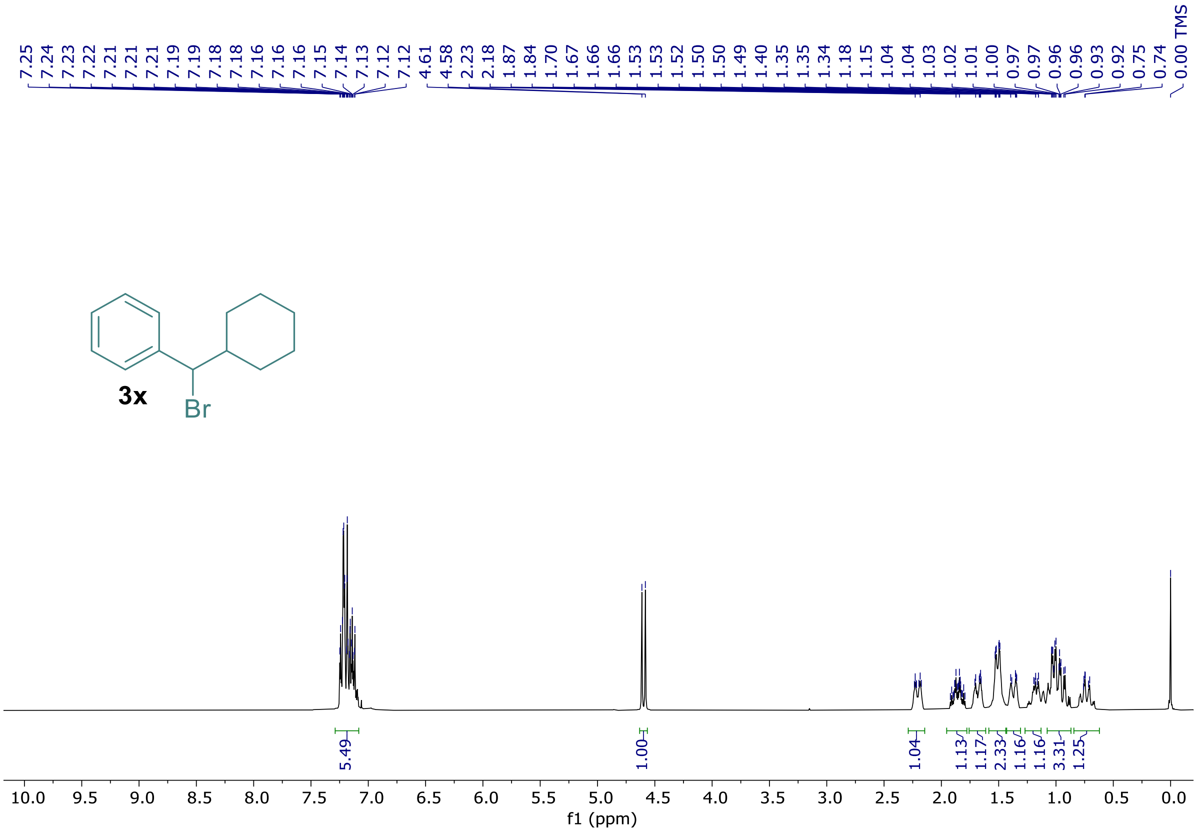

Figure S59: ^{1}H NMR spectrum of **3x** in $CDCl_3$ (300 MHz)

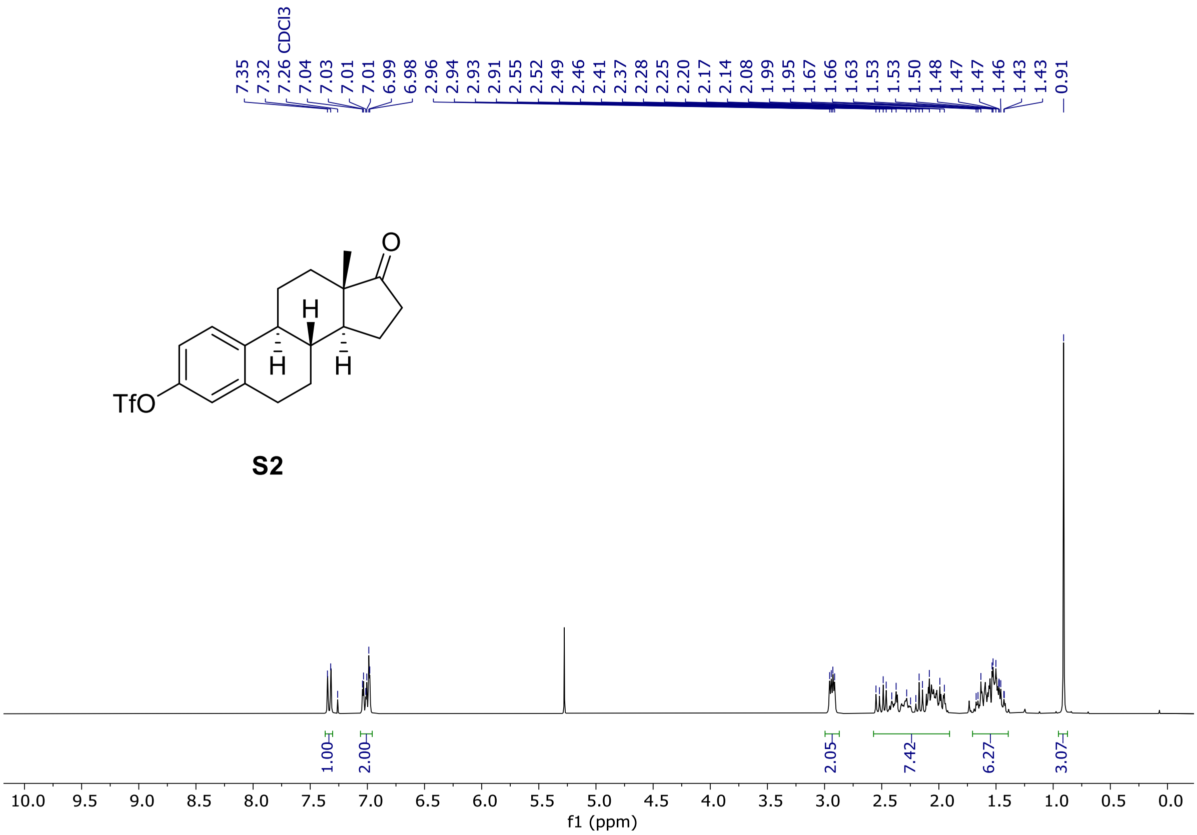

Figure S60: ^{1}H NMR spectrum of **S2** in $CDCl_3$ (300 MHz)

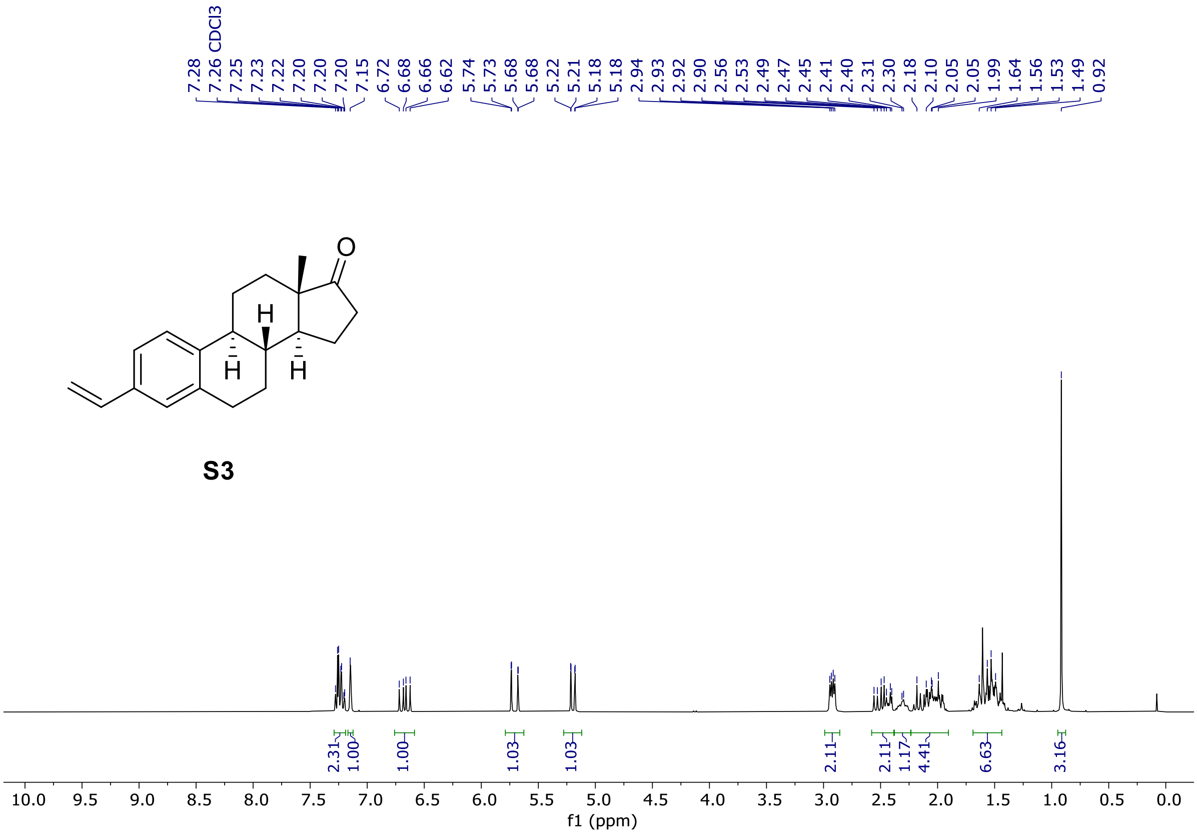

Figure S61: ^{1}H NMR spectrum of **S3** in $CDCl_3$ (300 MHz)

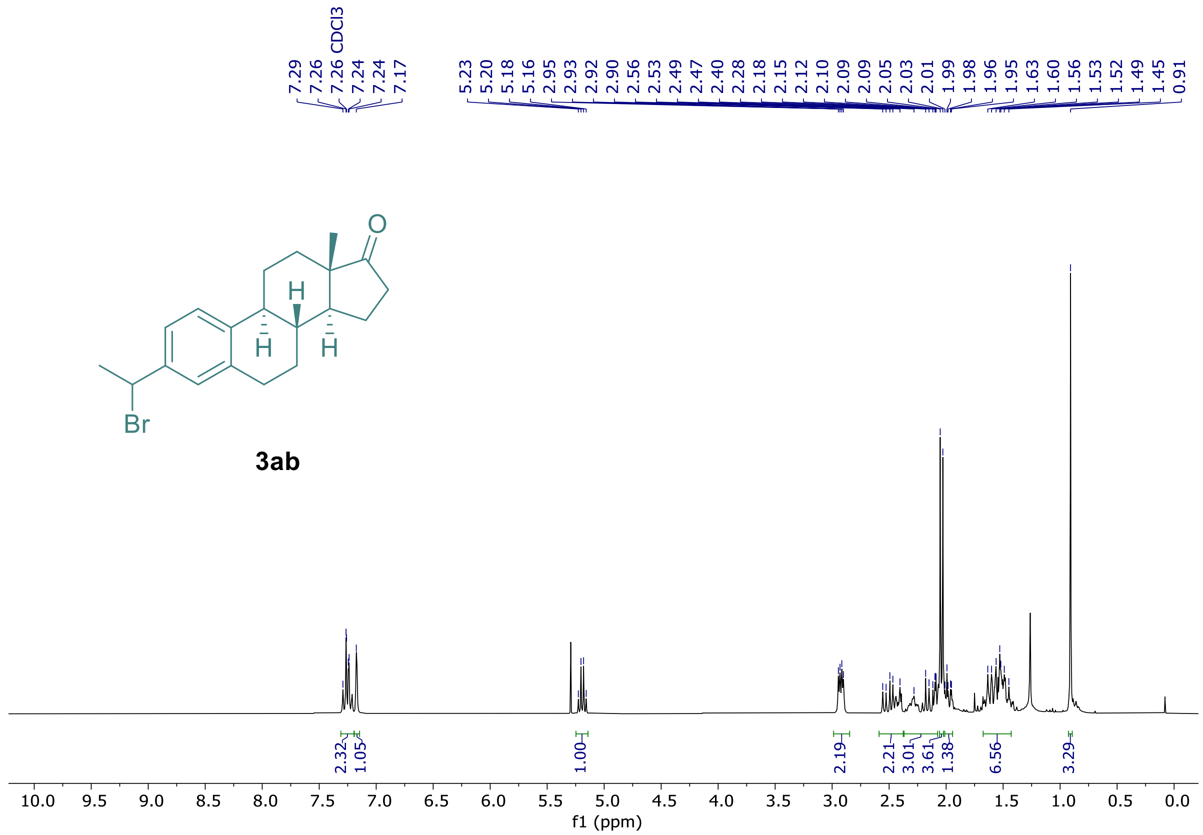

Figure S62: ^{1}H NMR spectrum of **3ab** in $CDCl_3$ (300 MHz)

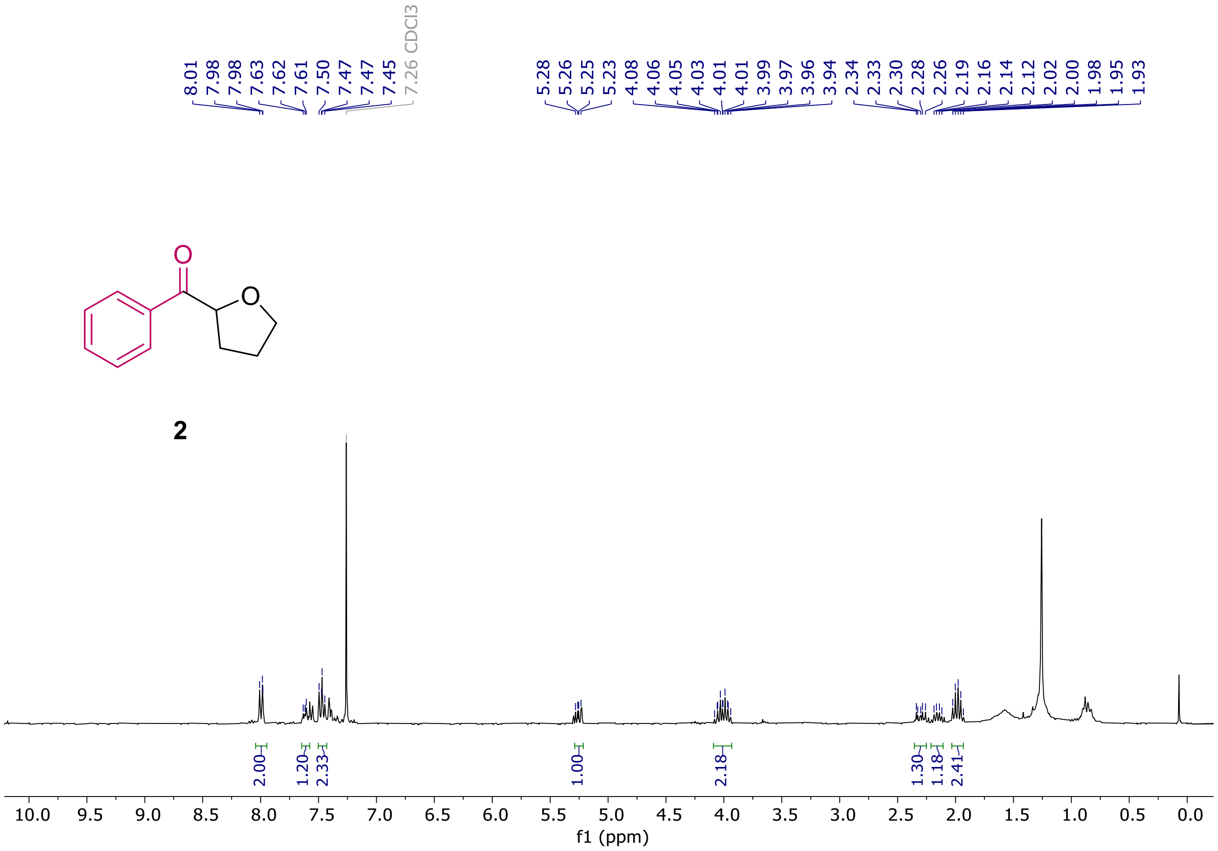

Figure S63: ^{1}H NMR spectrum of **2** in $CDCl_3$ (300 MHz)

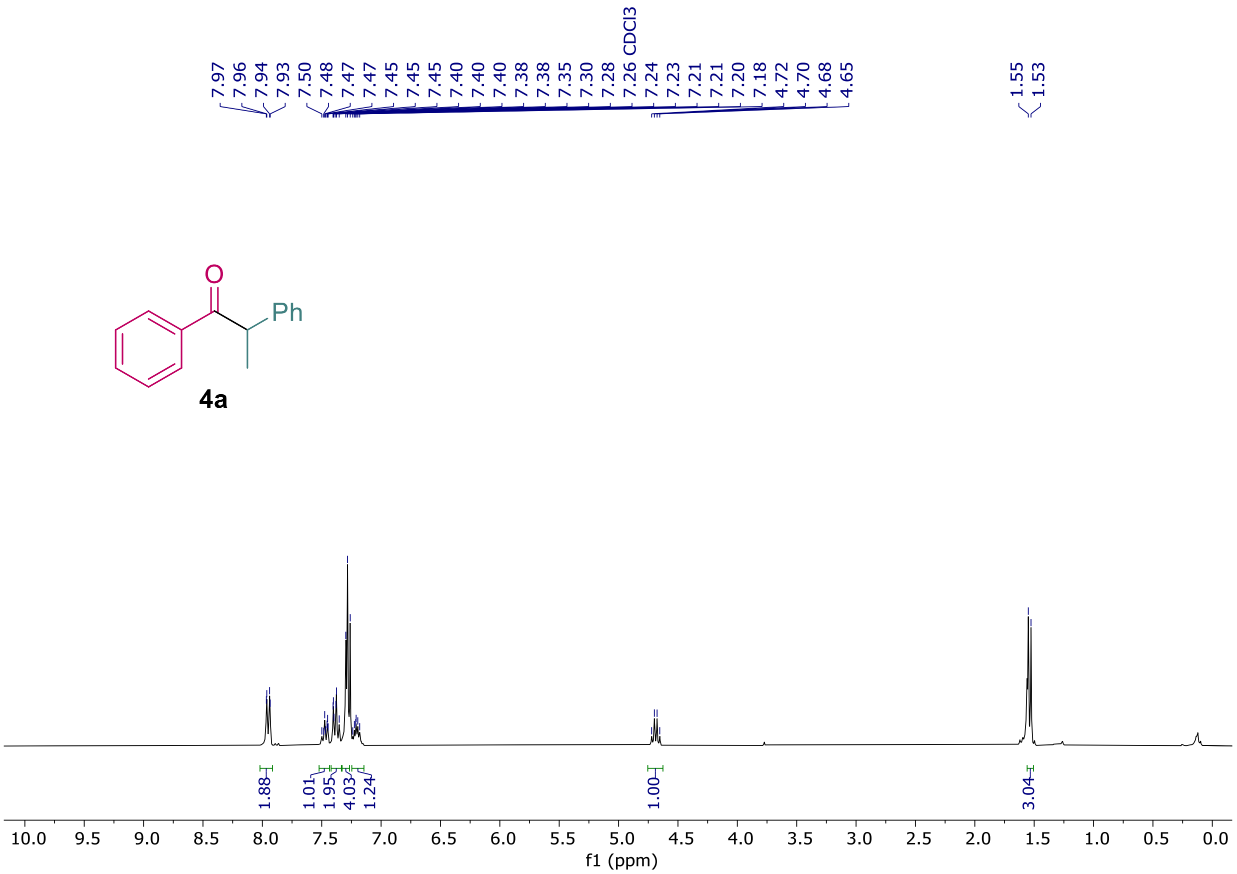

Figure S64: ^{1}H NMR spectrum of **4a** in $CDCl_3$ (300 MHz)

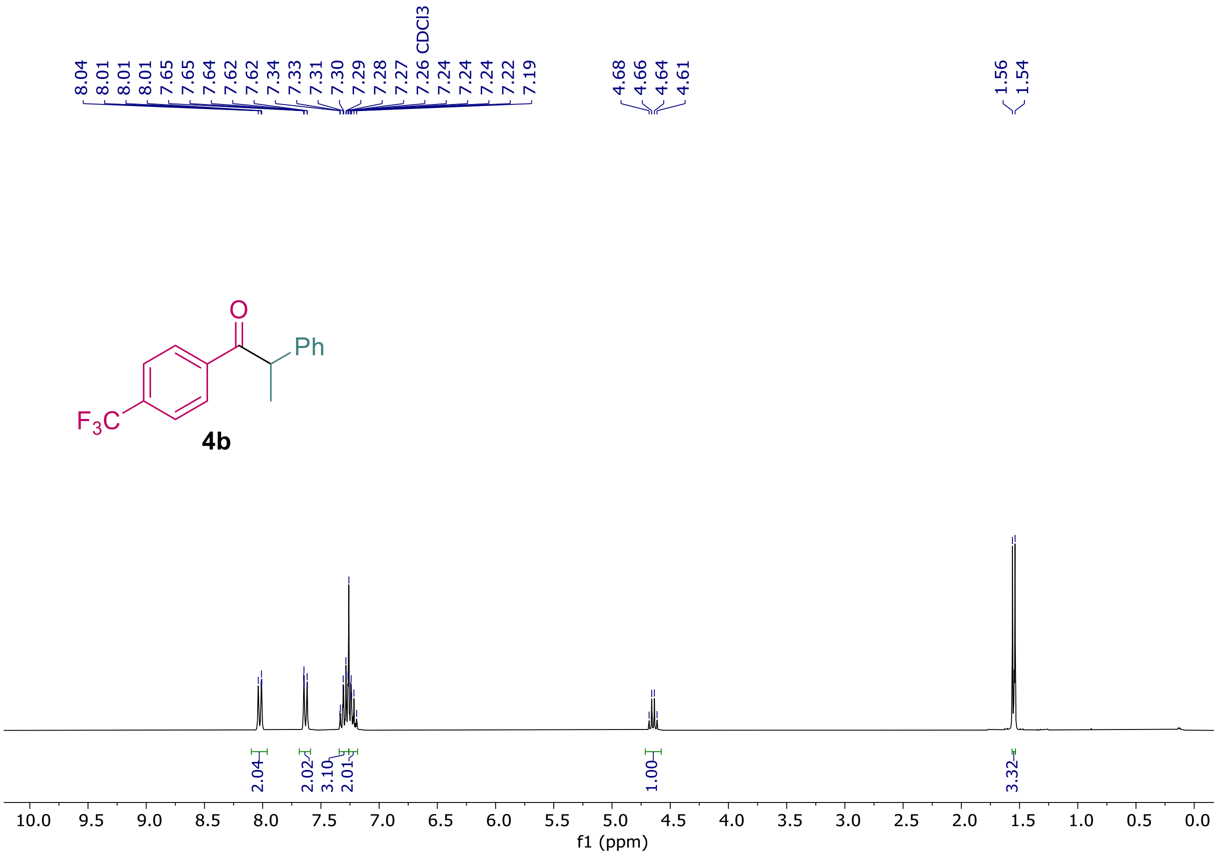

Figure S65: ^{1}H NMR spectrum of **4b** in $CDCl_3$ (300 MHz)

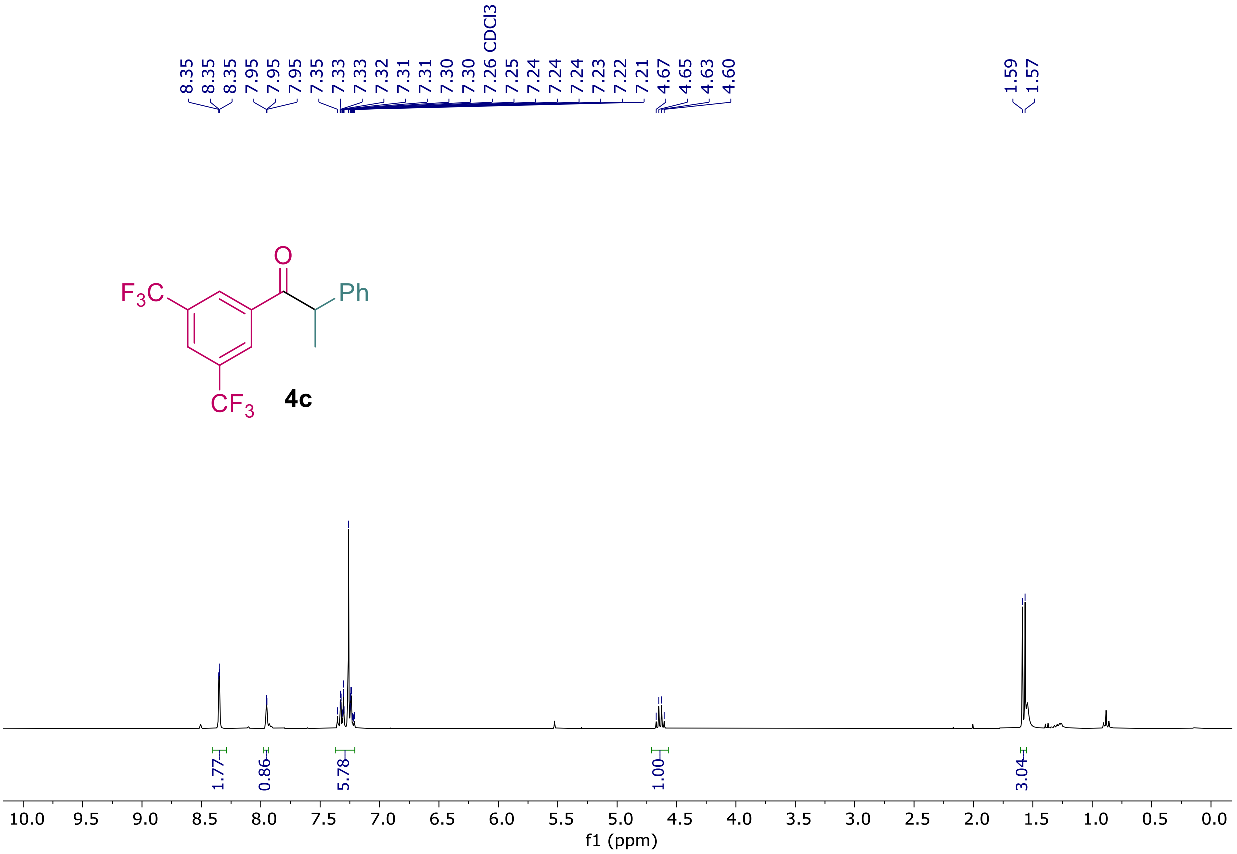

Figure S66: ^{1}H NMR spectrum of **4c** in $CDCl_3$ (300 MHz)

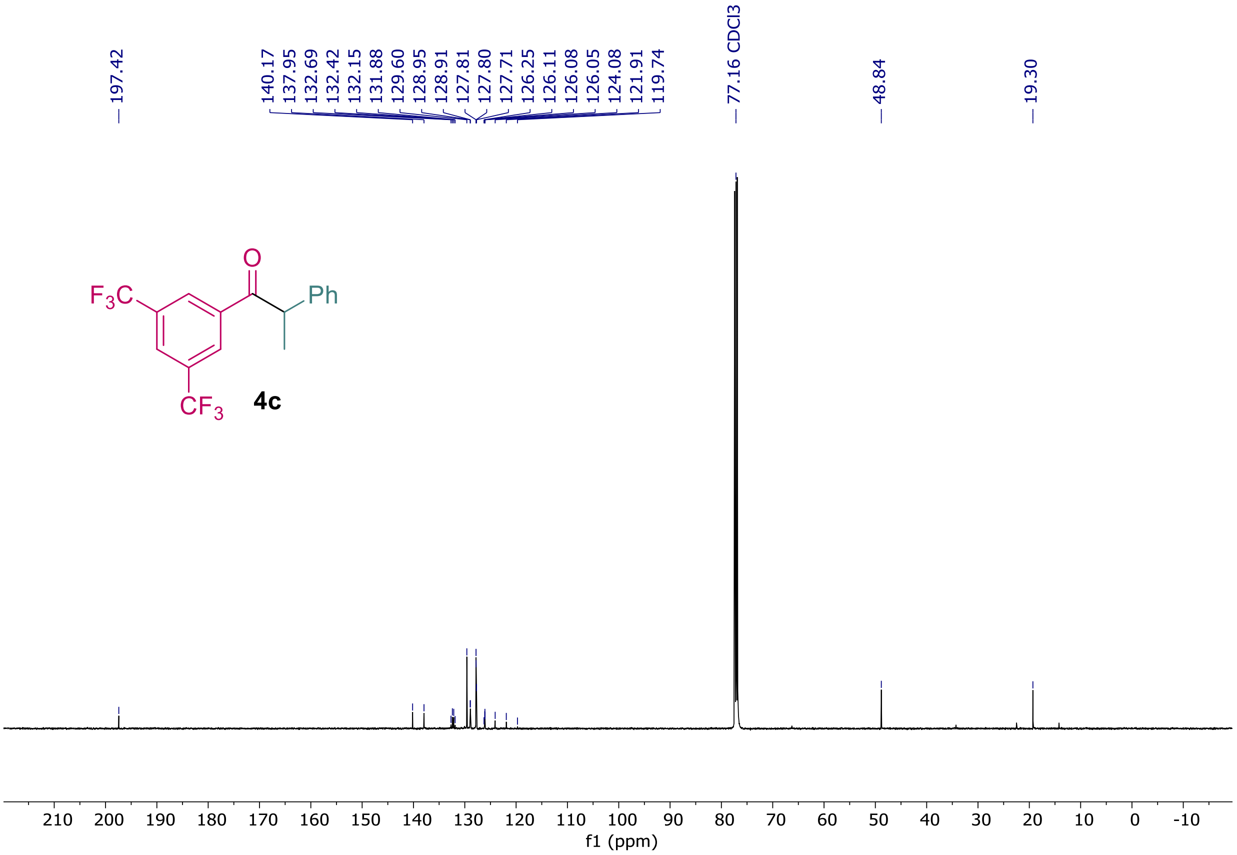

Figure S67: $^{13}C\{^{1}H\}$ NMR spectrum of **4c** in $CDCl_3$ (125 MHz)

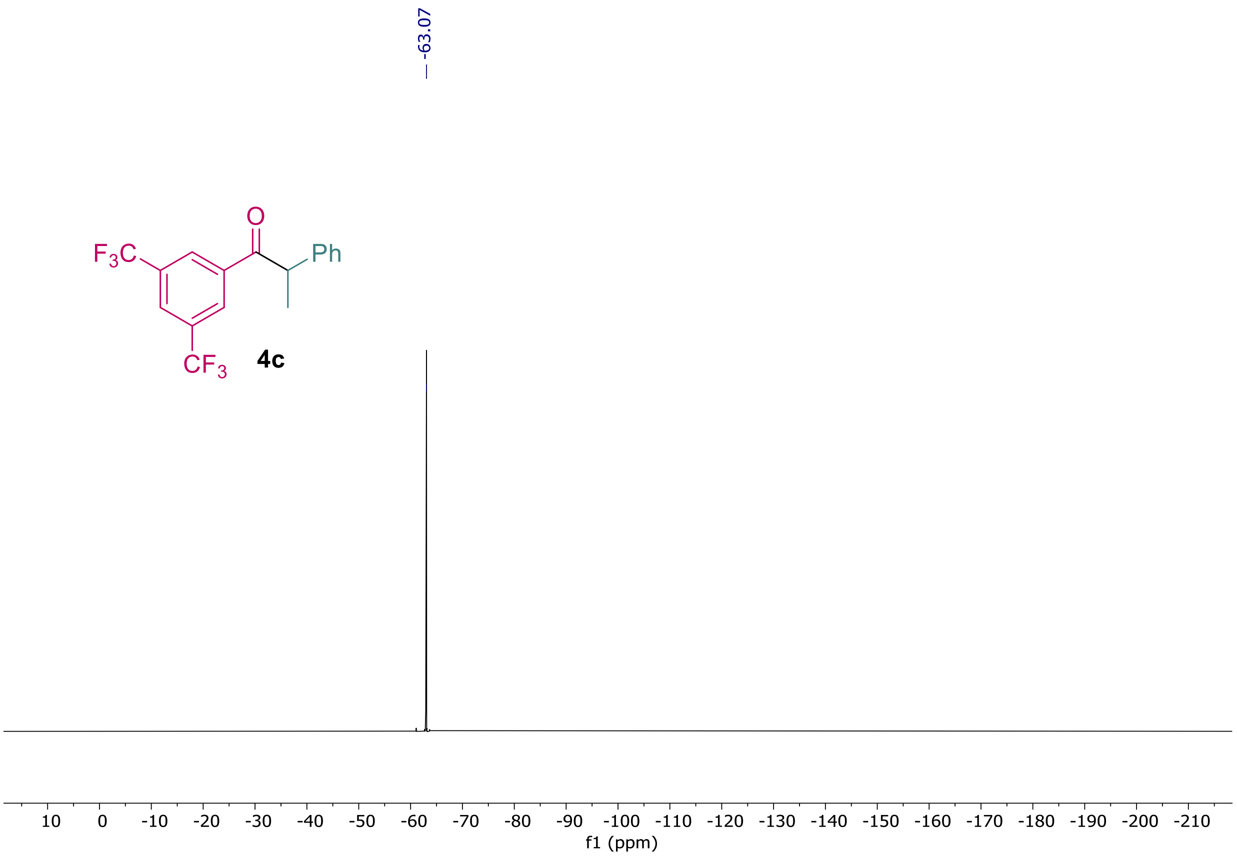

Figure S68: $^{19}F\{^{1}H\}$ NMR spectrum of **4c** in $CDCl_3$ (282 MHz)

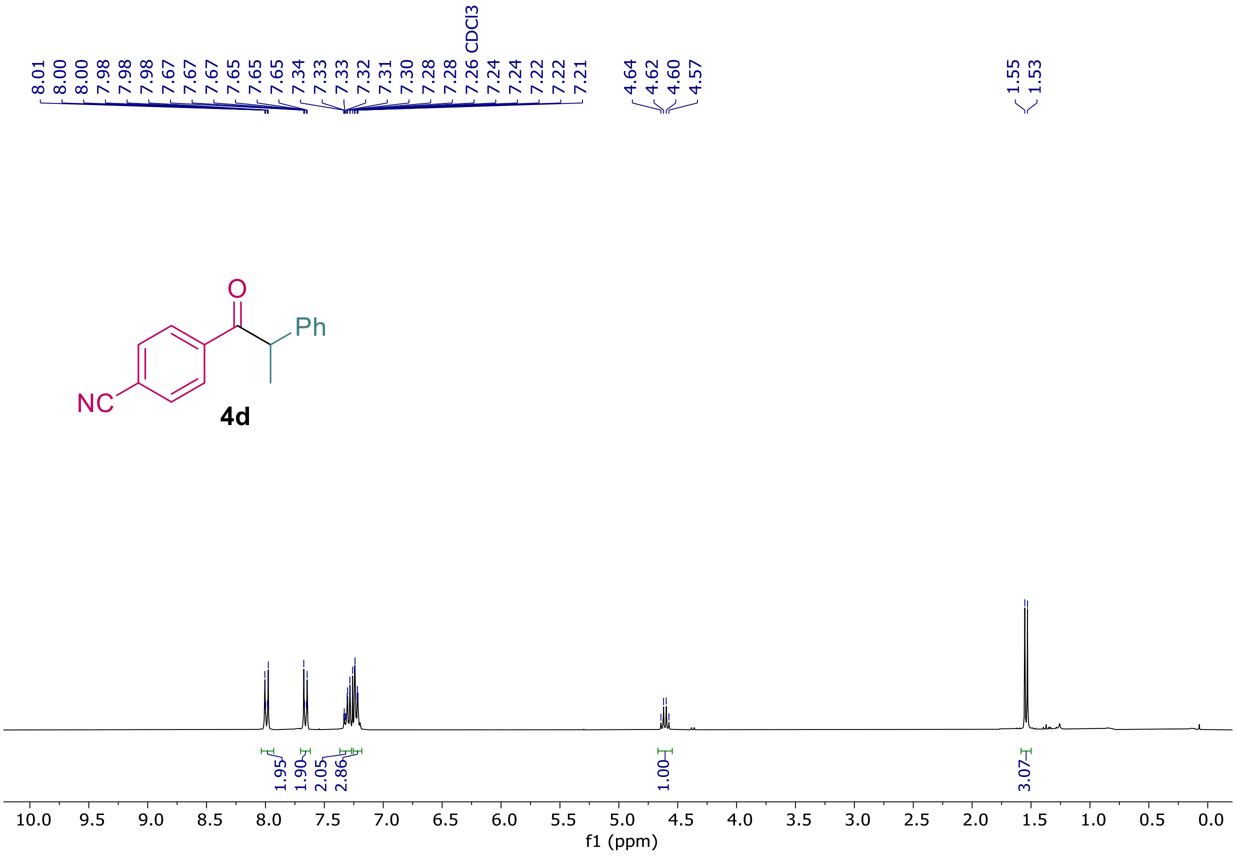

Figure S69: ^{1}H NMR spectrum of **4d** in $CDCl_3$ (300 MHz)

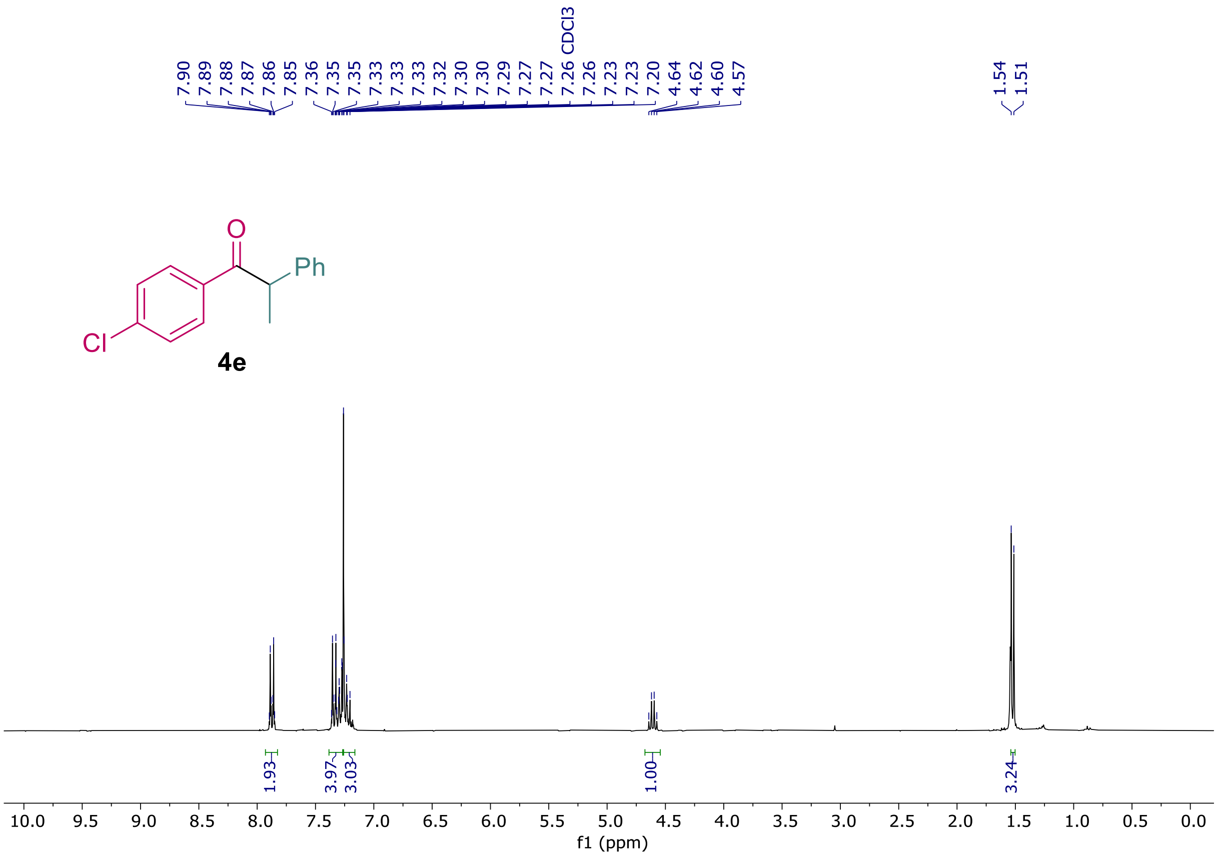

Figure S70: ^{1}H NMR spectrum of **4e** in $CDCl_3$ (300 MHz)

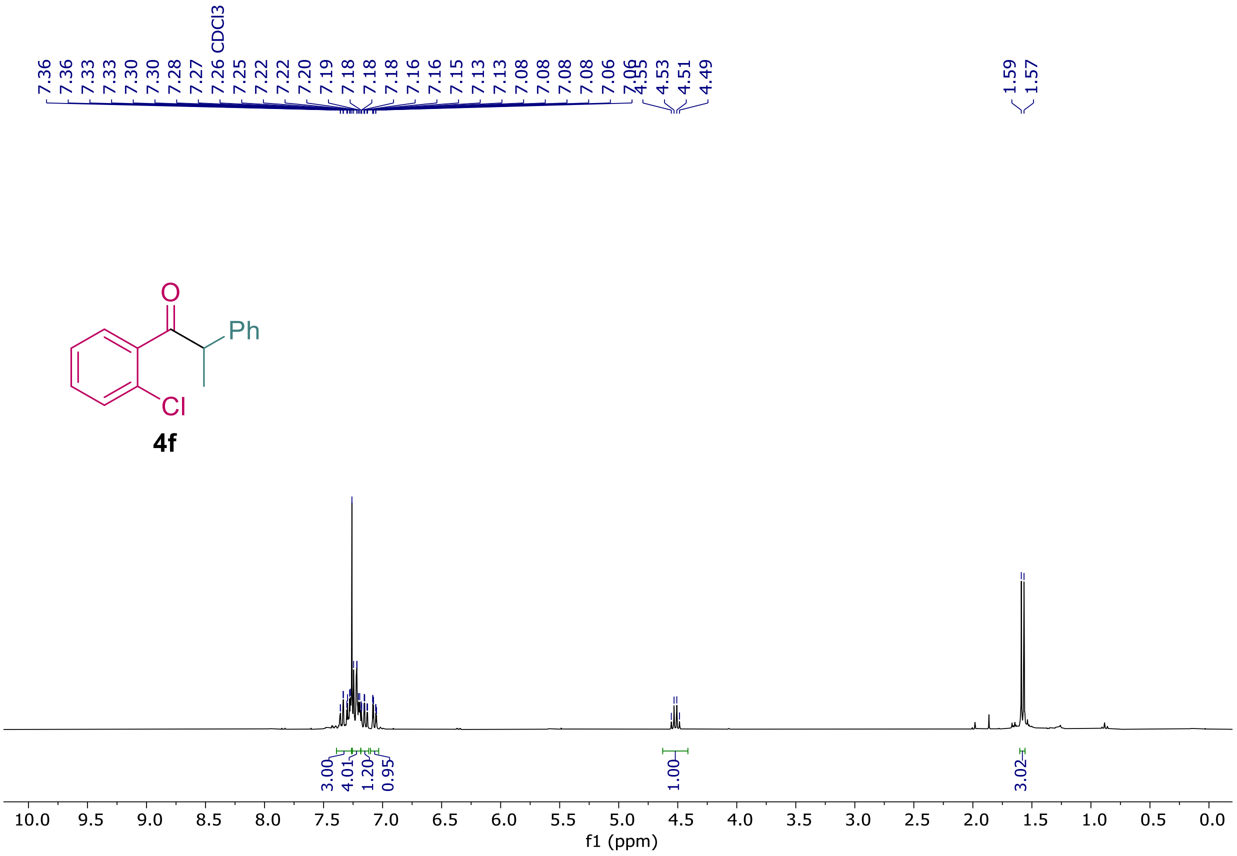

Figure S71: ^{1}H NMR spectrum of **4f** in $CDCl_3$ (300 MHz)

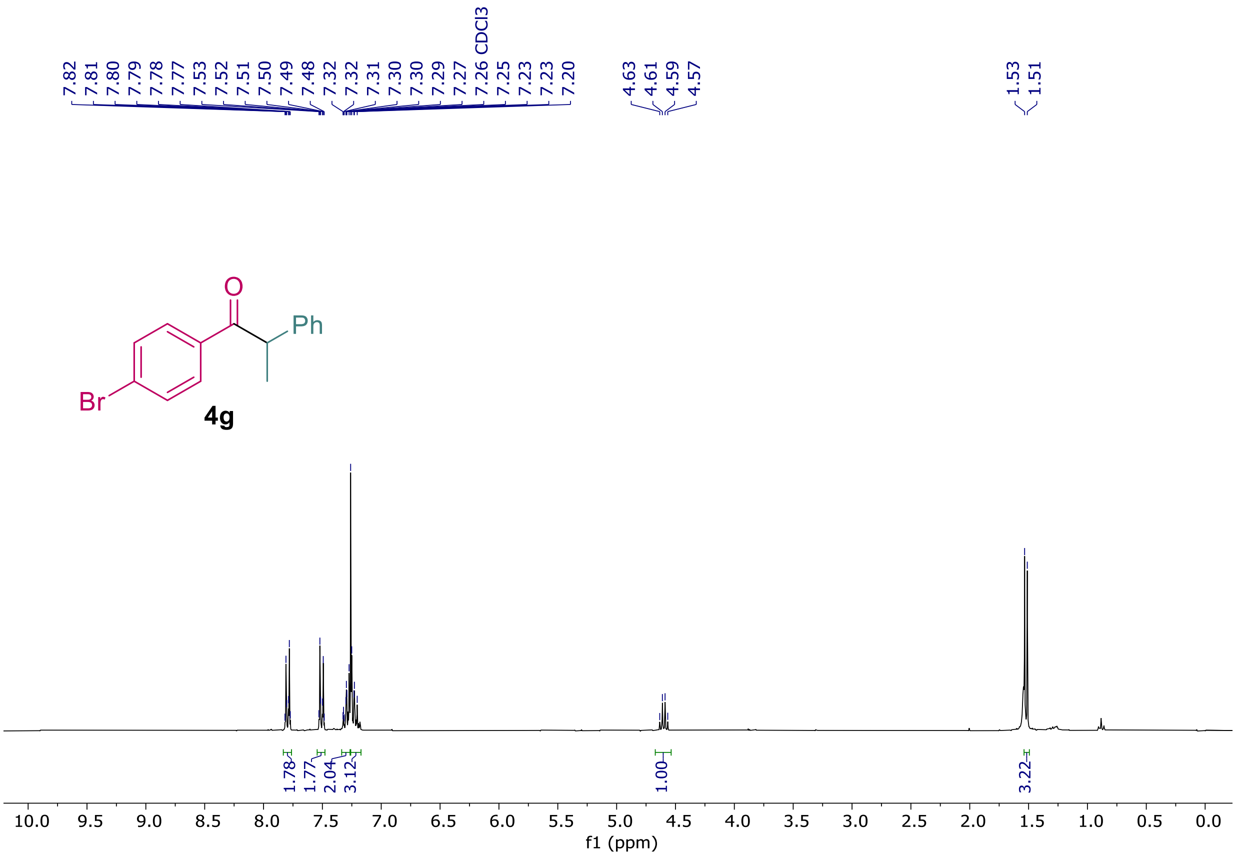

Figure S72: ^{1}H NMR spectrum of **4g** in $CDCl_3$ (300 MHz)

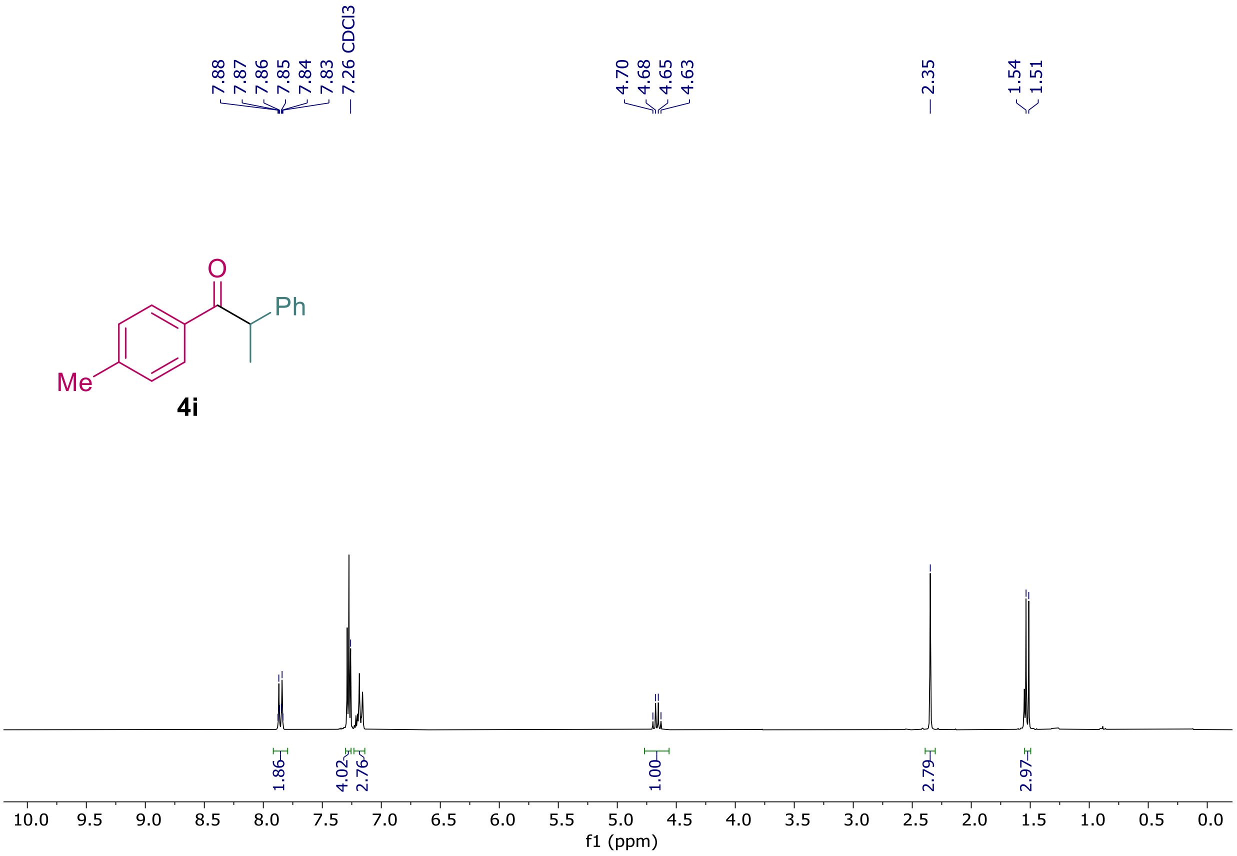

Figure S73: ^{1}H NMR spectrum of **4i** in $CDCl_3$ (300 MHz)

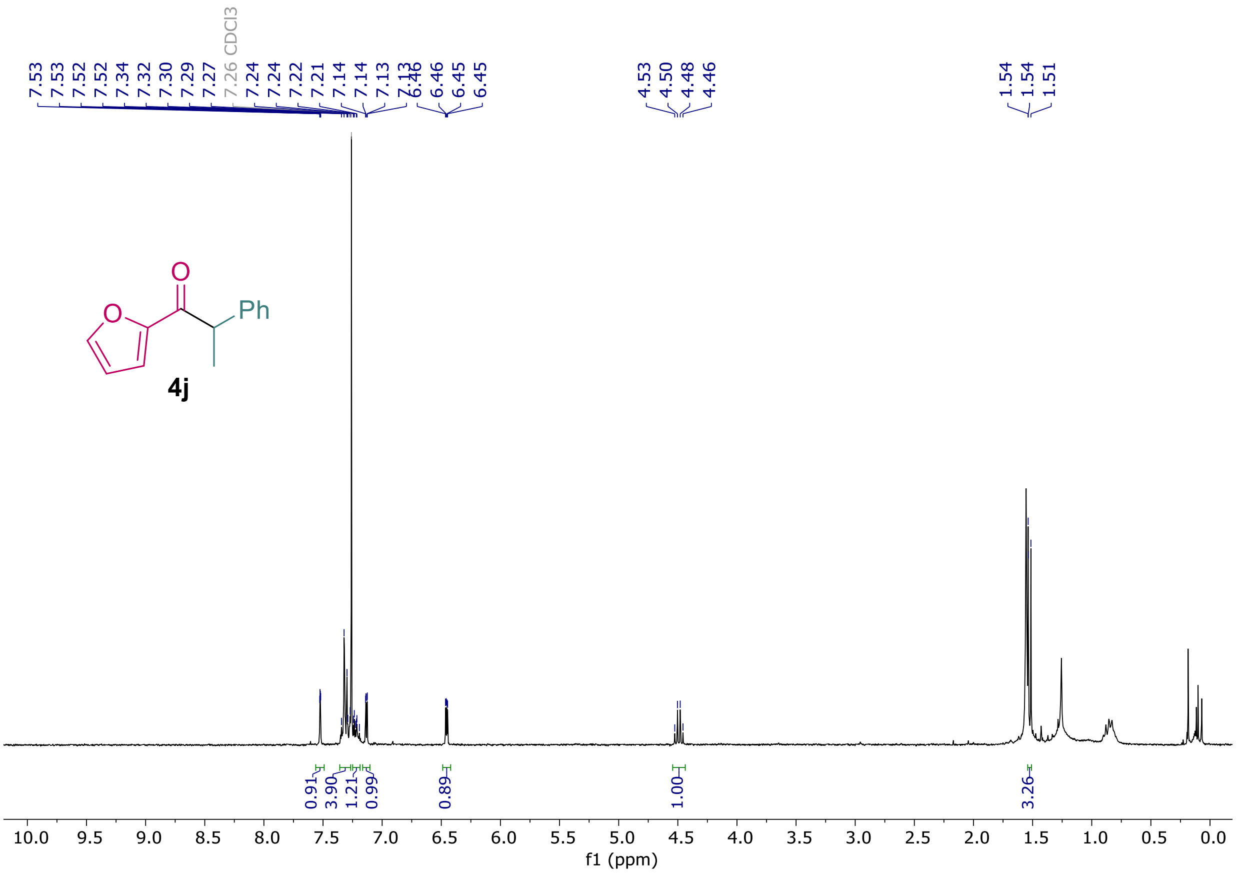

Figure S74: ^{1}H NMR spectrum of **4j** in $CDCl_3$ (300 MHz)

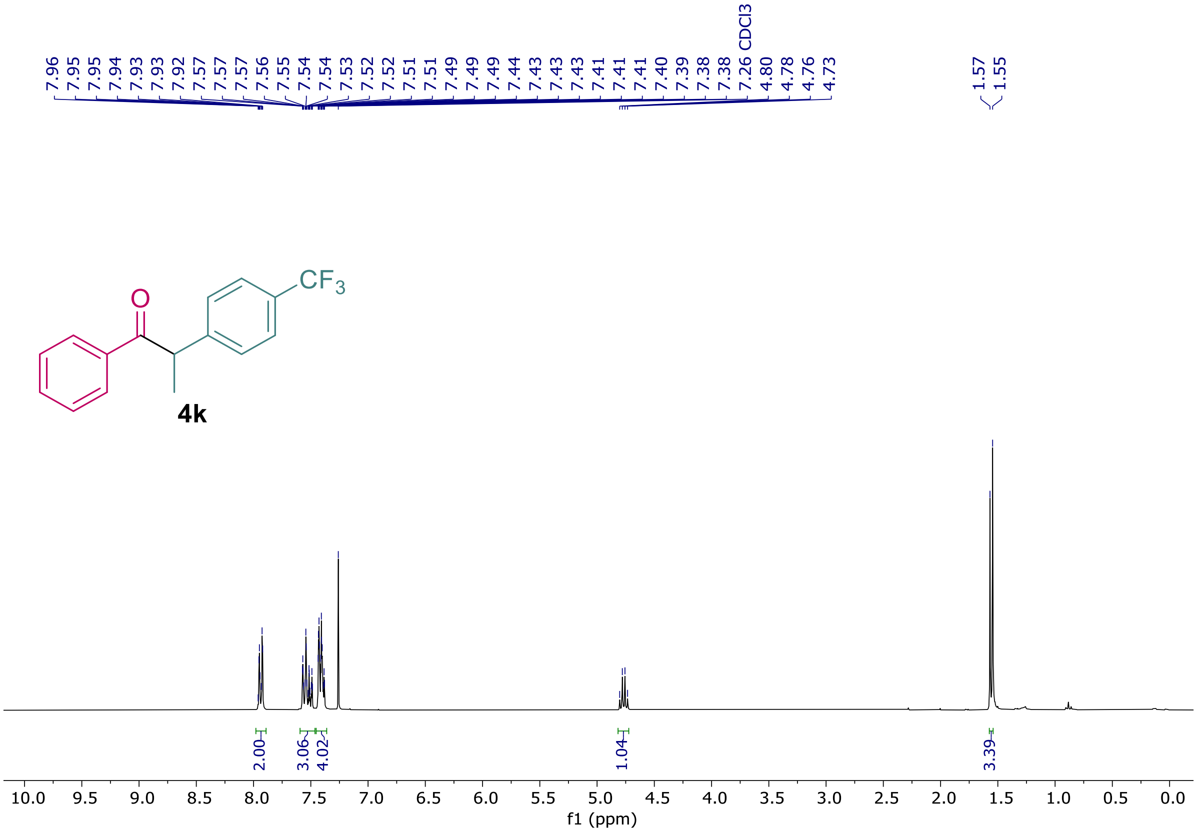

Figure S75: ^{1}H NMR spectrum of **4k** in $CDCl_3$ (300 MHz)

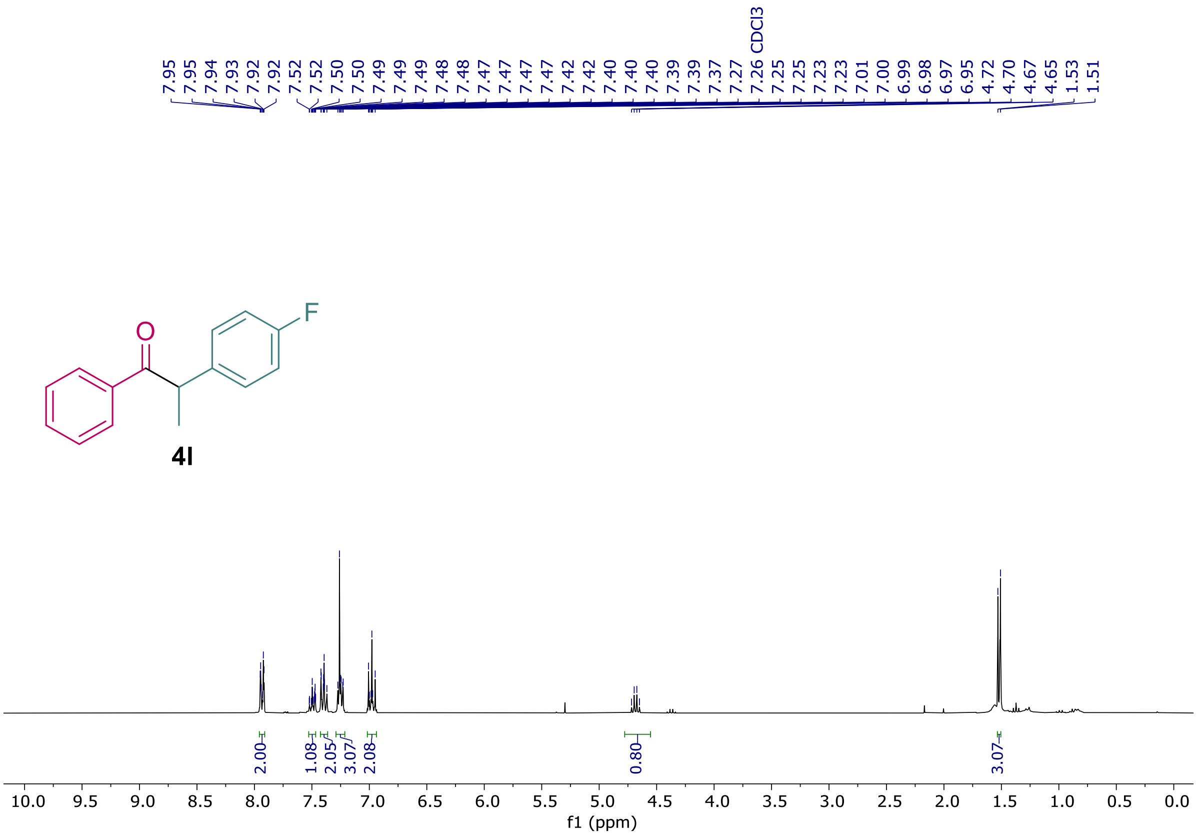

Figure S76: ^{1}H NMR spectrum of **4l** in $CDCl_3$ (300 MHz)

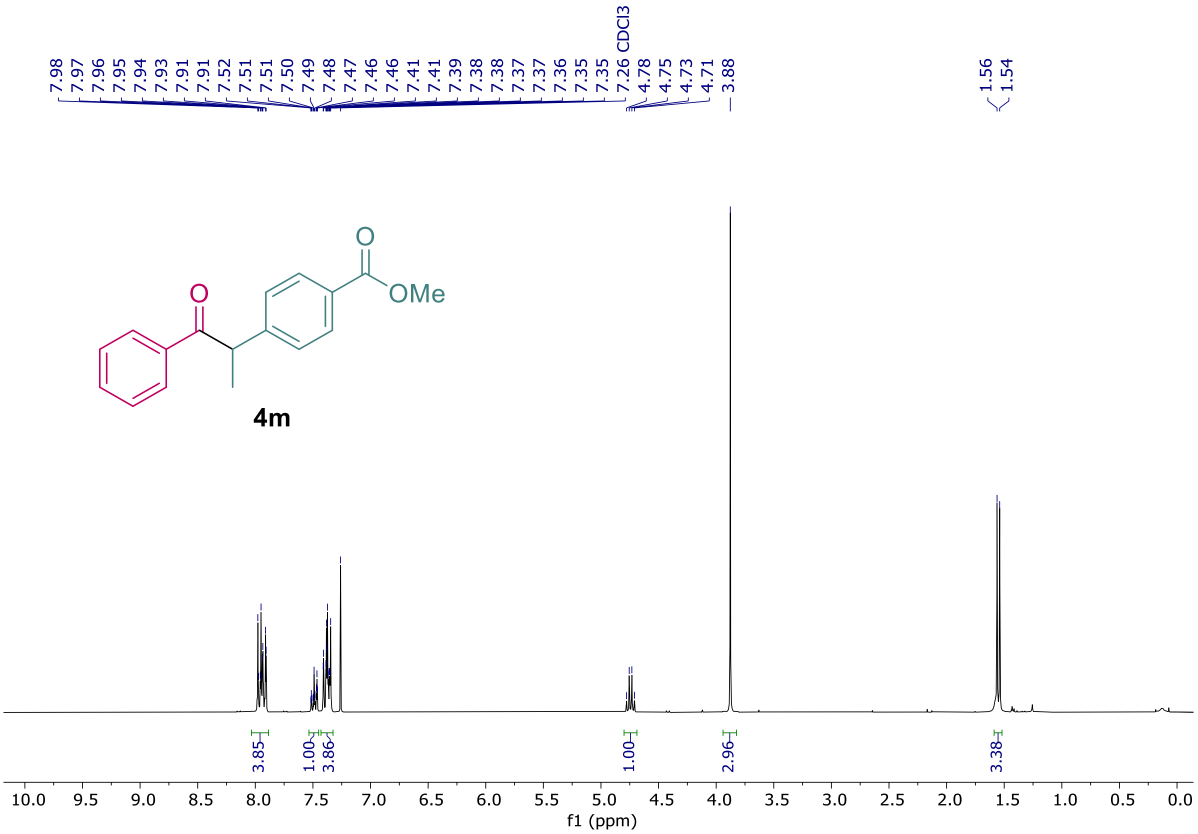

Figure S77: ^{1}H NMR spectrum of **4m** in $CDCl_3$ (300 MHz)

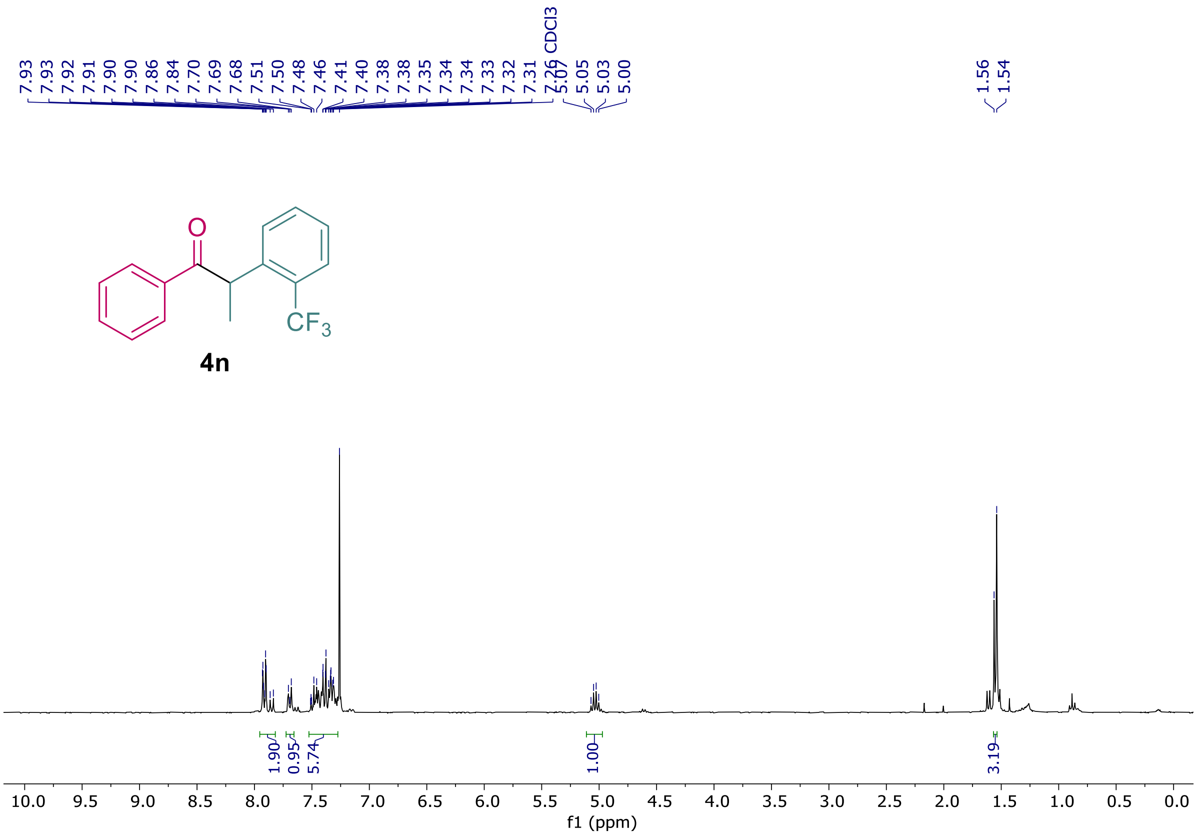

Figure S78: ^{1}H NMR spectrum of **4n** in $CDCl_3$ (300 MHz)

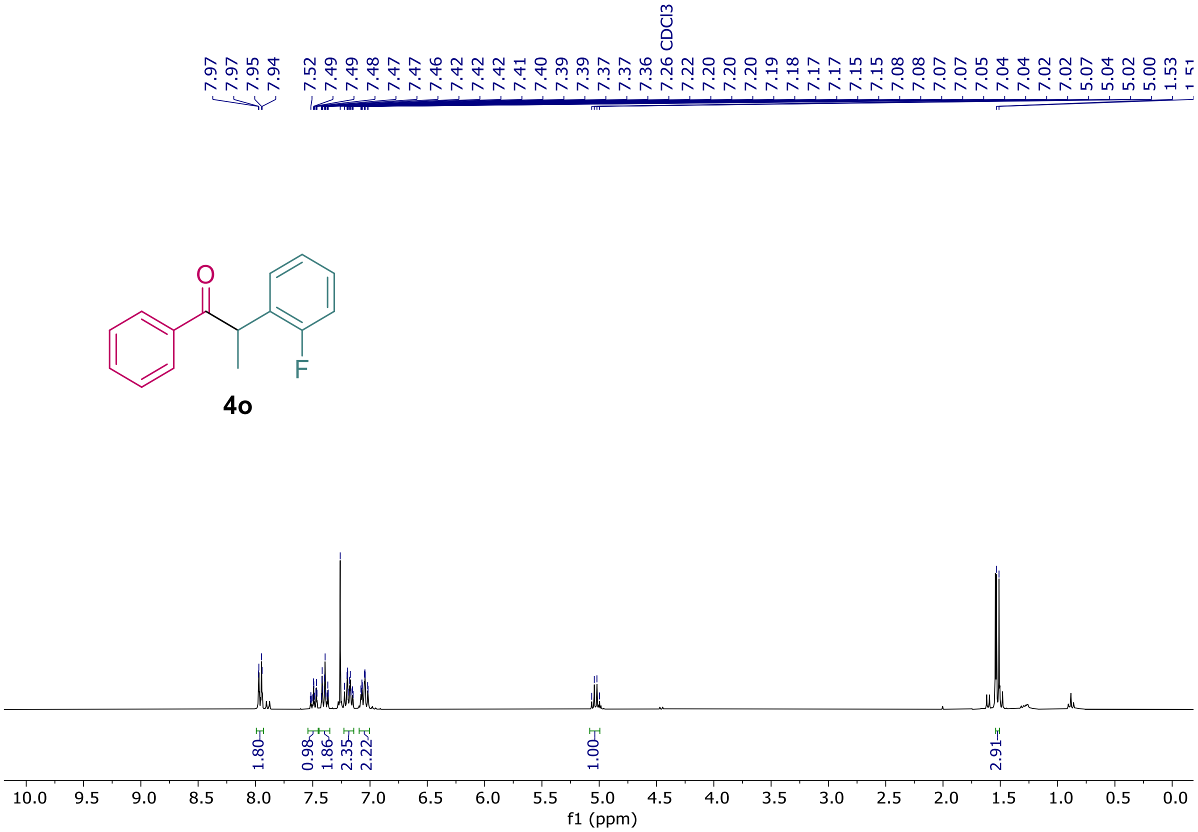

Figure S79: ^{1}H NMR spectrum of **4o** in $CDCl_3$ (300 MHz)

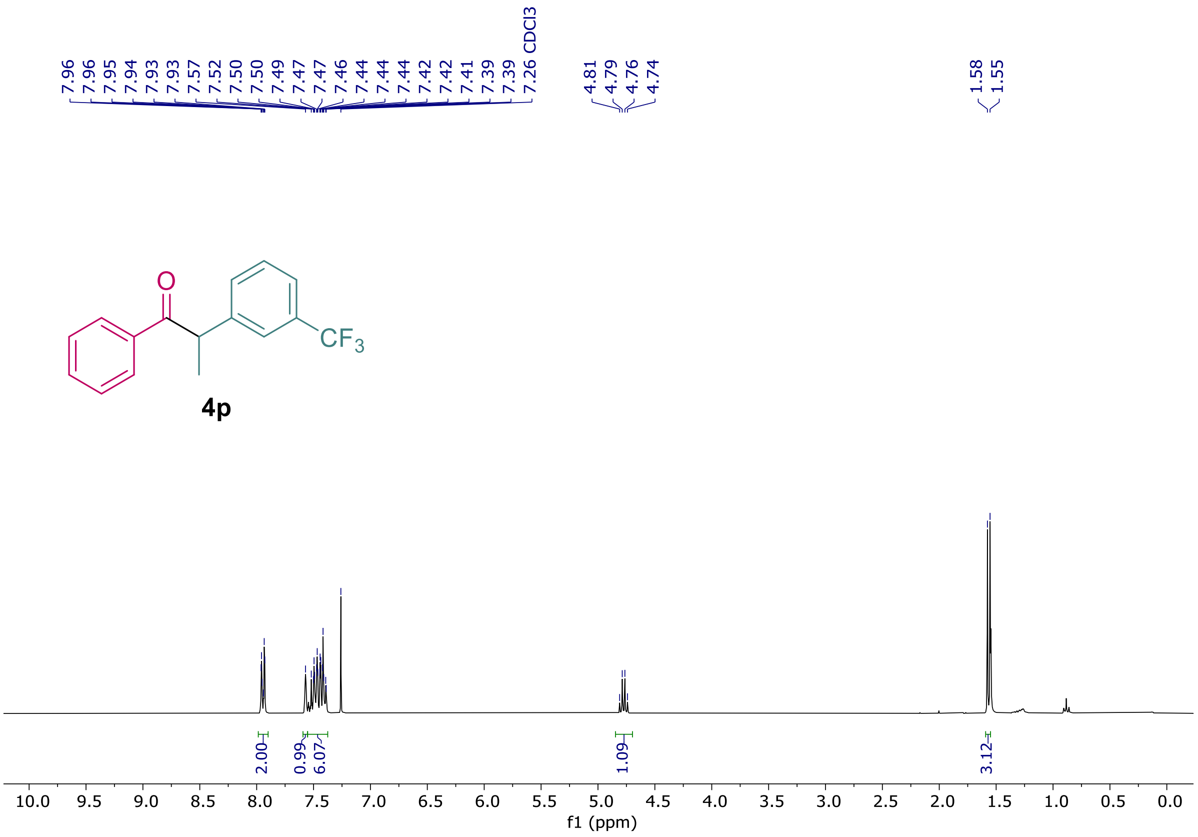

Figure S80: ^{1}H NMR spectrum of **4p** in $CDCl_3$ (300 MHz)

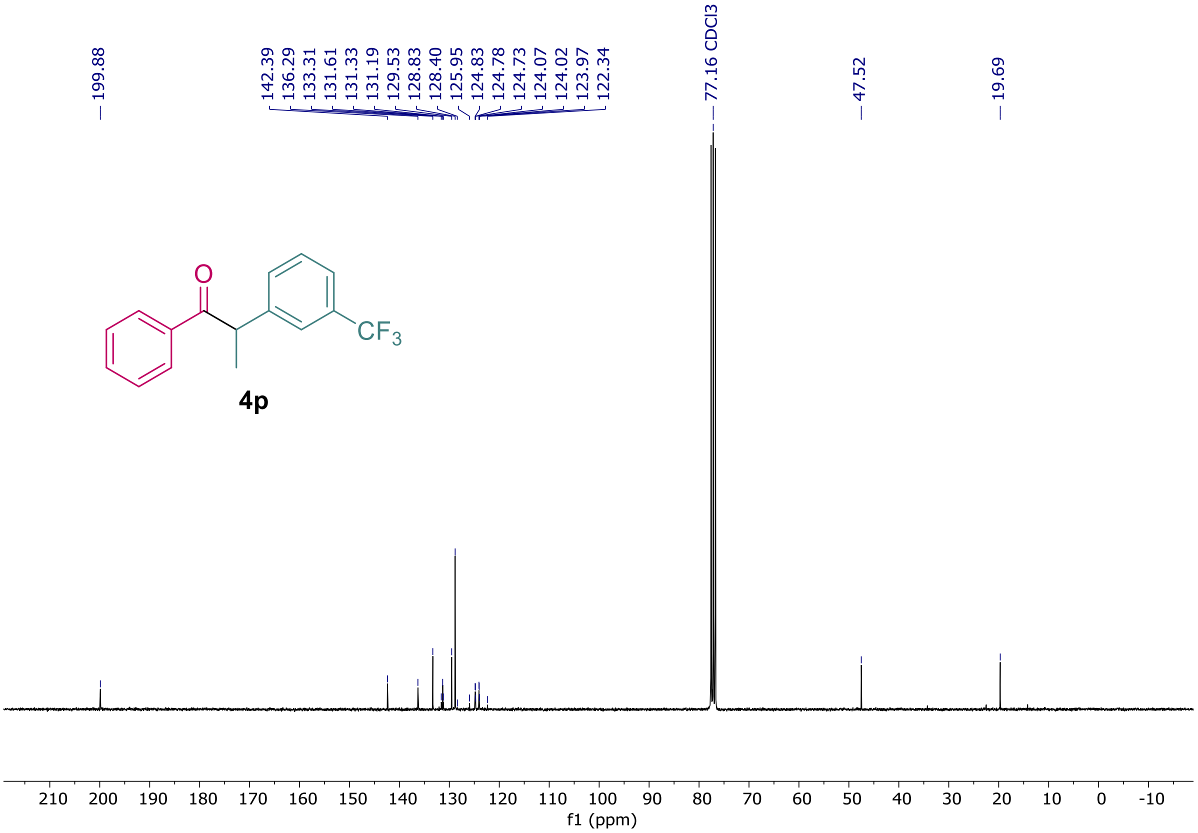

Figure S81: ^{13}C{^{1}H} NMR spectrum of **4p** in $CDCl_3$ (75 MHz)

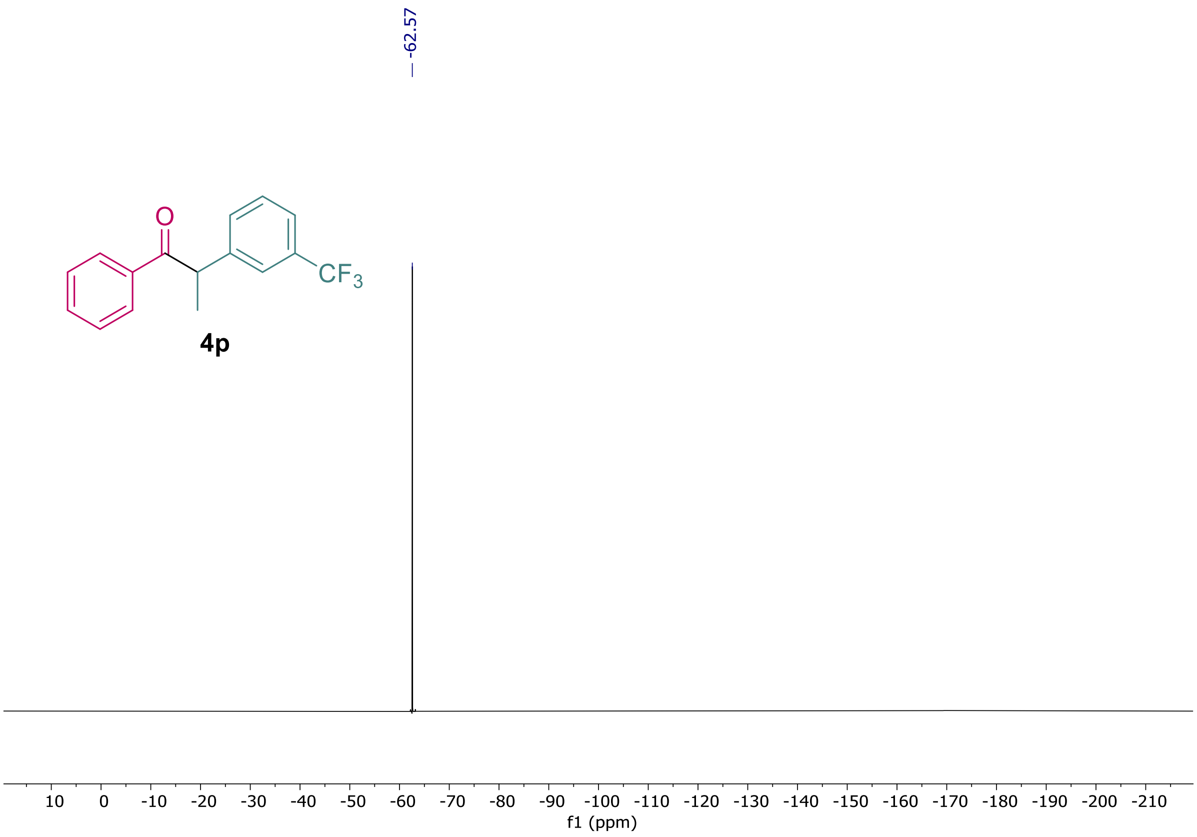

Figure S82: $^{19}F\{^1H\}$ NMR spectrum of **4p** in $CDCl_3$ (282 MHz)

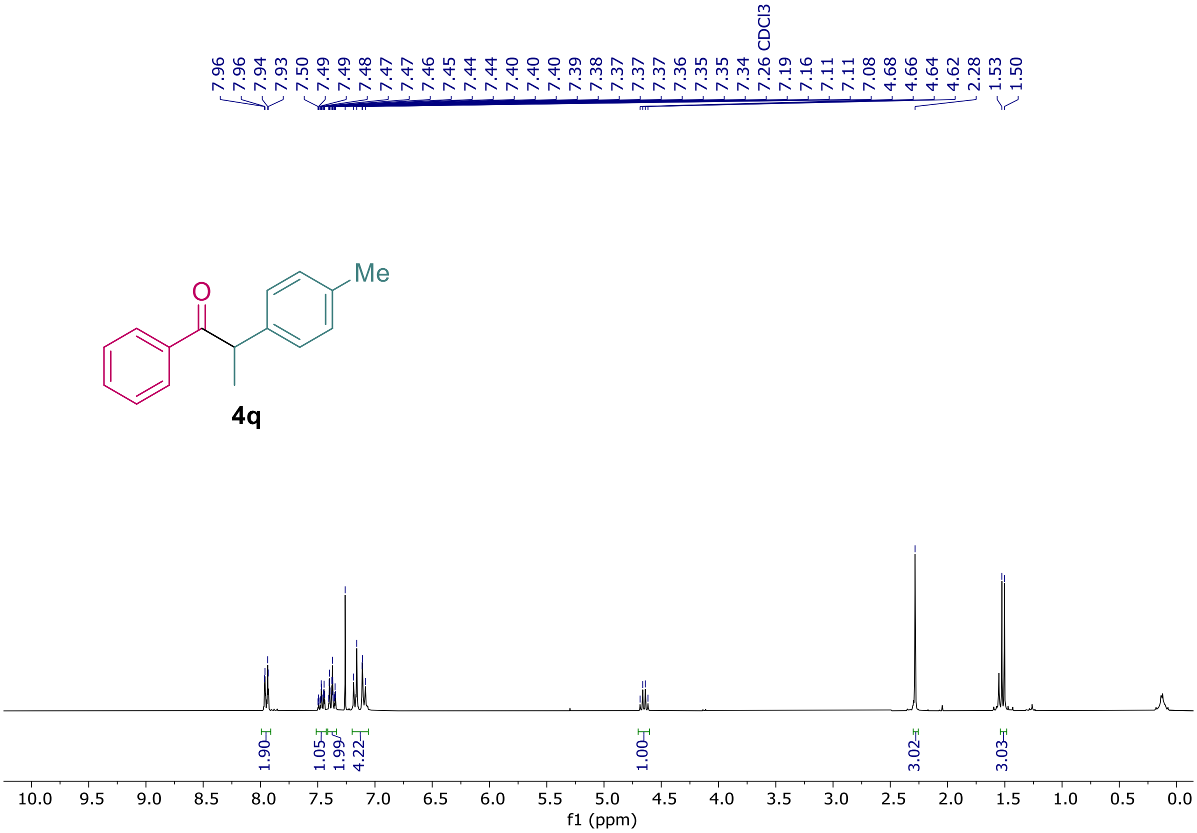

Figure S83: ^1H NMR spectrum of **3q** in $CDCl_3$ (300 MHz)

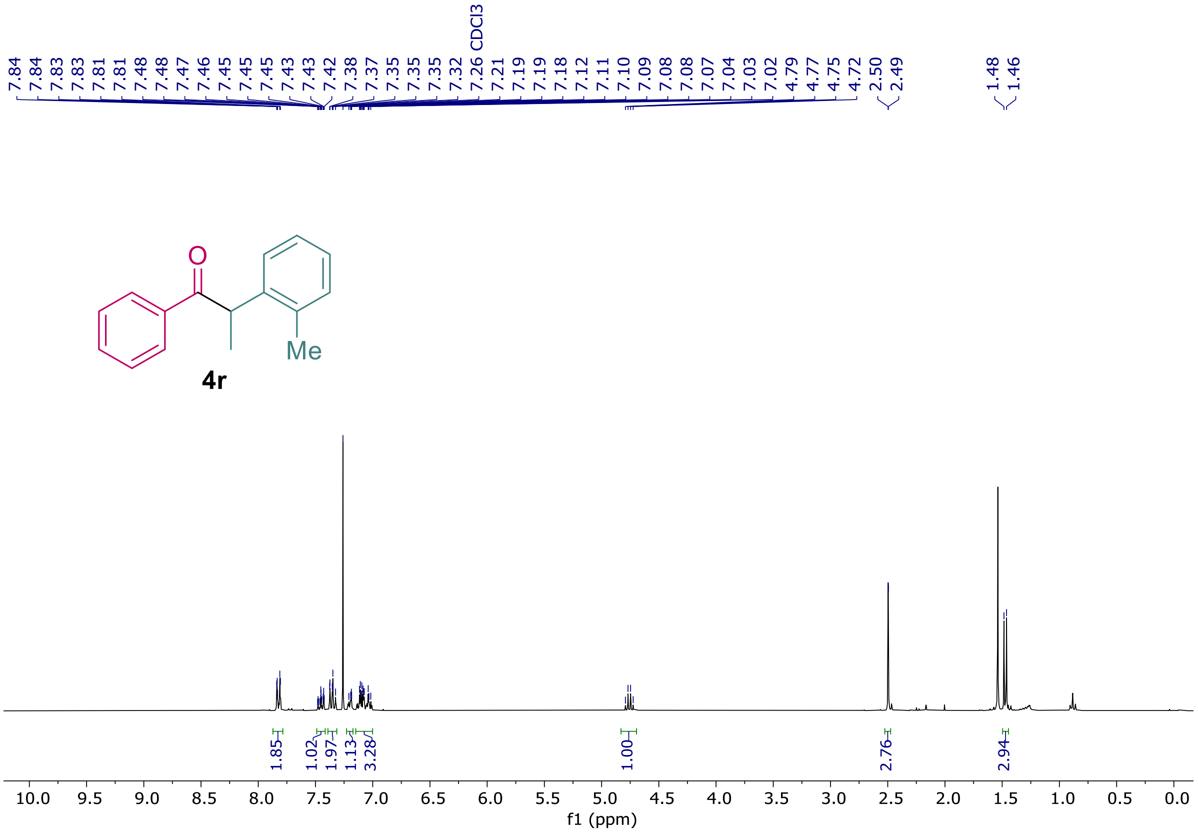

Figure S84: ^{1}H NMR spectrum of **4r** in $CDCl_3$ (300 MHz)

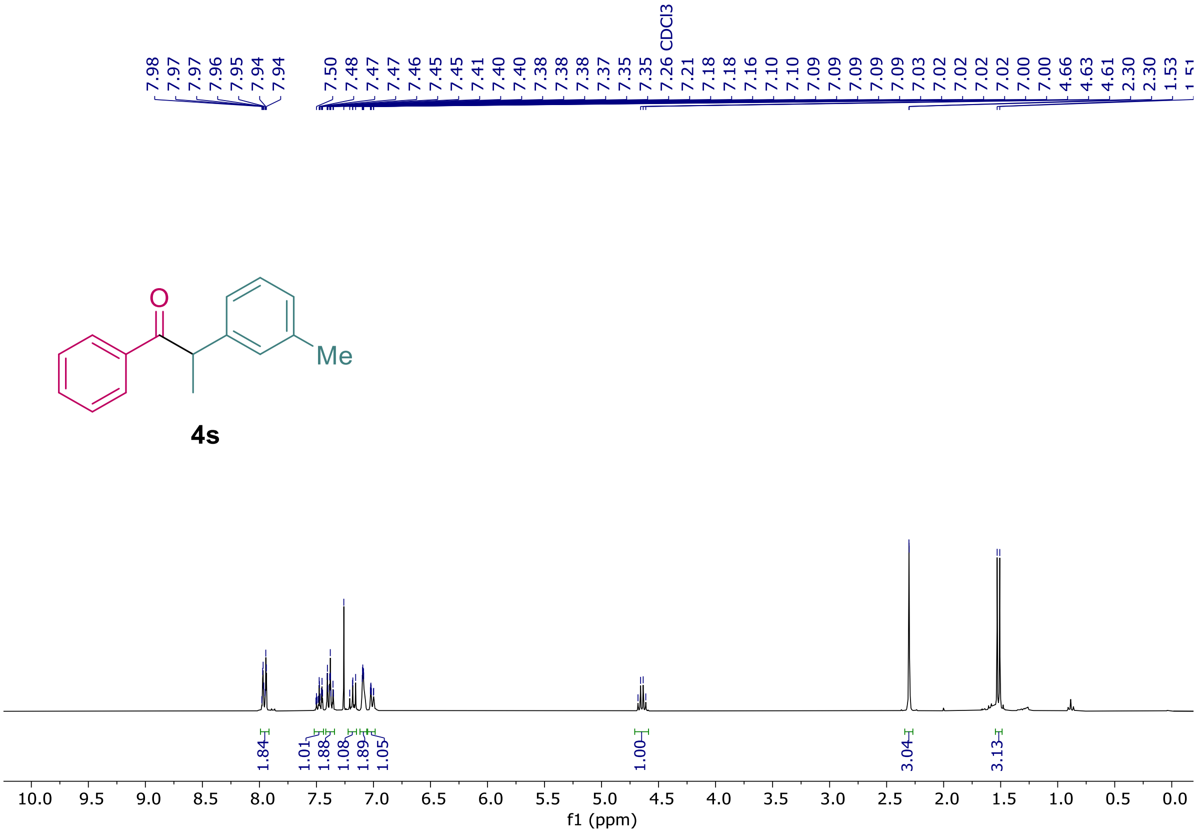

Figure S85: ^{1}H NMR spectrum of **4s** in $CDCl_3$ (300 MHz)

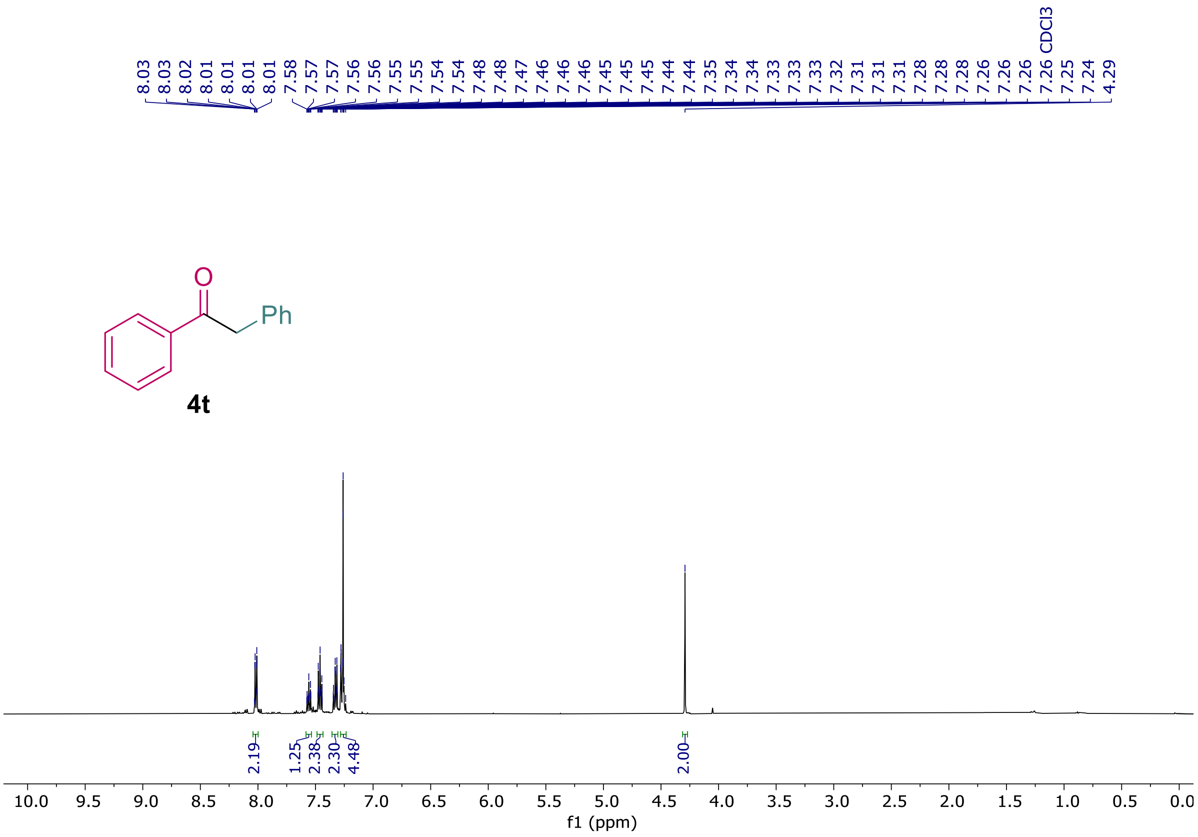

Figure S86: ^{1}H NMR spectrum of **4t** in $CDCl_3$ (500 MHz)

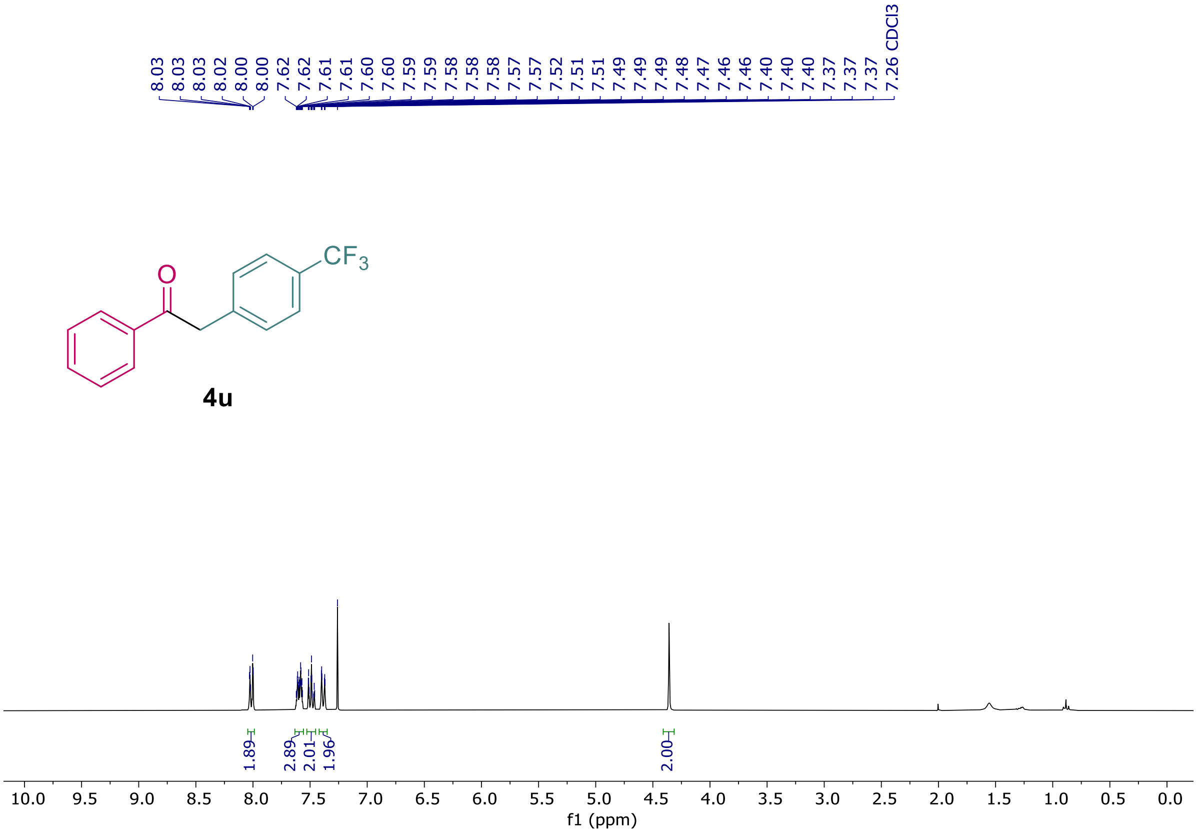

Figure S87: ^{1}H NMR spectrum of **4u** in $CDCl_3$ (300 MHz)

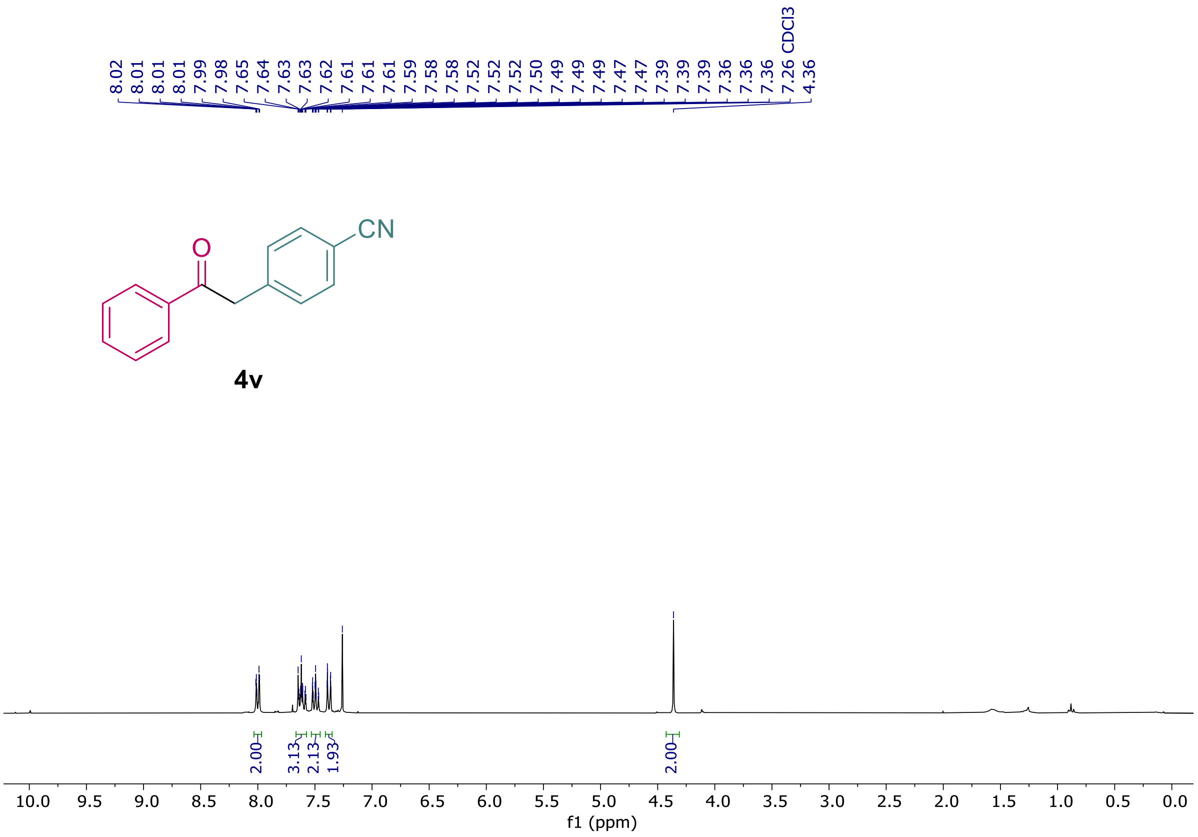

Figure S88: ^{1}H NMR spectrum of **4v** in $CDCl_3$ (300 MHz)

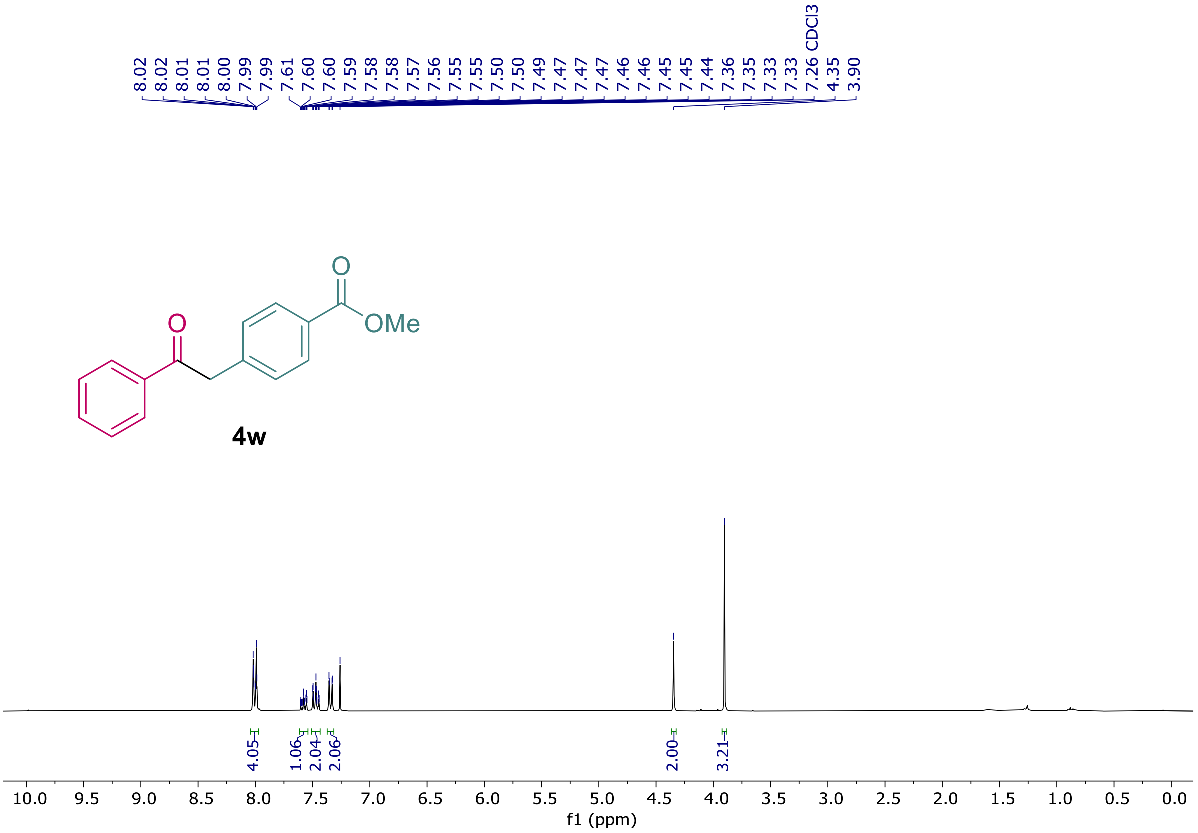

Figure S89: ^{1}H NMR spectrum of **4w** in $CDCl_3$ (300 MHz)

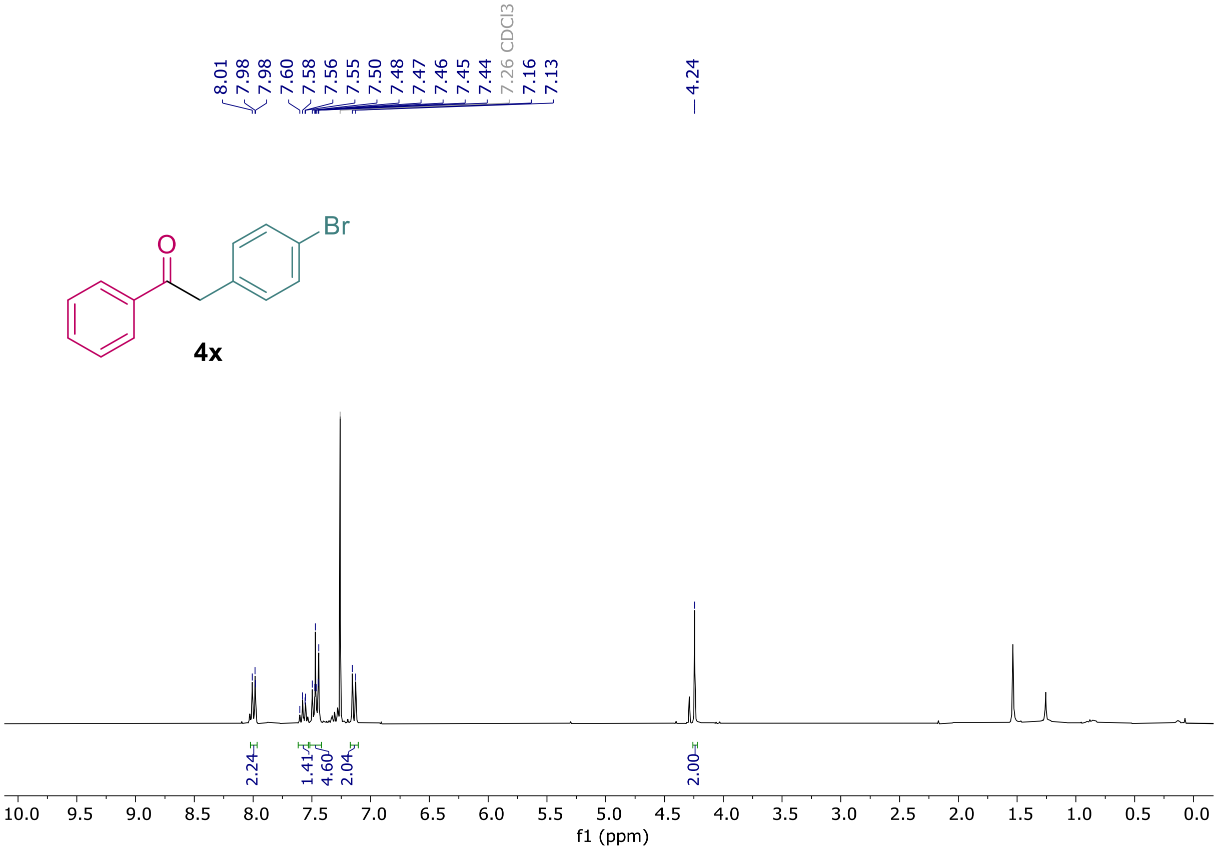

Figure S90: ^{1}H NMR spectrum of **4x** in $CDCl_3$ (300 MHz)

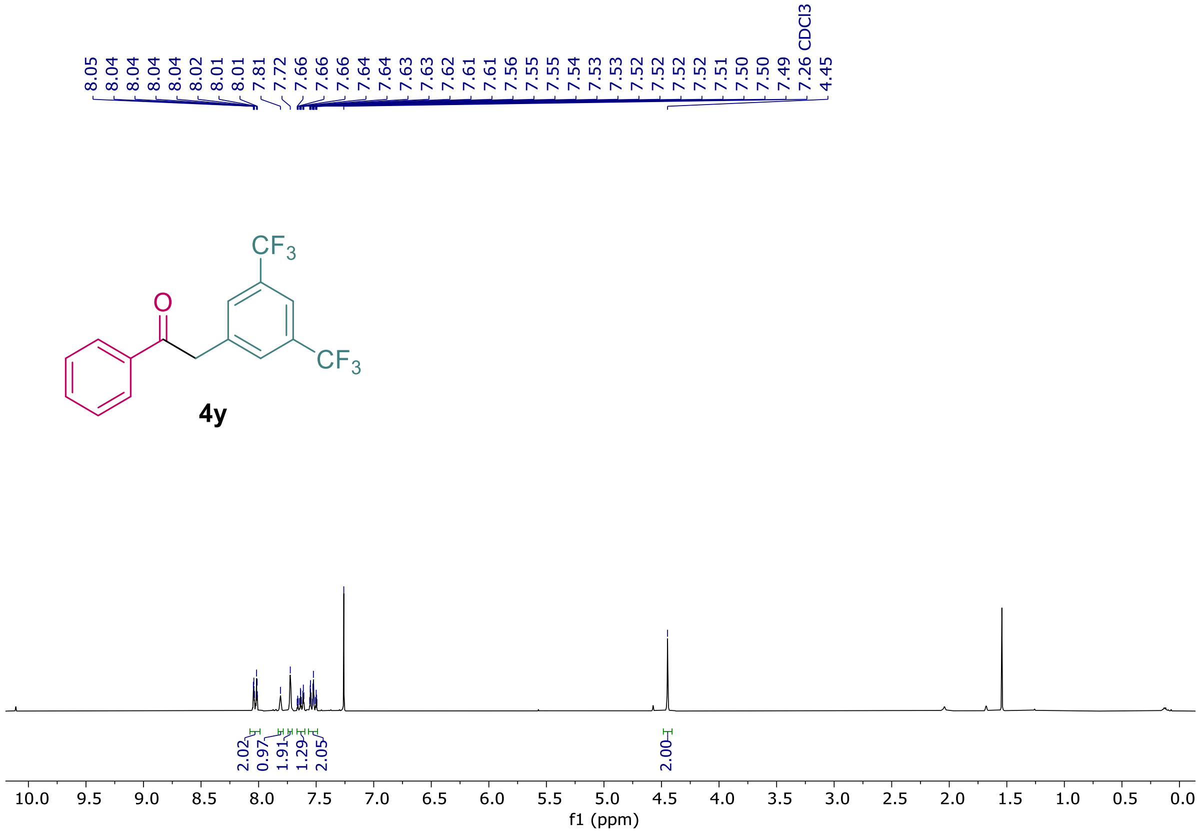

Figure S91: ^{1}H NMR spectrum of **4y** in $CDCl_3$ (300 MHz)

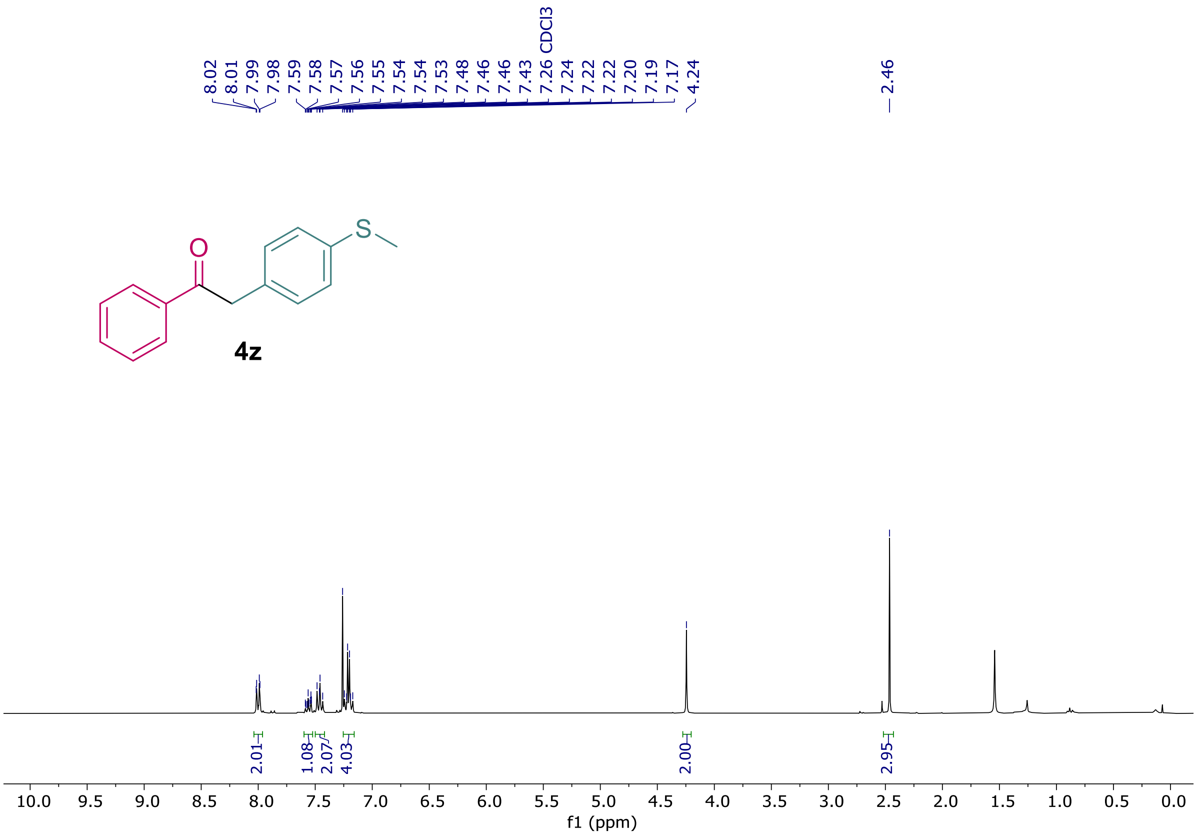

Figure S92: ^{1}H NMR spectrum of **4z** in $CDCl_3$ (300 MHz)

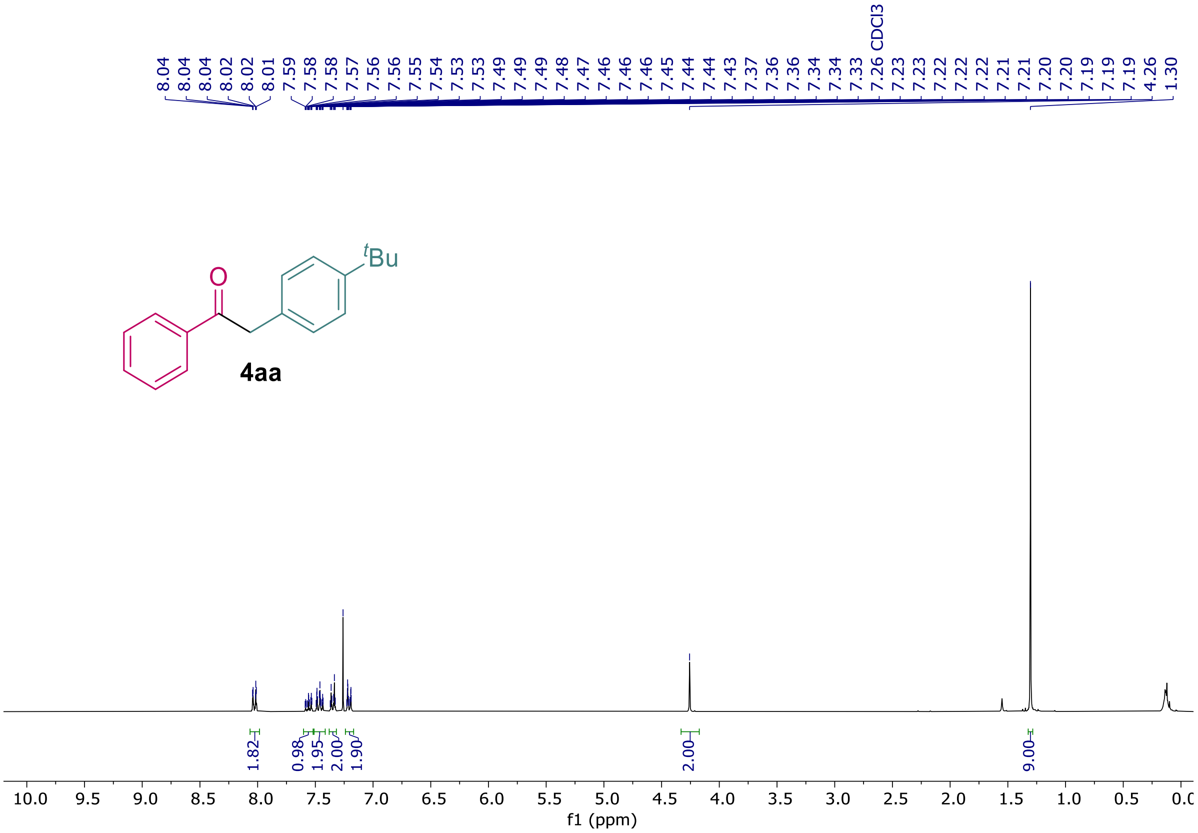

Figure S93: ^{1}H NMR spectrum of **4aa** in $CDCl_3$ (300 MHz)

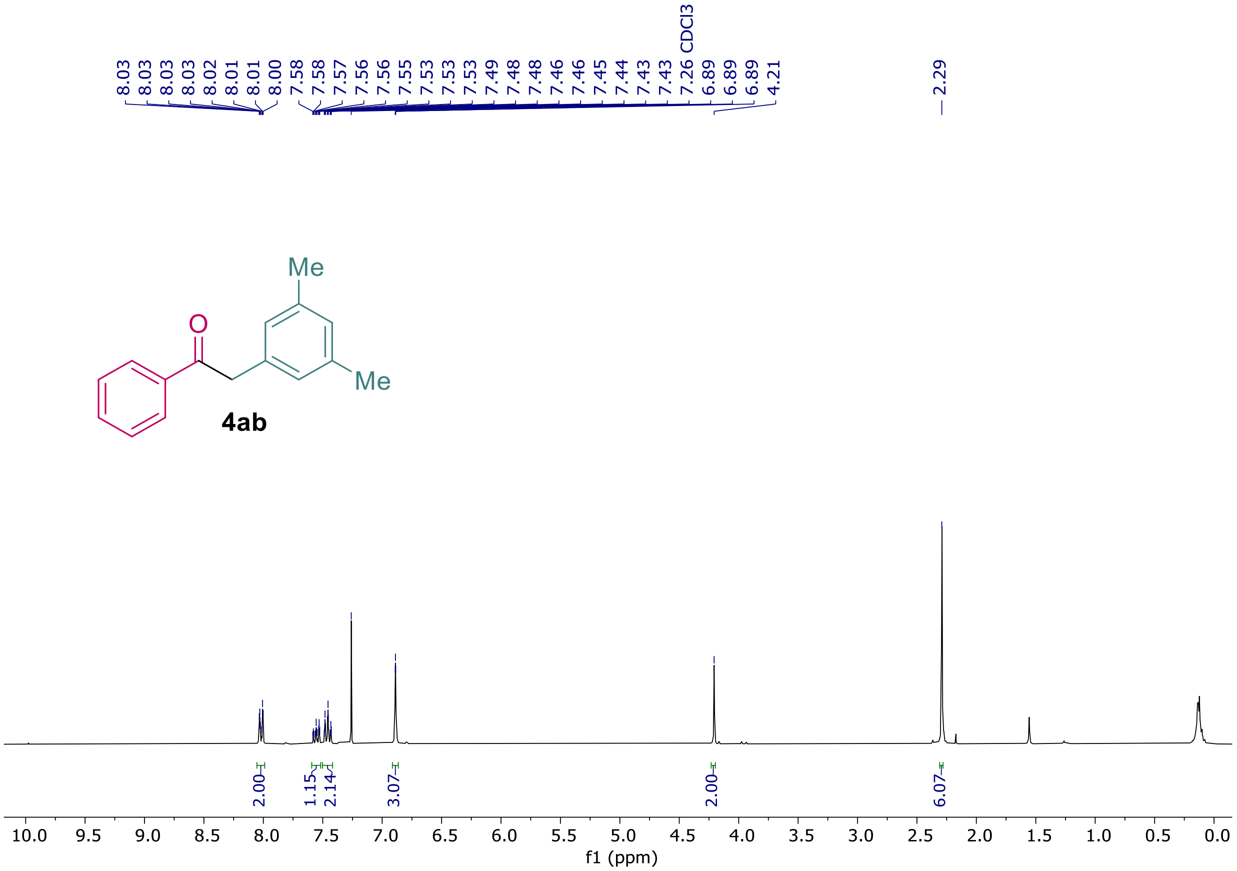

Figure S94: ^{1}H NMR spectrum of **4ab** in $CDCl_3$ (300 MHz)

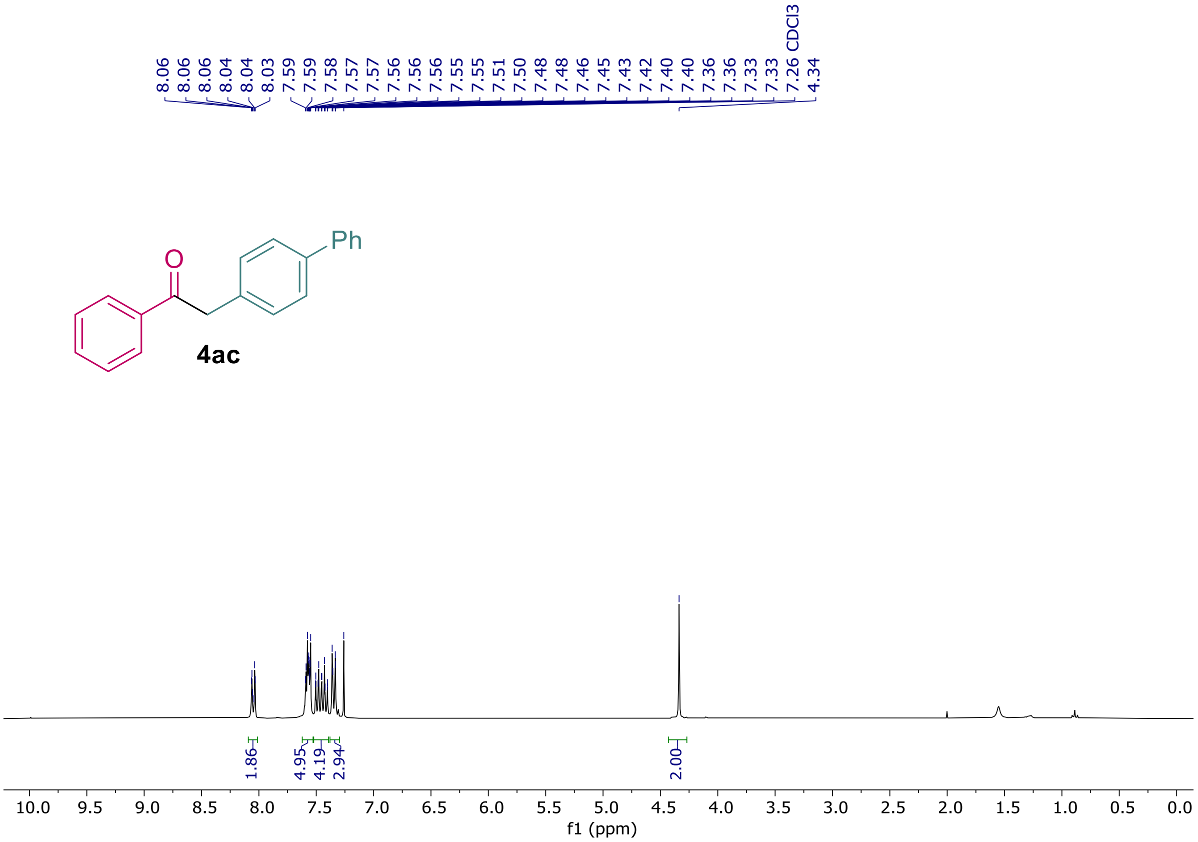

Figure S95: ^{1}H NMR spectrum of **4ac** in $CDCl_3$ (300 MHz)

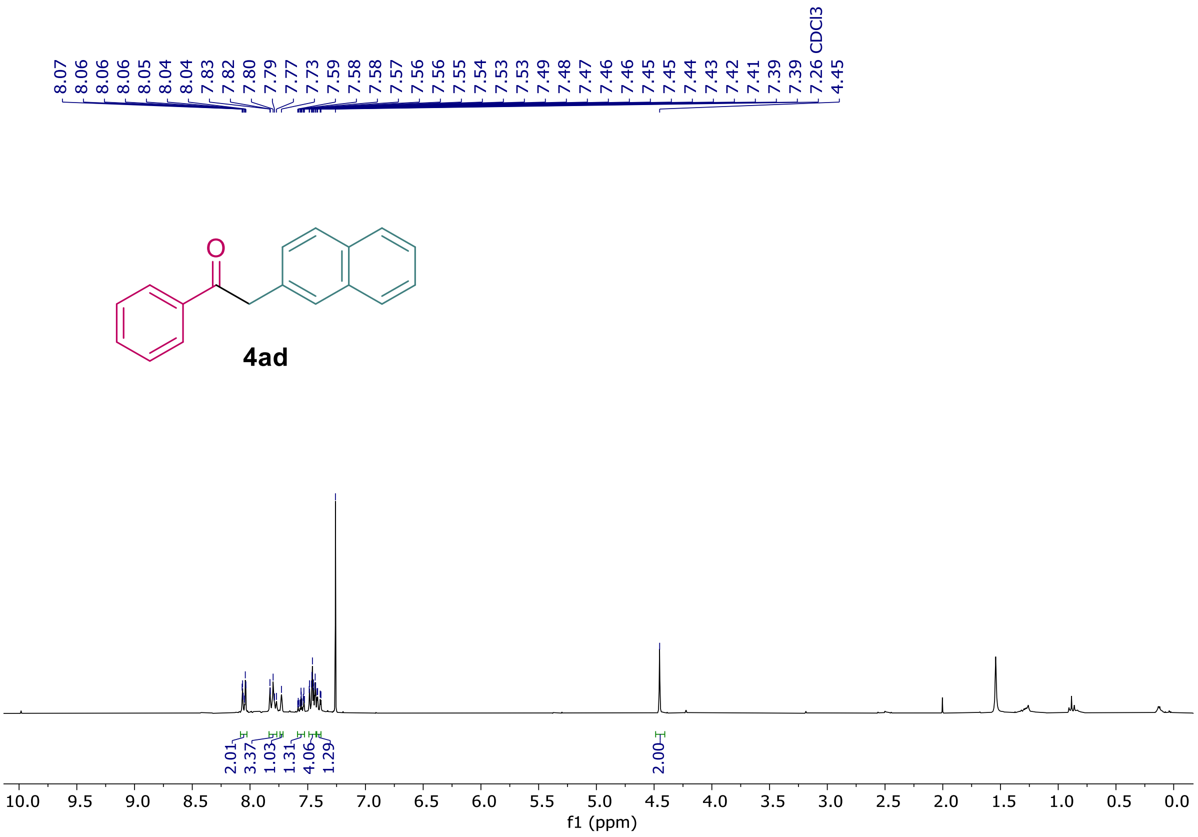

Figure S96: ^{1}H NMR spectrum of **4ad** in $CDCl_3$ (300 MHz)

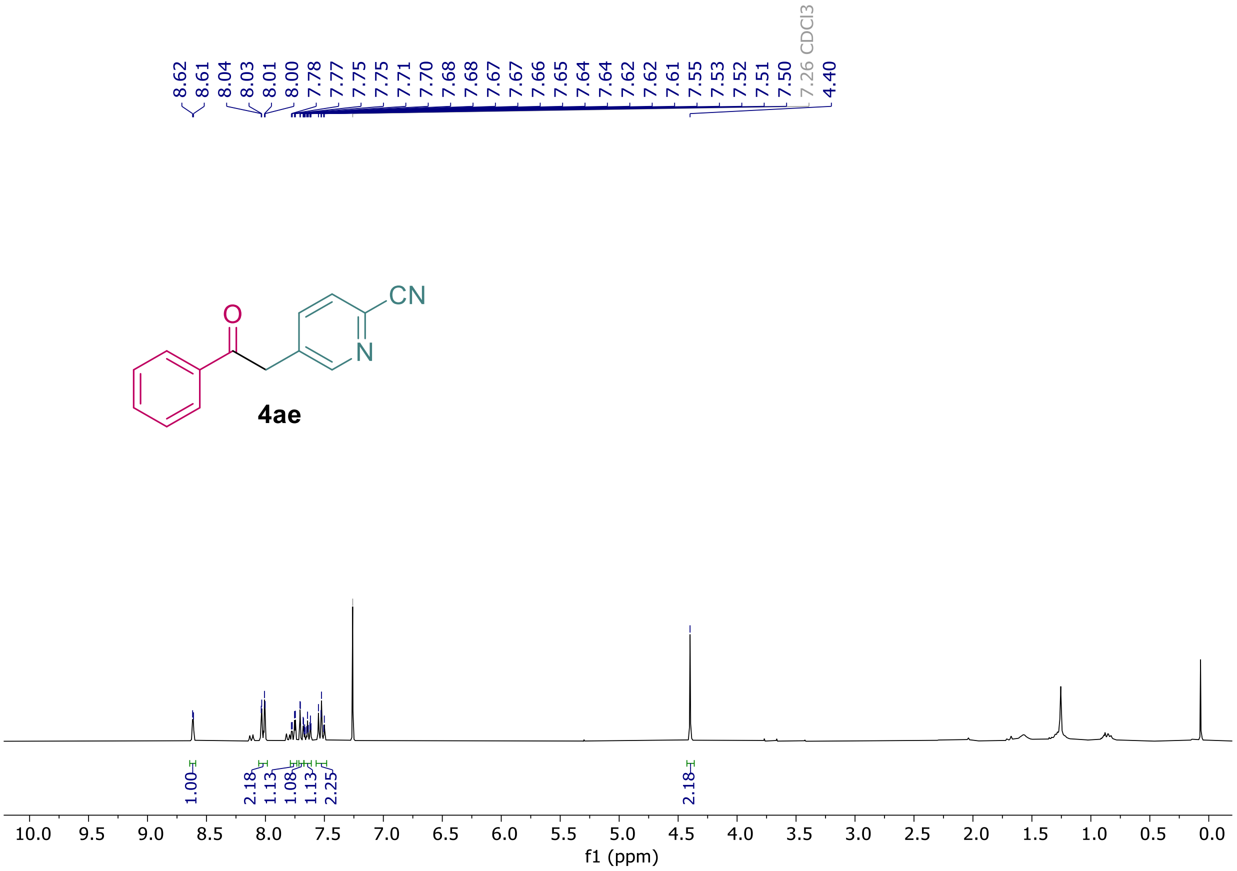

Figure S97: ^{1}H NMR spectrum of **4ae** in $CDCl_3$ (300 MHz)

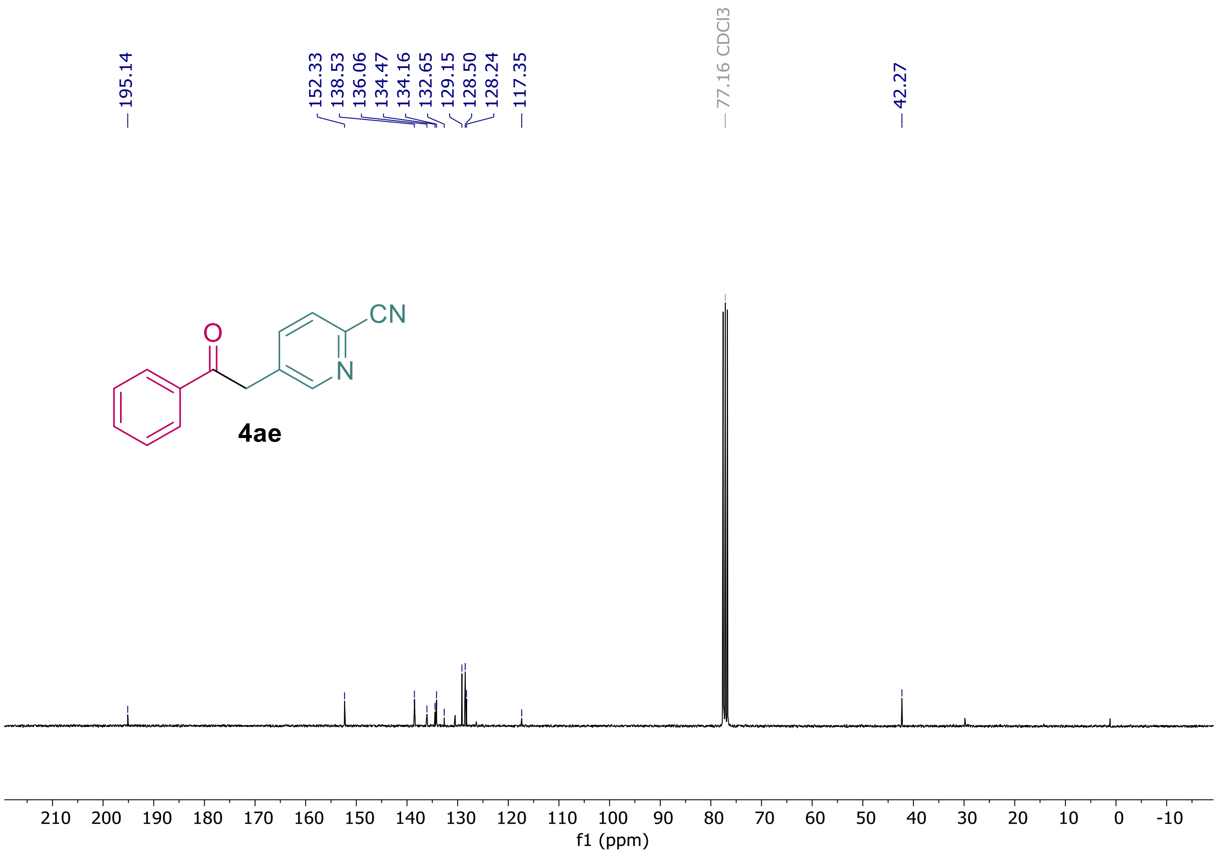

Figure S98: $^{13}C\{^1H\}$ NMR spectrum of **4ae** in $CDCl_3$ (75 MHz)

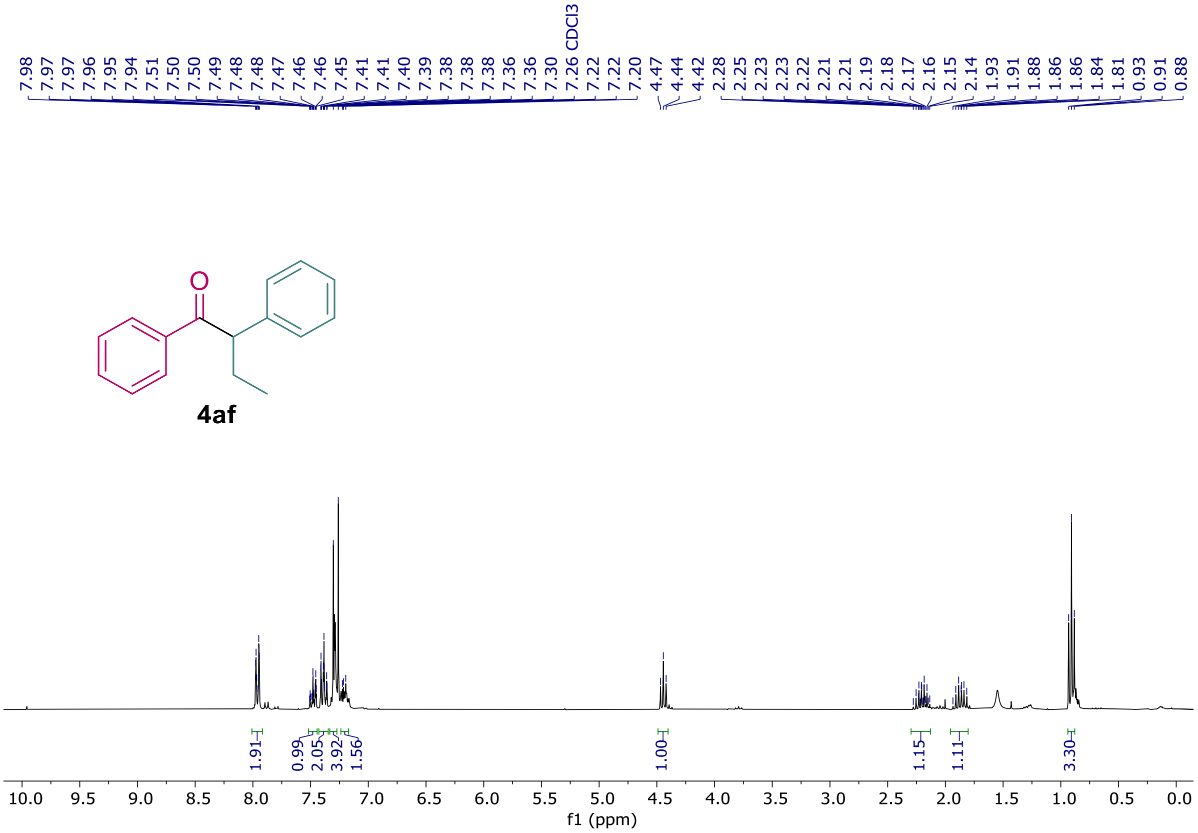

Figure S99: ^1H NMR spectrum of **4af** in $CDCl_3$ (300 MHz)

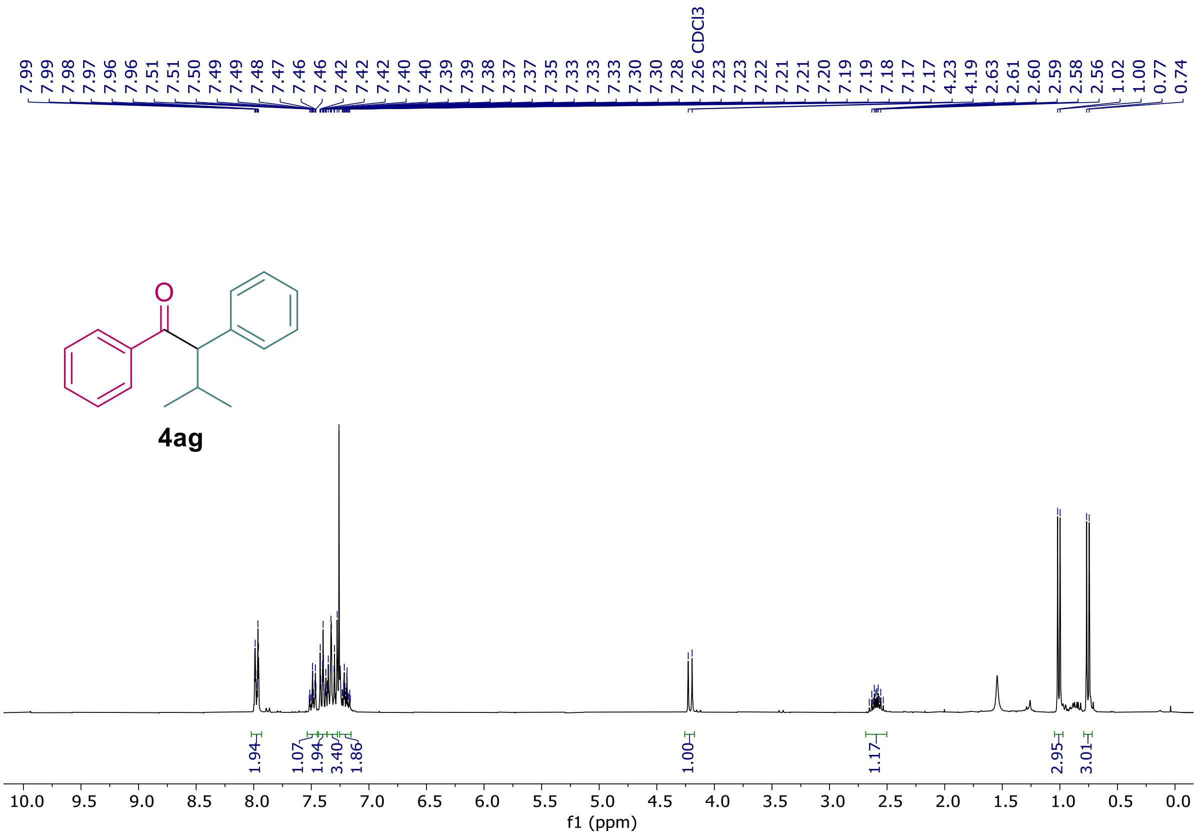

Figure S100: ^{1}H NMR spectrum of **4ag** in $CDCl_3$ (300 MHz)

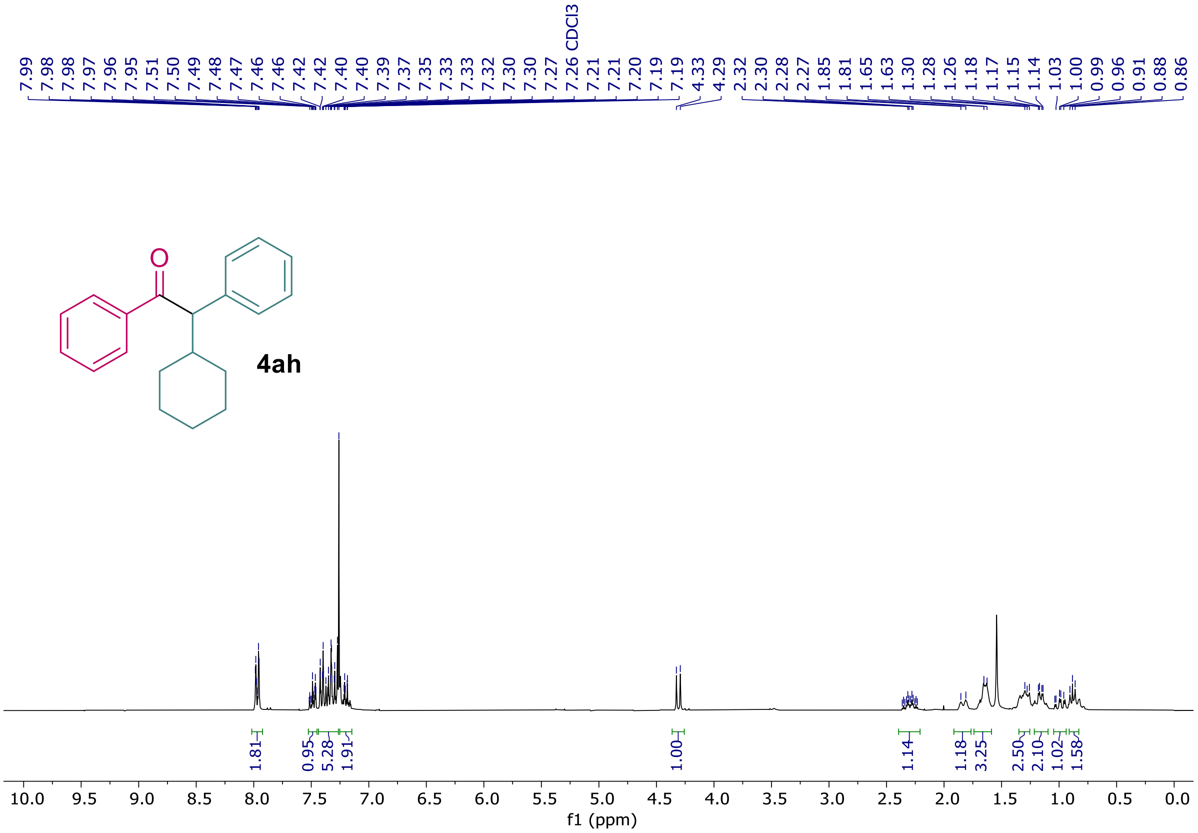

Figure S101: ^{1}H NMR spectrum of **4ah** in $CDCl_3$ (300 MHz)

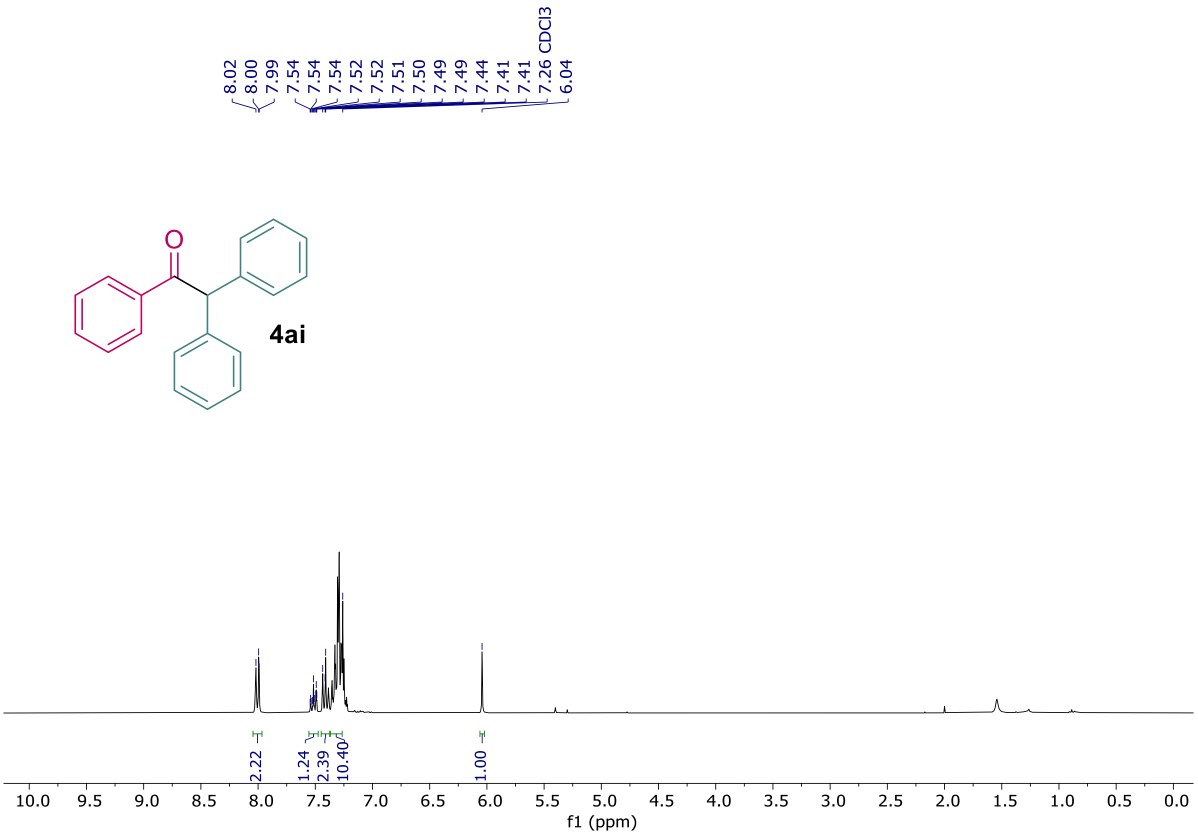

Figure S102: ^{1}H NMR spectrum of **4ai** in $CDCl_3$ (300 MHz)

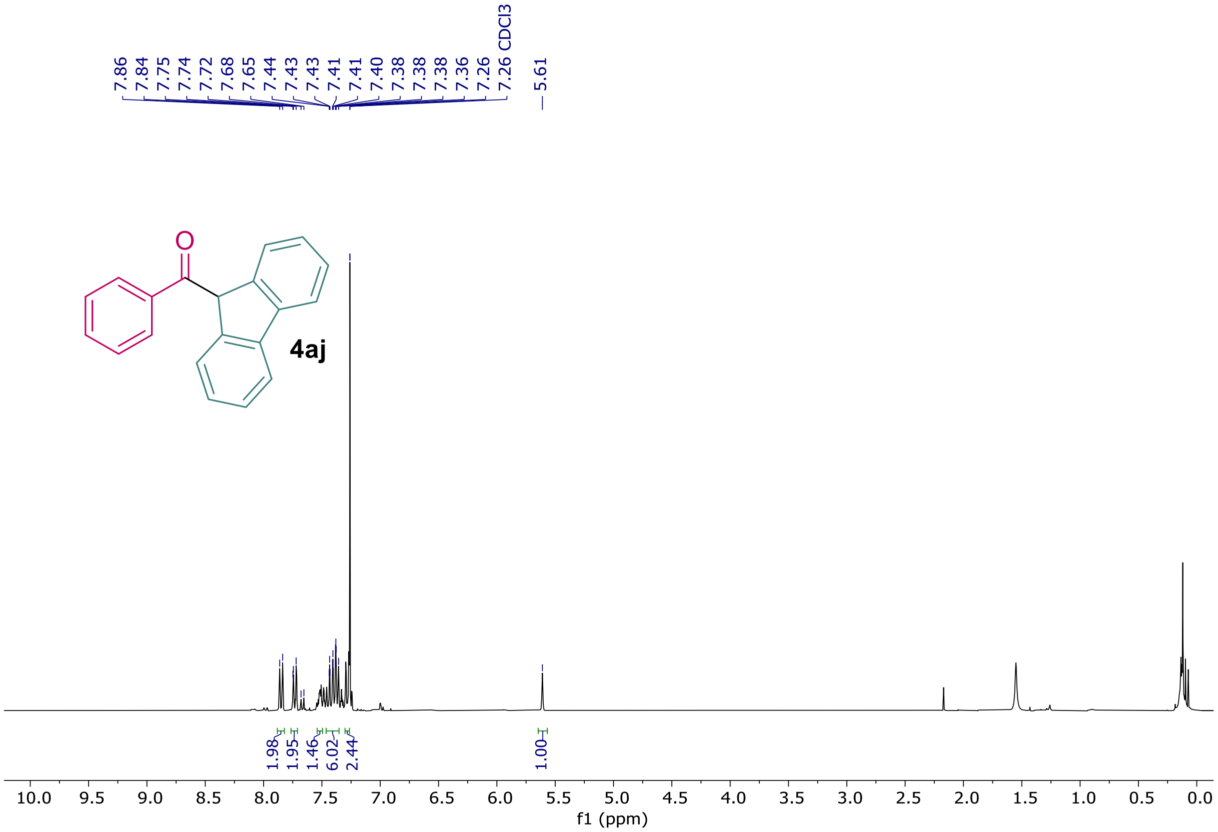

Figure S103: ^{1}H NMR spectrum of **4aj** in $CDCl_3$ (300 MHz)

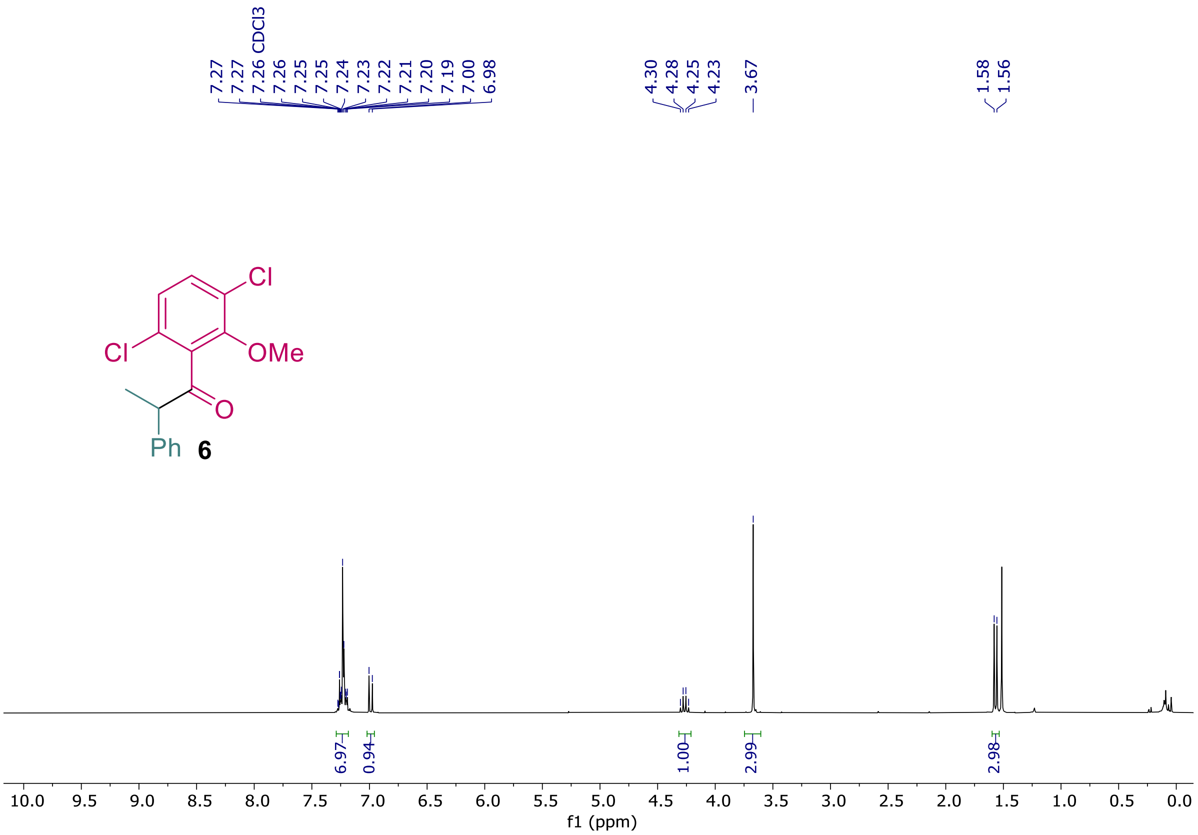

Figure S104: ^{1}H NMR spectrum of **6** in $CDCl_3$ (300 MHz)

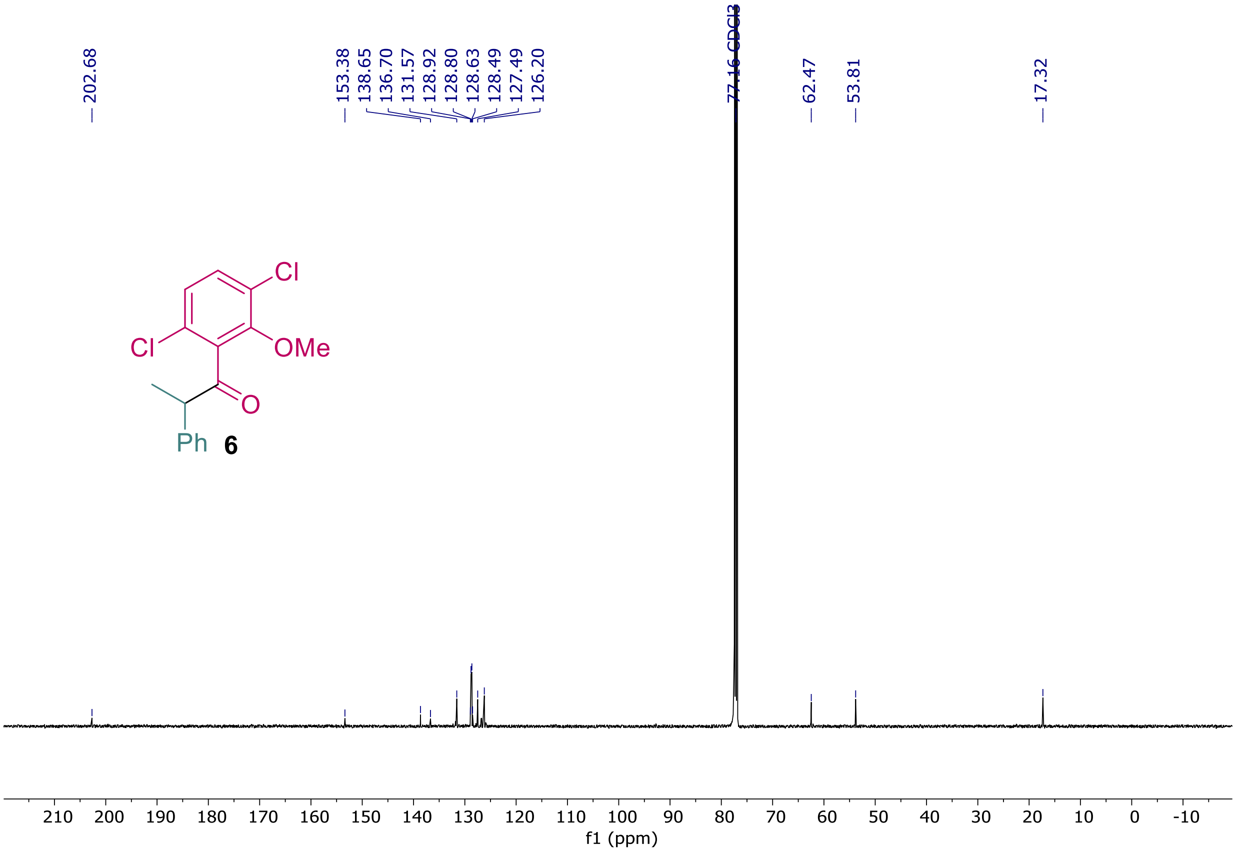

Figure S105: $^{13}C\{^{1}H\}$ NMR spectrum of **6** in $CDCl_3$ (125 MHz)

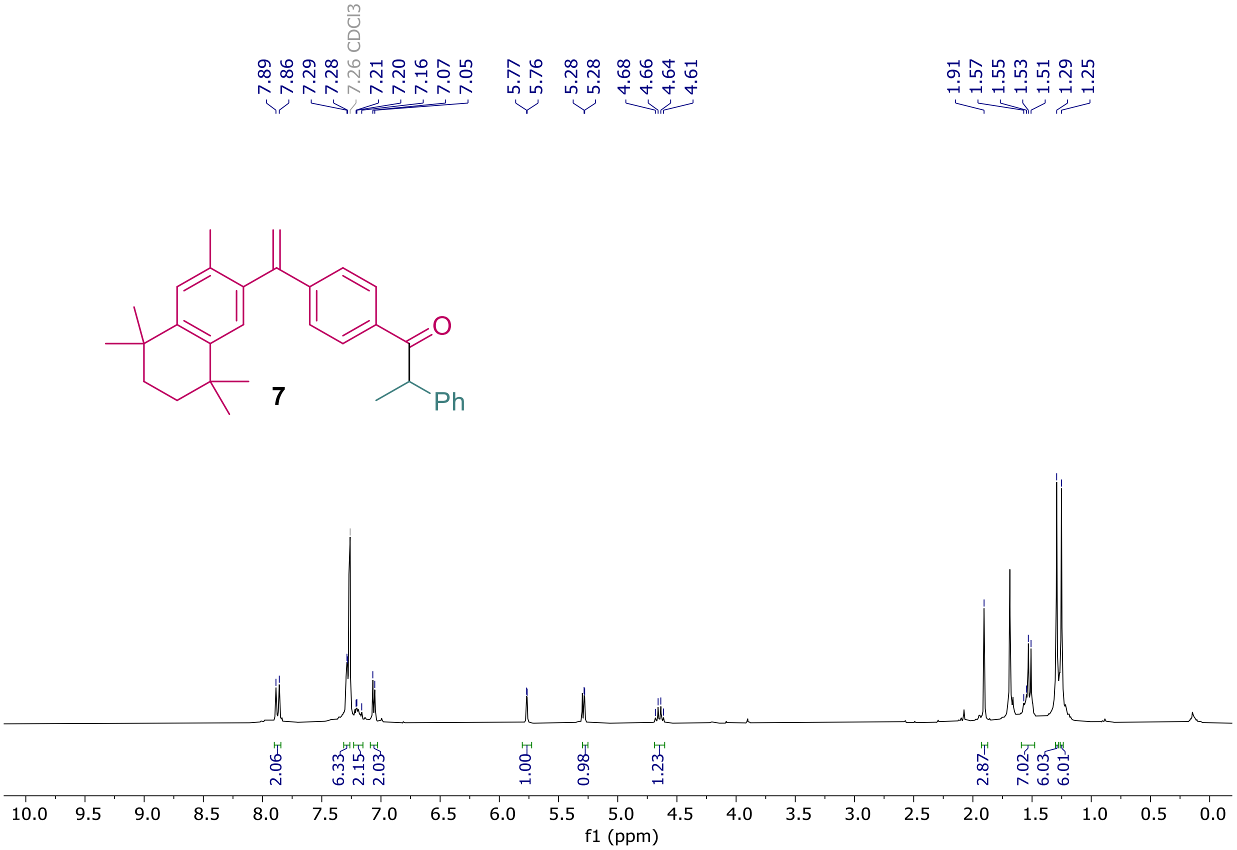

Figure S106: ^{1}H NMR spectrum of **7** in $CDCl_3$ (300 MHz)

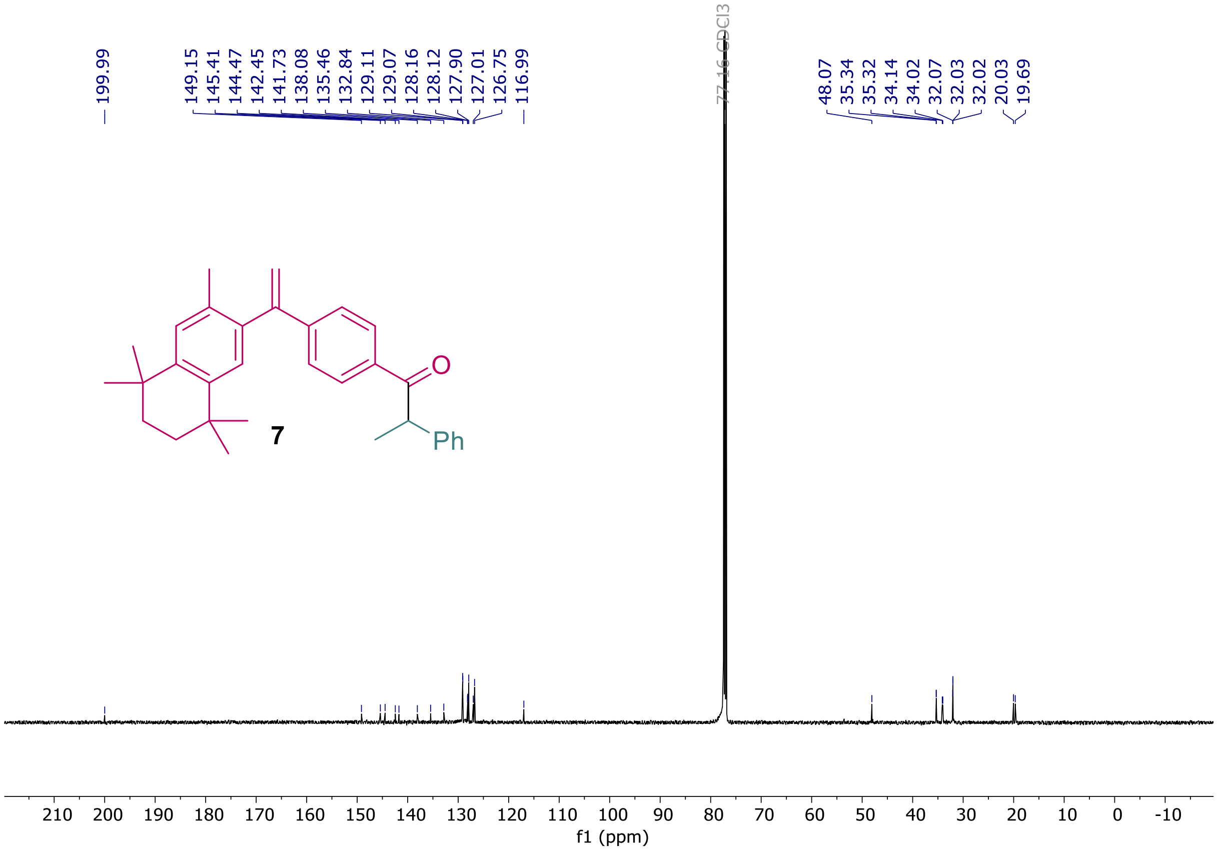

Figure S107: $^{13}C\{^{1}H\}$ NMR spectrum of **7** in $CDCl_3$ (125 MHz)

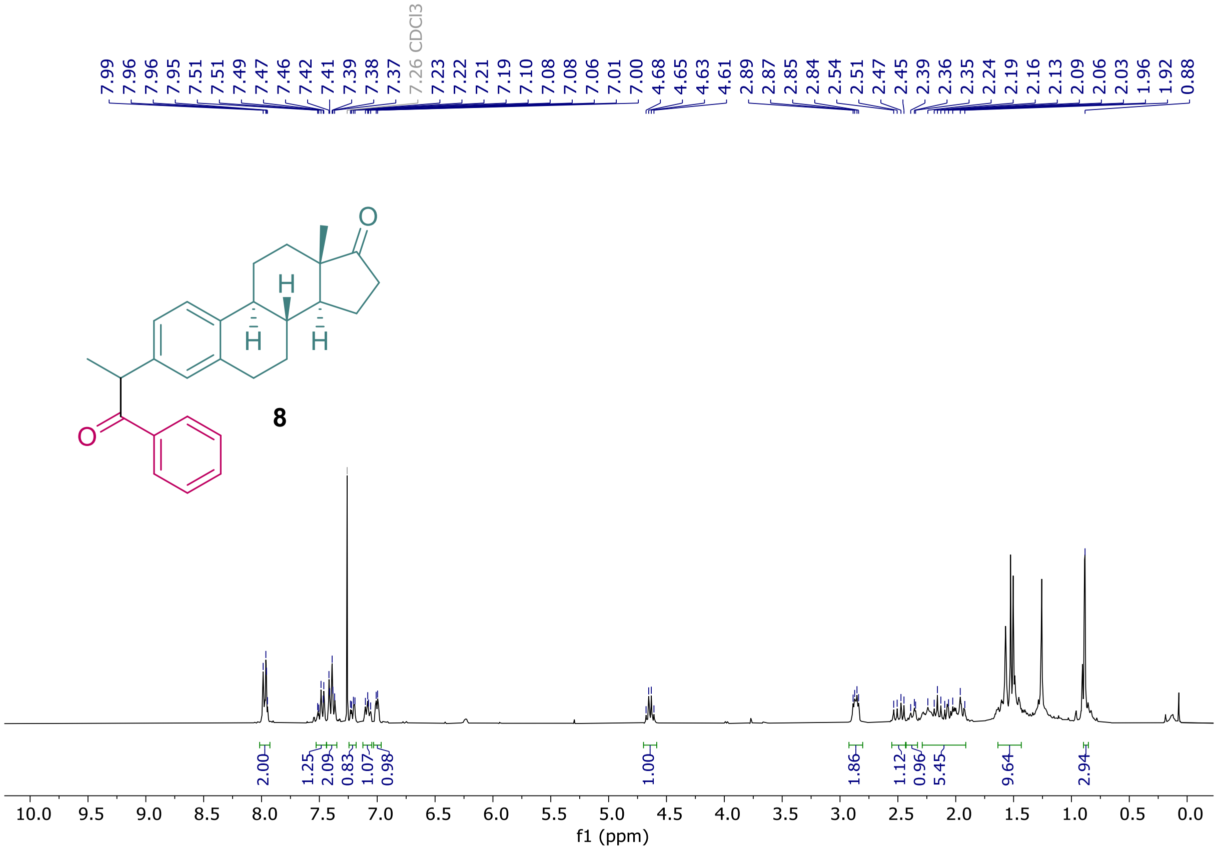

Figure S108: ^{1}H NMR spectrum of **8** in $CDCl_3$ (300 MHz)

8. References

References

1. Kutta, R.; Langenbacher, T.; Kensy, U.; Dick, B. Setup and performance of a streak camera apparatus for transient absorption measurements in the ns to ms range. *Applied Physics B* **2013**, *111*, 203–216.

2. Kutta, R. J. -Blitzlichtphotolyse- Untersuchung zu LOV-Dom\anen und photochromen Systemen. **2013**.

3. Zhu, J. L.; Schull, C. R.; Tam, A. T.; Rentería-Gómez, Á; Gogoi, A. R.; Gutierrez, O.; Scheidt, K. A. Photoinduced Acylations Via Azolium-Promoted Intermolecular Hydrogen Atom Transfer. *J. Am. Chem. Soc.* **2023**, *145*, 1535–1541.

4. Yin, D.; Lu, L.; Dou, Y.; Li, S.; Fu, M.; Zhu, Y.; Fan, S. 1,2-Acylphosphinylation of Styrenes to Access β-Aryl-γ-ketophosphine Oxides by Irradiation-Induced Radical Relay. *J. Org. Chem.* **2025**, *90*, 3848–3861.

5. Mahato, S. K.; Chatani, N. The Iridium(III)-Catalyzed Direct C(sp2)– and C(sp3)–H Alkynylation of 2-Acylimidazoles with Various Alkynyl Bromides: Understanding the Full Catalytic Cycle. *ACS Catal.* **2020**, *10*, 5173–5178.

6. Rourke, M. J.; Wang, C. T.; Schull, C. R.; Scheidt, K. A. Acyl Azolium–Photoredox-Enabled Synthesis of β-Keto Sulfides. *ACS Catal.* **2023**, *13*, 7987–7994.

7. Wicker, G.; Zhou, R.; Schoch, R.; Paradies, J. Sigmatropic [1,5] Carbon Shift of Transient C3 Ammonium Enolates. *Angew. Chem. Int. Ed.* **2022**, *61*, e202204378.

8. Holz, J.; Pfeffer, C.; Zuo, H.; Beierlein, D.; Richter, G.; Klemm, E.; Peters, R. In Situ Generated Gold Nanoparticles on Active Carbon as Reusable Highly Efficient Catalysts for a C −C Stille Coupling. *Angew. Chem. Int. Ed.* **2019**, *58*, 10330–10334.

9. Semenya, J.; Yang, Y.; Picazo, E. Cross-Electrophile Coupling of Benzyl Halides and Disulfides Catalyzed by Iron. *J. Am. Chem. Soc.* **2024**, *146*, 4903–4912.

10. Salaverri, N.; Mas-Ballesté, R.; Marzo, L.; Alemán, J. Visible light mediated photocatalytic [2 + 2] cycloaddition/ring-opening rearomatization cascade of electron-deficient azaarenes and vinylarenes. *Communications Chemistry* **2020**, *3*, 132.

11. Zhang, L.; Liu, B.; Lin, H.; Liang, M.; Huang, X. China National Intellectual Property Administration Patent CN110627610A, 2019.

12. Pichette Drapeau, M.; Fabre, I.; Grimaud, L.; Ciofini, I.; Ollevier, T.; Taillefer, M. Transition-Metal-Free α-Arylation of Enolizable Aryl Ketones and Mechanistic Evidence for a Radical Process. *Angew. Chem. Int. Ed.* **2015**, *54*, 10587–10591.

13. Schedler, M.; Wang, D.; Glorius, F. NHC-Catalyzed Hydroacylation of Styrenes. *Angew. Chem. Int. Ed.* **2013**, *52*, 2585–2589.

14. Lindner, H.; Carreira, E. M. Cobalt-Catalyzed Photo-Semipinacol Rearrangement of Unactivated Allylic Alcohols. *Angew. Chem. Int. Ed.* **2024***, 63*, e202407827.

15. Guo, Y.; Qi, J.; Guo, H.; Liu, R.; Zhou, R. Cross-Coupling of Benzylic and Aldehydic C–H Bonds via Photocatalytic Tandem Radical–Radical Coupling and Acceptorless Alcohol Dehydrogenation. *J. Org. Chem.* **2024***, 89*, 2032–2038.

16. Lunic, D.; Vystavkin, N.; Qin, J.; Teskey, C. J. Dual-Catalytic Structural Isomerisation as a Route to α-Arylated Ketones. *Angew. Chem. Int. Ed.* **2024***, 63*, e202409388.

17. Hong, Y.; Barchuk, A.; Krische, M. J. Branch-Selective Intermolecular Hydroacylation: Hydrogen-Mediated Coupling of Anhydrides to Styrenes and Activated Olefins. *Angewandte Chemie International Edition* **2006***, 45*, 6885–6888.

18. Templ, J.; Schnürch, M. Selective α-Methylation of Aryl Ketones Using Quaternary Ammonium Salts as Solid Methylating Agents. *J. Org. Chem.* **2022***, 87*, 4305–4315.

19. Kim, J.; Jang, J.; Lee, Y.; Shin, K. Exogenous Ligand-Free NiH-Catalyzed Hydroacylation of Aryl Alkenes with Aroyl Fluorides. *Org. Lett.* **2022***, 24*, 5412–5416.

20. Singh, R.; Nolan, S. P. An efficient and mild protocol for the α-arylation of ketones mediated by an (imidazol-2-ylidene)palladium(acetate) system. *Journal of Organometallic Chemistry* **2005***, 690*, 5832–5840.

21. Liu, F.; Hu, Y.; Li, D.; Zhou, Q.; Lu, J. N-Heterocyclic carbene-palladacyclic complexes: synthesis, characterization and their applications in the C-N coupling and α-arylation of ketones using aryl chlorides. *Tetrahedron* **2018***, 74*, 5683–5690.

22. Liu, J.; Tian, Z.; Wu, Z.; Huang, T.; Lin, Z.; Zhang, L.; Chen, J.; Hai, L.; Guo, L.; Wu, Y. Access to Ketones via Nickel-Catalyzed Coupling between S-2-Pyridyl Thioesters and Redox-Active Esters Using an Organic Reductant. *J. Org. Chem.* **2024***, 89*, 17059–17068.

23. Venkatesh, R.; Singh, A. K.; Lee, Y. R.; Kandasamy, J. Palladium-catalyzed synthesis of α-aryl acetophenones from styryl ethers and aryl diazonium salts via regioselective Heck arylation at room temperature. *Org. Biomol. Chem.* **2021***, 19*, 7832–7837.

24. Nguyen, T. H.; Bosse, A. T.; Ly, D.; Suarez, C. A.; Fu, J.; Shimabukuro, K.; Musaev, D. G.; Davies, H. M. L. Diaryldiazoketones as Effective Carbene Sources for Highly Selective Rh(II)-Catalyzed Intermolecular C–H Functionalization. *J. Am. Chem. Soc.* **2024***, 146*, 8447–8455.

25. Li, Q.; Long, Y.; Tao, Q.; Jin, Z.; Yan, X.; Zhou, X. Palladium-Catalyzed C(O)–C Bond Cleavage of Unstrained Ketones Assisted with Aryl Handles: An Approach to Diaryl Ketones. *Org. Lett.* **2025***, 27*, 2758–2763.

26. Anderson, D. E.; Tortajada, A.; Hevia, E. Highly Reactive Hydrocarbon Soluble Alkylsodium Reagents for Benzylic Aroylation of Toluenes using Weinreb Amides. *Angew. Chem. Int. Ed.* **2023***, 62*, e202218498.

27. Zhang, G.; Feng, B.; Wang, Y.; Chen, J.; Ma, X.; Song, Q. 1,1-Oxycarbonation of Terminal Alkynes via Sequential Borylation, 1,2-Migration, and Oxidation with Oxone. *Org. Lett.* **2024***, 26*, 3109–3113.

28. Li, B. X.; Le, D. N.; Mack, K. A.; McClory, A.; Lim, N.; Cravillion, T.; Savage, S.; Han, C.; Collum, D. B.; Zhang, H.; Gosselin, F. Highly Stereoselective Synthesis of Tetrasubstituted Acyclic All-Carbon Olefins via Enol Tosylation and Suzuki–Miyaura Coupling. *J. Am. Chem. Soc.* **2017**, *139*, 10777–10783.

29. Wang, J.; Shen, X.; Chen, X.; Bao, Y.; He, J.; Lu, Z. Cobalt-Catalyzed Enantioconvergent Negishi Cross-Coupling of α-Bromoketones. *J. Am. Chem. Soc.* **2023**, *145*, 24958–24964.

30. Zhang, Z.; Li, J.; Cai, Z.; Kang, S.; Wang, J.; Cui, Y.; Han, S.; Sheng, L.; Yin, Q.; Dai, A.; Zhao, W.; Zhao, F. Electrochemical aerobic Wacker-type oxygenation of triaryl substituted alkenes to 1,2,2-triarylethanones. *Chem. Commun.* **2024**, *60*, 3035–3038.

31. Zou, D.; Gan, L.; Yang, F.; Wang, J.; Li, L.; Li, J. Selective transition metal-free aroylation of diarylmethanes with 2-acyl-imidazolium salts via acyl C–C bond cleavage. *Tetrahedron Lett.* **2020**, *61*, 152532.

32. Ueda, Y.; Iwai, T.; Sawamura, M. Nickel-Copper-Catalyzed Hydroacylation of Vinylarenes with Acyl Fluorides and Hydrosilanes. *Chem. Eur. J.* **2019**, *25*, 9410–9414.

33. Guillaumont, D.; Bazin, H.; Benech, J.; Boyer, M.; Mathis, G. Luminescent Eu(III) and Gd(III) Trisbipyridine Cryptates: Experimental and Theoretical Study of the Substituent Effects. *ChemPhysChem* **2007**, *8*, 480–488.

34. Sivaraj, C.; Maiti, D.; Gandhi, T. Photo-Catalyzed Acyl Azolium Promoted Selective α-C(sp3)−H Acylation of Acetone via HAT: Access to Thermodynamically Less Favoured (Z)-α,β-Unsaturated Ketones. *Chem. Eur. J.* **2024**, *30*, e202303626.

35. Liu, X.; Wang, J.; Tong, Y.; Song, Q. Regioselectivity and Competition of the Paternò–Büchi Reaction and Triplet–Triplet Energy Transfer between Triplet Benzophenones and Pyrimidines: Control by Triplet Energy Levels. *Chem. Eur. J.* **2013**, *19*, 13216–13223.

36. Bachrach, R. Z. A Photon Counting Apparatus for Kinetic and Spectral Measurements. *Rev. Sci. Instrum.* **1972**, *43*, 734–737.

37. Gilmore, E. H.; Gibson, G. E.; McClure, D. S. Absolute Quantum Efficiencies of Luminescence of Organic Molecules in Solid Solution. *J. Chem. Phys.* **1952**, *20*, 829–836.

38. Bensasson, R. V.; Gramain, J. Benzophenone triplet properties in acetonitrile and water. Reduction by lactams. *J. Chem. Soc. , Faraday Trans. 1* **1980**, *76*, 1801–1810.

39. Porter, G.; Wilkinson, F. Primary photochemical processes in aromatic molecules. Part 5.—Flash photolysis of benzophenone in solution. *Trans. Faraday Soc.* **1961**, *57*, 1686–1691.

40. Koroli, L. L.; Kuzmin, V. A.; Khudyakov, I. V. Kinetics of recombination, dismutation, and disproportionation reactions involving neutral ketyl radicals and radical anions. *Int J Chem Kinet* **1984**, *16*, 379–396.
